# Transdermal delivery of botulinum toxin-A through phosphatidylcholine/cholesterol nanoliposomes for treatment of post-acne scarring

**DOI:** 10.1007/s10856-024-06810-1

**Published:** 2024-07-29

**Authors:** Lannan Chen, Lei Cui, Jiabing Ran, Zhengrui Liu, Xiongbin Zhu

**Affiliations:** 1 Department of Medical Cosmetology, The First Clinical Medical College of China Three Gorges University, Yichang Central People’s Hospital, Institute of Medical Cosmetology, China Three Gorges University, Yichang, China; 2 College of Biological & Pharmaceutical Sciences, China Three Gorges University, Yichang, China; 3 Hubei Key Laboratory of Natural Products Research and Development, China Three Gorges University, Yichang, China

## Abstract

As an acne sequela, post-acne scarring (PSA) has huge negative impact on sufferers’ quality of life because of aesthetical embarrassment. Transdermal delivery of botulinum toxin-A (BTXA) is a promising strategy for PAS treatment, but currently reported approaches are far from satisfactory. In this work, phosphatidylcholine/cholesterol (PC/Chol) nanoliposomes were utilized for encapsulation and transdermal delivery of BTXA. The composition, structure, morphology, size, size distribution, etc. of as-prepared BTXA@liposome nanoparticles were investigated in detail. Simulated transdermal delivery assay indicated that the diffusion depth of the BXTA@liposome nanoparticles was nearly 8 times that of pure BTXA and reached 380 μm. 12 facial PSA patients were recruited to evaluate the curative effect of the BTXA@liposome nanoparticles on PSA. Through ECCA (échelle d’évaluation clinique des cicatrices d’acné) scoring and self-evaluation of patients, the resultant data indicated that compared to hyaluronic acid (HA) hydrogel treatment the BTXA@liposome/HA hydrogel treatment could better relieve PSA to some extent but didn’t show significant advantage. Further work is needed to verify the feasibility and curative effect of this method in PSA treatment in the future.

**Graphical Abstract:**

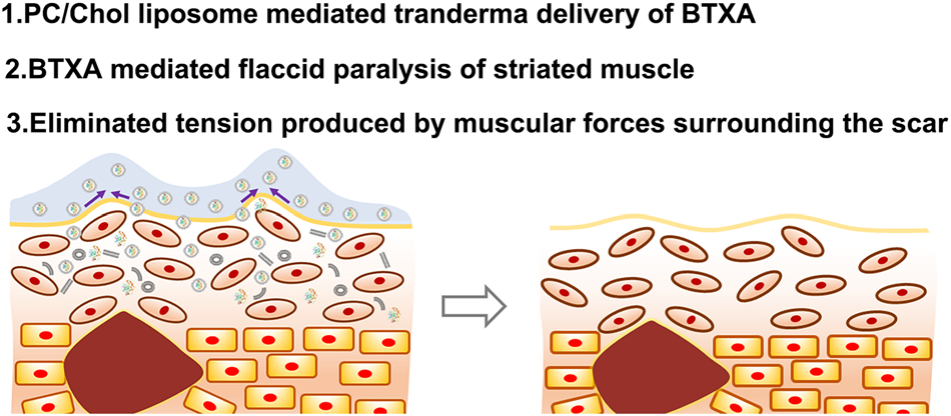

## Introduction

PAS, a distressing acne sequela featuring contour abnormality (hypertrophy or atrophy) and color change (red, white, or brown) of skin, was recorded in 14% of women and 11% of men [1]. PAS could be roughly categorized into superficial macules, dermal troughs, ice picks, multi-channeled fistulous tracts and subcutaneous atrophy on basis of the extent of severity [2, 3]. Owing to aesthetical embarrassment, patients with facial PAS commonly suffer from severe self-consciousness and difficulties in social activities (e.g., poor employment prospects) [4]. Especially, facial PSA has been reported to be an “at risk” factor for suicide of adolescents [5]. However, conventional treatment strategies are far from satisfactory [1]. For instance, the curative effect of dermabrasion and punch techniques highly depends on specific operator [6, 7]; the degree of long-term improvement of chemical peeling and rhytidectomy is insufficient to satisfy most patients [8, 9]; CO_2_/erbium laser resurfacing is restricted by its high cost [10]; scars undermining or dermal grafting may cause some undesired complications (e.g., excessive response for the former and cyst formation for the latter) [11]. In recent years, in situ implanting tissue augmenting agents for PAS treatment has drawn tremendous attention, either. However, autologous filler agents (e.g., fat) are contentious while non-autologous counterparts (e.g., collagen and silicon) still need further validation [2]. Thus, developing advanced techniques/biomaterials to realize simple, safe, and cost-efficient PAS treatment is of vital importance.

BTXA, a potent neurotoxin derived from *Clostridium botulinum*, could cause flaccid paralysis of striated muscle by inhibiting acetylcholine release at the neuromuscular junction [12], therefore eliminating the tension produced by muscular forces surrounding the scar [13]. In addition, BTXA has also been reported to have an inhibitory effect on fibroblasts and collagen remodeling activity [14]. Recently, some researchers have found that BTXA probably has an anti-inflammatory effect in acne [15]. Thus, BTXA holds great promise for PAS reduction. However, BTXA is charged in physiological pH and its molecular weight is as high as 70 kDa, so it is impossible for it to diffuse across the skin spontaneously [16–18]. In clinic, injection has been successfully utilized for transdermal delivery of BTXA [19]. Since BTXA occupies the first place among the most dangerous poisons (toxins) for human health and life, its injection must be operated by a professional physician so as not to undermine deeper muscular activity, which, to some extent, increases the time cost of this method [13, 15]. In addition, BTXA is commonly injected together with hemagglutinin. Such a combination increases the risk of neutralizing antibodies formation, which may lead to manifestation of secondary resistance [15]. Thus, developing non-invasive transdermal delivery system of BTXA is in urgent need for PAS patients.

Stratum corneum (SC) is the main physical barrier preventing BTXA from entering the deeper layers of the skin [17, 18]. SC comprises of corneocytes surrounded by hydrophobic and insoluble keratins. Ceramides, free fatty acids, and cholesterol constitute the intercellular lipid matrix within the SC, which assumes a lamellar structure. These lamellae are connected in a repeatedly stratified structure, forming the barrier to suppress the permeation of hydrophilic substance [20]. Until now, much work has been done to achieve non-invasive transdermal delivery of large-sized proteins/peptides [21, 22]. For instance, iontophoresis/ultrasound-mediated transdermal delivery technique [18, 23], ionic liquid aided transdermal delivery technique [24, 25], microneedle patch based transdermal delivery technique [26, 27], laser-assisted transdermal delivery technique [18], electroporation [28], thermal and radiofrequency ablation [28], sonophoresis/phonophoresis [18], jet injection [18], etc. Nevertheless, whether these techniques are suitable for transdermal delivery of BTXA or not still need further validation. Ebrahim et al. corroborated that microneedling delivery of BTXA could be a simple and safe way to improve the appearance and decrease the depth of atrophic acne scars [19, 29]. The same conclusion was drawn in the work of Albalat et al., who utilized BTXA coated microneedling combined with platelet-rich plasma for the treatment of atrophic acne scars [14]. However, sterility of microneedles for clinical applications is a valid concern. Moreover, following microporation of microneedles, duration of time taken for the pores to close is also an important concern as it may cause irritation and infection at the site [18].

**Fig. 1:**
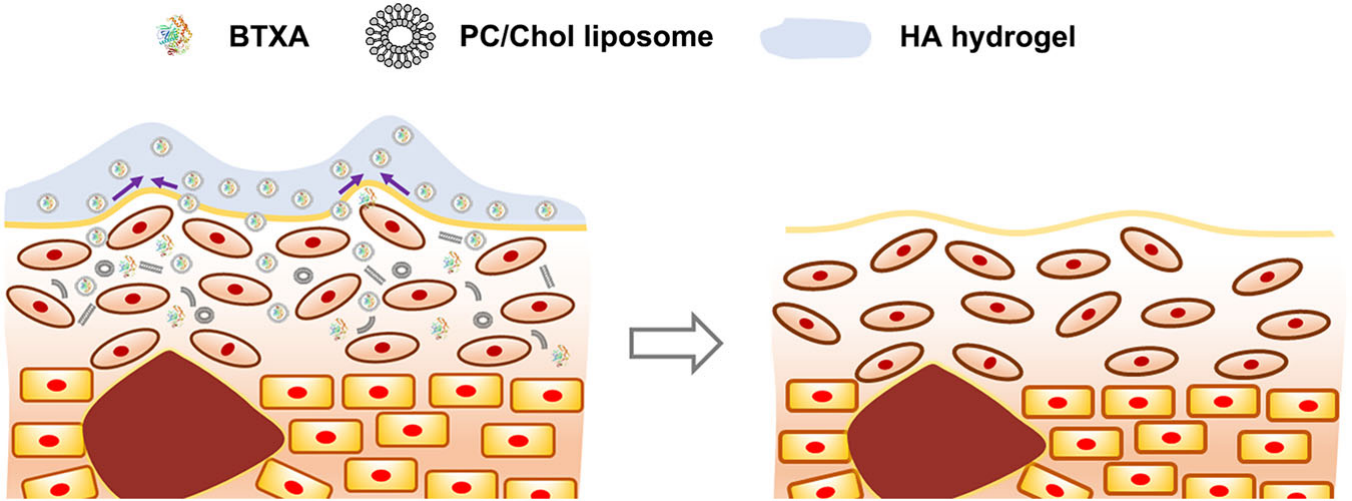
Schematic diagram of transdermal delivery of the BTXA@liposome nanoparticles for PSA treatment

Liposome-based nanoparticles are another biocompatible carrier for non-invasive transdermal delivery of both hydrophobic and hydrophilic compounds and has drawn increasing attention [21, 30]. Lipid based carrier could easily penetrate skin owing to the presence of epidermal lipids as the chief component within the penetration barrier in high amount. Besides, skin-carrier interaction involves attachment of these carriers to skin with a view to permit exchange of lipid between the outermost layers of the stratum corneum [31]. Foldvari et al. have successfully utilized lipid-based delivery system for transdermal delivery of interferon α (molecular weight 19 kDa) [32]. In this regard, we encapsulated BTXA into PC/Chol liposome (termed BTXA@liposome) and utilized it for facial PSA treatment. Figure 1 shows the schematic diagram of transdermal delivery of the BTXA@liposome nanoparticles for PSA treatment. The composition, structure, size, morphology, and encapsulation efficiency etc. of the BTXA@liposome nanoparticles were investigated in detail. Especially, the bioactivity of BTXA within the lipid and the penetration capability of the BTXA@liposome into skin were evaluated, either. Several volunteers with facial PSA were firstly screened and then invited to estimate the effect of the BTXA@liposome nanoparticles in PSA treatment. This work, we believe, will shed some light on clinical treatment of PSA in the future.

## Experimental section

### Materials

Phosphatidylcholine (from soybean, >98%), cholesterol (cholesterol, >95%), PBS buffer (PBS buffer, 1×, pH7.2–7.4, sterile enzyme-free), trehalose (≥95%), sodium hyaluronate (HA, >99.0%), carbomer (carbomer 934), sodium chloride (≥99.5%) were purchased from *Macklin Co., Ltd. (Shanghai, China)*. Sucrose fatty acid ester (monoester content 55%, HLB value 11) was purchased from *Aladdin Chemical Reagent Co., Ltd. (Shanghai, China)*. BSA-FITC (BSA ≈ 5 mg/mL) was purchased from *Solarbio Co., Ltd. (Beijing, China)*. All reagents are as used and not purified. Ultrapure water was used throughout the experiment.

### Fabrication of the BTXA@liposome and FITC-BSA@liposome nanoparticles

96 mg phosphatidylcholine was dissolved in 24 mL absolute ethanol through ultrasound for 45 s (solution A). Then, 48 mg cholesterol was dissolved in the solution A through ultrasound for 25 s. The resultant mixed solution was called solution B. Next, 24 mL PBS was dripped into the above solution B (30 drops/min). Afterwards, the as-obtained solution was subjected to ultrasound for 30 s and then rotation evaporation at 40 °C for 30 min to remove ethanol. The resultant solution was mixed with 30 mg trehalose and 10 mg of sucrose fatty acid lipid through ultrasound for 45 s. As a resultant, the blank PC/Chol liposome was obtained. The blank liposome suspension was co-incubated with 100 U/mL botulinum toxin solution and subjected to ultrasound for 45 s to prepare the BTXA@liposome suspension. The fabrication process of the FITC-BSA@liposome was nearly the same with that of the BTXA@liposome, except that BXTA was replaced with FITC-BSA. To determine the encapsulation efficiency, Sephadex G-75 gel column was used to separate liposomes and free drugs. 0.02 mol/L PBS was utilized for elution at 1 mL/min, the eluate was collected in sections, and the botulinum toxin content was determined by Coomassie brilliant blue G-250 staining method. The encapsulation efficiency was calculated using the following equation:

$${Encapsulation\; efficiency}\,\left( \% \right)=\left(1-\frac{{C}_{1}}{{C}_{{total}}}\right)\times 100 \%$$

Where C_total_ means the initial content of BTXA, C_1_ means the BTXA content after passing the column.

### Fabrication of the BTXA@liposome/HA and FITC-BSA/liposome/HA hydrogels

**Fig. 2:**
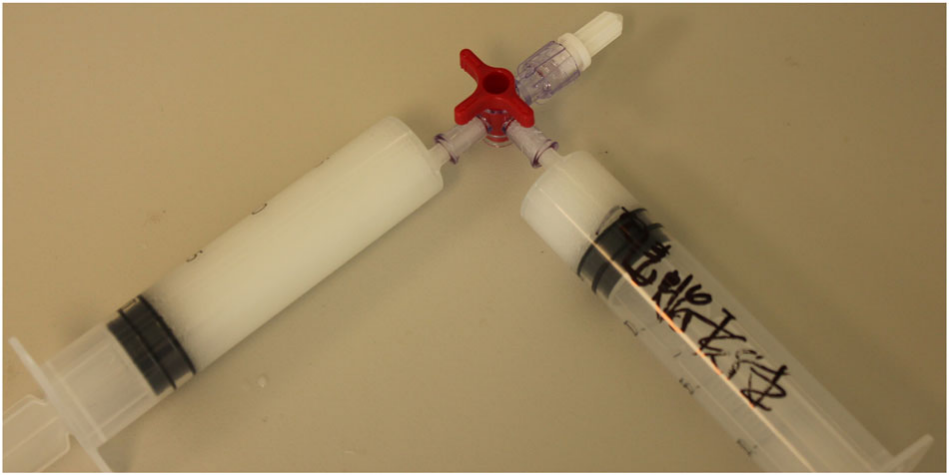
The device for the preparation of the BTXA@liposome/HA hydrogel and the FITC-BSA@liposome/HA hydrogel

1 mg/mL HA solution was supplemented with 9 mg/mL sodium chloride and 10 mg/mL carbomer 980 to prepare a colorless transparent gel. The prepared colorless transparent gel and BXTA@liposomes suspension were respectively installed in two 20 mL syringes, connected with a three-way connecting tube. Next, they were thoroughly mixed until there was no precipitation and flocculent to obtain the BTXA@liposome/HA hydrogel (Fig. 2). The fabrication procedure of the FITC-BSA@liposome/HA hydrogel was nearly the same with that of the BTXA@liposome/HA hydrogel, except the BTXA@liposome was replaced with the FITC-BSA@liposome.

### Characterizations of the BTXA@liposome nanoparticles

#### Fourier transform infrared spectrum (FT-IR)

FT-IR (*Nicolet 550 II, Thermo Fisher, USA*) was utilized to investigate the composition of the BTXA@liposome and the interaction between different functional groups. The BTXA@liposome was firstly grinded into powder than then subjected to FT-IR test by using a KBr method.

#### X-ray diffraction (XRD)

XRD (*Panalytical BV, Netherlands*) was utilized to investigate the crystal phases of the BTXA@liposome. The BTXA@liposome was firstly grinded into powder, and then subjected to XRD test. The working condition of XRD was CuK_0_ radiation via a rotation anode at 40 kV and 40 mA. The data were collected in a step pf 0.1° and a range of diffraction angles (2θ) from 5° to 90°.

#### BET surface area test

The BET surface area and BJH pore size distribution of the BTXA@liposome nanoparticles and PC/Chol liposome nanoparticles were determined using a BET analytical tester (*Novatouch, Quantachrome, USA*). The average pore diameter was given by the instrument.

#### Circular dichroism (CD) spectrum

To investigate the secondary structure of BTXA, CD spectra were recorded on a JASCO J-815 spectrometer (5 mm pathlength, CH_2_Cl_2_, 20 °C). Eight scans were averaged.

#### Dynamic light scattering (DLS)

To determine the particle size and particle size distribution of the PC/Chol liposome and the BXTA@liposome, a DLS instrument (*ZeataSizer Pro, Malvern Panalytical*) was applied. The temperature was set at 25 °C, the PC/Chol liposome and the BXTA@liposome was diluted 100-fold with pure water. The whole process was carried out without bubbles.

#### Zeta potential

The zeta potential was determined by a DLS instrument (*ZeataSizer Pro, Malvern Panalytical*) to verify the colloidal dispersion stability and isoelectric point of the PC/Chol liposome and the BXTA@liposome.

#### Scanning electron microscope (SEM) and transmission electron microscope (TEM)

SEM (*Sigma, Zeiss, Germany*) and TEM (JEM-2100, JOEL, Japan) were utilized to observe the morphology, structure, size, and size distribution the as-prepared BTXA@Liposome. After obtaining the SEM images, the size and size distribution of the BTXA@liposome nanoparticles were analyzed using Image J.

### Determination of the diffusion depth of the FITC-BSA@liposome nanoparticles within the pigskin

Since FITC-BSA and BTXA are both proteins and have nearly identical molecular weight, FITC-BSA was used as a substitute for BTXA. We selected a piece of fresh pork rind and wipe the surface with an alcohol cotton pad to remove the surface grease. Then the pork rind was subjected to fractional CO_2_ laser treatment and the corresponding parameters were as follows: deep mode, wavelength 10600 nm, density 11.1%, energy 40 mJ/cm^2^, spot size 18 × 18 mm, number of repetitions 1 time, sweep the 1 × 1 cm^2^ area. 1 mL of FITC-BSA@ liposome suspension or FITC-BSA solution were added dropwise to the 1 × 1 cm^2^ area. Three replicates were carried out for each group. Finally, frozen sections were prepared and the longitudinal diffusion depth of the FITC-BSA@liposome and the FITC-BSA was determined using a immunofluorescence microscopy.

### Clinical experiment

#### Clinical samples

This is a randomized, controlled, double-blind, split-plane clinical experiment approved by the China Three Gorges University Ethics Committee and informed consent was obtained from the patient prior to the study. We recruited 12 Chinese participants (four males, eight females) with a mean age of 25 years (age range 19 to 35 years). Prior to treatment, all patients had almost the same acne Pillsbury 4 scale and overall Goodman and Baron qualitative global scar grading system (QGS) and ECCA qualitative scar scoring systems on both faces. No other acne treatments were conducted in the entire study or follow-up period. All patients signed a written informed consent form prior to treatment. Ethics Approval No. (CLA2022/CLAA2022/CLLA2022).

#### Criteria for inclusion for the study

Twelve participants with different types and grades of acne scars were selected to participate the study (according to QGS), patients aged over 18 years with facial acne scars were included in the study.

#### Criteria for exclusion for the study

(1) active acne scarring; (2) skin infection; (3) patients who have undergone any facial cosmetic surgery, especially botulinum toxin injections within the past 6 months; (4) patients who are photosensitic or allergic to the preparations used, bleeding disorders.

#### Clinical study protocol

All patients were instructed to eat properly and develop good habits. 30 min before the start of treatment, compound lidocaine cream (*Tongfang Pharmaceutical, Guoyao Zhunzi: H20063466*) was used to administer topical anesthesia of the patient’s bilateral face. 40–60 min’ late, the anesthetic cream was washed off and wiped with an alcohol swab to disinfect. Both groups of patients were treated with ultra-pulsed fractional CO_2_ laser with the same energy. (2) The deep lattice mode was used to select the treatment area according to the size and shape of the skin lesion. Treatment parameters: Deep mode, wavelength 10,600 nm, density 11.1%, energy 40 mJ/cm^2^, spot size 18 × 18 mm, 1 repetition to sweep acne and acne scar area, pay attention to avoid eyes, mouth and nose during the operation. After the laser treatment, 10 mL of the BTXA@liposome/HA hydrogel was evenly applied to the lesion area on one side of the face, which was the test side (side A). On the other side, 10 mL of HA hydrogel should be applied for the control side (side B). Both the patient and the doctor had no idea about the contents of the syringe.

The clinical evaluation was done on basis of a serials of photographs recorded using a digital camera (*Canon EOS-6D, China)* at baseline and the end of the course of treatment. Two independent blinded investigators were used to analyze the treatment effects, calculate the ECCA score, and record the relevant indicators after treatment.

Criteria for judging the efficacy: (i) significant effect, persistent erythema after oily skin or facial acne has been improved, and the repair rate of scar appearance is more than 60%, which is close to normal skin tone; (ii) effective, the scar appearance repair rate is 30% ~ 60%; (iii) ineffective, no change in appearance, and the rate of scar appearance repair does not exceed 30%. Treatment response rate (%) = [(number of effective cases + number of effective cases)/total number of cases] × 100%. The scar status of patients was evaluated according to the Acne Scar Clinical Rating Scale (ECCA). Digital photography was used at baseline and 2 months post-treatment to assess efficacy. Two plastic surgeons who were not involved in the treatment rated the degree of acne scarring on both sides of the patient’s face using the ECCA rating scale and took the average of the two. The ECCA score is scored according to the type of depressed scar (icicle-shaped scar is 4 mm in length and diameter, with a wavy appearance), the degree, morphology, and development of skin lesions, with scores of 15, 20, 25, and 30; The b value measures the density of the patient’s scars (in terms of forehead or one cheek): 0 = no scars, 1 = a small number of scars (number of scars ≤5), 2 = a certain number of scars (5–20 scars); The A × B values were the final composite score of the scar site. This score quantitatively reflected the severity of acne scars to some extent. Patient satisfaction was evaluated on a quartile rating scale: grade 1 = 1 point (slight improvement: 0–25%), grade 2 = 2 points (moderate improvement: 26–50%), grade 3 = 3 points (significant improvement: 51–75%), grade 4 = 4 points (significant improvement: 76–100%). At the 2-month follow-up after the last treatment, two dermatologists who did not participate in the treatment were solicited and recorded on the quartile rating scale of patient self-evaluation. The adverse reactions and the incidence and duration of adverse reactions in the two groups were observed and recorded.

#### Statistical analysis

We used a one-way Kolmogorov–Smirnov (KS) test to assess the normal distribution of the study variables. Since the KS test values showed that the included variables did not follow a normal distribution, we used the Wilcoxon signed-rank test for numerical data analysis. The measurement data were expressed as all standard deviations (X ± S), and the t-test was used for comparison. Count data were expressed in (%), and the X2 test was used for comparison, and a *p*-value of less than 5% was considered statistically significant. We used SPSS *(v. 22, Chicago, IL)* as our analysis software.

## Results and discussions

### Successful fabrication of BTXA@Liposome nanoparticles

**Fig. 3:**
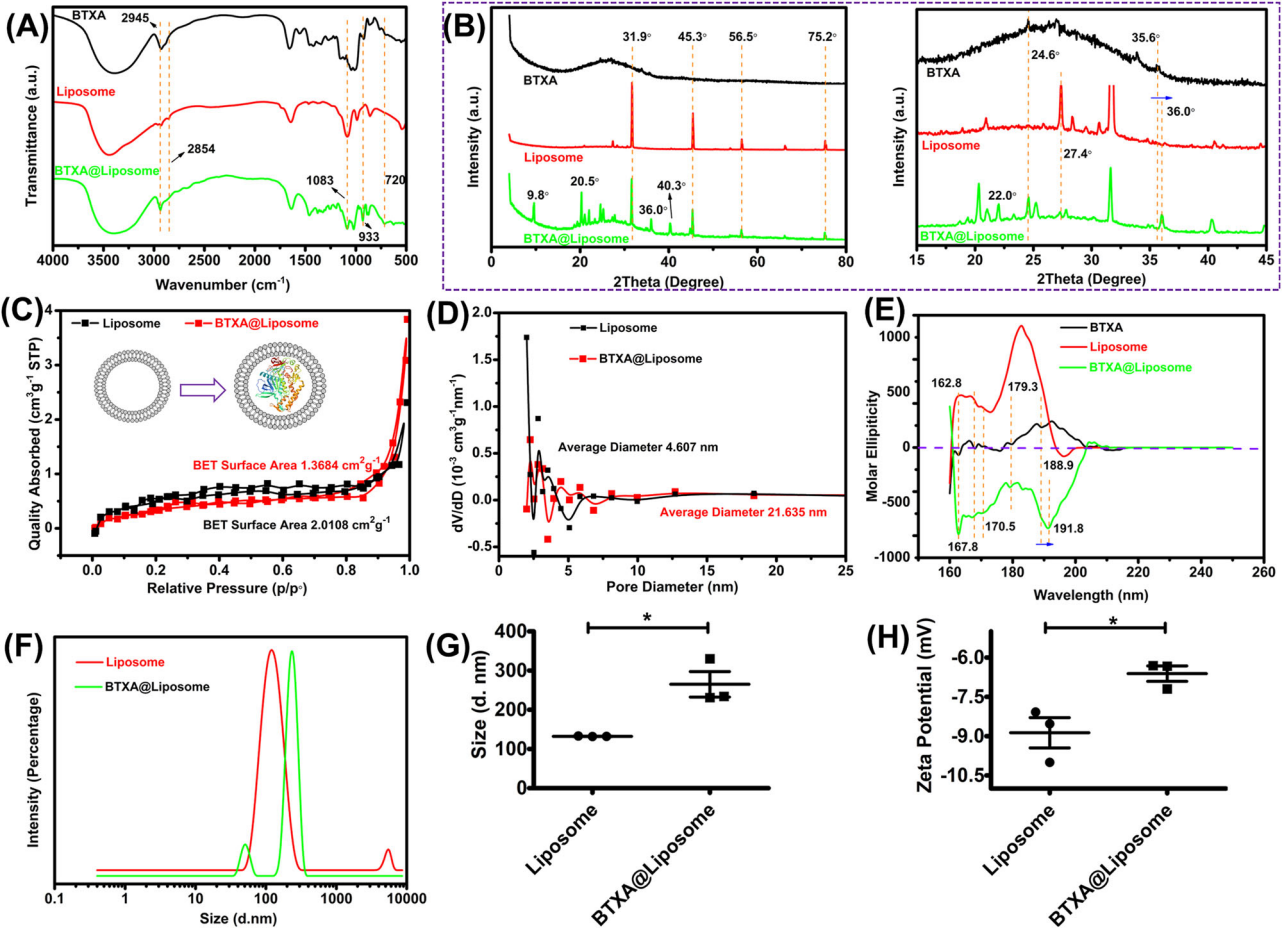
**A** FTIR spectra and (**B**) XRD spectra of BTXA, pure PC/Chol liposome, and the BTXA@liposome. **C** N_2_ adsorption and desorption isotherms of pure PC/Chol liposome and the BTXA@liposome. The insert in (**C**) shows the schematic diagram of encapsulation of BTXA within the PC/Chol liposome. **D** shows the BJH pore size distribution of the two samples. **E** CD spectra of BTXA, pure PC/Chol liposome, and the BTXA@liposome. **F** size distribution of pure PC/Chol liposome and the BTXA@liposome obtained from DLS. **G**, **H** show the average size and zeta potential of two nanoparticles

Figure 3A exhibits the FT-IR spectra of BTXA, pure PC/Chol liposome, and the BTXA@liposome. BXTA shows three representative absorbance peaks at 2945 cm^−1^, 933 cm^−1^, and 720 cm^−1^ while pure PC/Chol liposome shows two representative absorbance peaks at 2854 cm^−1^ and 1083 cm^−1^ [33, 34]. These peaks could also be found in the FT-IR spectrum of the BTXA@liposome, indicating the successful hybridization of BTXA and the PC/Chol liposome. The same conclusion could be drawn from the results of XRD (Fig. 3B). BTXA is a kind of organic compound and has no ordered arrangements in microscopic structure, so it doesn’t demonstrate sharp diffraction peaks just like inorganic crystal. However, at 24.6° and 35.6°, two weak diffractions peaks could be found in the XRD spectrum of BTXA. Owing to the order arrangement of the liposome, it showed obvious diffractions peaks at 31.9°, 45.3°, 56.5°, and 75.2°. Interest, the abovementioned peaks could also be found in the XRD spectrum of the BTXA@liposome, indicating the successful hybridization of BTXA and the liposome [35, 36]. In addition, the BTXA@liposome, compared to BTXA and pure PC/Chol liposome, also demonstrated five newly appeared peaks at 9.8°, 20.5°, 22.0°, 36.0°, and 40.3°. Besides, after hybridization, the intensity of diffraction peak at 27.4° of the PC/Chol liposome was greatly reduced, and the diffraction peak of BTXA at 35.6° was slightly moved to 36°. Here, we assumed that there might exist some intermolecular interactions (e.g., hydrogen bond and electrostatic interaction) between BTXA and the PC/Chol liposome, therefore affecting the ordered arrangement of both BTXA molecules and the PC/Chol liposome [37]. To figure out whether BTXA has been encapsulated by the PC/Chol liposome, nitrogen adsorption/desorption isotherms of pure PC/Chol liposome and the BTXA@liposome were tested and plotted in Fig. 3C. It could be found that the BET surface area of the PC/Chol liposome was significantly lower than that of the BTXA@liposome, indicating the successful loading of BXTA within the cavity of the PC/Chol liposome. Through analyzing the BJH pore size distribution (Fig. 3D), it could be found that the average pore diameter of the PC/Chol liposome was much smaller than that of the BXTA@liposome, which might be owing to the impaction of BXTA within the liposome. The encapsulation efficiency of BXTA was determined to be 42.8 ± 2.0%. Figure 3E exhibits the CD spectra of BTXA, pure PC/Chol liposome, and the BTXA@liposome. Through comparing the secondary structure of BTXA before and after the PC/Chol liposome loading, it could be concluded that the bioactivity of BTXA was perfectly maintained during the fabrication process of the BTXA@liposome. The slight red shift from 188.9 nm to 191.8 nm might be because of intermolecular interaction between BTXA and the PC/Chol liposome. The particle size of pure PC/Chol liposome and the BTXA@liposome was demonstrated in Fig. 3F. After BTXA encapsulation, the average particle size of the BTXA@liposome, compared to pure PC/Chol liposome, was increased from 132.3 nm to 265.0 nm. In addition, the corresponding zeta potential was also decreased from 8.44 mV (pure PC/Chol liposome) to 5.02 mV (the BTXA@liposome). These results also proved the successful encapsulation of BTXA within the PC/Chol liposome to some extent.

### Morphology, size, and transdermal diffusion capability of the BTXA@Liposome nanoparticles

**Fig. 4:**
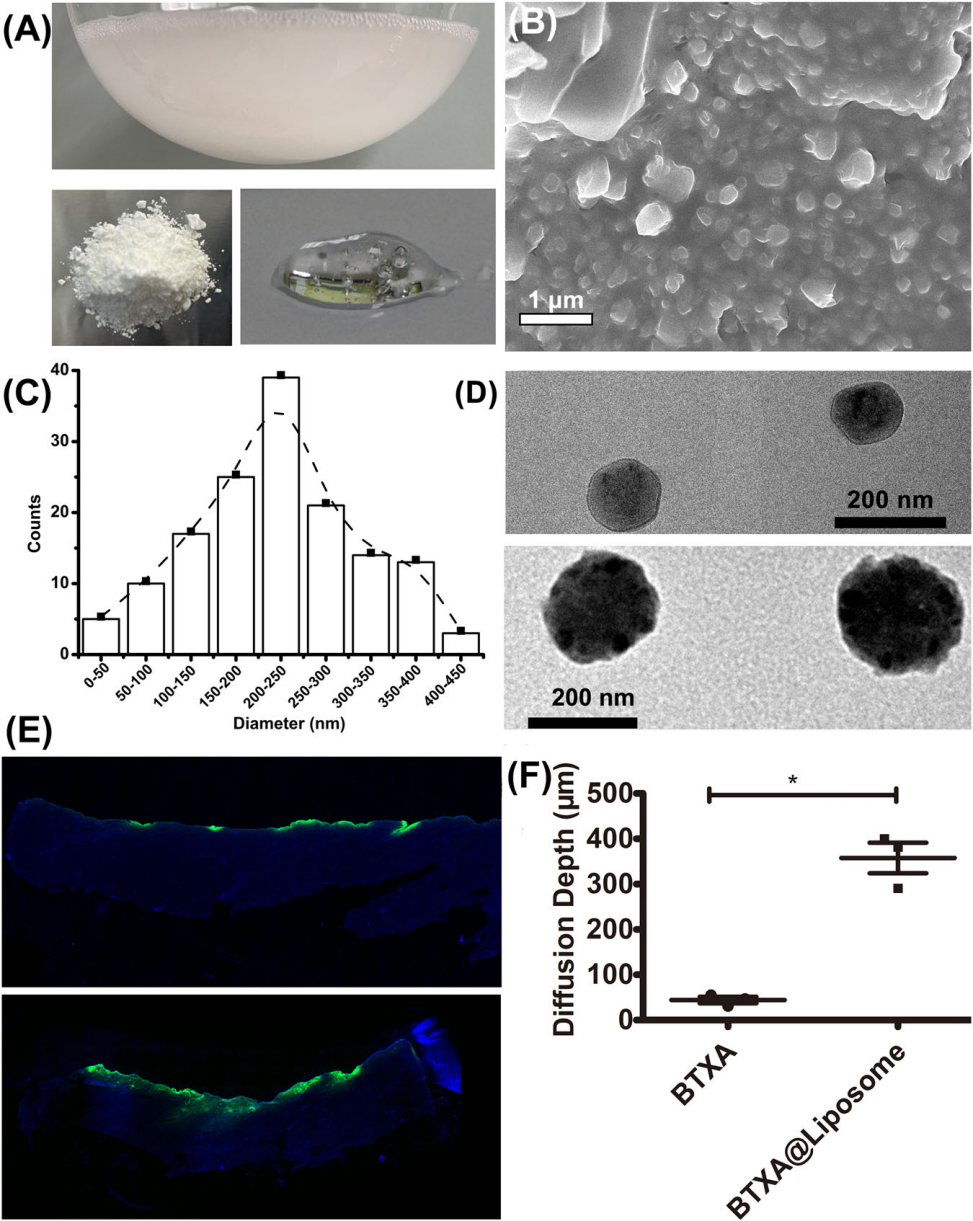
**A** Digital photographs of as-prepared BTXA@liposome nanoparticles and the BTXA@liposome/HA hydrogel; (**B**) SEM and (**D**) TEM images of (upper) the liposome and (lower) the BTXA@liposome nanoparticles; (**C**) particle size distribution of the BTXA@liposome nanoparticles obtained from (**B**). **E** representative fluorescence image of longitudinal section of pigskin smeared with the FITC-BSA/HA hydrogel (upper) and the FITC-BSA@liposome/HA hydrogel for 24 h. **F** quantitative analysis of the average diffusion depth. *p < 0.05

Figure 4A demonstrates the digital photographs of the as-prepared BTXA@liposome nanoparticles and the BXTA@liposome/HA hydrogel. Figure 4B shows the SEM images of the BTXA@liposome nanoparticles. They were in nanoscale and average diameter of the majority was in the range of 200–250 nm (Fig. 4C), in accordance with the results of DLS. Through comparing the TEM images of the liposome and the BTXA@liposome (Fig. 4D), we could obviously find that BTXA has been successfully encapsulated within the PC/Chol liposome, and the particle size of the BTXA@liposome nanoparticles was around 250 nm. In the “introduction” section, we explained why liposome encapsulation facilitate transdermal diffusion of proteins. Here, we used FITC-BSA as a substitute of BTXA because it emits green fluorescence and has nearly identical molecular weight (67 kDa) with BTXA (70 kDa). From Fig. 4E, we could clearly observe that the FITC-BSA@liposome, compared to pure FITC-BSA, could diffuse deeper into skin. Through quantitative analysis (Fig. 4F), we found that the average diffusion depth of the FITC-BSA@liposome was 8 times that of pure FITC-BSA (50 μm) and reached to as high as 380 μm.

### Demographic characteristics of the patients

Table 1 demonstrates that 12 patients in total with facial PSA were included, and all of them cooperated our work until the end of the study. Among them, 8 cases (66.67%) were males and 4 cases (33.33%) were females. Participants were ranged in age from 18 to 35 years, with a mean age of 25.58 ± 4.52. The skin acne type was Pillsbury grade I-IV, and 5 patients in the control group had type I, 4 patients had type II, and 3 patients had grade III. In the experimental group, 4 patients were type I, 2 patients were type II, 5 patients were grade III, and 1 patient was grade IV. Here, cutaneous acne scars were divided into five categories: blotchy, mild, moderate, and severe. All patients were followed up 1 week after the end of the last treatment.

**Table 1:** Characteristics of general information of patients

		Fractional CO_2_ laser + HA	Fractional CO_2_ laser + BXTA@liposome/HA
Left side of the face		9	3
Right side of the face		3	9
gender	Male	8	8
	Female	4	4
age	18–30	10	10
	>30	2	2
Preoperative acne grading	Mild grade I	5	4
	Moderate grade II	4	2
	Moderate grade	3	5
	Severe grade IV	0	1
Preoperative acne scar grading	Spotty/ macular	2	2
	Mild	6	6
	Moderate	2	2
	Severe	2	2

### Clinical efficacy evaluation

**Fig. 5:**
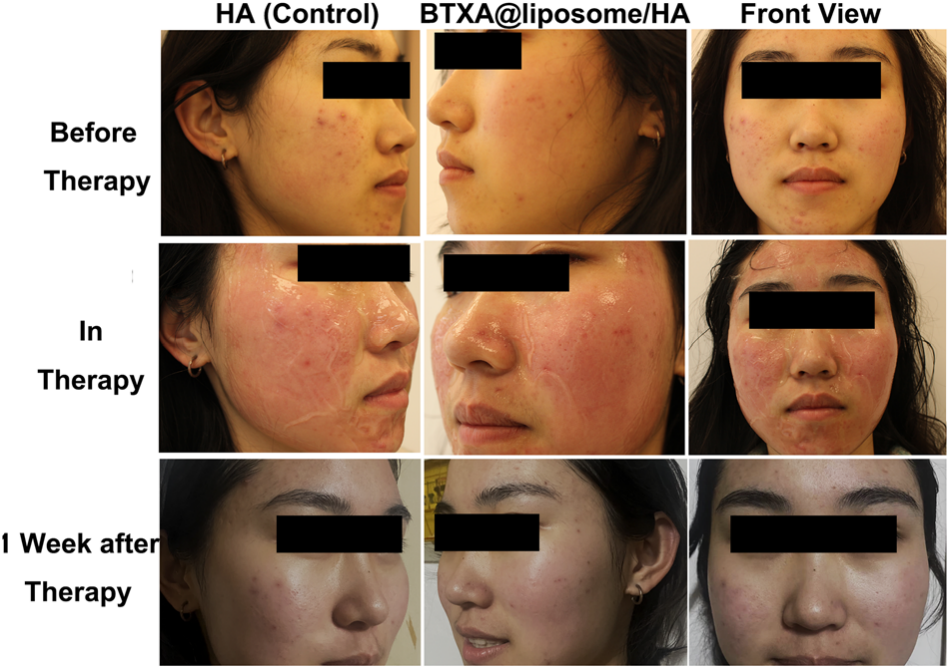
Representative demonstration of the clinical efficacy

Figure 5 and Figs. S1–S11 record the clinical efficacy of the 12 PSA patients after the BTXA@liposome/HA hydrogel treatment. HA hydrogel treatment was used as control. Comparison of ECCA scores between the two groups was as follows: before treatment, there was no significant differences in ECCA scores between the two groups (*p* > 0.05). After treatment, the ECCA scores of the two groups were lower than those before treatment, and the ECCA scores of the experimental group were lower than those of the control group (*p* < 0.05) (Table 2). The results of self-evaluation showed that at the follow-up of 1 week after the treatment, 2 cases of the experimental group had significant improvement, 4 cases had noticeable improvement, 5 cases had moderate improvement, and 1 case had mild improvement. There was no significant differences in the scores of the quartile scale between the two sides (*p* > 0.05) (Table 3). Comparison of efficacy between the two groups was as follows: after treatment, the total effective rate of the experimental group was 91.67%, and the total effective rate of the control group was 66.67%. There was no significant differences in the total effective rate between the two groups (*p* > 0.05) (Table 4). Safety comparison between the two groups was as follows: during the treatment period, the incidence of total adverse reactions in the experimental group was 47.22%, which was lower than that in the control group (65.50%), and the differences between the two groups were statistically significant (*p* < 0.05). After the operation, the symptoms gradually disappeared after about a week, as shown in Table 5.

**Table 2:** Comparison of ECCA scores on both sides of face after treatments (Case (%), X ± S)

	Before treatment	After treatment	*T* value	*p* value
Control group	45.42 ± 13.05	38.33 ± 12.12	2.754	0.019
Experimental group	52.92 ± 14.84	39.58 ± 8.11	4.485	0.001
*T* value	1.320	0.297		
*P* value	0.200	0.769

**Table 3:** Comparison of patient self-evaluation Quartile Scale on Both Sides (Case (%), $\bar{{\rm{X}}}$ ± S))

Group	Case	Mild improvement 0–25% (1 point)	Moderate improvement 25–50% (2 points)	Noticeable improvement 51–75% (3 points)	Significant improvement 70–100% (4 points)	*Z* value	*p* value
Control group	12	3	8	1	0	6.500	0.261
Experimental group	12	1	5	4	2

**Table 4:** Comparison of the effective rates of clinical treatment between the two groups (Case (%))

Group	Case	Slightly effective	effective	void	Always effective
Control group	12	2	6	4	8 (66.67%)
Experimental group	12	6	5	1	11 (91.67%)
X2					2.274
*P*					0.317

**Table 5:** Comparison of the incidence of adverse reactions between the two groups during treatment (Case (%))

Group	Case	dropsy	erythema	pain	Itching	effusion	Worsening of skin lesions	Incidence of total adverse reactions
Control group	12	6/12	10/12	10/12	8/12	4/12	4/12	42/72 (58.3)
Experimental group	12	2/12	5/12	4/12	4/12	2/12	1/12	18/72 (25.0)
X2								16.457
P								0.0092

In sum, the BTXA@liposome/HA hydrogel treatment could, to some extent, relieve PSA but didn’t show significant advantage over the HA hydrogel treatment. This situation, we though, could be attributed to two reasons: (1) the vicious HA molecules inhibited the infiltration of the BTXA@liposome nanoparticles into skin; (2) the limited sample capacity and the relatively short curative time affected the final conclusion.

## Conclusions

In this work, the BTXA@PC/Chol liposome nanoparticles were synthesized and applied for PSA treatment. The results of FTIR spectra, XRD spectra, N_2_ absorption/desorption tests, CD spectra, DLS tests, and Zeta potential tests proved the BTXA had been encapsulated within the cavity of PC/Chol liposome with encapsulation efficiency of 42.8 ± 2.0%. In addition, CD spectra also proved that BTXA maintained its bioactivity during the preparation process of the BXTA@liposome nanoparticles. SEM and TEM images demonstrated that the BTXA@liposome nanoparticles were in nanoscale and average diameter of the majority was in the range of 200–250 nm. Simulated pigskin diffusion assay indicated the liposome encapsulation facilitated infiltration of BTXA within the skin. The average diffusion depth of the BTXA@liposome nanoparticles was reached 380 μm. Clinical efficacy evaluation indicated that BTXA@liposome/HA hydrogel treatment could, to some extent, relieved PSA but didn’t show significant advantage over HA treatment. This might be because of that (1) the vicious HA molecules inhibited the infiltration of the BTXA@liposome nanoparticles into skin and (2) the limited sample capacity and curative time affected the final conclusion. Thus, further work is needed to verify the feasibility and curative effect of this method in PSA treatment in the future.

## Supplementary information

Supplementary Information
