# Supplementary material for: Transdermal delivery of botulinum toxin-A through phosphatidylcholine/cholesterol nanoliposomes for treatment of post-acne scarring
Source: J Mater Sci Mater Med. 2024 Jul 29;35(1):41. doi: 10.1007/s10856-024-06810-1 (PMC11286643; doi:10.1007/s10856-024-06810-1)
Supplement: Supplementary file 1 — Supplementary Information [file 10856_2024_6810_MOESM1_ESM.docx]

Consolidating Rotten/Pulverized Silk Fabrics through Spatially Adaptive 3D Strong/Tough Framework Mediated Structural Shaping of Fiber Bundles

Xiongbin Zhu^a^, Hao Wu^b^, Yanting Yu^a^, Beisong Fang^b^, Shaohui Chen^b^, Huan Xie^b^, Qiong Zhang^b^, Changying Yang^a, *^, Yang Zhao^b, *^, Jiabing Ran^a,^ *

^a^ College of Biological & Pharmaceutical Sciences, China Three Gorges University, Yichang 443002, China

^b^ Jingzhou Preservation Centre of Cultural Relics, Jingzhou 434020, China

*Corresponding Authors: Changying Yang ([changying.yang@ctgu.edu.cn](mailto:changying.yang@ctgu.edu.cn)); Yang Zhao ([729130036@qq.com](mailto:729130036@qq.com)); Jiabing Ran ([jiabingran@outlook.com](mailto:jiabingran@outlook.com); [ranjiabing@ctgu.edu.cn](mailto:ranjiabing@ctgu.edu.cn))


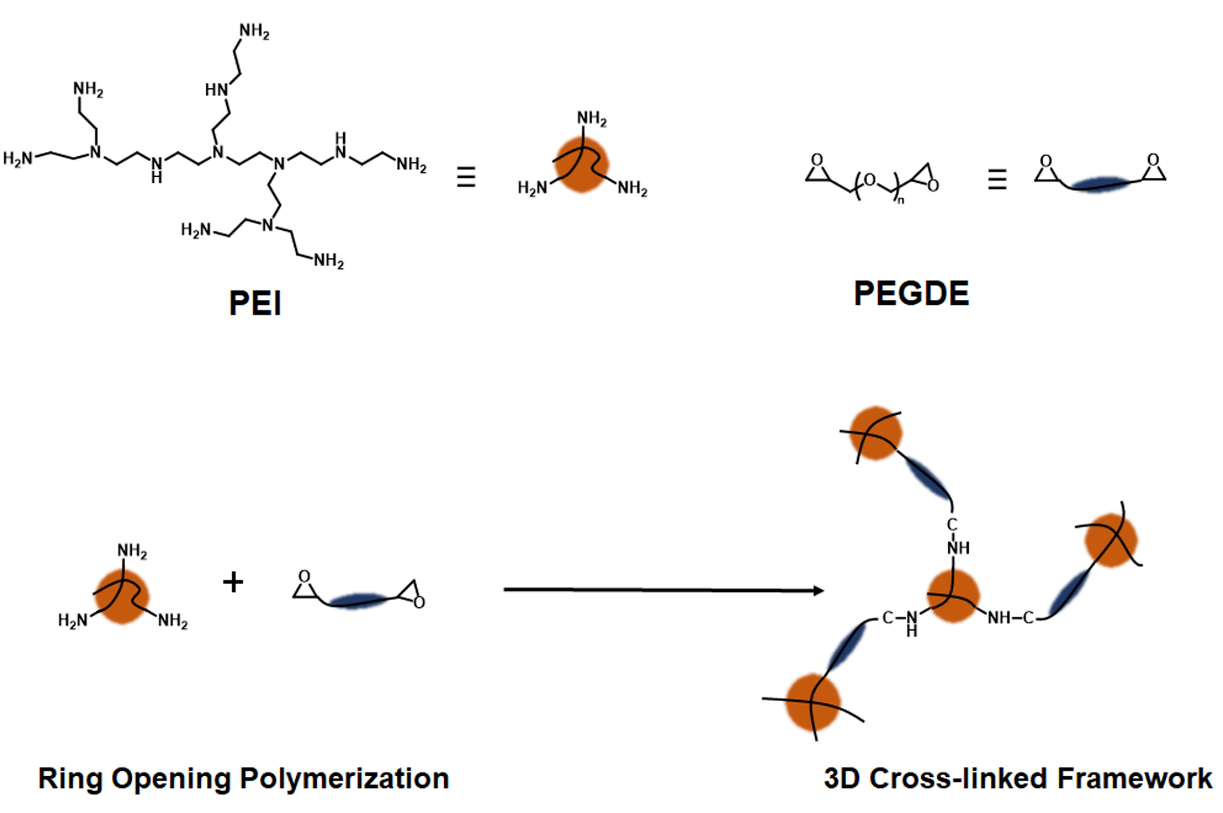
Fig. S1 Schematic diagram of the ring opening polymerization between PEI molecules and PPEGDE molecules


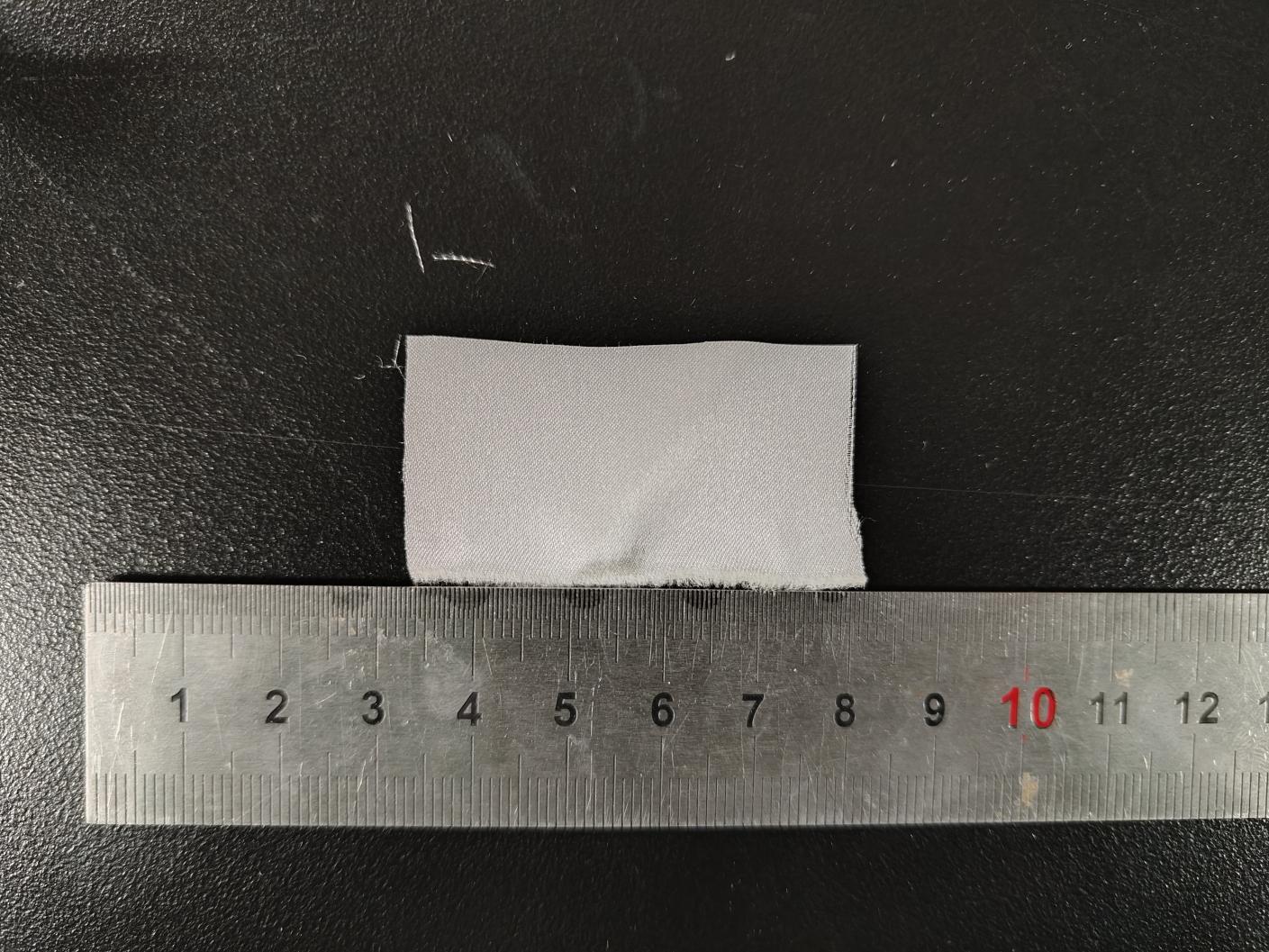


Fig.S2 Digital photograph of original silk fabric


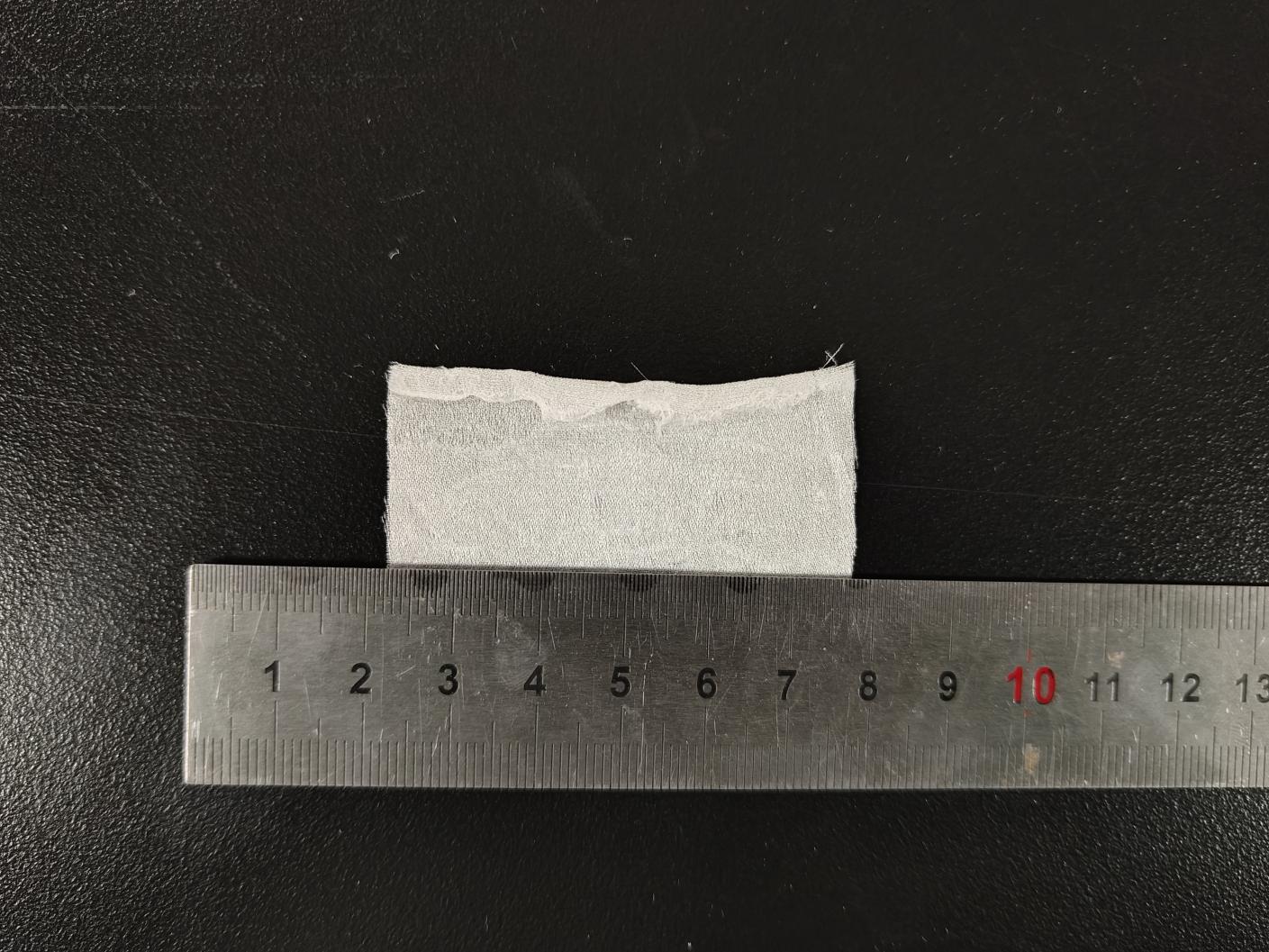


Fig.S3 Digital photograph of simulated rotten/pulverized silk fabric


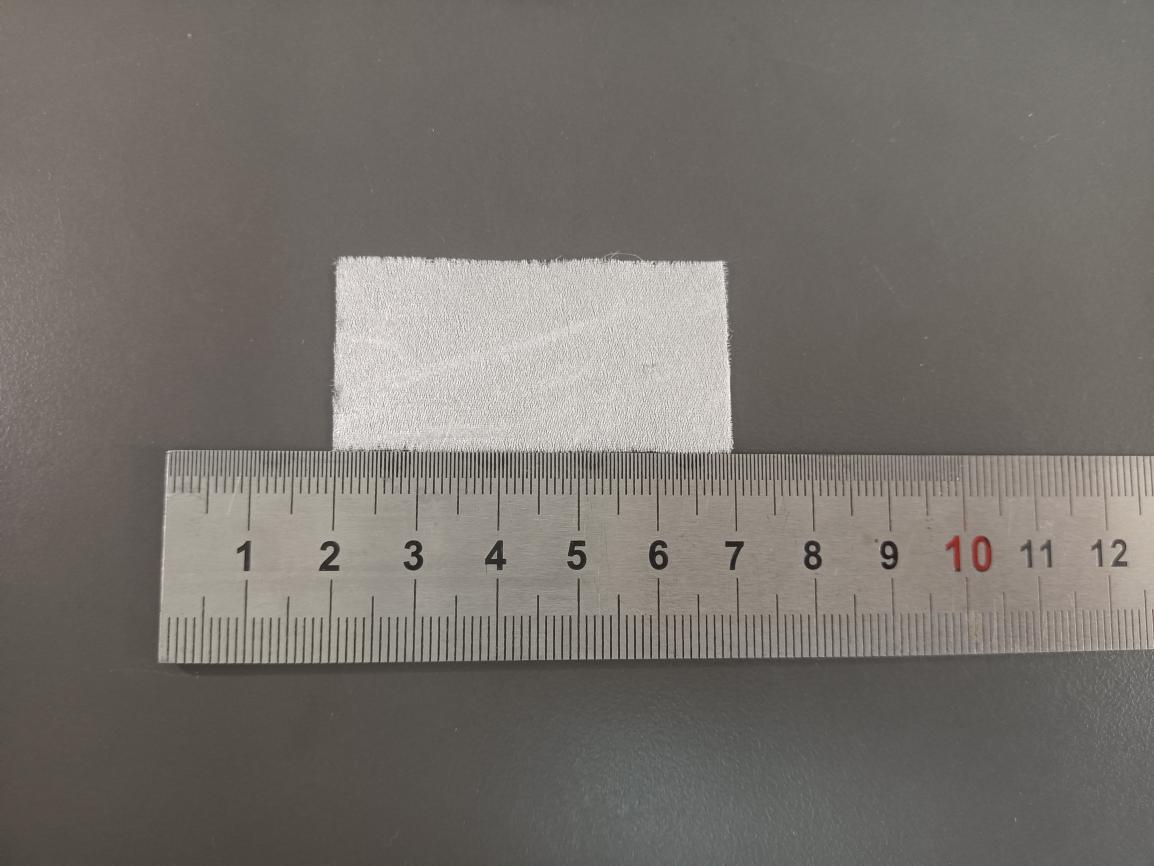


Fig.S4 Digital photograph of simulated rotten/pulverized silk fabric treated with 0% PEI + 0% PPEGDE + 1% IPP


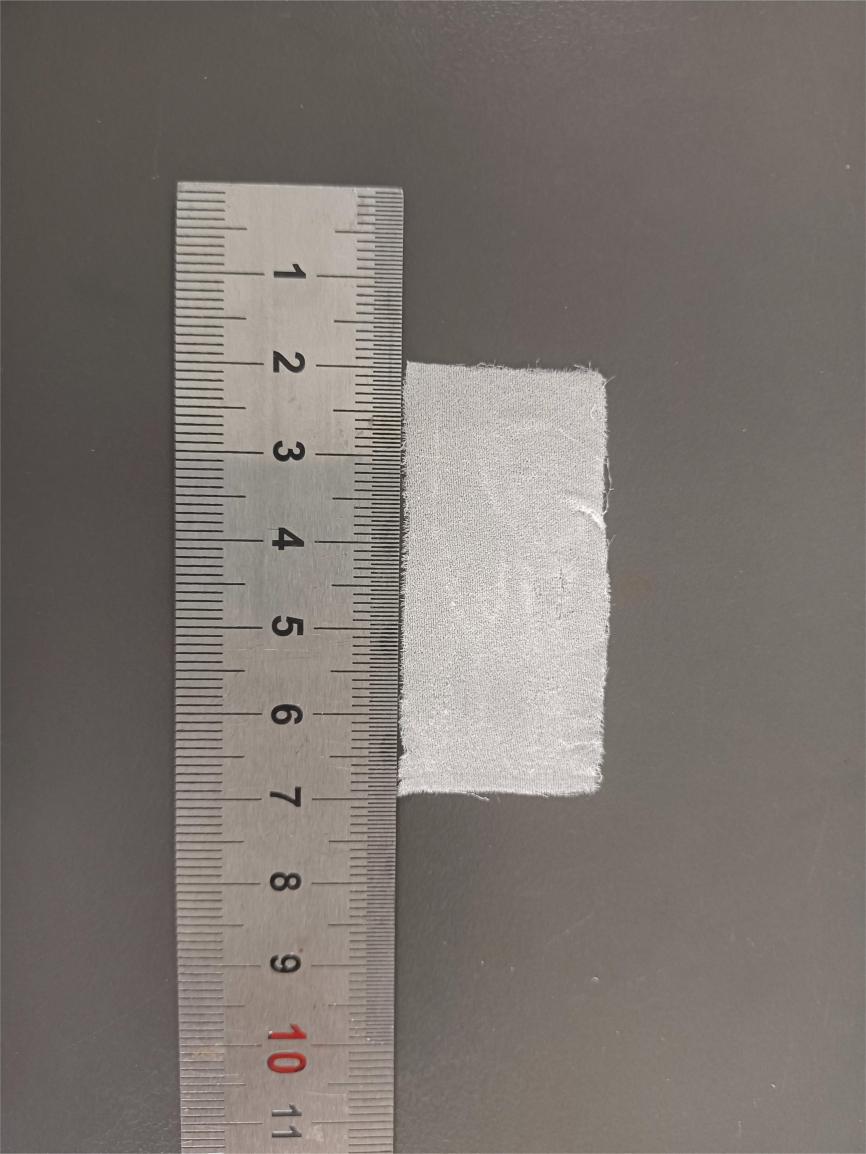
Fig.S5 Digital photograph of simulated rotten/pulverized silk fabric treated with 0% PEI + 0.5% PPEGDE + 1% IPP


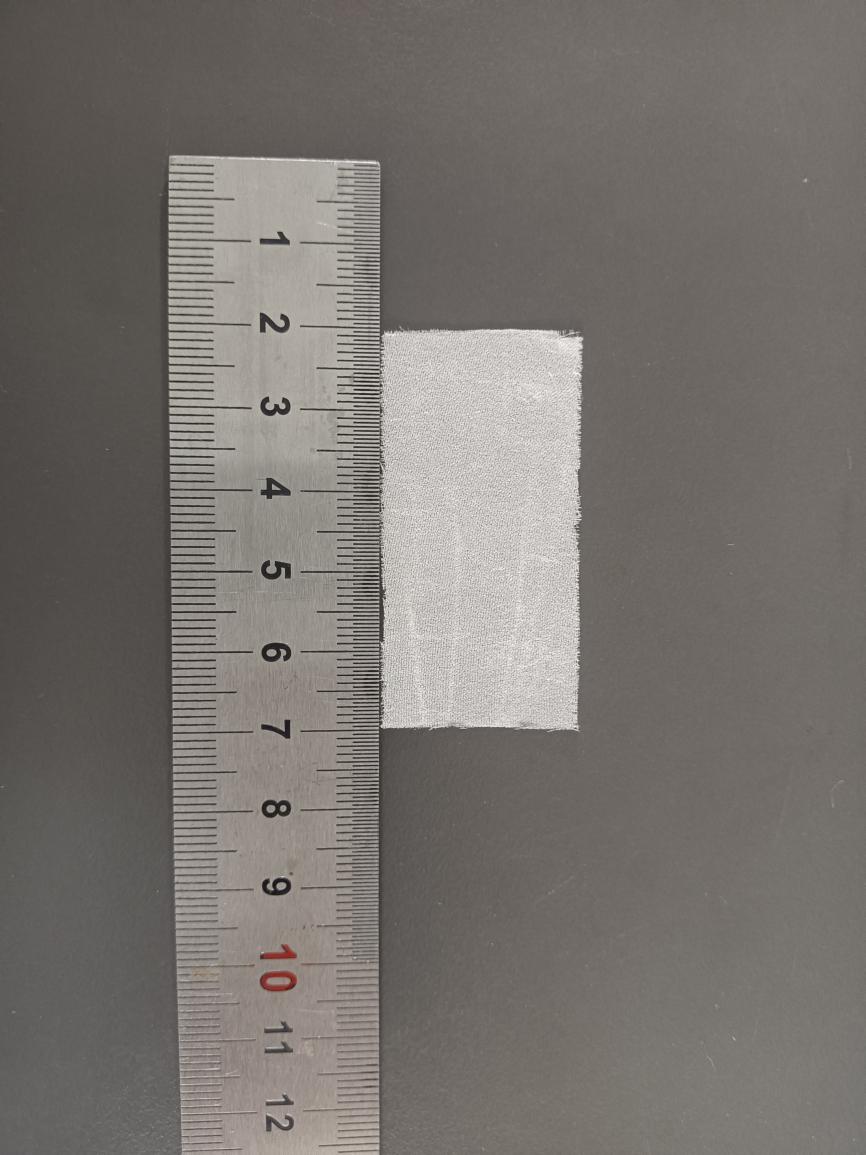
Fig.S6 Digital photograph of simulated rotten/pulverized silk fabric treated with 0% PEI + 1% PPEGDE + 1% IPP


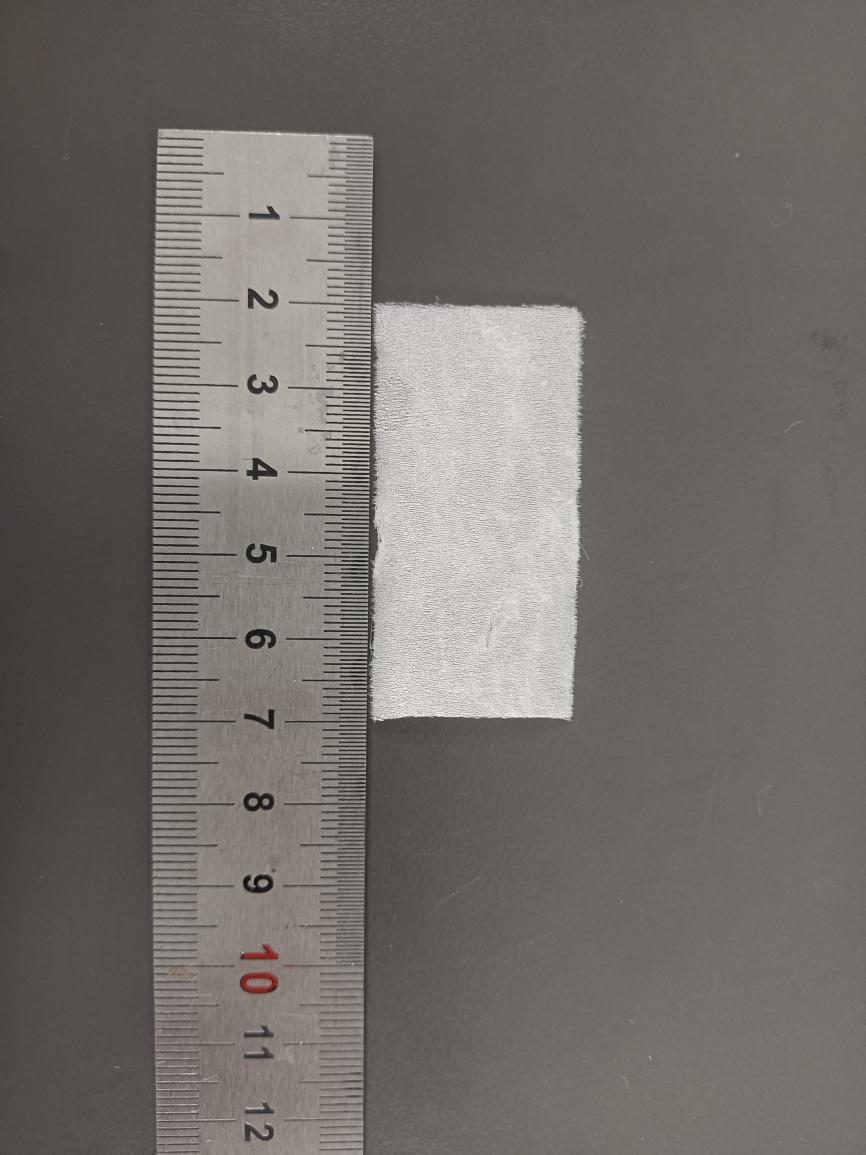
Fig.S7 Digital photograph of simulated rotten/pulverized silk fabric treated with 0% PEI + 1.5% PPEGDE + 1% IPP


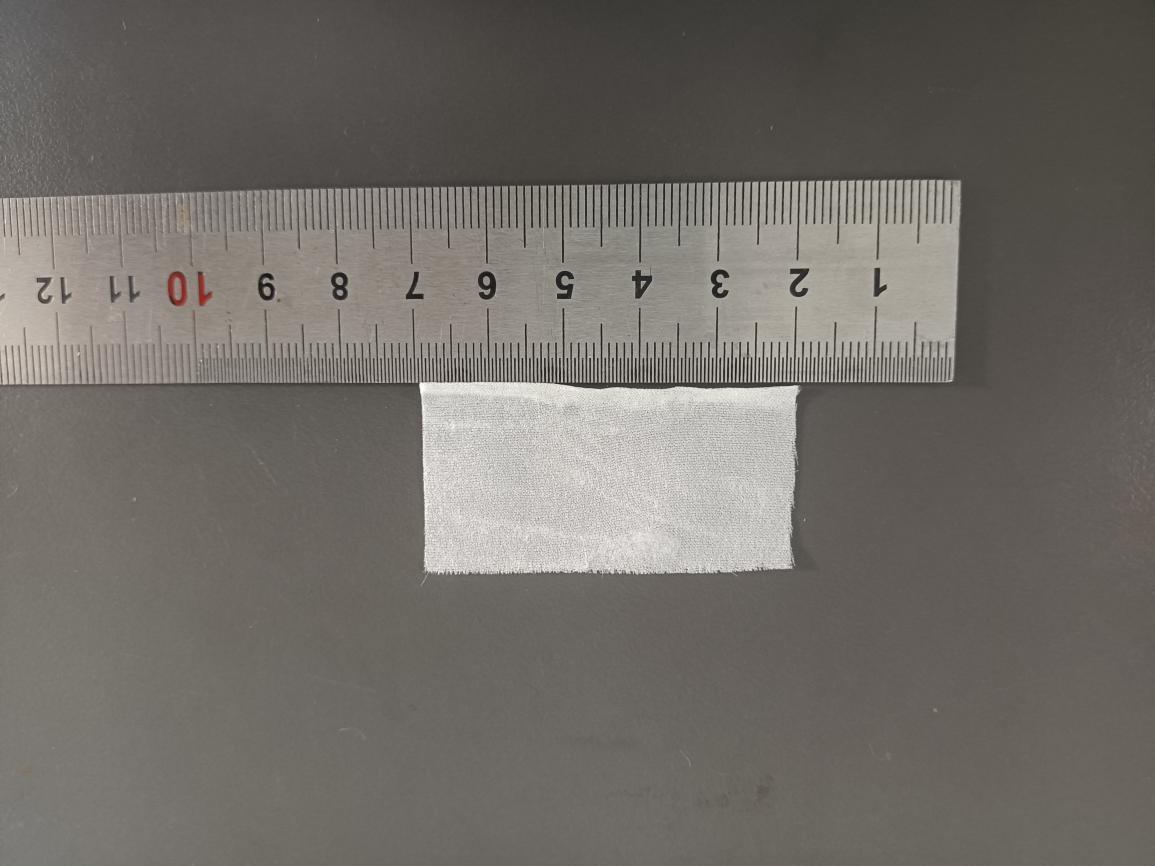


Fig.S8 Digital photograph of simulated rotten/pulverized silk fabric treated with 0% PEI + 2% PPEGDE + 1% IPP


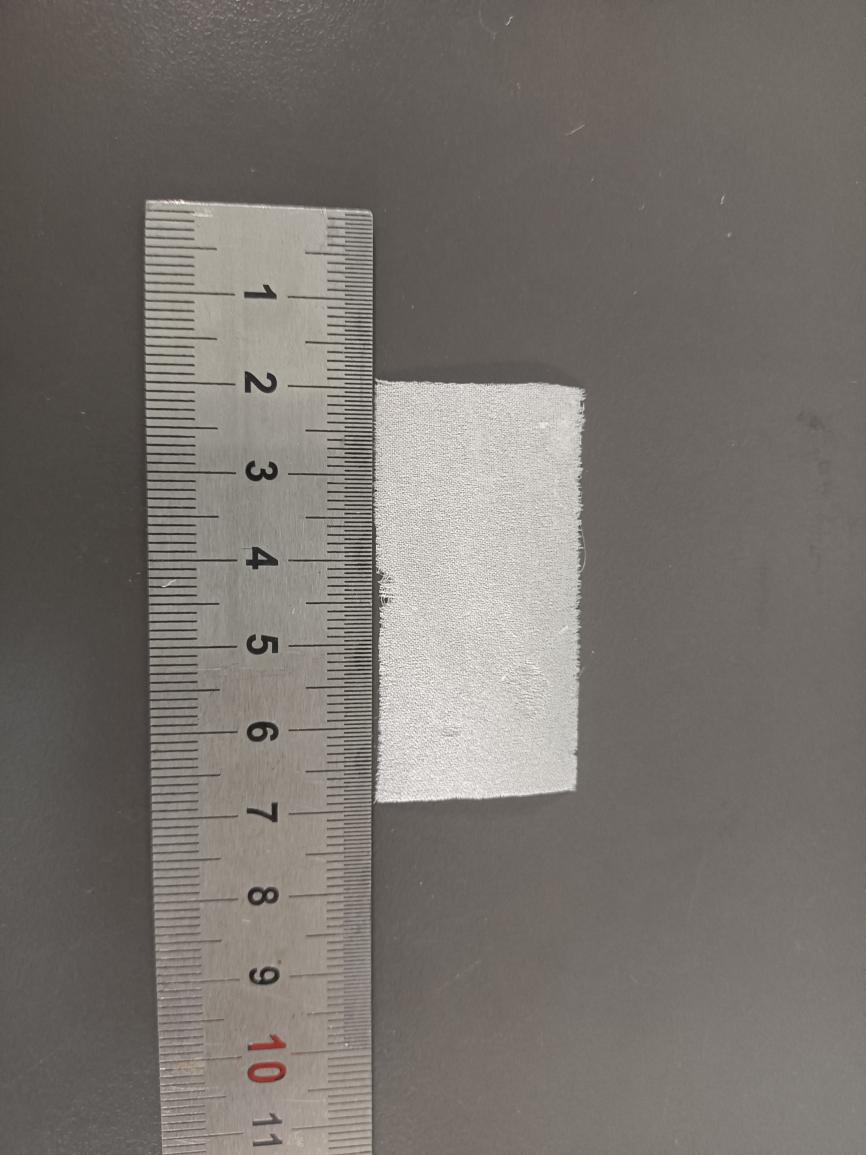


Fig.S9 Digital photograph of simulated rotten/pulverized silk fabric treated with 0% PEI + 2.5% PPEGDE + 1% IPP


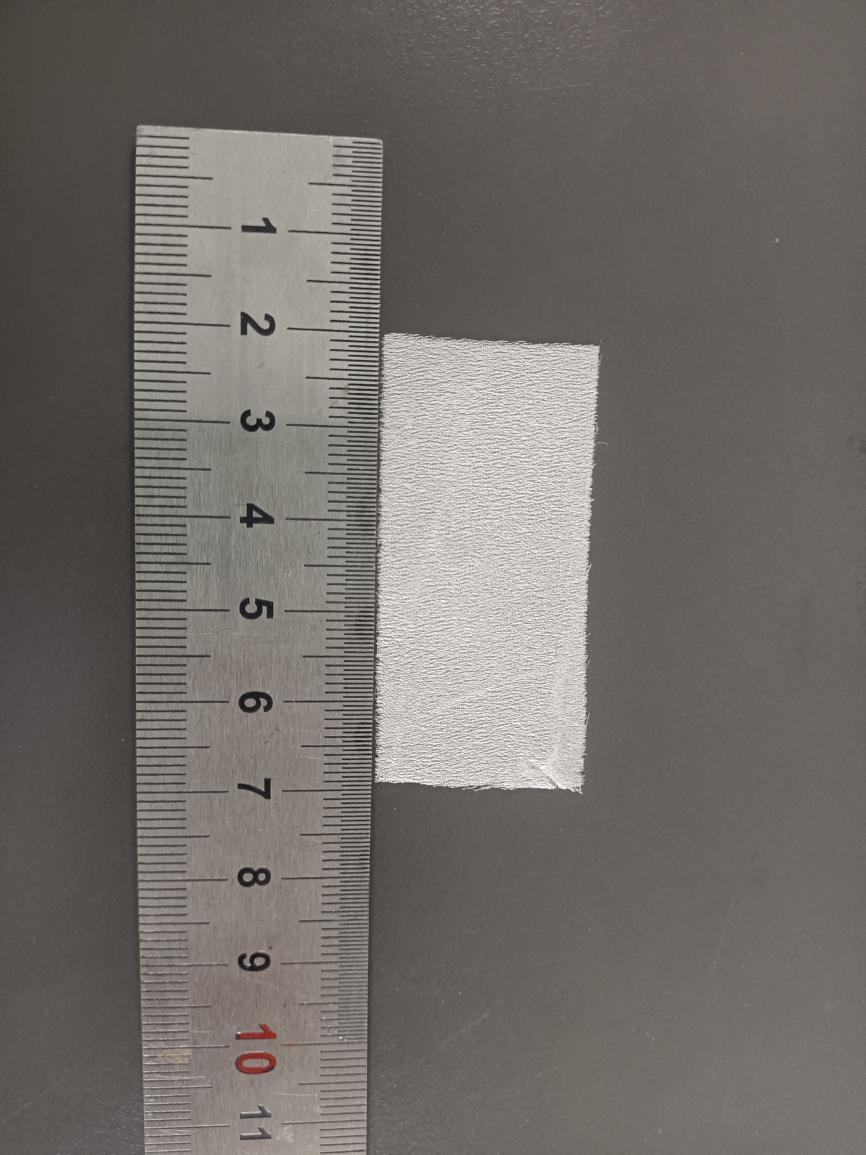


Fig.S10 Digital photograph of simulated rotten/pulverized silk fabric treated with 0% PEI + 3% PPEGDE + 1% IPP


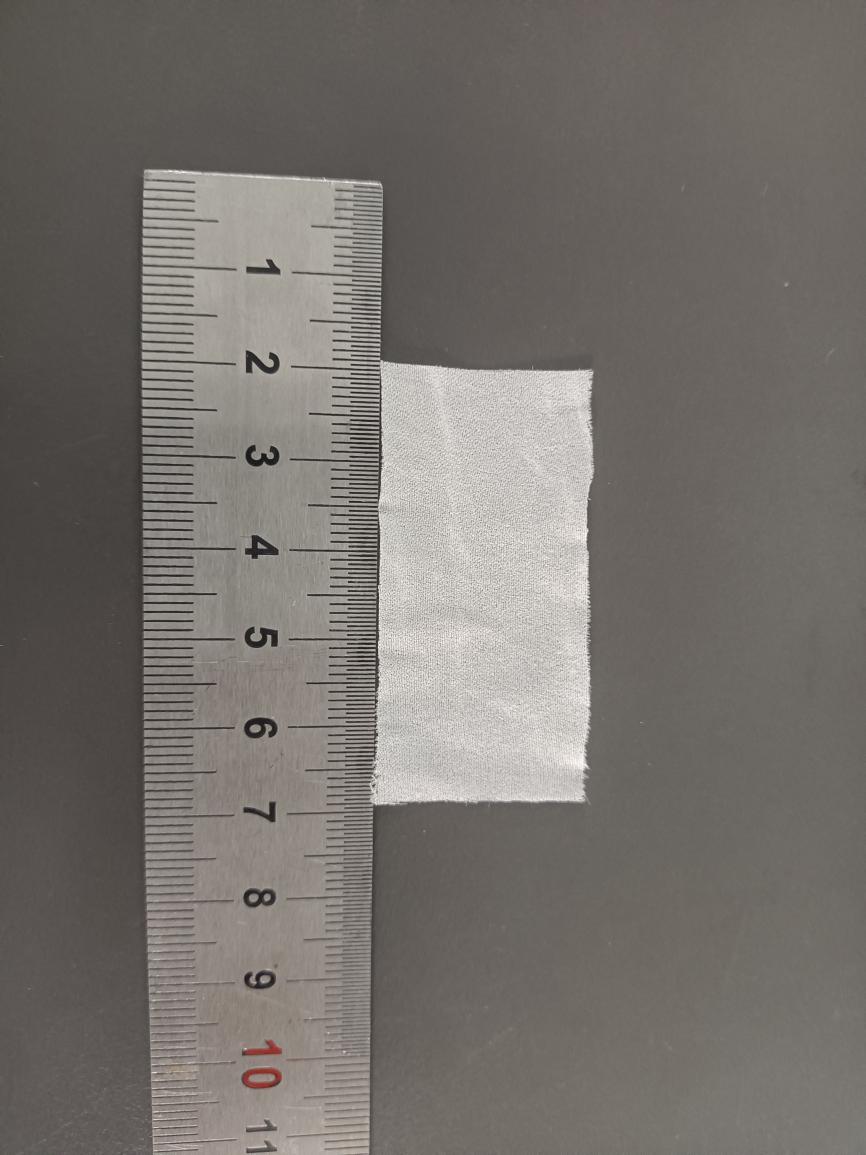


Fig.S11 Digital photograph of simulated rotten/pulverized silk fabric treated with 0% PEI + 3.5% PPEGDE + 1% IPP


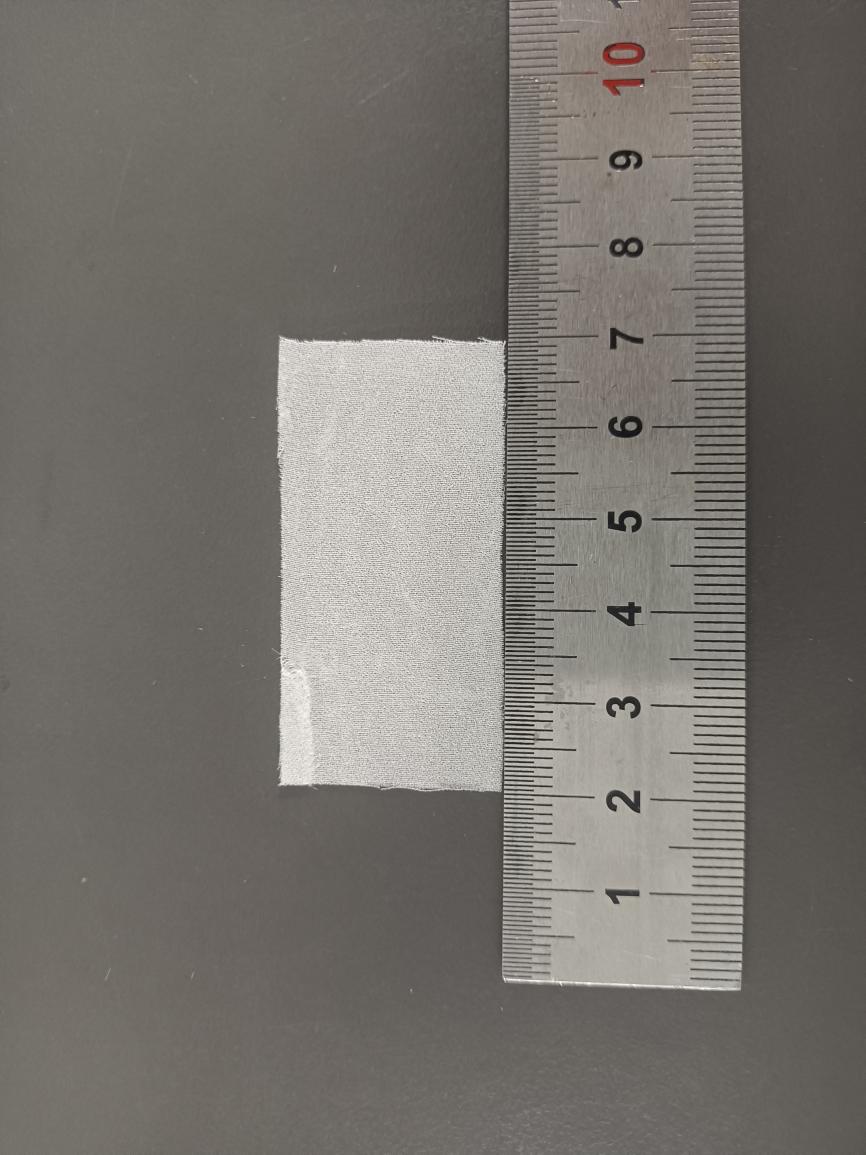


Fig.S12 Digital photograph of simulated rotten/pulverized silk fabric treated with 0% PEI + 4% PPEGDE + 1%IPP


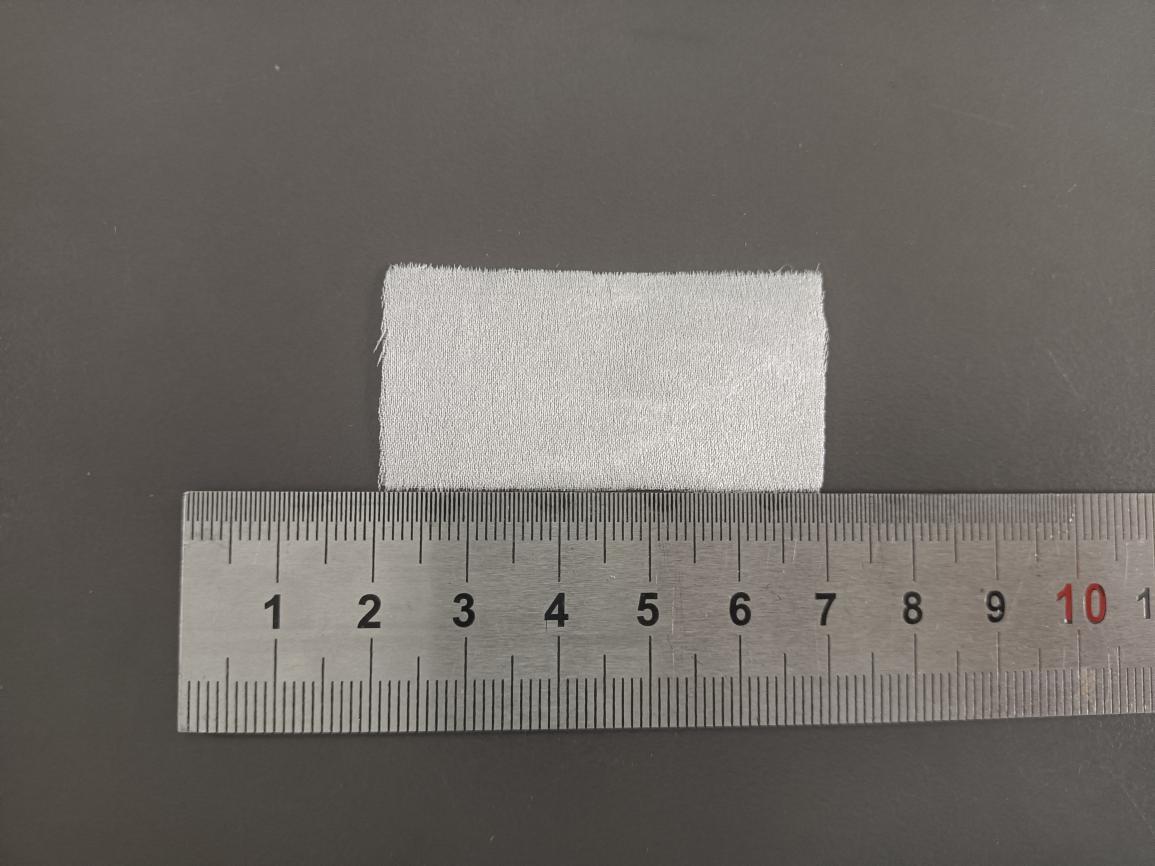


Fig.S13 Digital photograph of simulated rotten/pulverized silk fabric treated with 0% PEI + 4.5% PPEGDE + 1% IPP


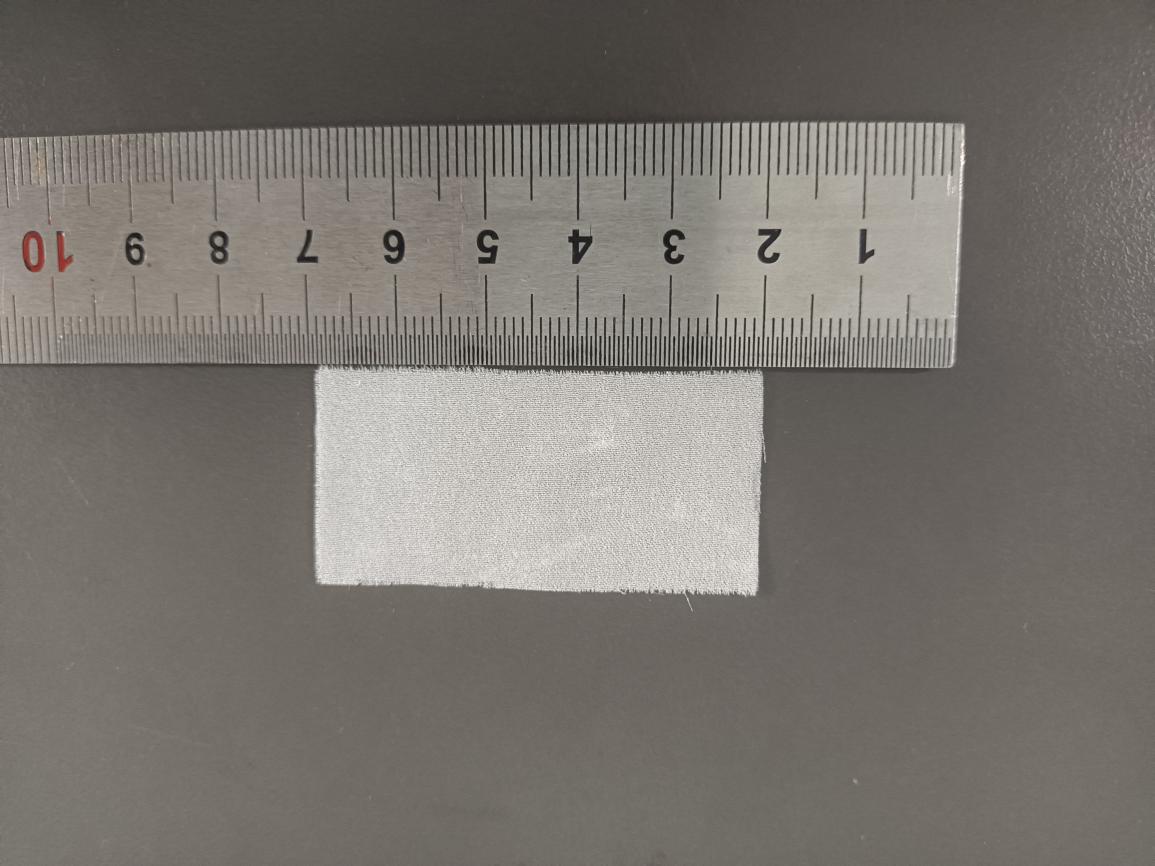


Fig.S14 Digital photograph of simulated rotten/pulverized silk fabric treated with 0% PEI + 5% PPEGDE + 1% IPP


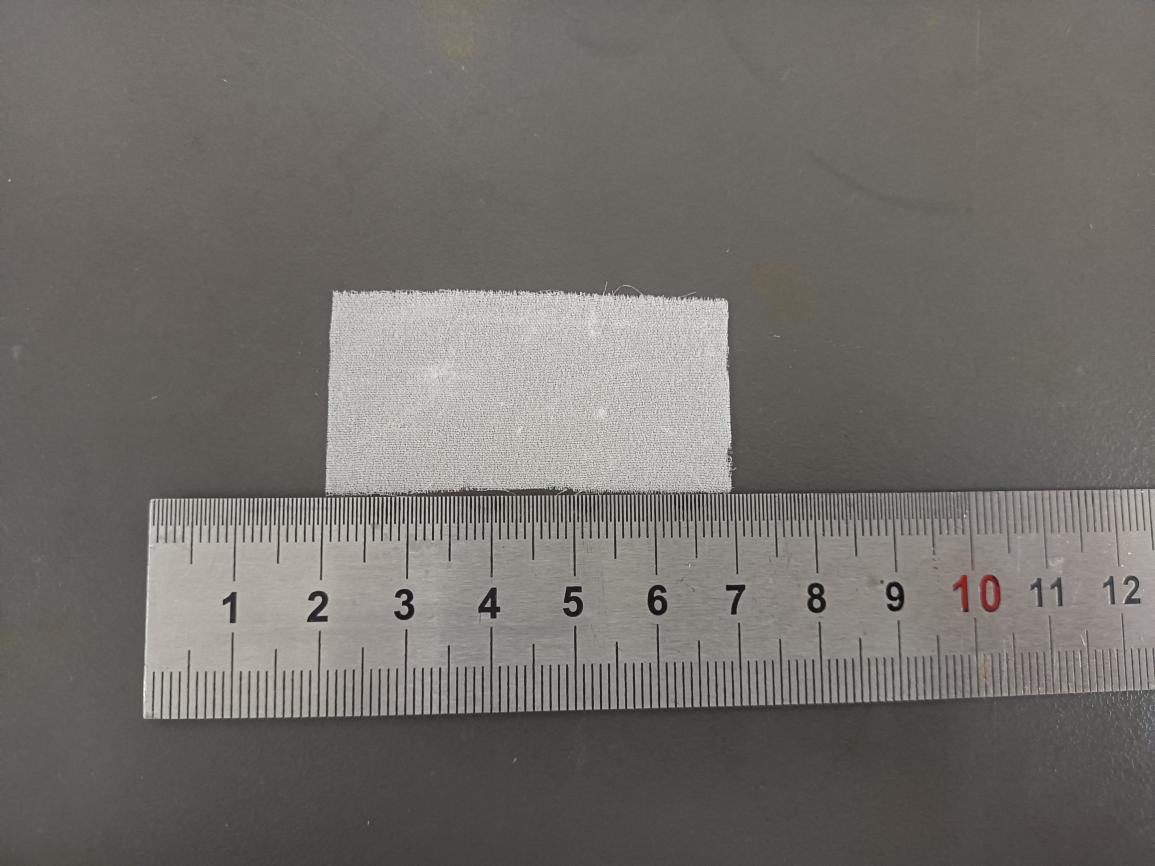


Fig.S15 Digital photograph of simulated rotten/pulverized silk fabric treated with 2% PEI + 0% PPEGDE + 1% IPP


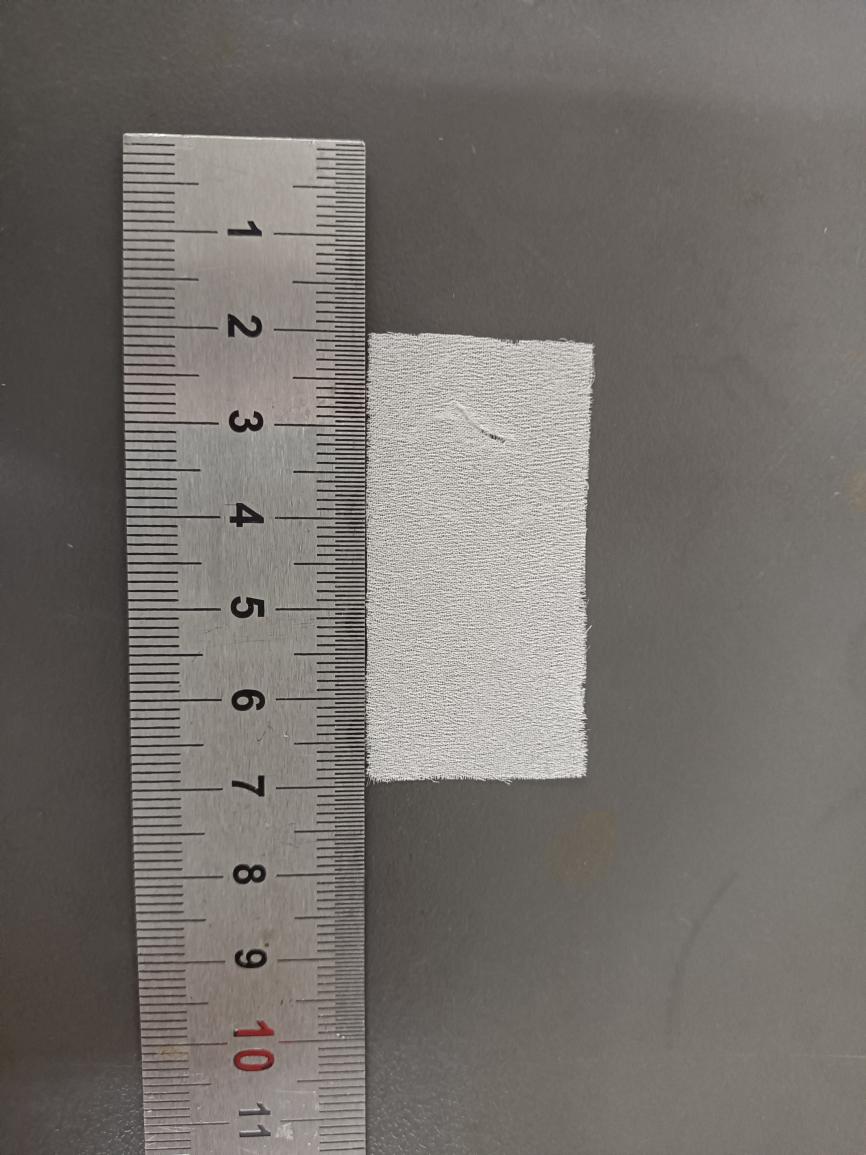
Fig.S16 Digital photograph of simulated rotten/pulverized silk fabric treated with 2% PEI + 0.5% PPEGDE + 1% IPP


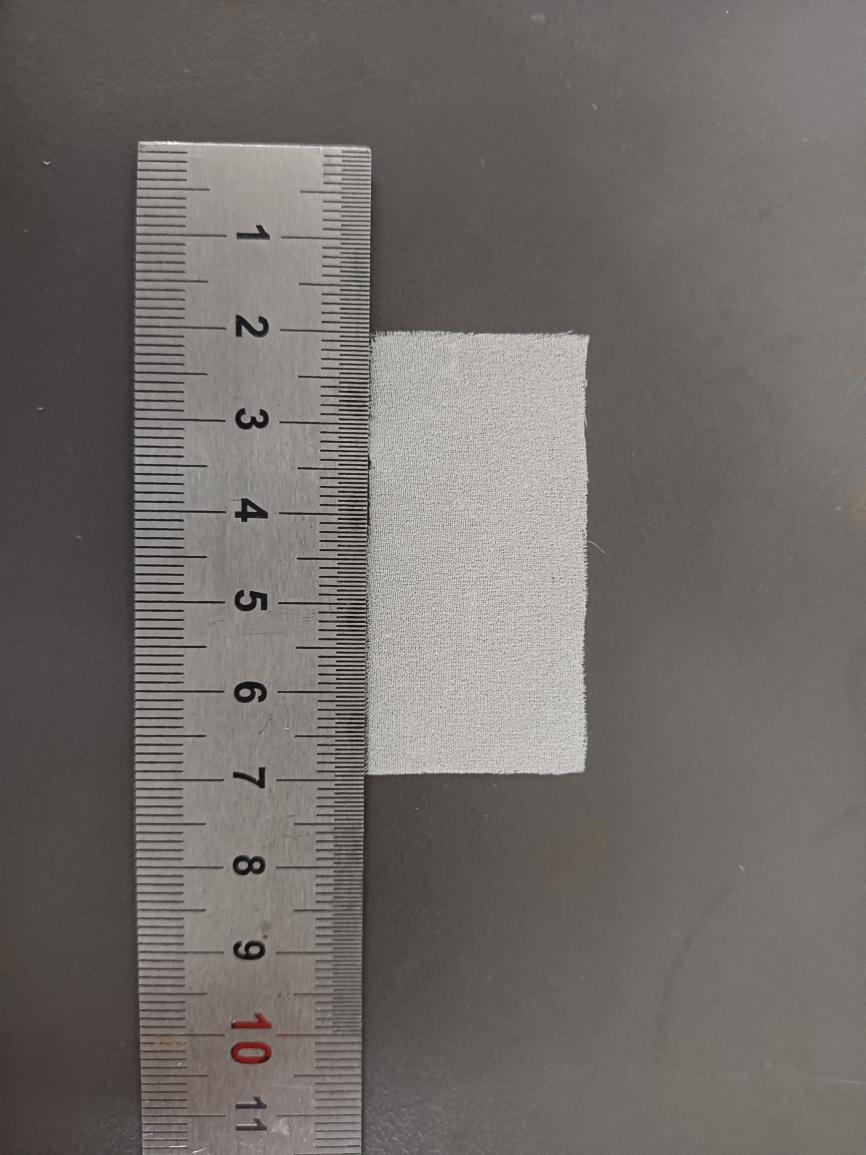
Fig.S17 Digital photograph of simulated rotten/pulverized silk fabric treated with 2% PEI+1% PPEGDE + 1%IPP


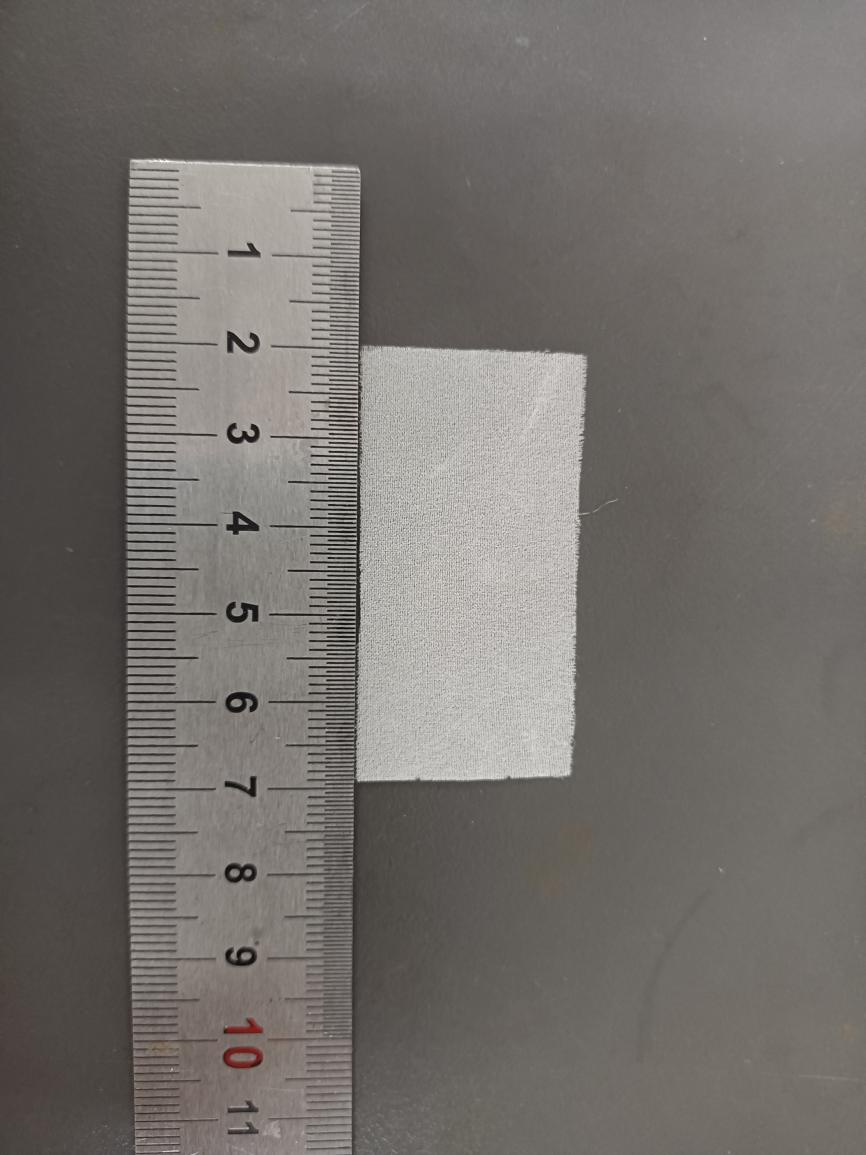
Fig.S18 Digital photograph of simulated rotten/pulverized silk fabric treated with 2% PEI + 1.5% PPEGDE + 1% IPP


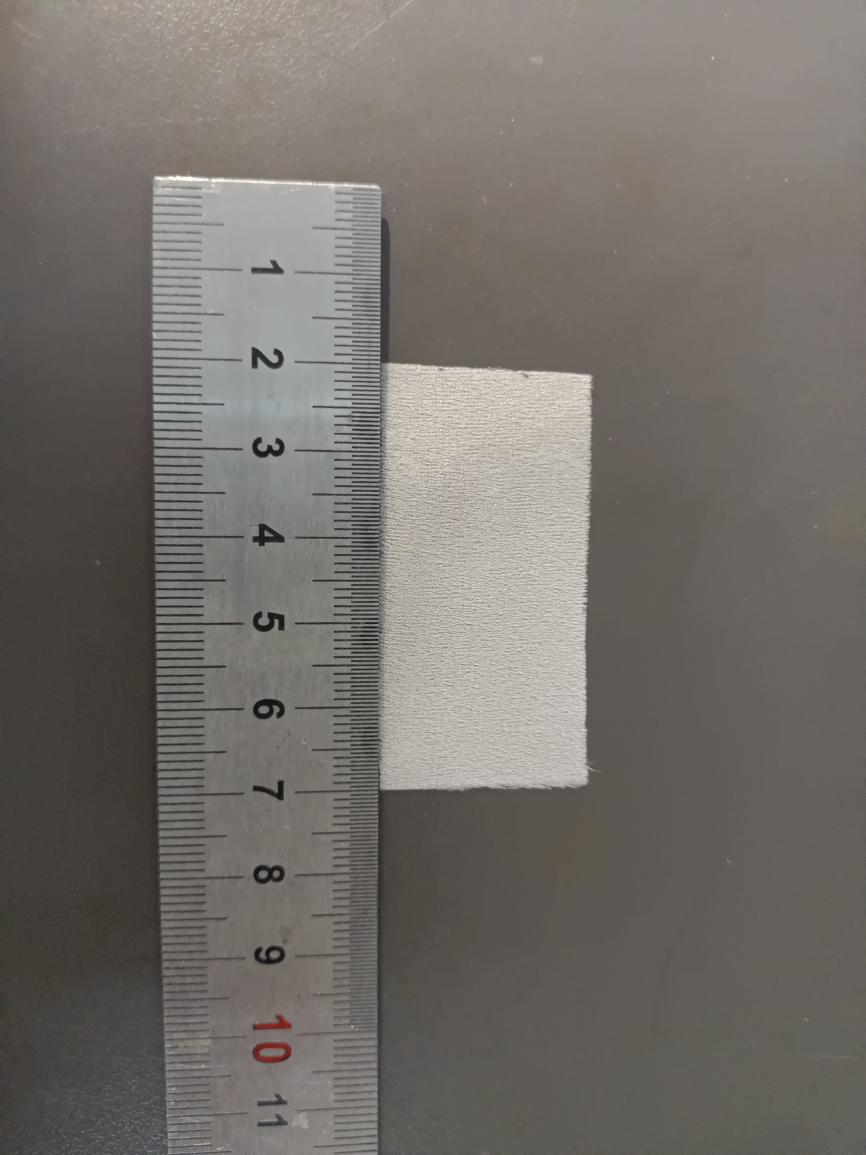
Fig.S19 Digital photograph of simulated rotten/pulverized silk fabric treated with 2% PEI + 2% PPEGDE + 1% IPP


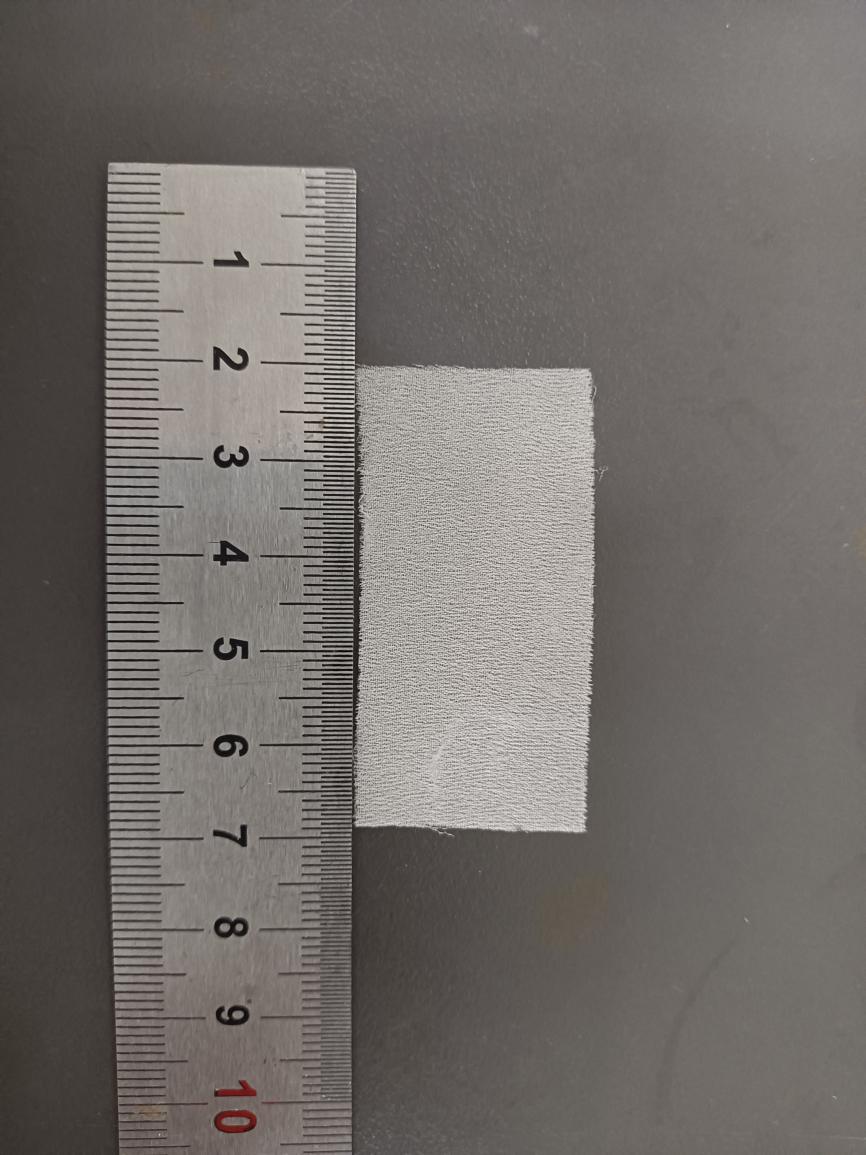
Fig.S20 Digital photograph of simulated rotten/pulverized silk fabric treated with 2% PEI + 2.5% PPEGDE + 1% IPP


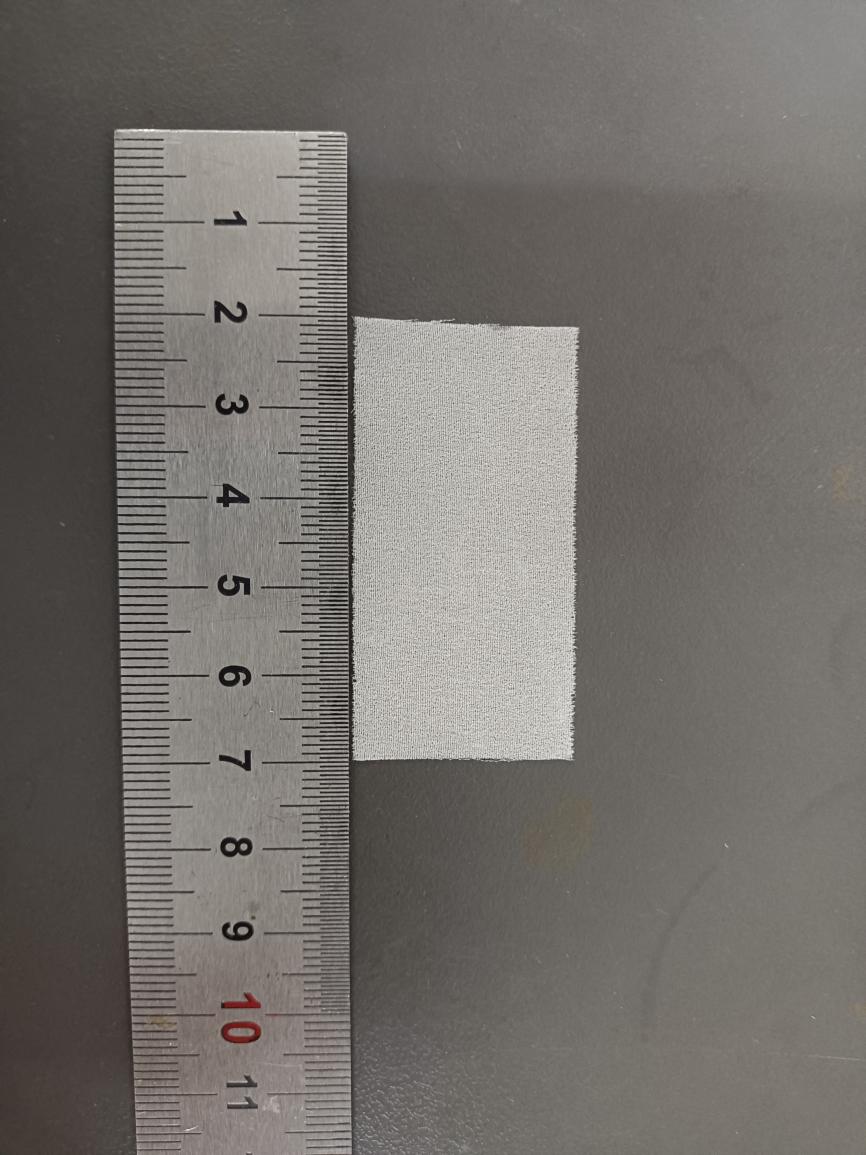
Fig.S21 Digital photograph of simulated rotten/pulverized silk fabric treated with 2% PEI + 3% PPEGDE + 1%IPP


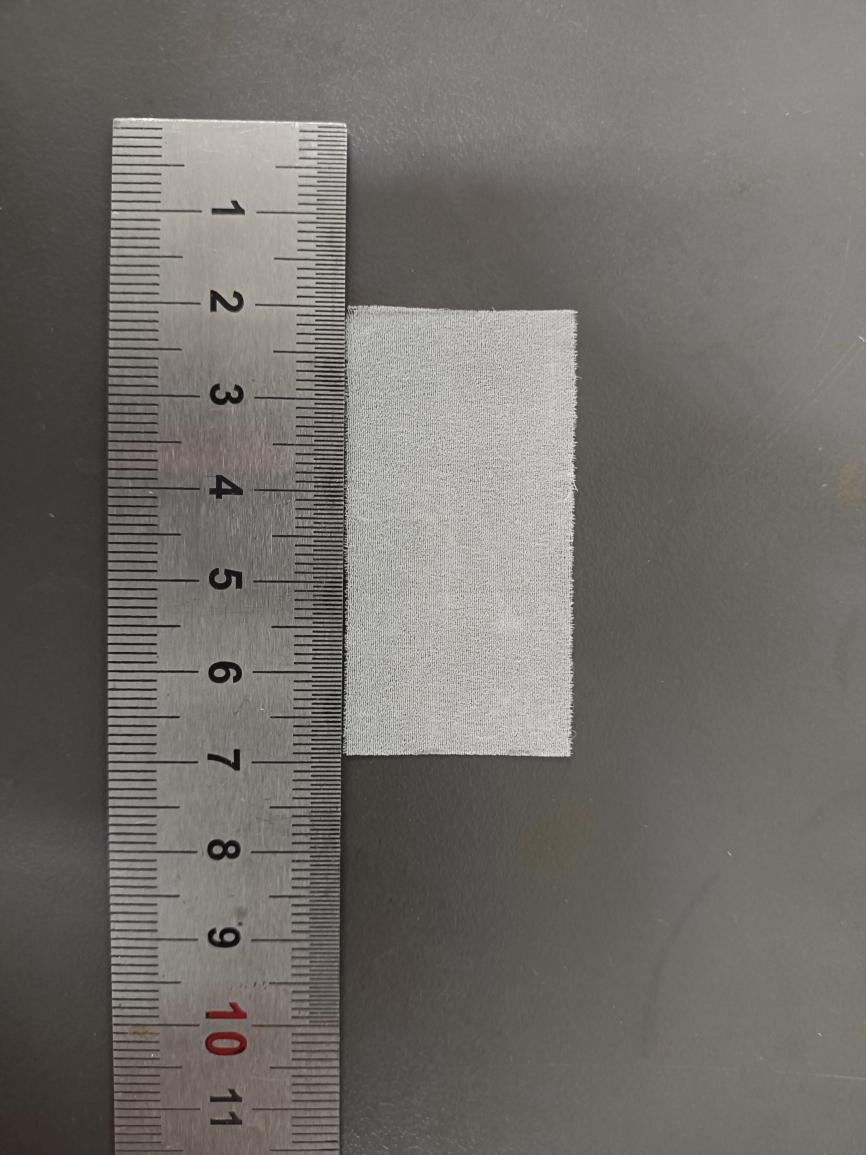
Fig.S22 Digital photograph of simulated rotten/pulverized silk fabric treated with 2% PEI + 3.5% PPEGDE + 1% IPP


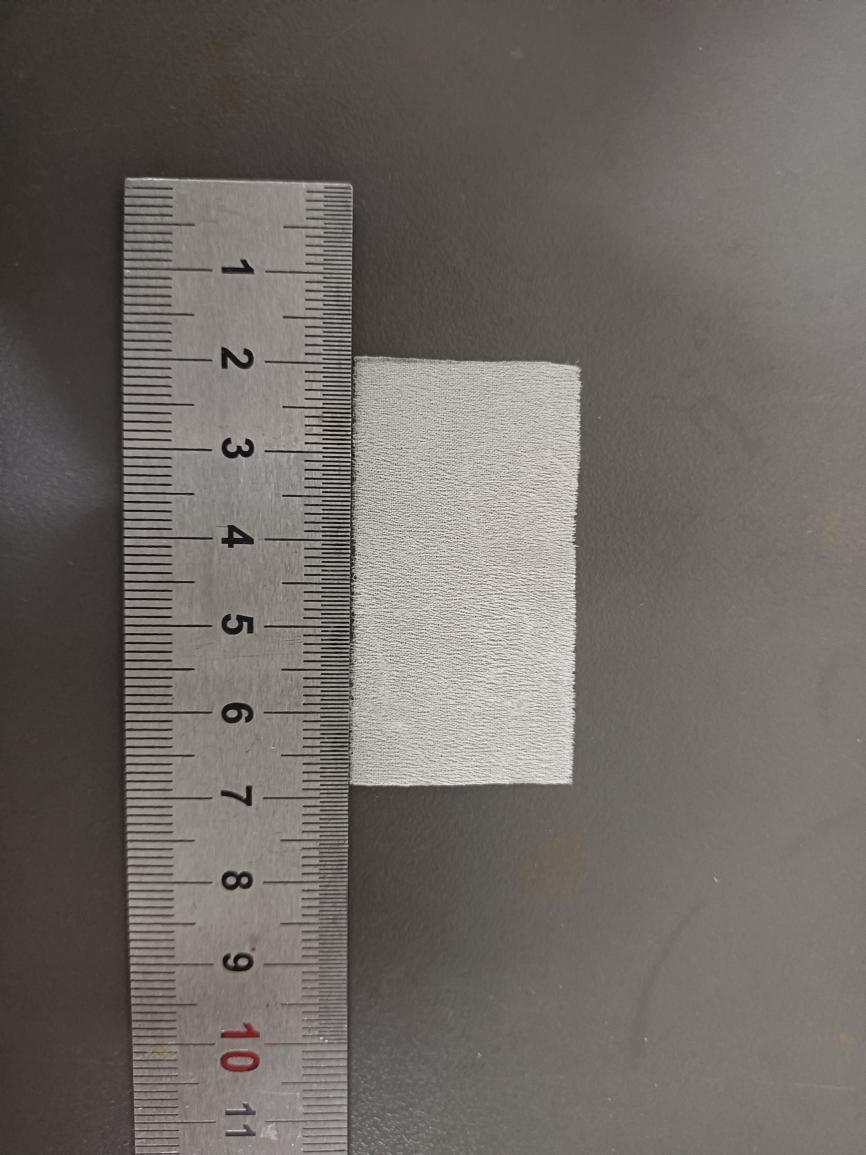
Fig.S23 Digital photograph of simulated rotten/pulverized silk fabric treated with 2% PEI + 4% PPEGDE + 1% IPP


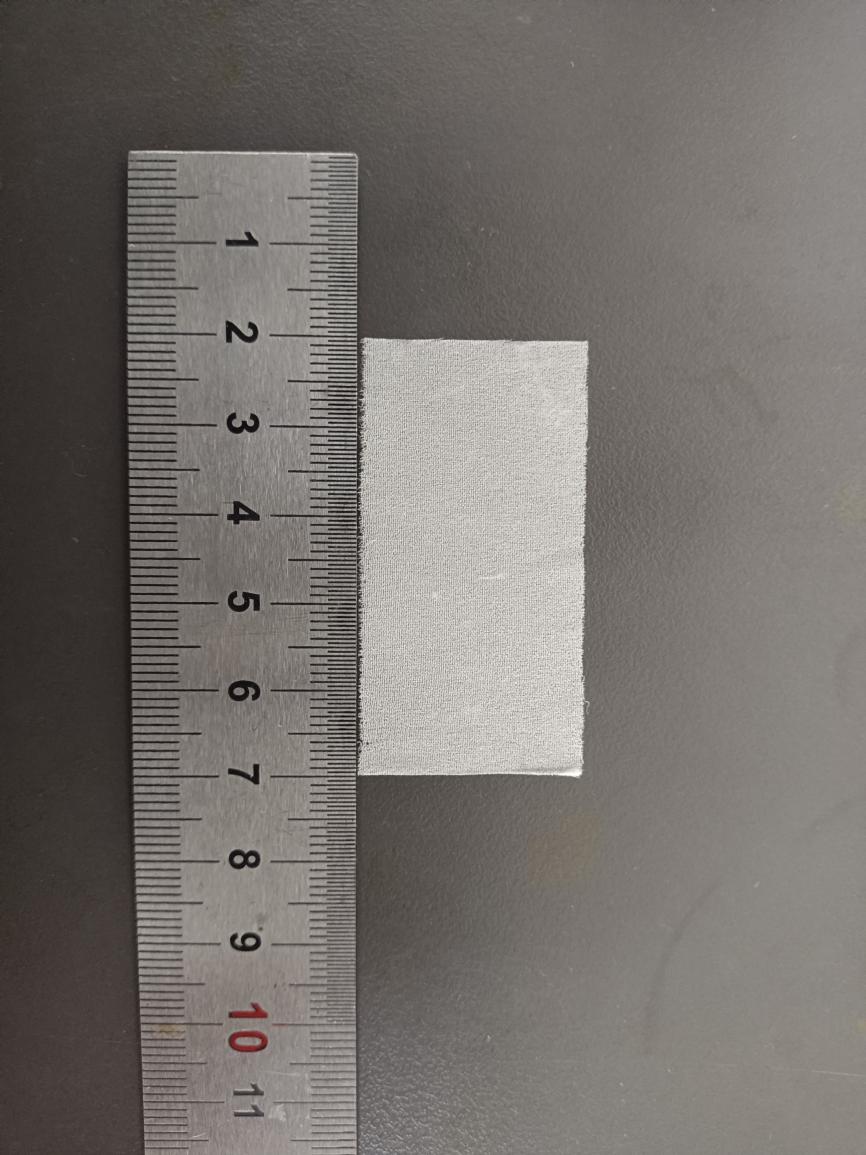
Fig.S24 Digital photograph of simulated rotten/pulverized silk fabric treated with 2% PEI + 4.5% PPEGDE + 1% IPP


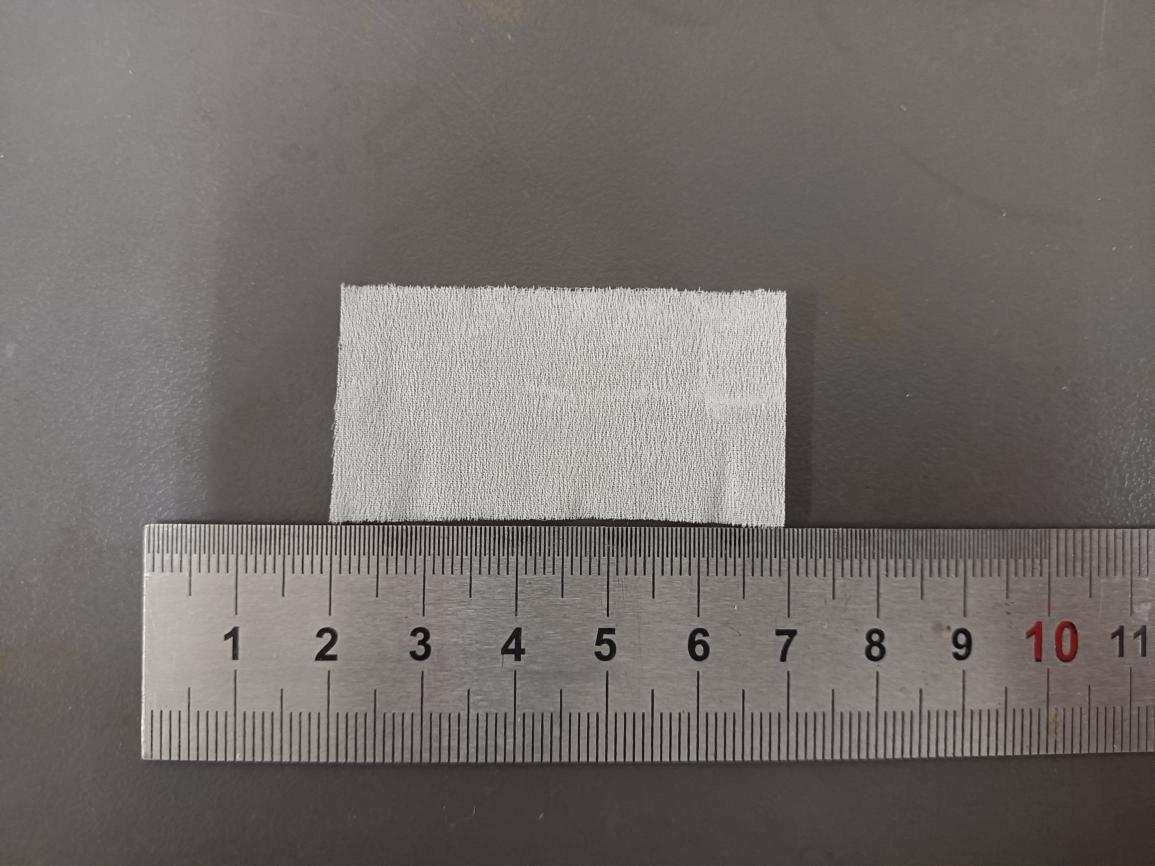


Fig.S25 Digital photograph of simulated rotten/pulverized silk fabric treated with 2% PEI + 5% PPEGDE + 1% IPP


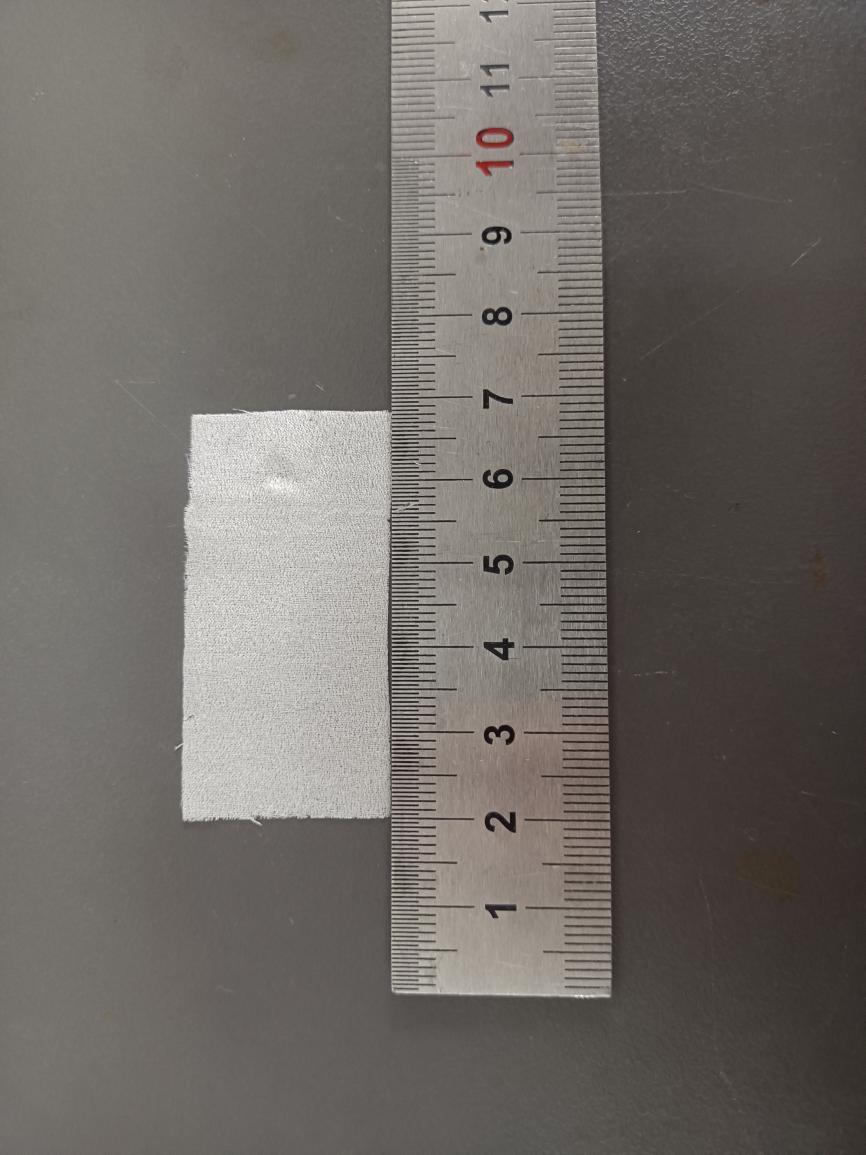
Fig.S26 Digital photograph of simulated rotten/pulverized silk fabric treated with 4% PEI + 0% PPEGDE + 1% IPP


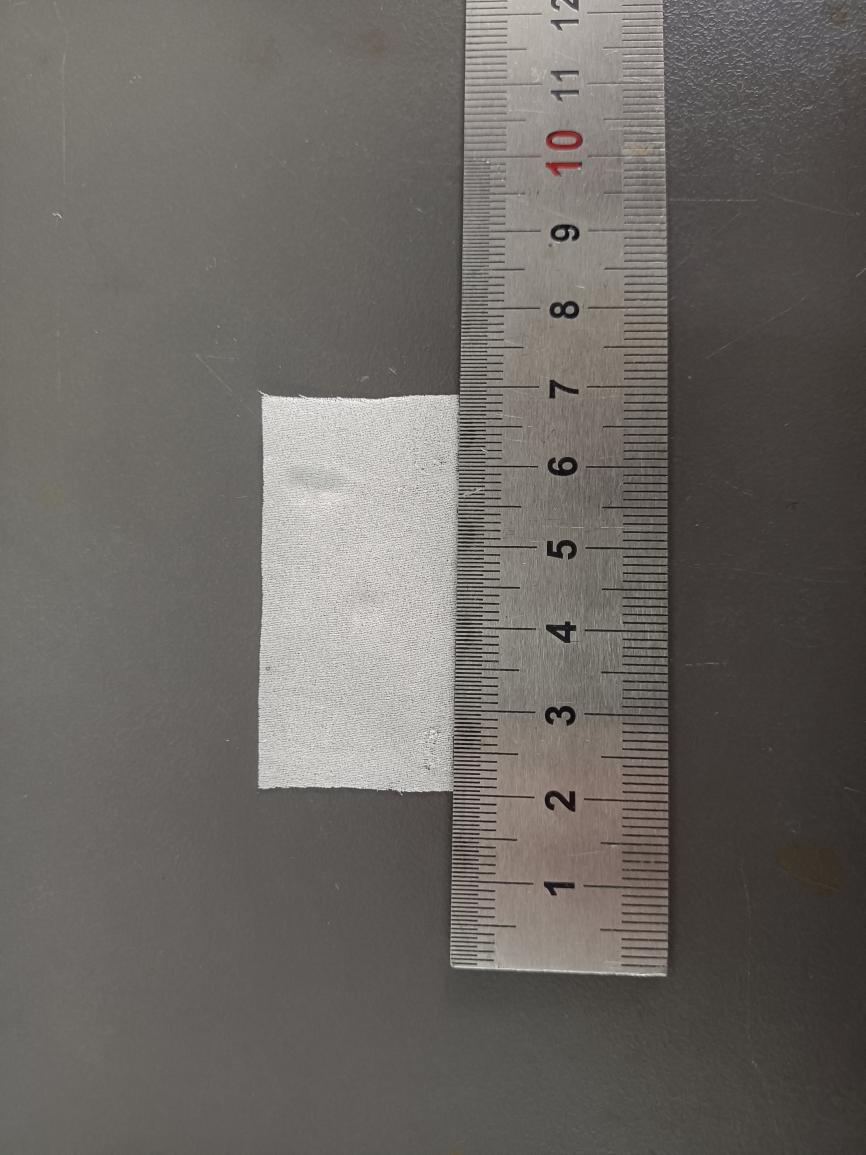
Fig.S27 Digital photograph of simulated rotten/pulverized silk fabric treated with 4% PEI + 0.5% PPEGDE + 1% IPP


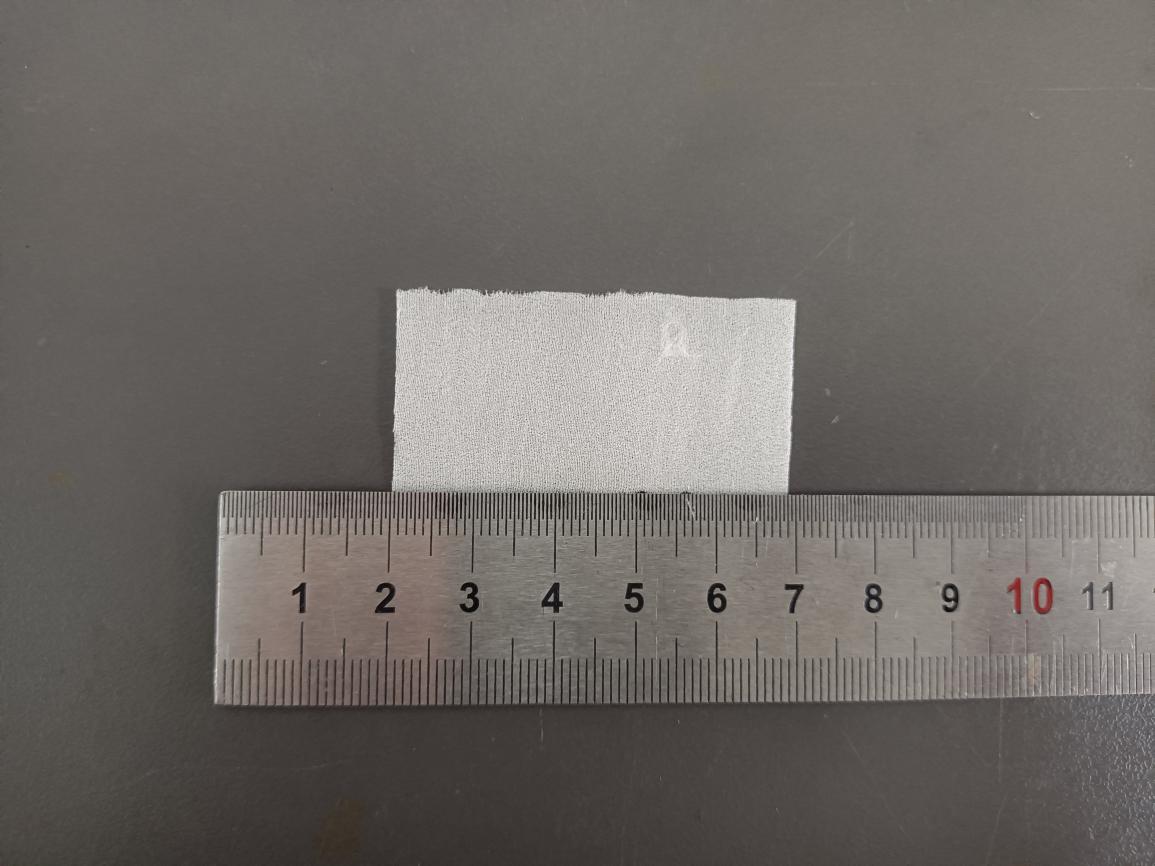


Fig.S28 Digital photograph of simulated rotten/pulverized silk fabric treated with 4% PEI + 1% PPEGDE + 1% IPP


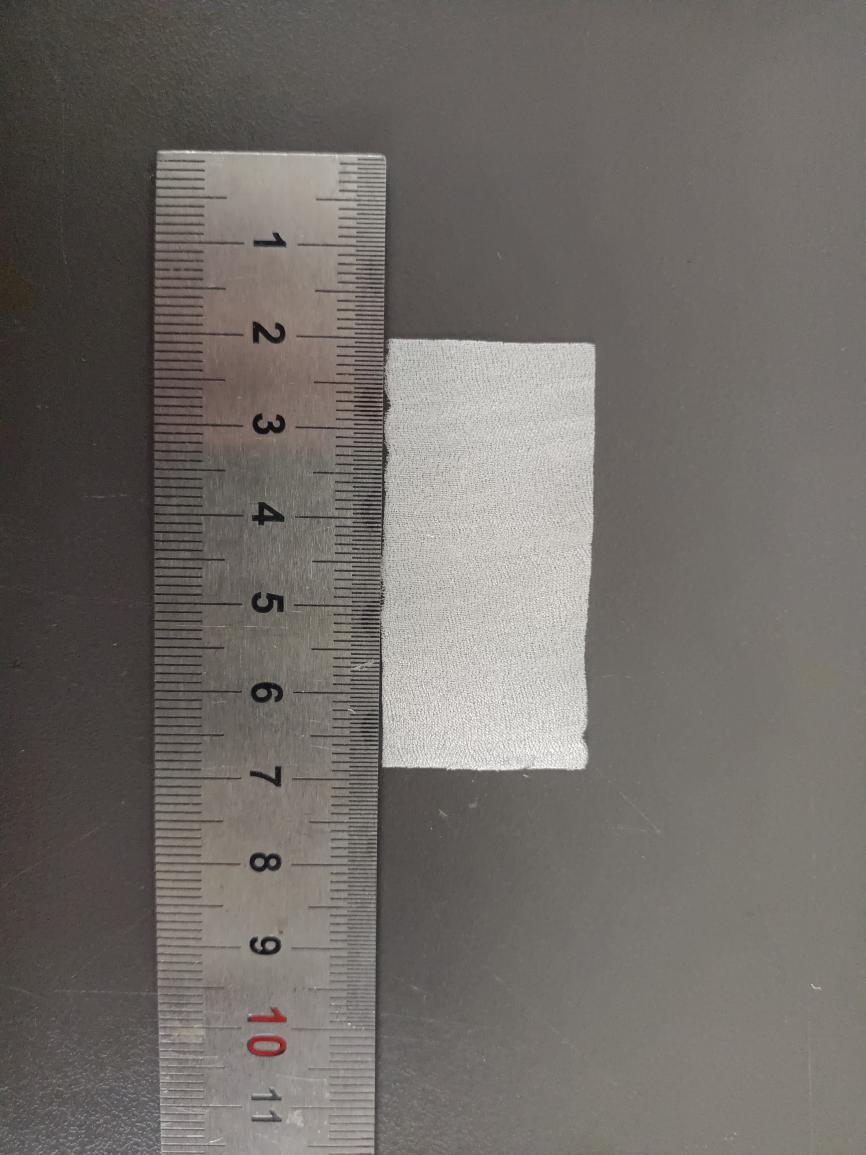
Fig.S29 Digital photograph of simulated rotten/pulverized silk fabric treated with 4% PEI + 1.5% PPEGDE + 1% IPP


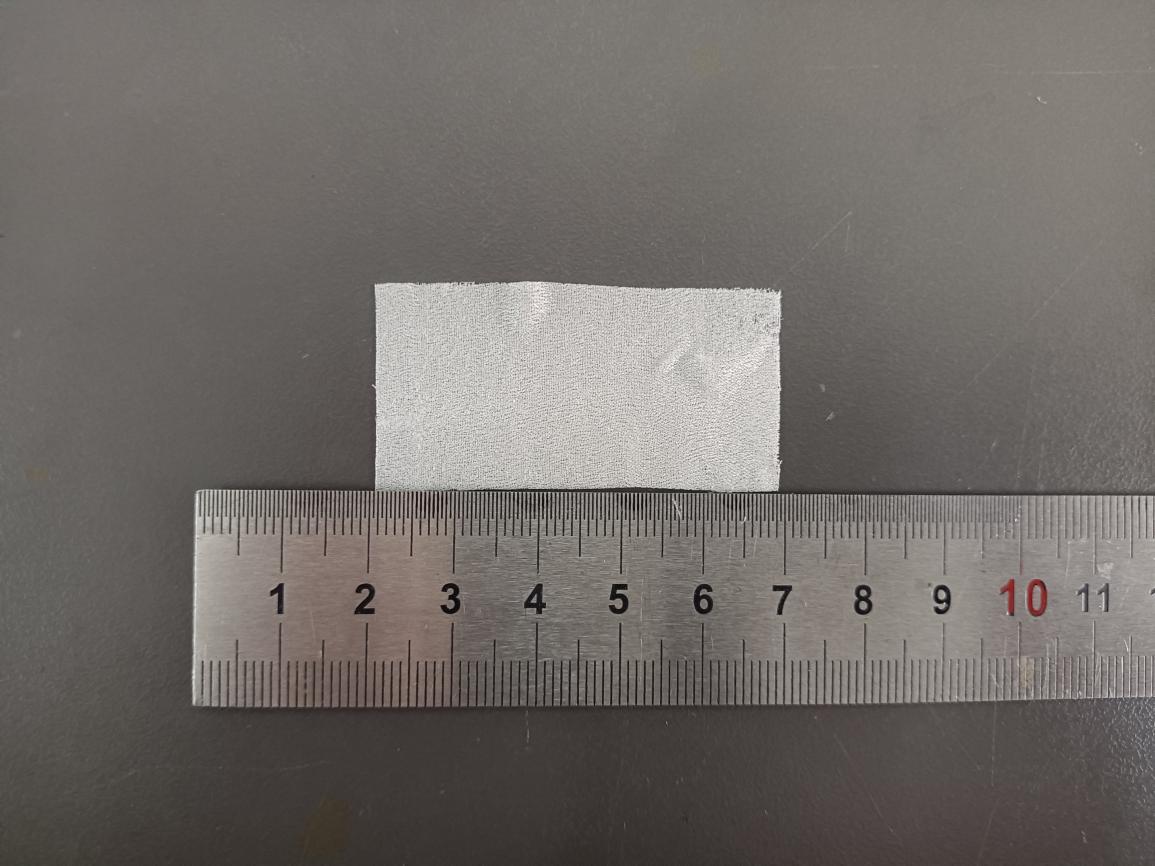


Fig.S30 Digital photograph of simulated rotten/pulverized silk fabric treated with 4% PEI + 2% PPEGDE + 1% IPP

Fig.S31 Digital photograph of simulated rotten/pulverized silk fabric treated with 4% PEI + 2.5% PPEGDE + 1% IPP
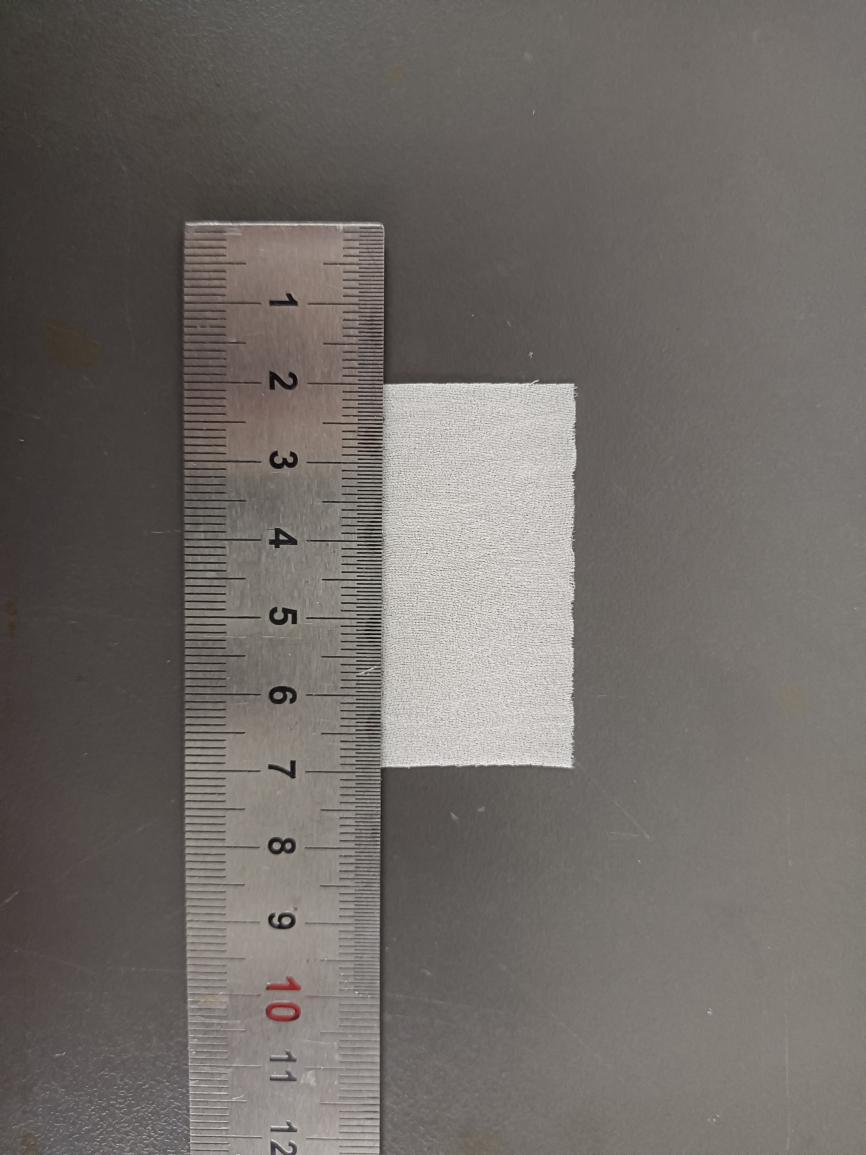


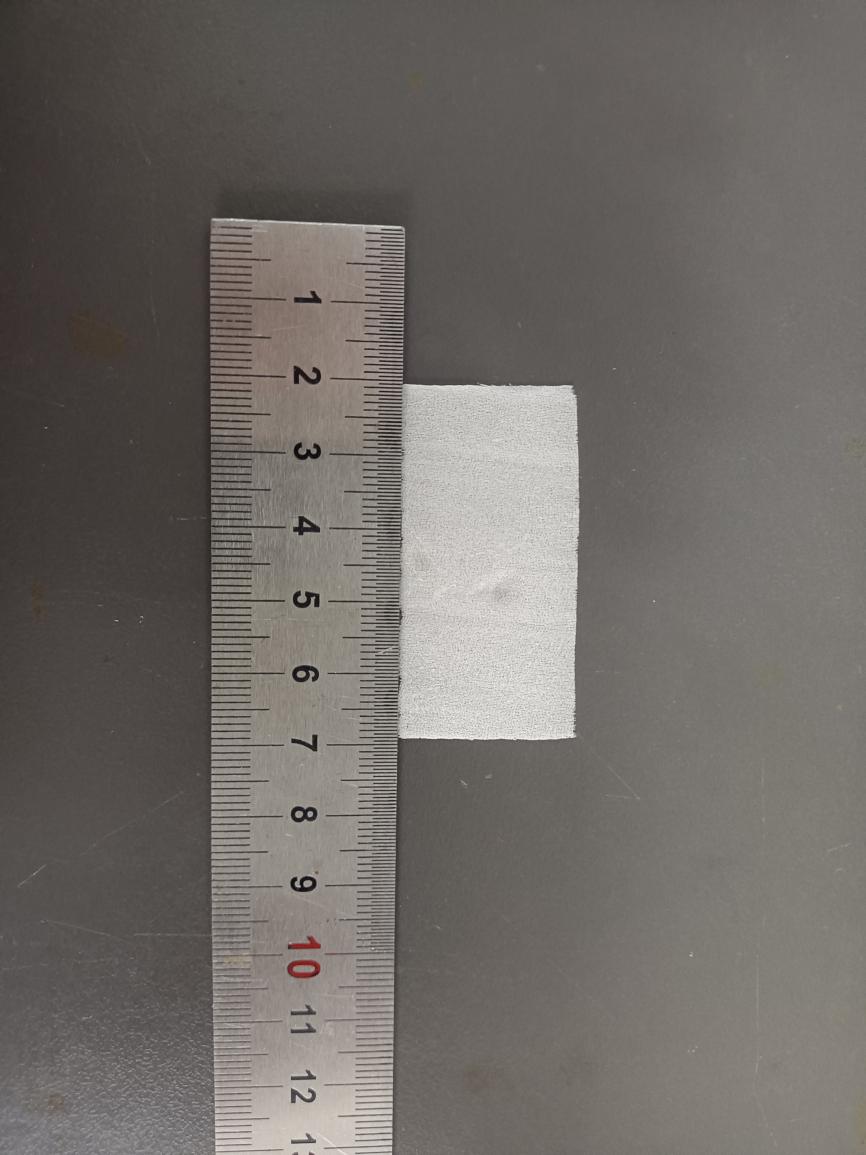
Fig.S31 Digital photograph of simulated rotten/pulverized silk fabric treated with 4% PEI + 3% PPEGDE + 1% IPP


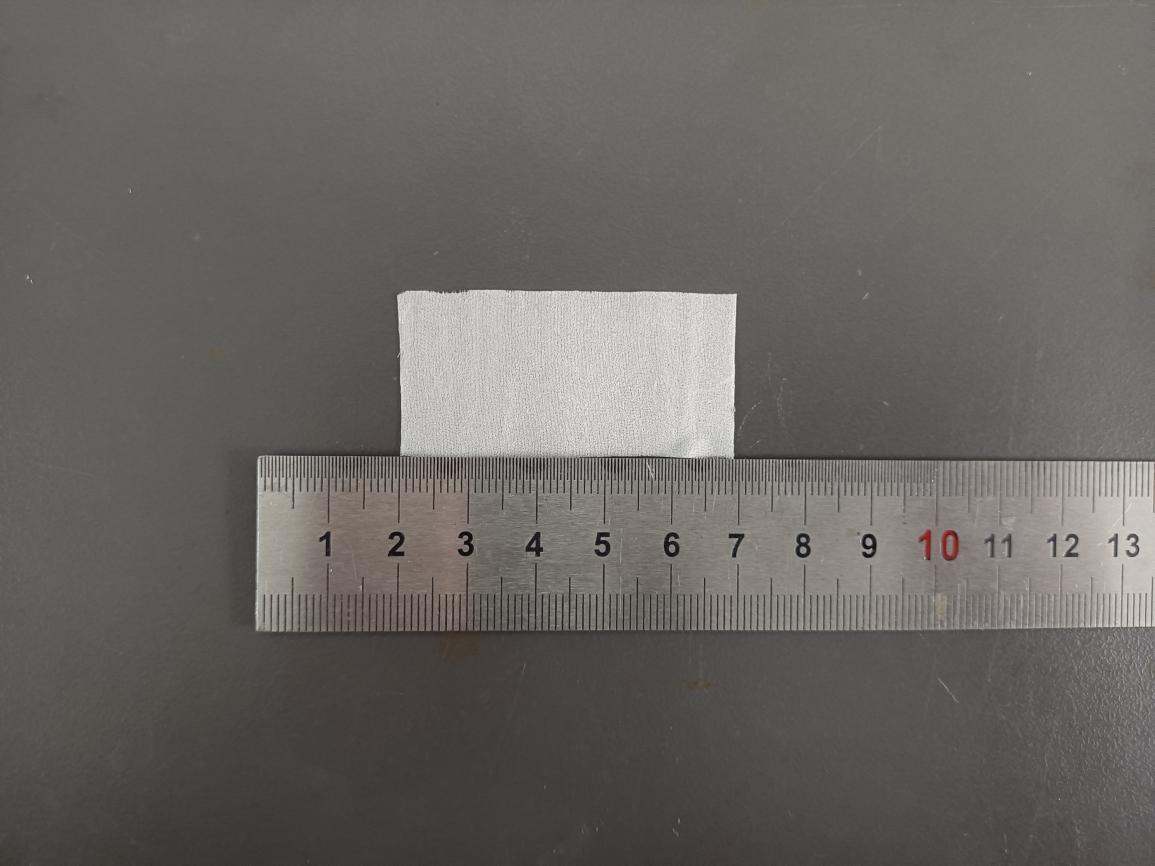


Fig.S32 Digital photograph of simulated rotten/pulverized silk fabric treated with 4% PEI + 3.5% PPEGDE + 1% IPP


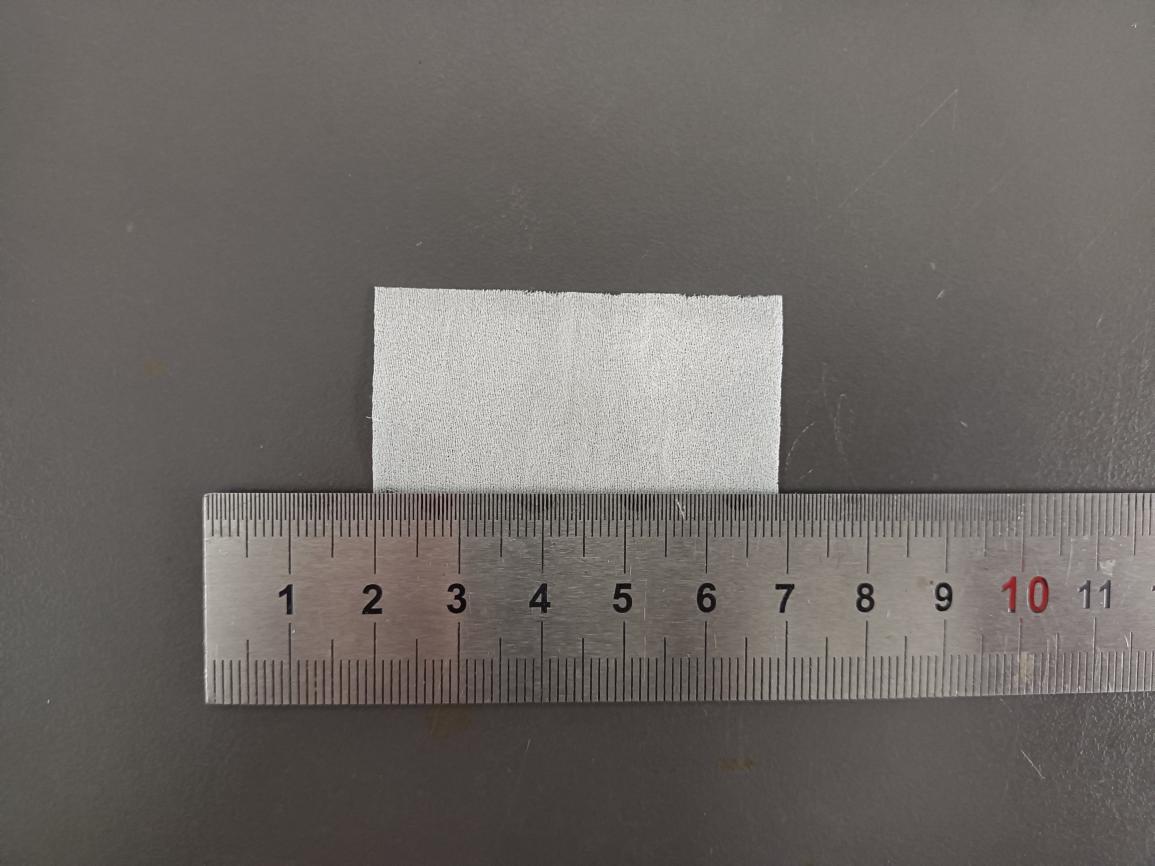


Fig.S33 Digital photograph of simulated rotten/pulverized silk fabric treated with 4% PEI + 4%PPEGDE +1% IPP


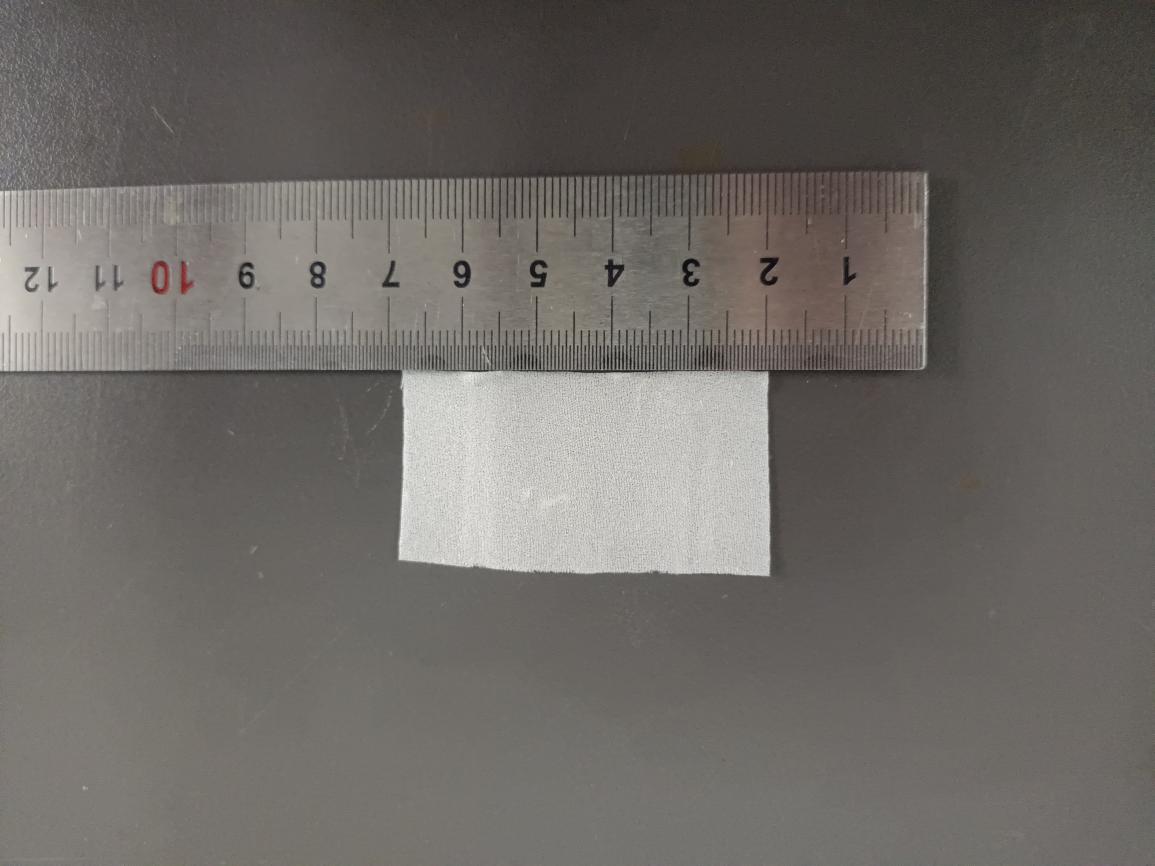


Fig.S34 Digital photograph of simulated rotten/pulverized silk fabric treated with 4% PEI + 4.5% PPEGDE + 1%IPP


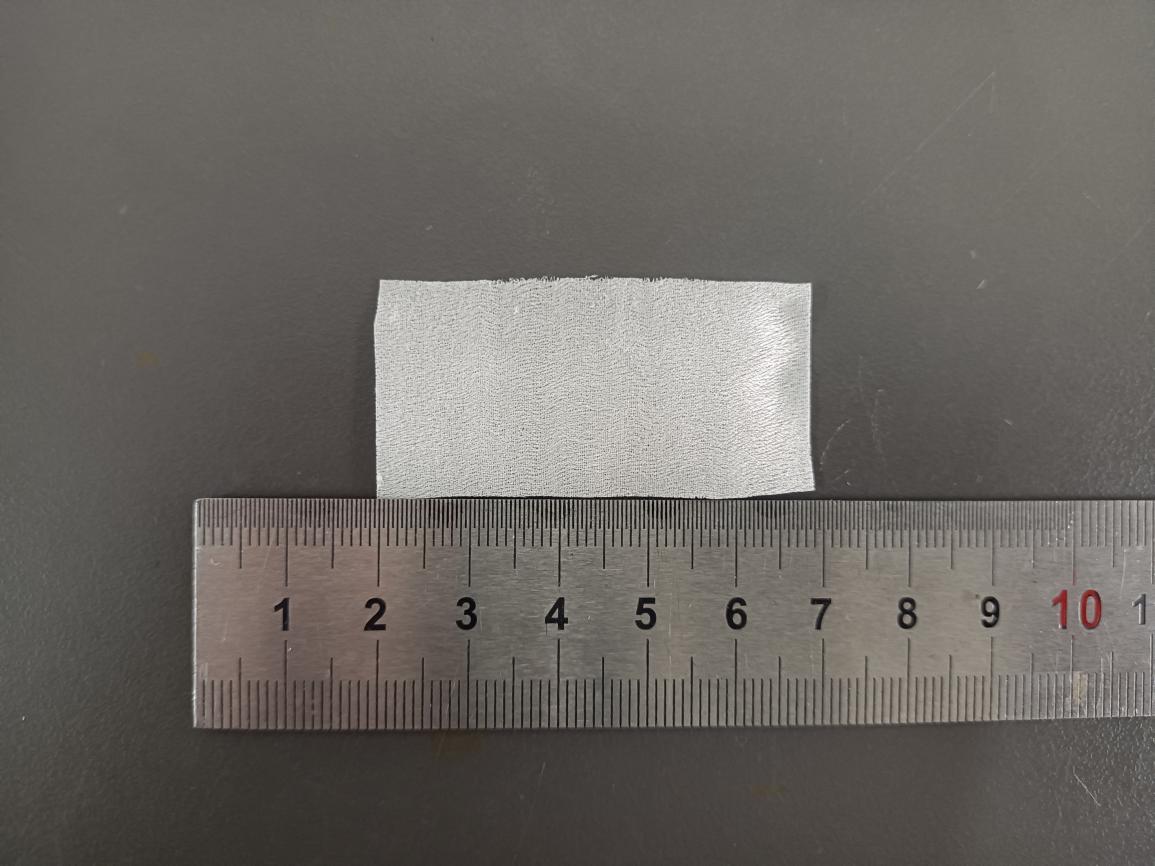


Fig.S35 Digital photograph of simulated rotten/pulverized silk fabric treated with 4% PEI + 5% PPEGDE + 1% IPP


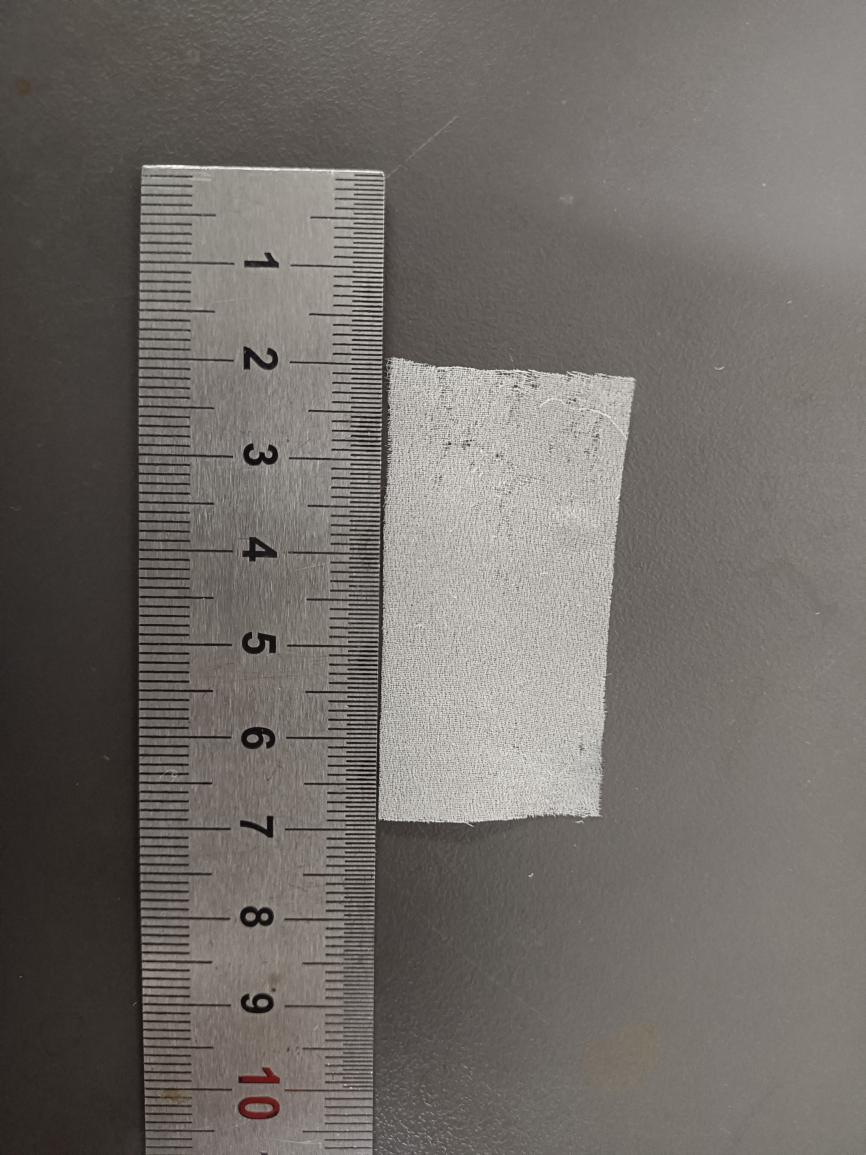
Fig.S36 Digital photograph of simulated rotten/pulverized silk fabric treated with 6% PEI+ 0% PPEGDE + 1% IPP


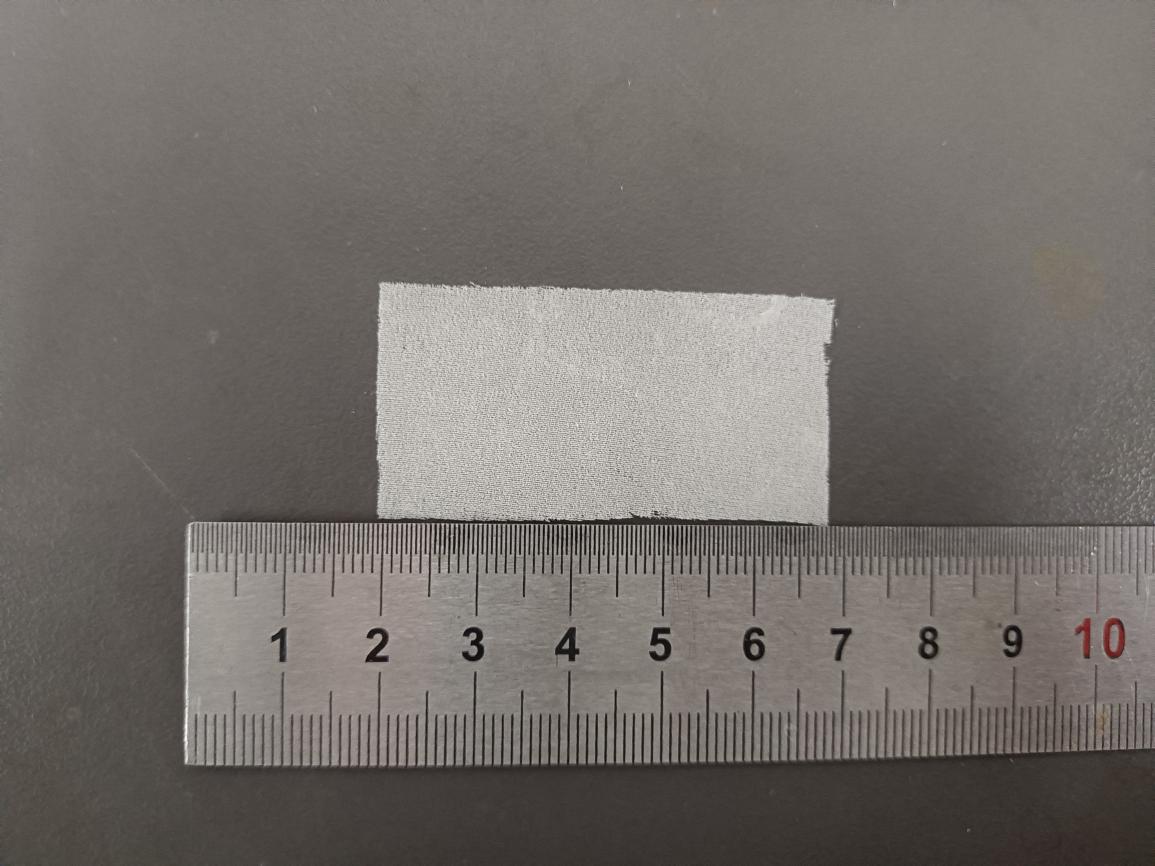


Fig.S37 Digital photograph of simulated rotten/pulverized silk fabric treated with 6% PEI + 0.5% PPEGDE + 1% IPP


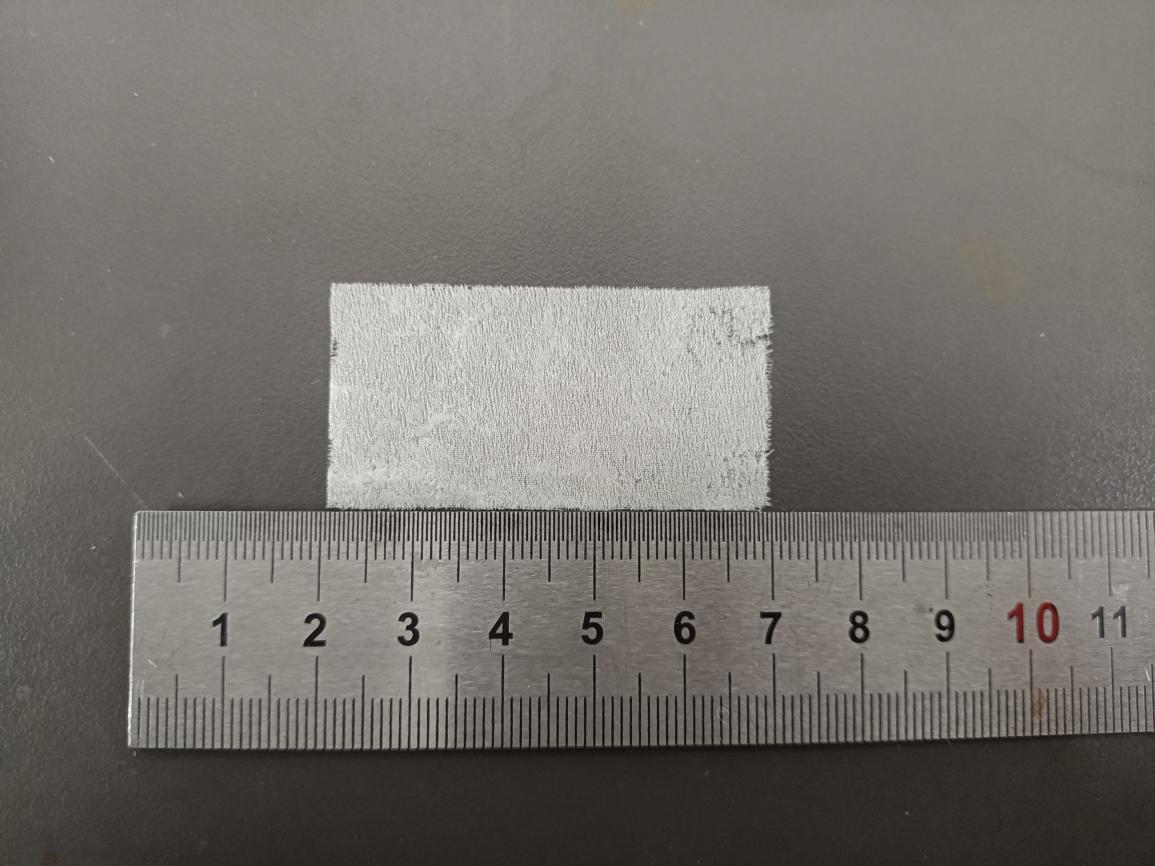


Fig.S38 Digital photograph of simulated rotten/pulverized silk fabric treated with 6% PEI + 1% PPEGDE + 1% IPP


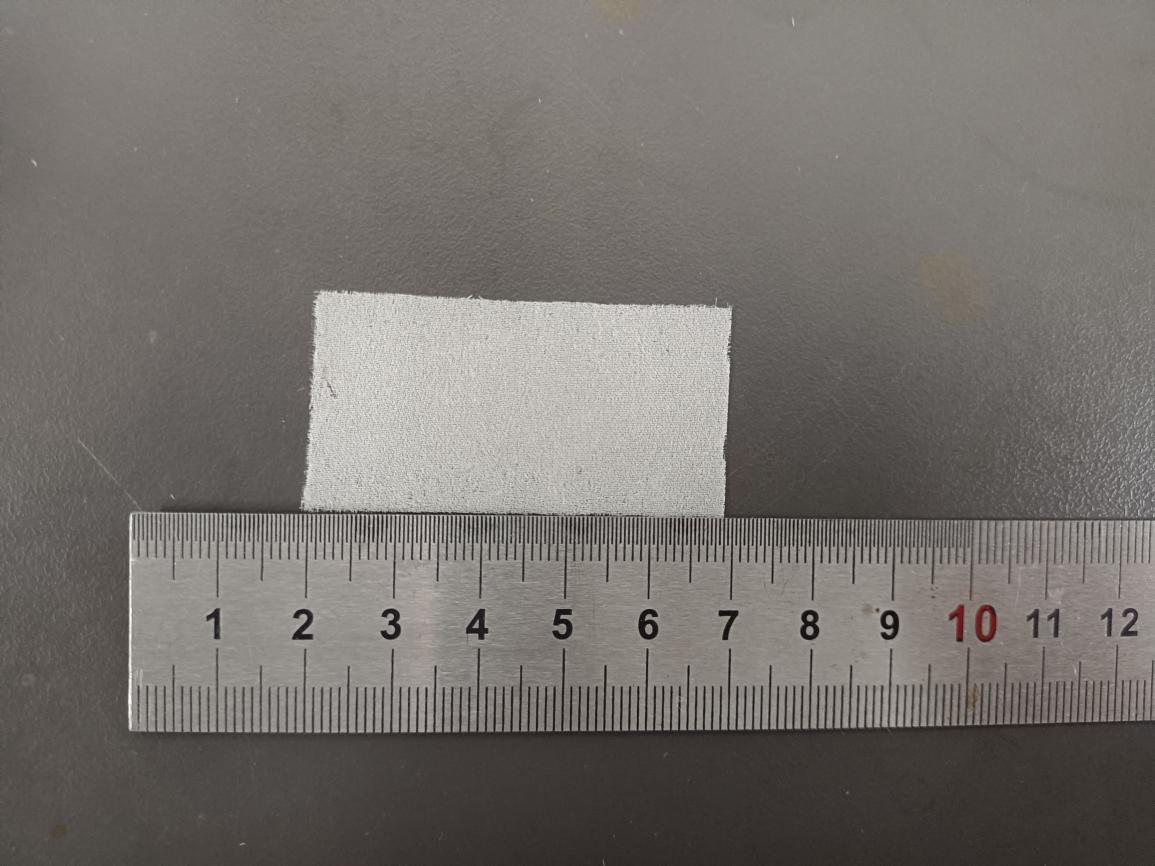


Fig.S39 Digital photograph of simulated rotten/pulverized silk fabric treated with 6% PEI + 1.5% PPEGDE + 1% IPP


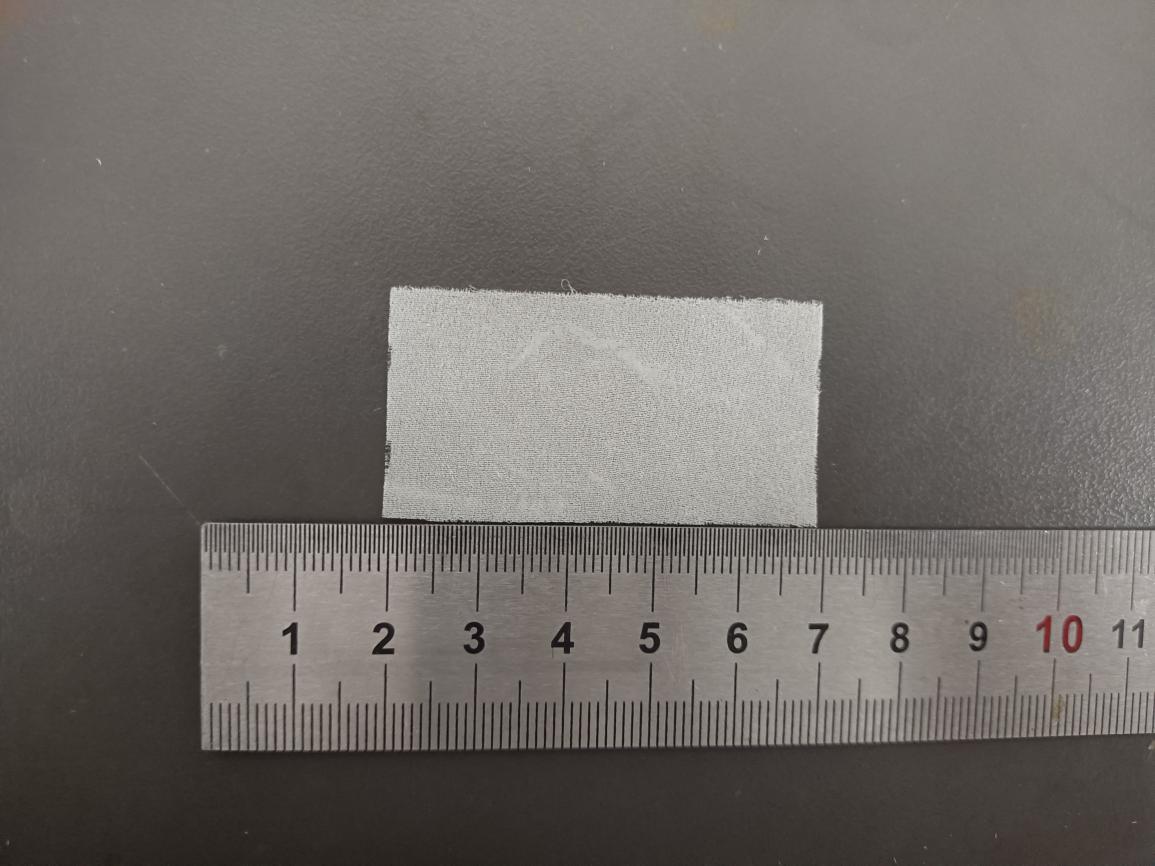


Fig.S40 Digital photograph of simulated rotten/pulverized silk fabric treated with 6% PEI + 2% PPEGDE + 1% IPP


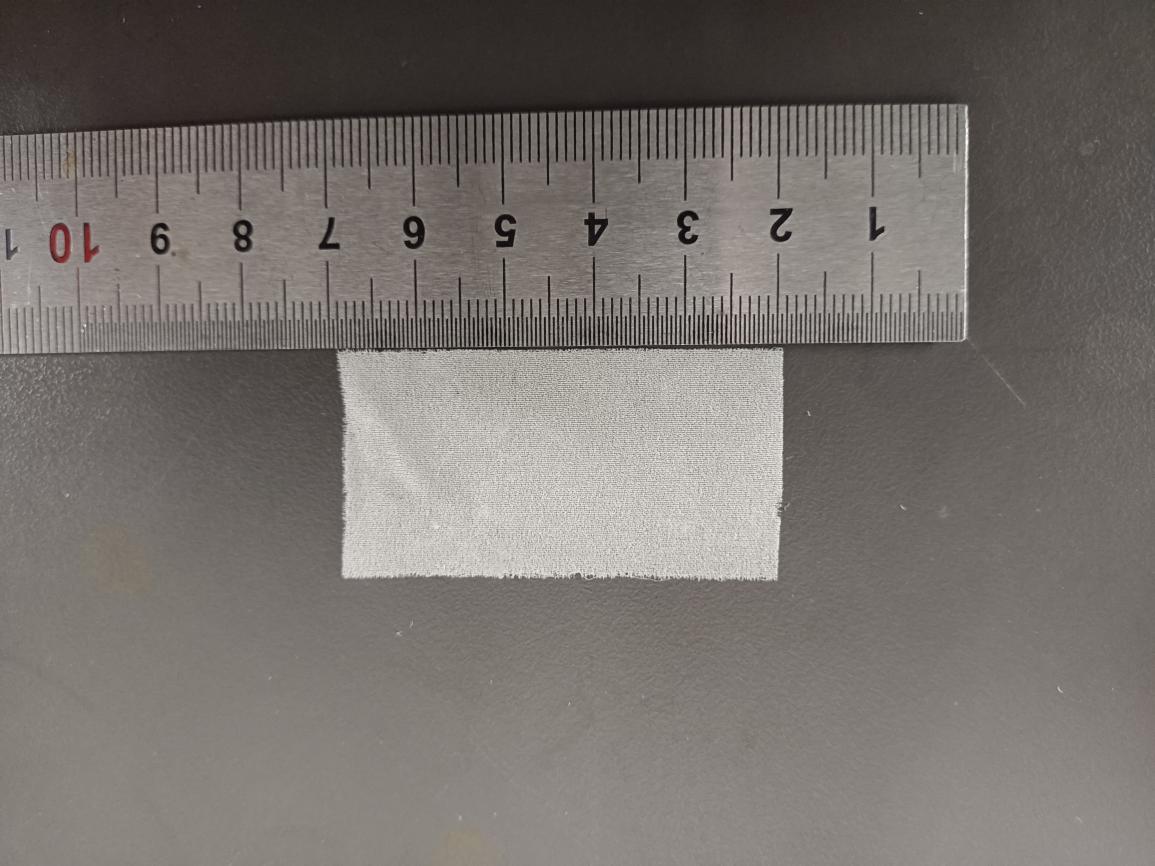


Fig.S41 Digital photograph of simulated rotten/pulverized silk fabric treated with 6% PEI + 2.5% PPEGDE + 1% IPP

Fig.S42 Digital photograph of simulated rotten/pulverized silk fabric treated with
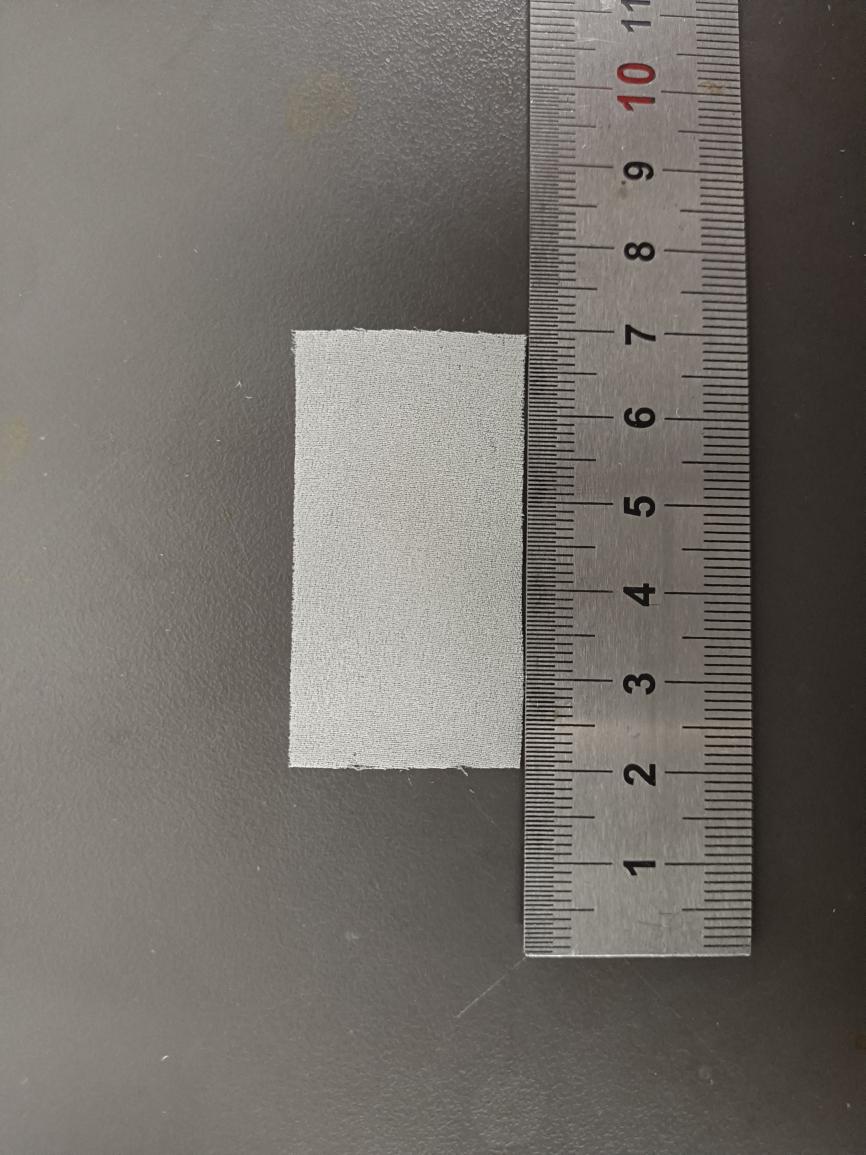
6% PEI + 3% PPEGDE + 1% IPP


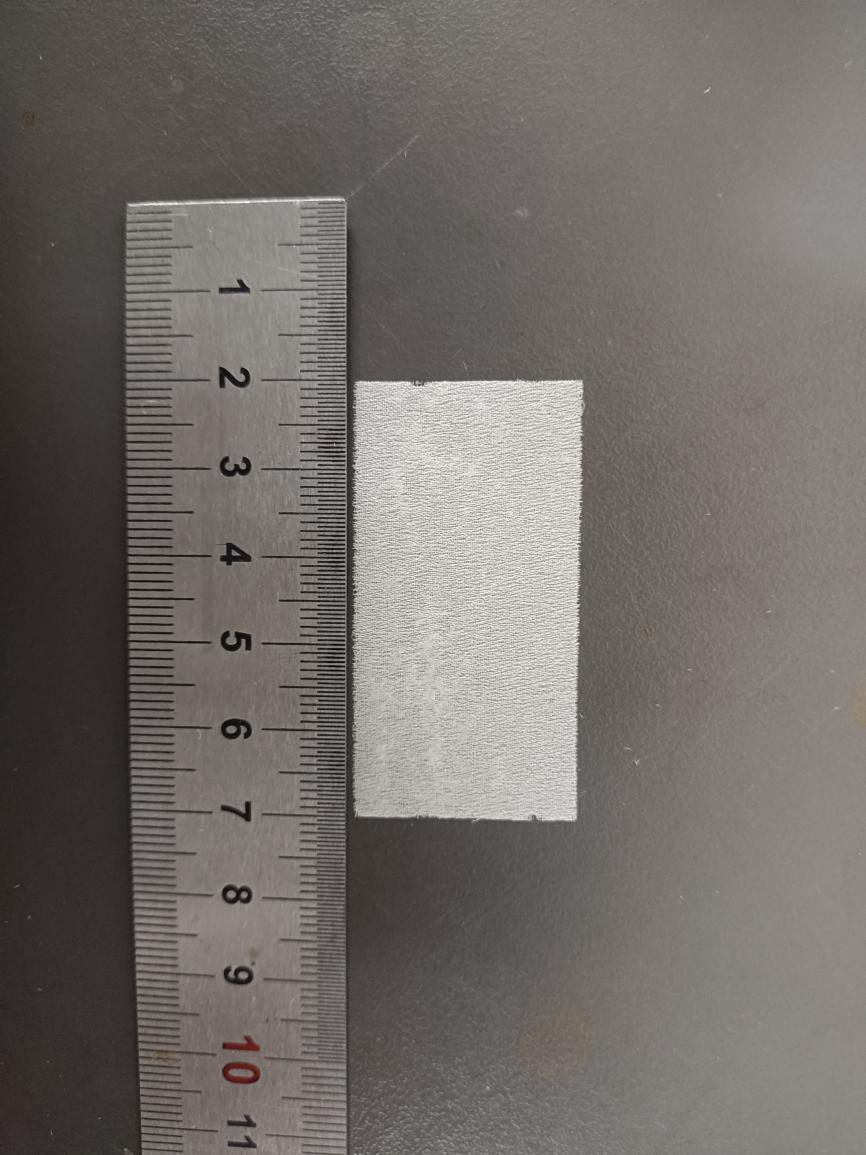
Fig.S43 Digital photograph of simulated rotten/pulverized silk fabric treated with 6% PEI + 3.5% PPEGDE + 1% IPP


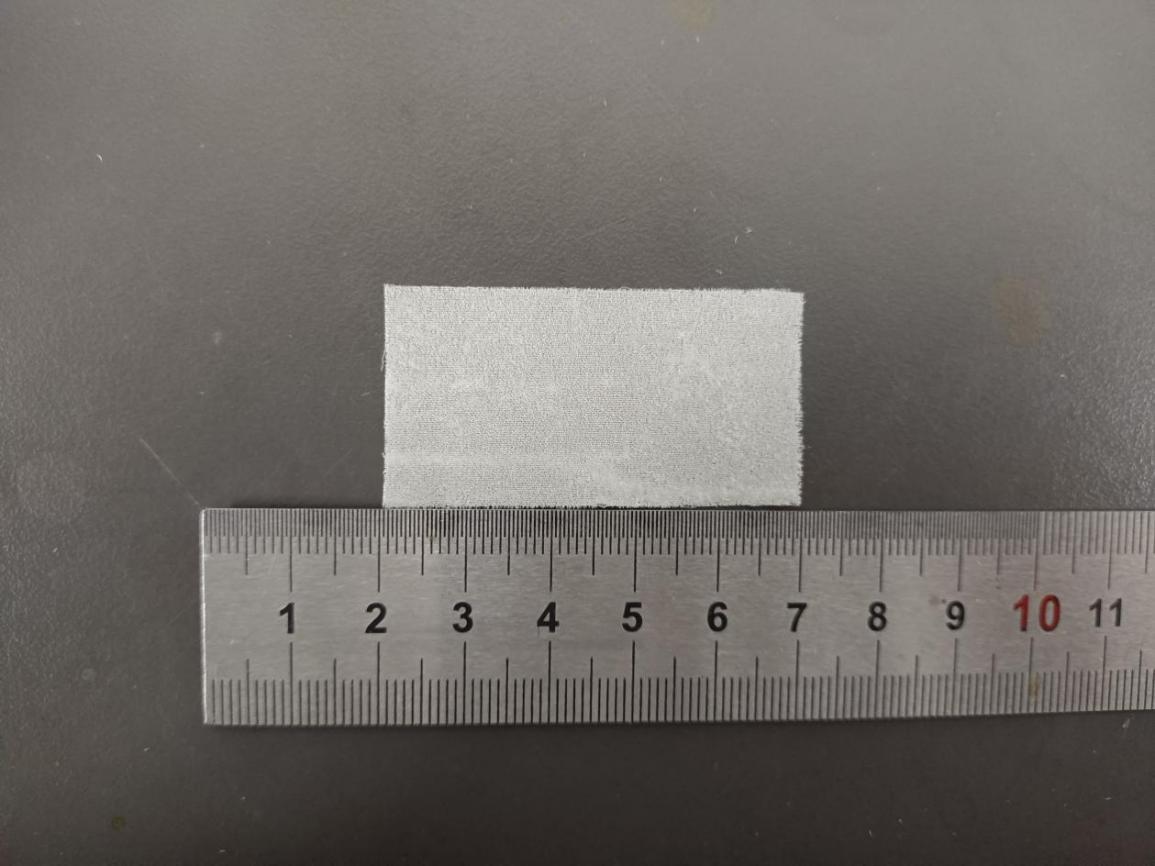


Fig.S44 Digital photograph of simulated rotten/pulverized silk fabric treated with 6% PEI + 4% PPEGDE + 1% IPP


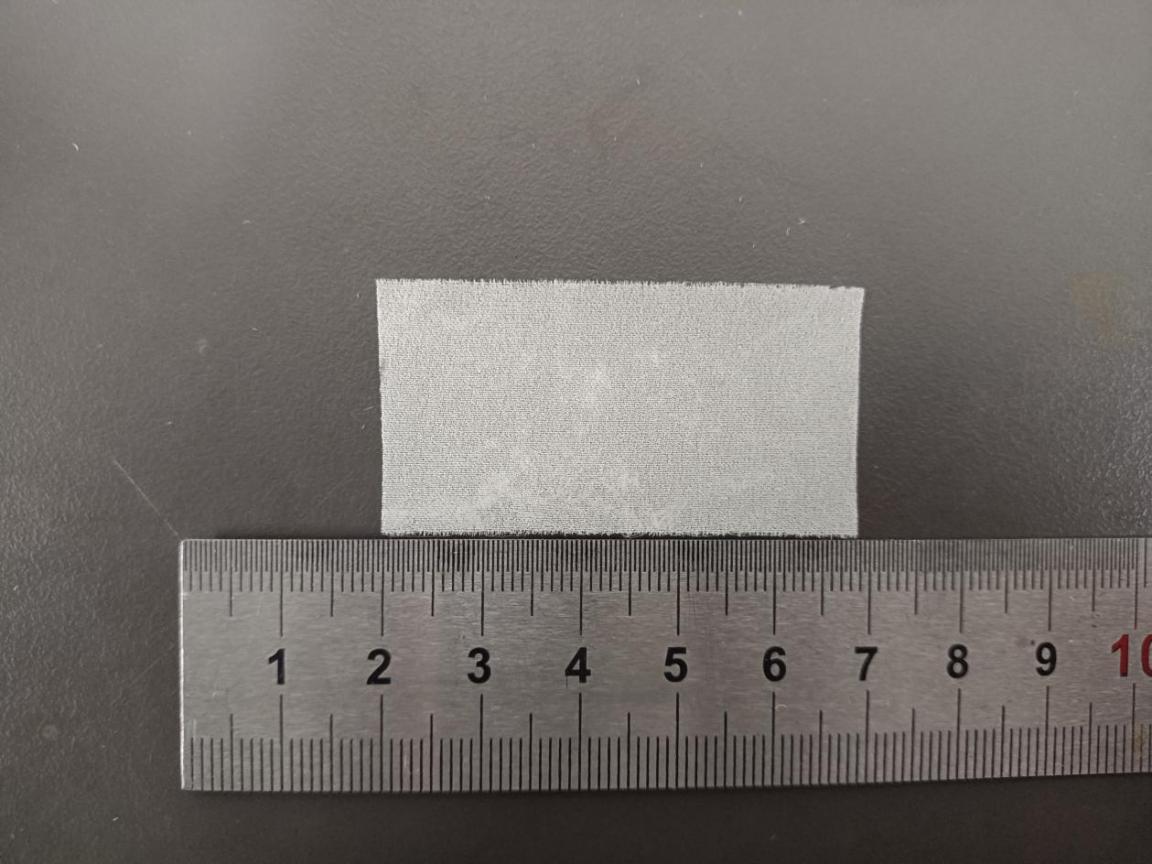


Fig.S45 Digital photograph of simulated rotten/pulverized silk fabric treated with 6% PEI + 4.5% PPEGDE + 1% IPP


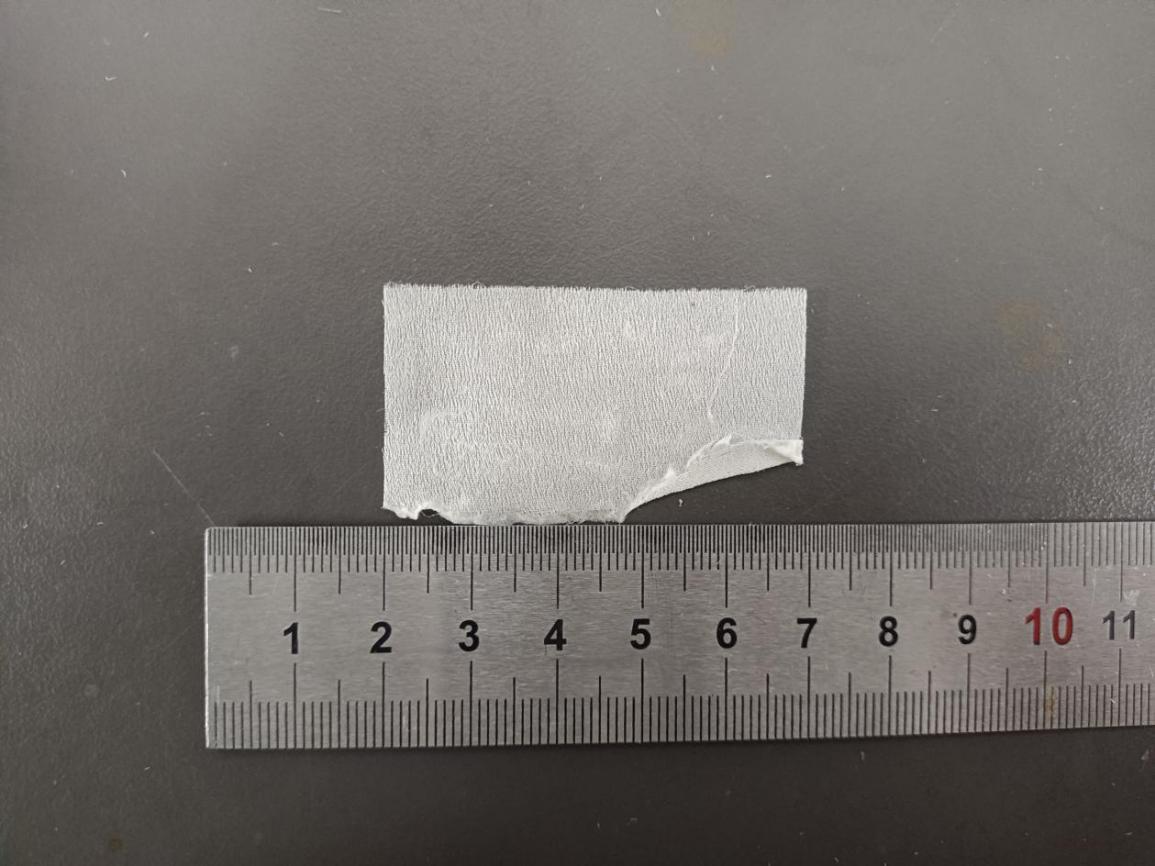


Fig.S46 Digital photograph of simulated rotten/pulverized silk fabric treated with 6% PEI + 5% PPEGDE + 1% IPP


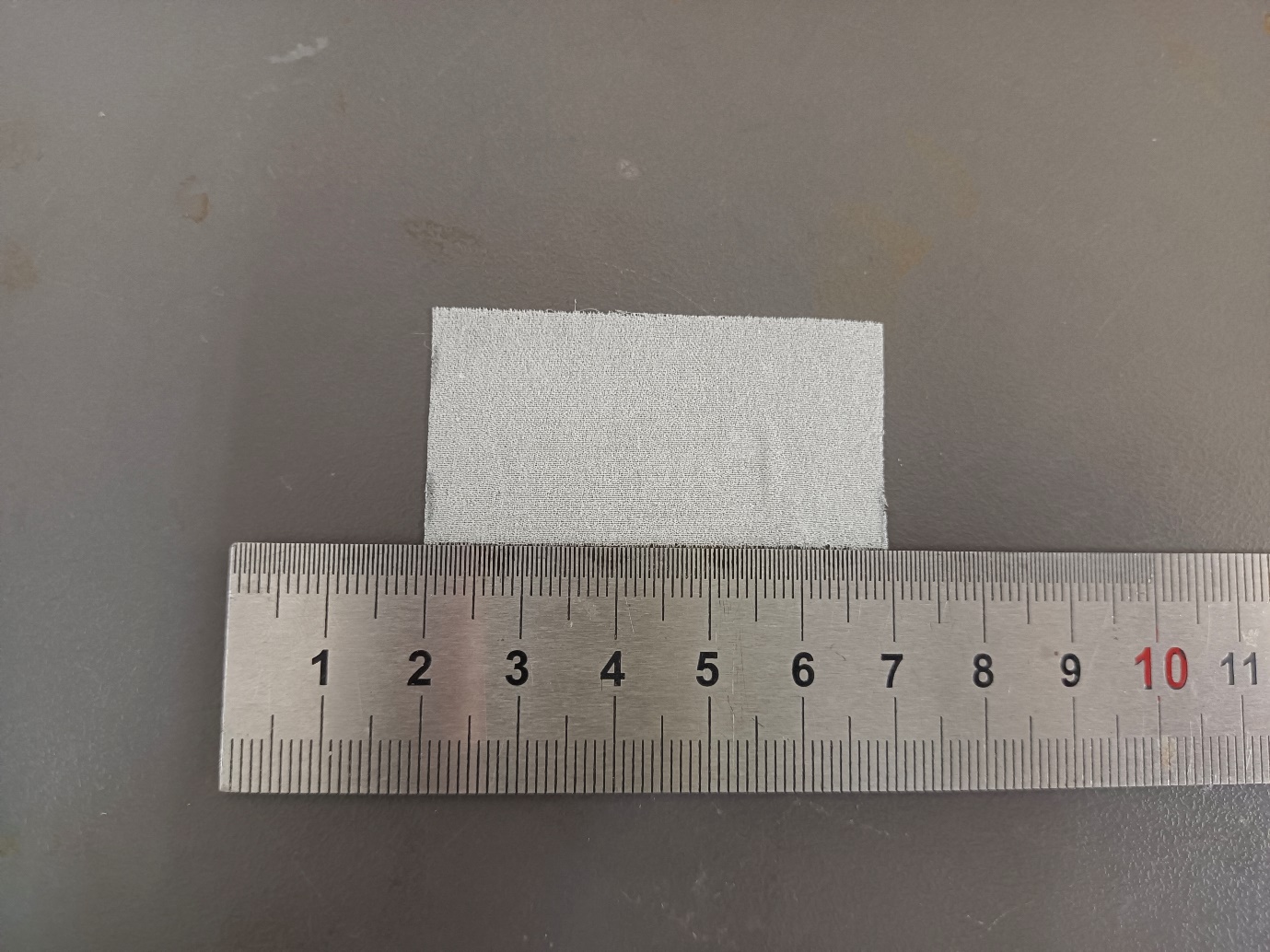


Fig.S47 Digital photograph of simulated rotten/pulverized silk fabric treated with 8% PEI + 0% PPEGDE + 1% IPP


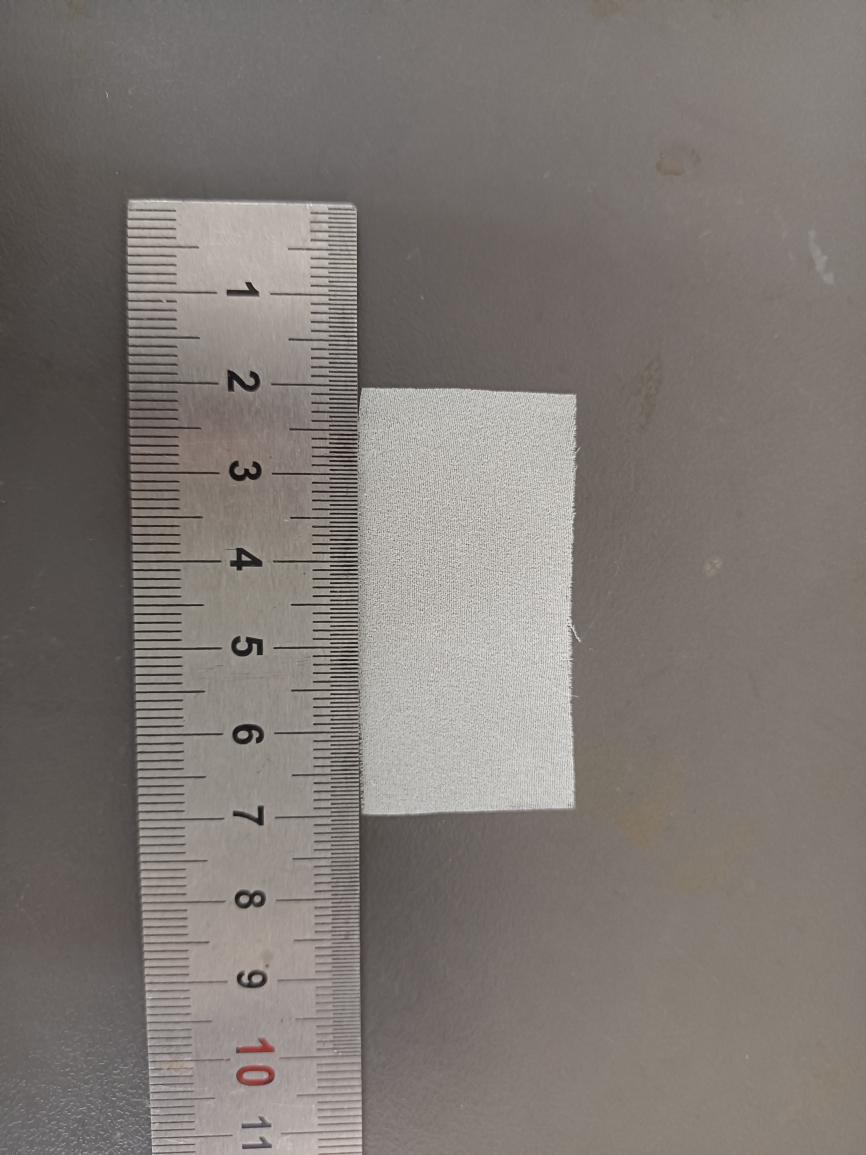
Fig.S48 Digital photograph of simulated rotten/pulverized silk fabric treated with 8% PEI + 0.5% PPEGDE + 1% IPP


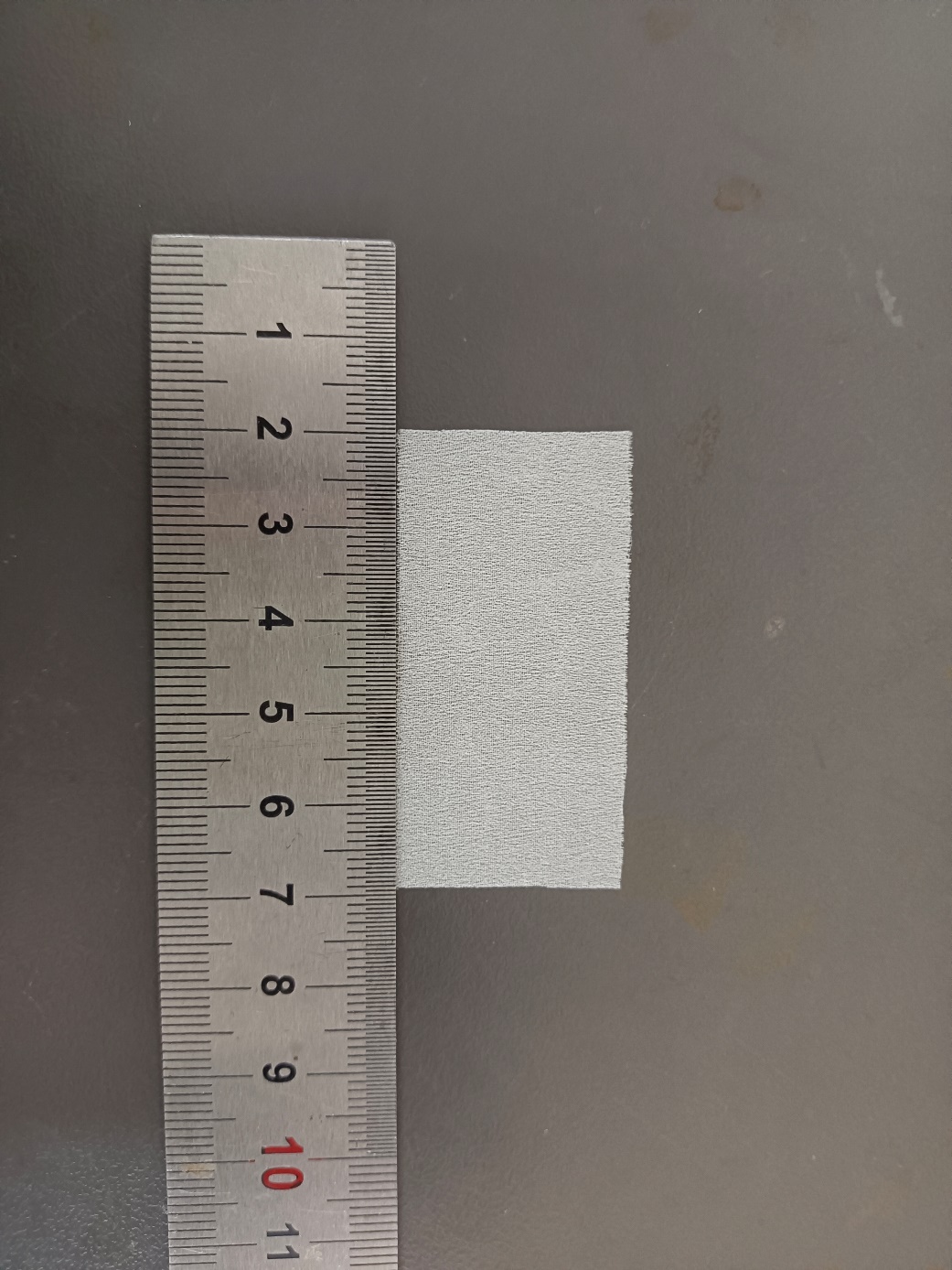
Fig.S49 Digital photograph of simulated rotten/pulverized silk fabric treated with 8% PEI + 1% PPEGDE + 1% IPP


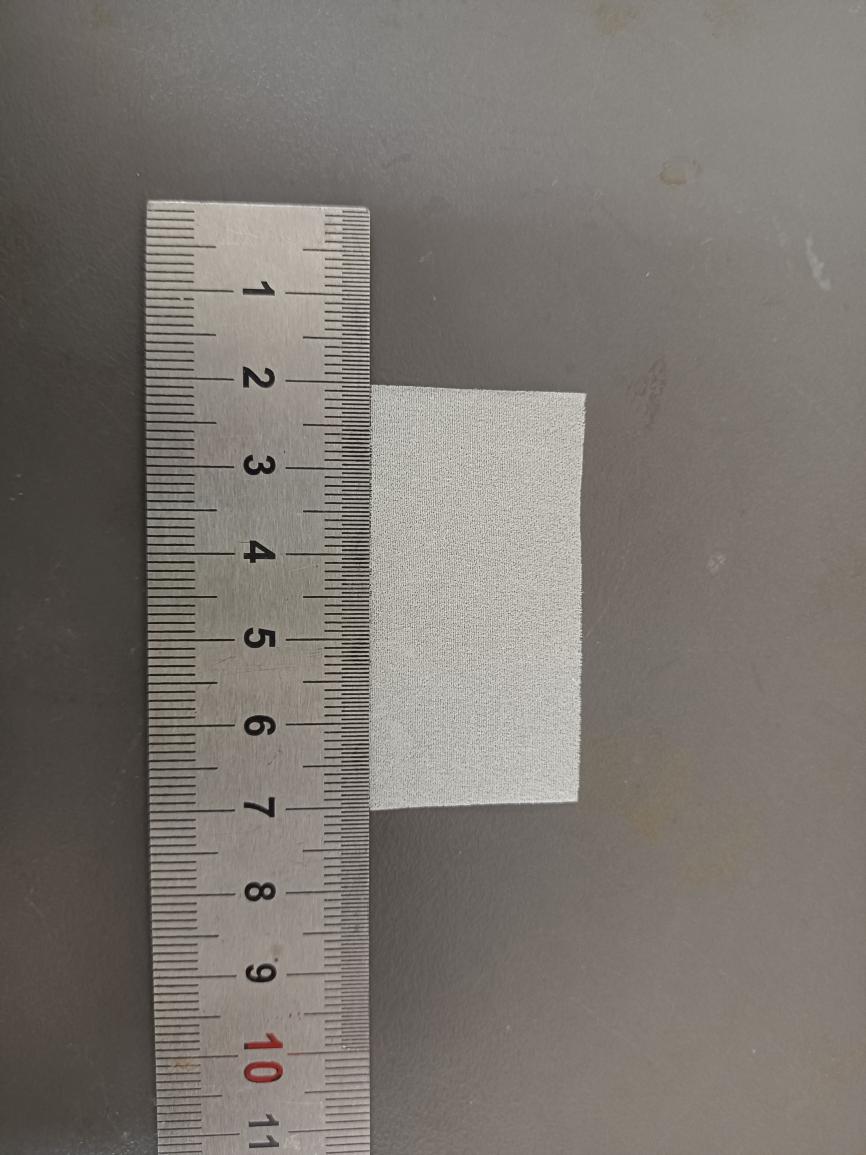
Fig.S50 Digital photograph of simulated rotten/pulverized silk fabric treated with 8% PEI + 1.5% PPEGDE + 1% IPP


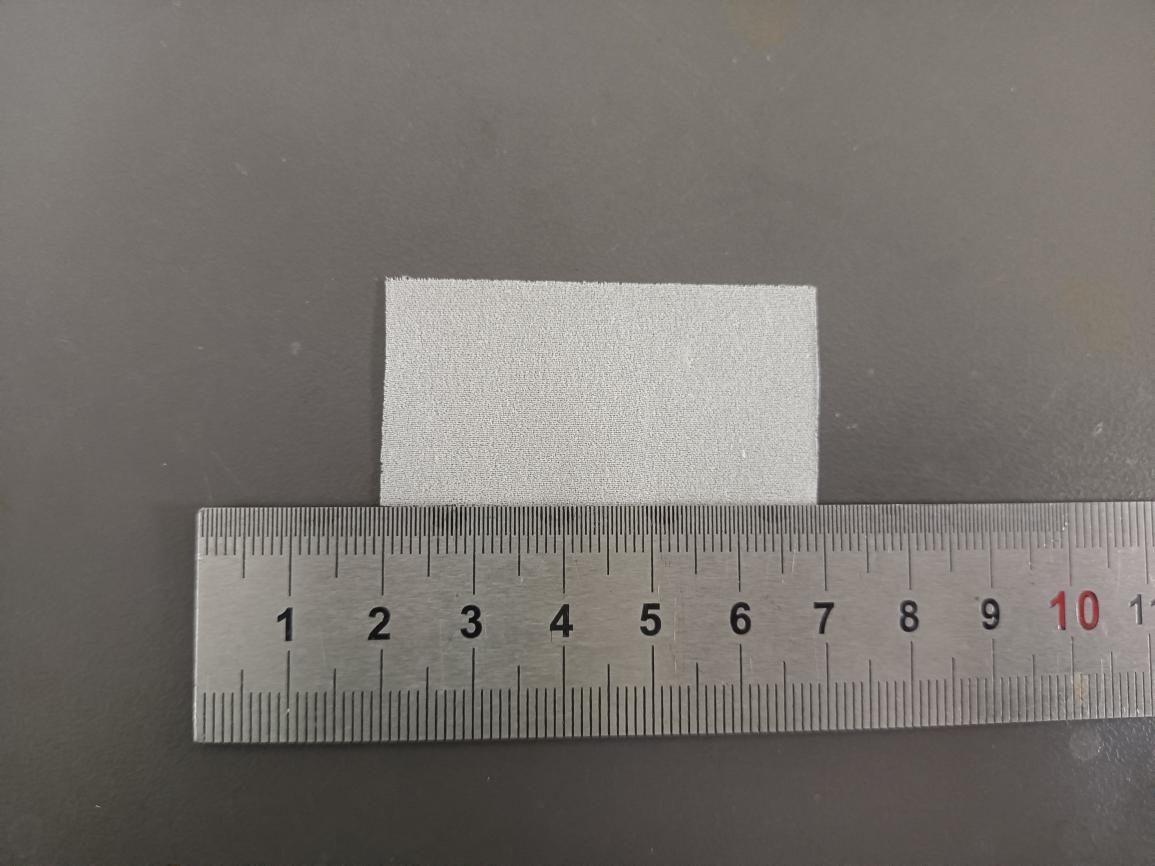


Fig.S51 Digital photograph of simulated rotten/pulverized silk fabric treated with 8% PEI +2% PPEGDE + 1% IPP


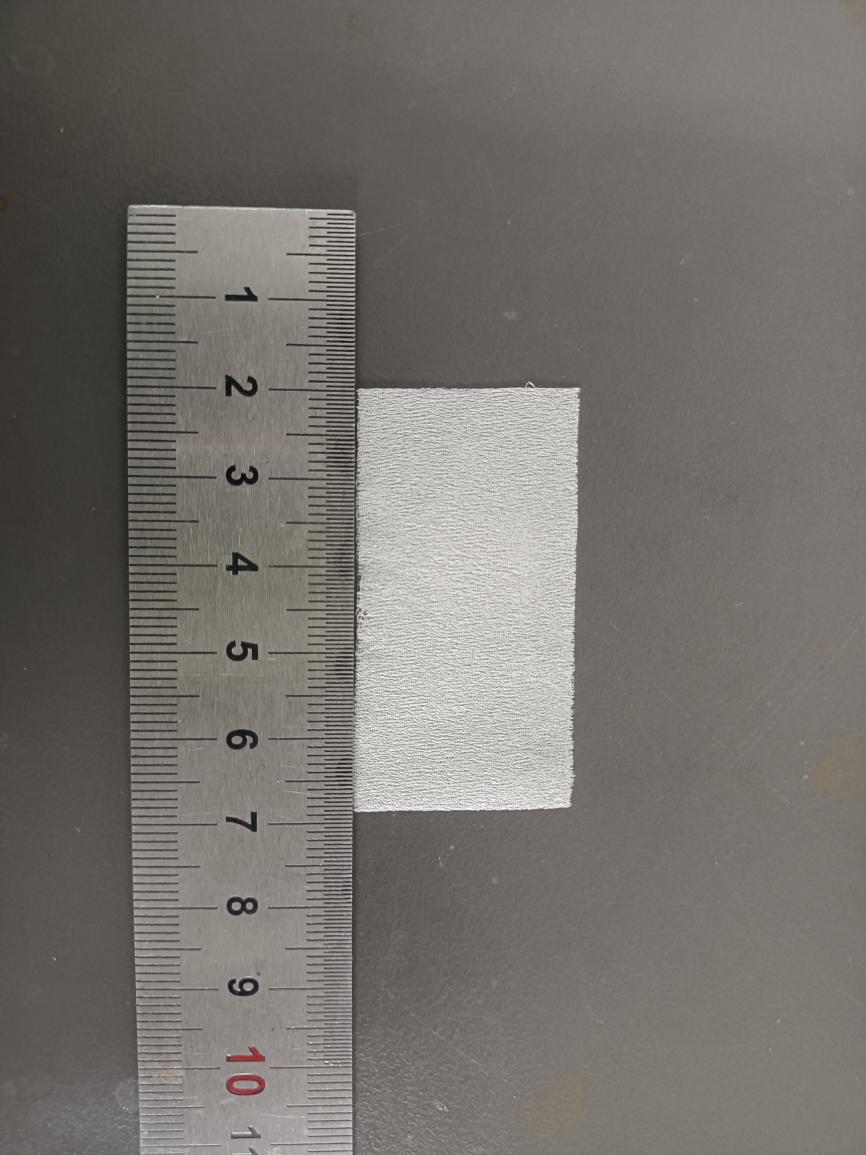
Fig.S52 Digital photograph of simulated rotten/pulverized silk fabric treated with 8% PEI + 2.5% PPEGDE + 1% IPP


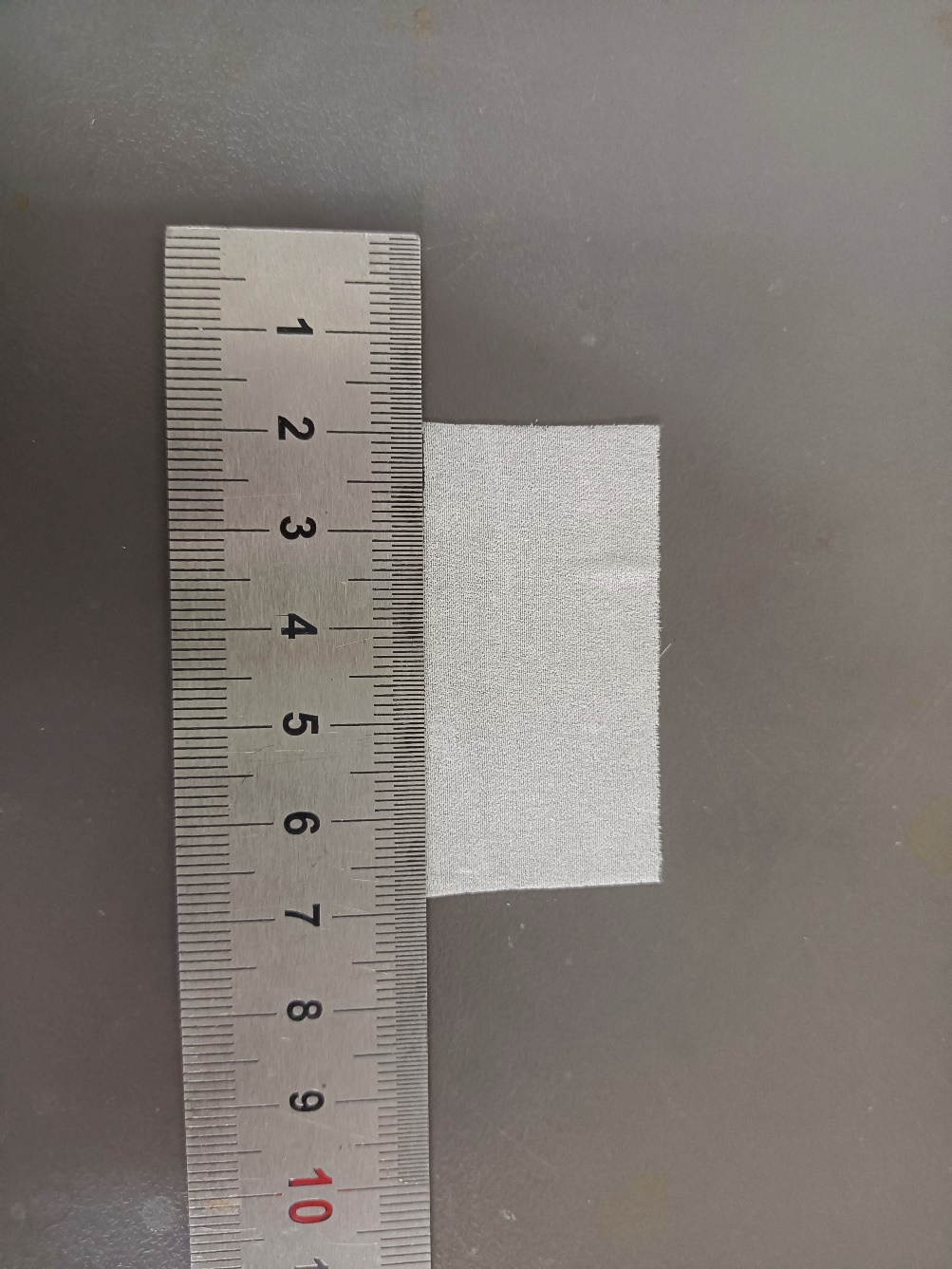
Fig.S53 Digital photograph of simulated rotten/pulverized silk fabric treated with 8% PEI + 3% PPEGDE + 1% IPP


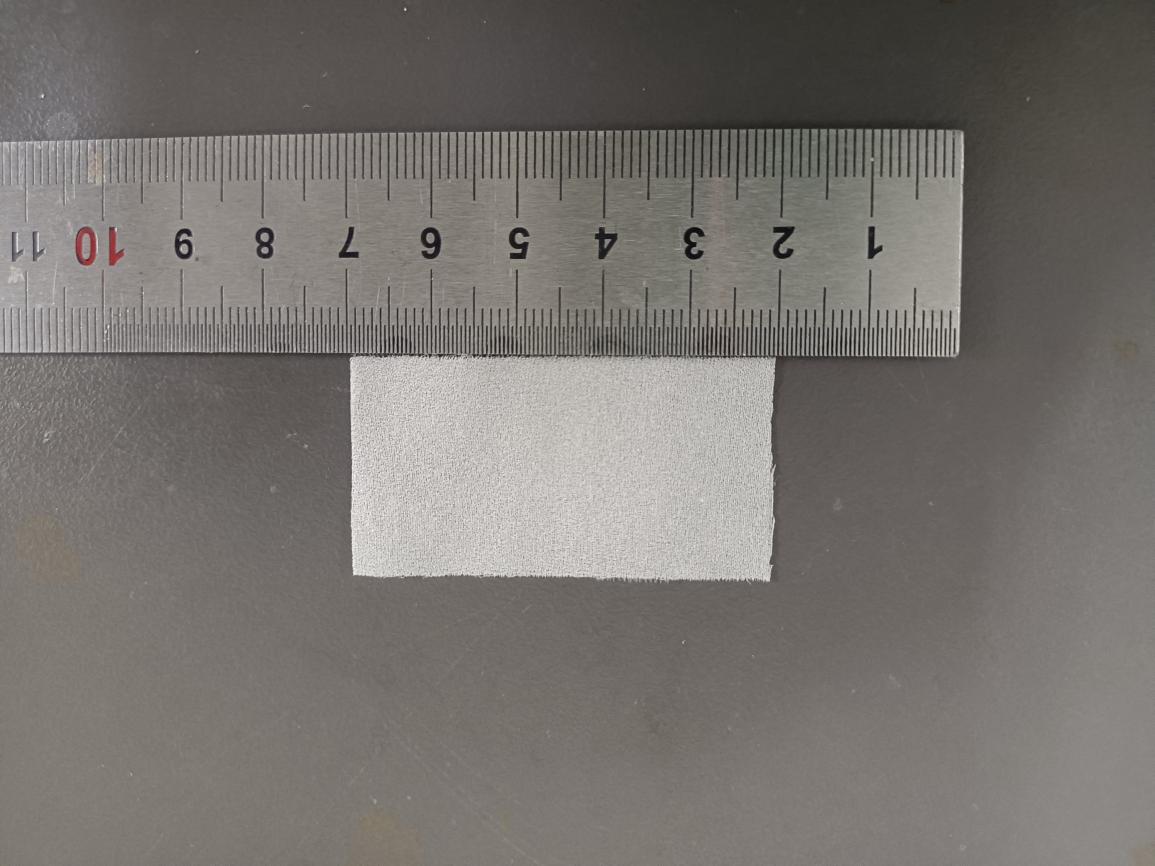


Fig.S54 Digital photograph of simulated rotten/pulverized silk fabric treated with 8% PEI + 3.5% PPEGDE + 1% IPP


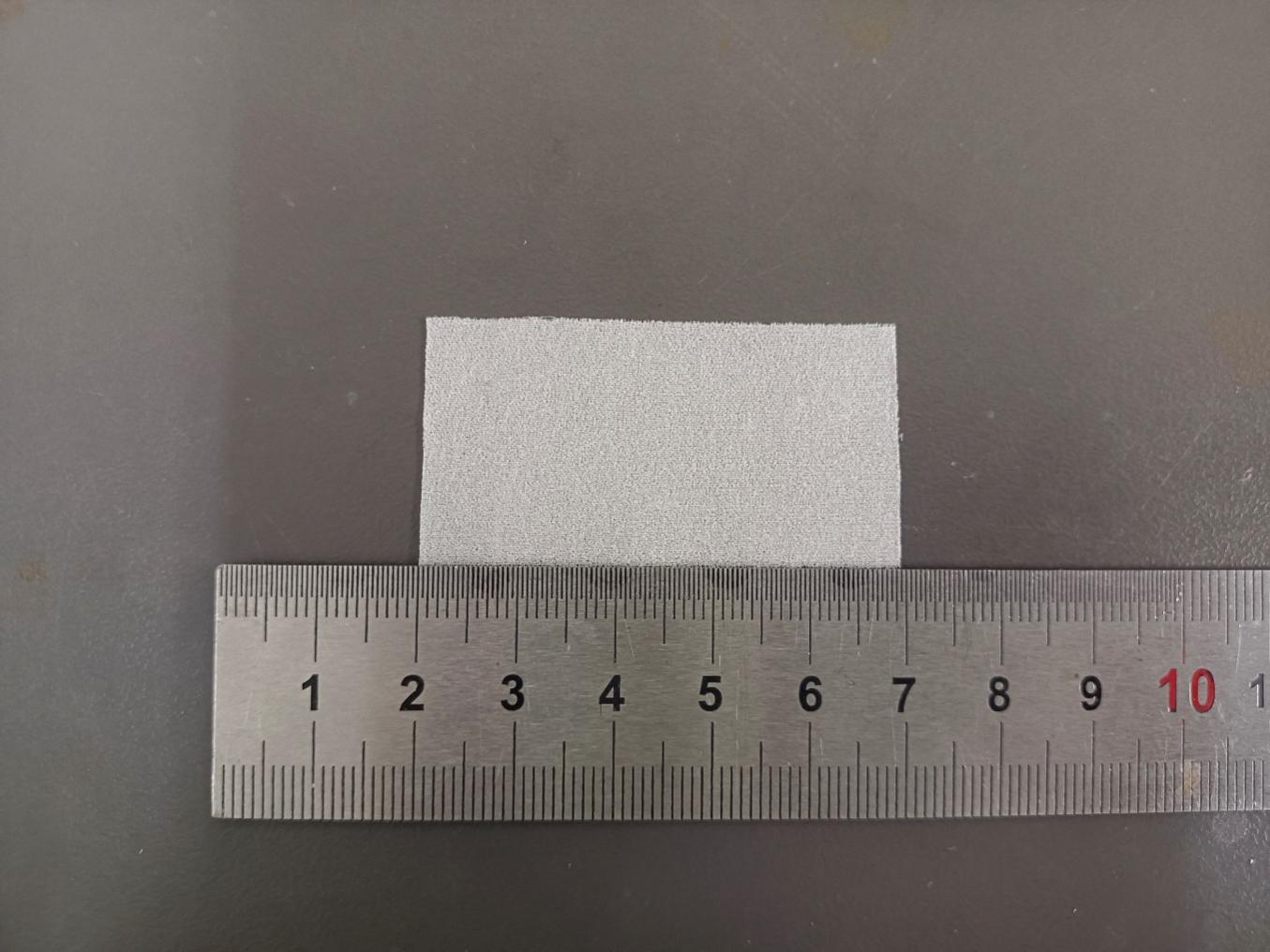


Fig.S55 Digital photograph of simulated rotten/pulverized silk fabric treated with 8% PEI + 4% PPEGDE + 1% IPP


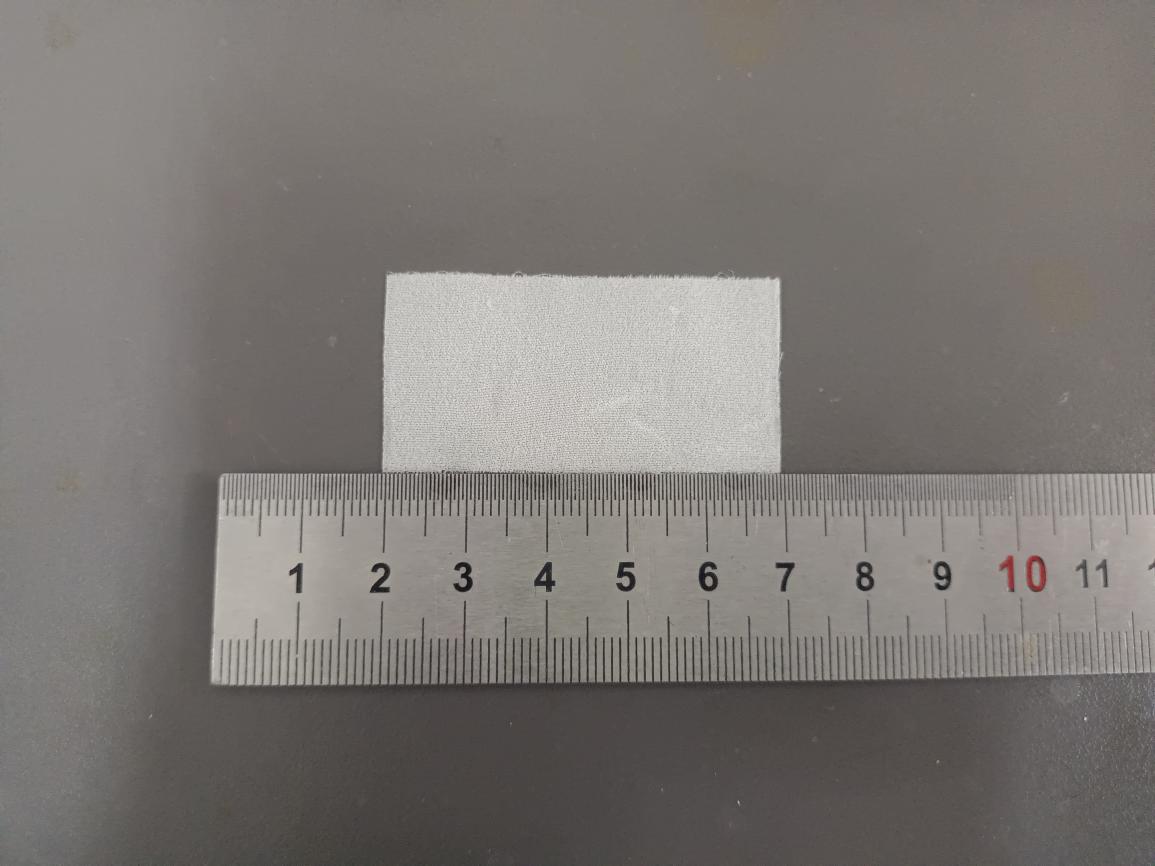


Fig.S56 Digital photograph of simulated rotten/pulverized silk fabric treated with 8% PEI + 4.5% PPEGDE + 1% IPP


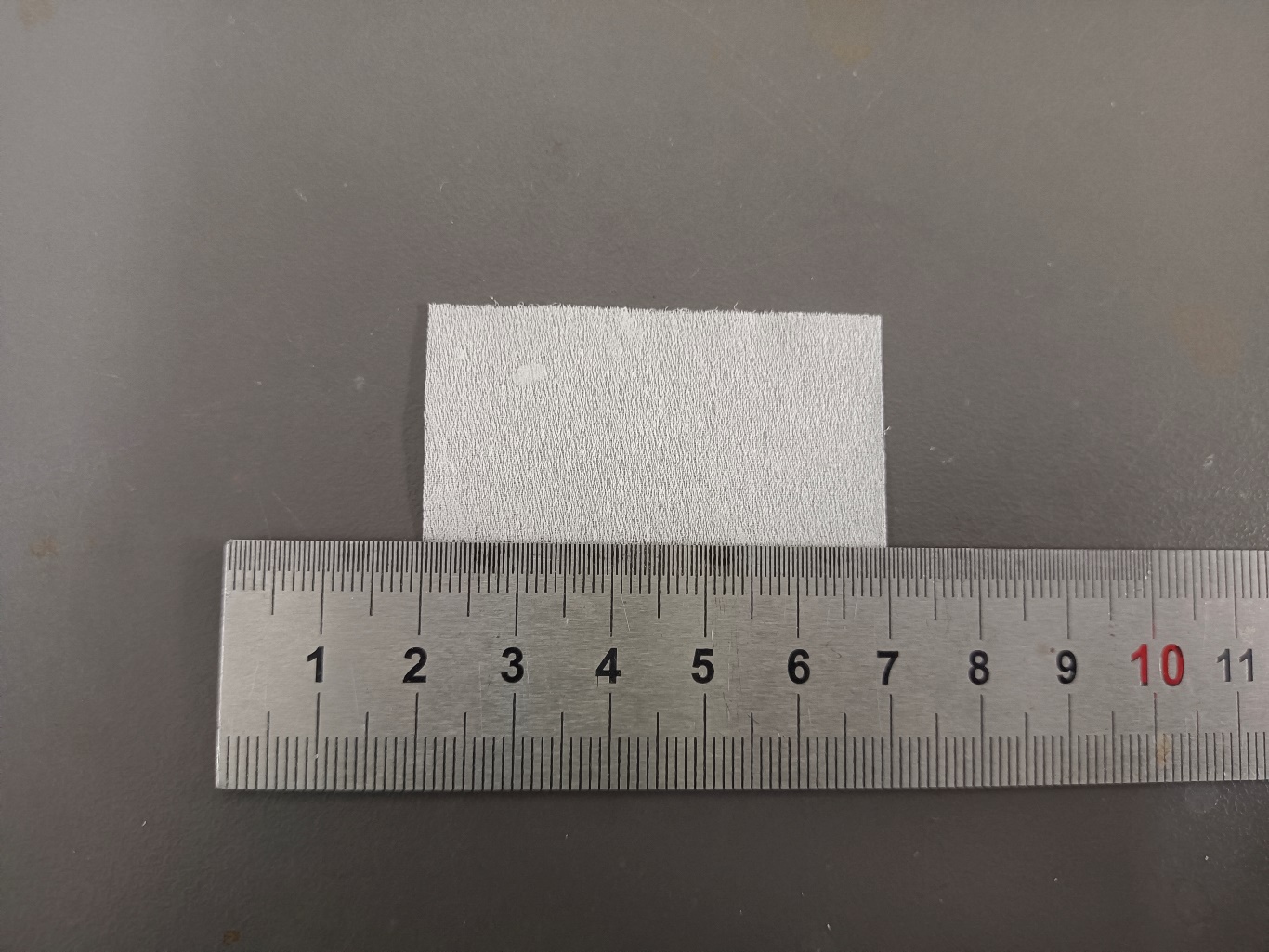


Fig.S57 Digital photograph of simulated rotten/pulverized silk fabric treated with 8% PEI + 5% PPEGDE + 1% IPP


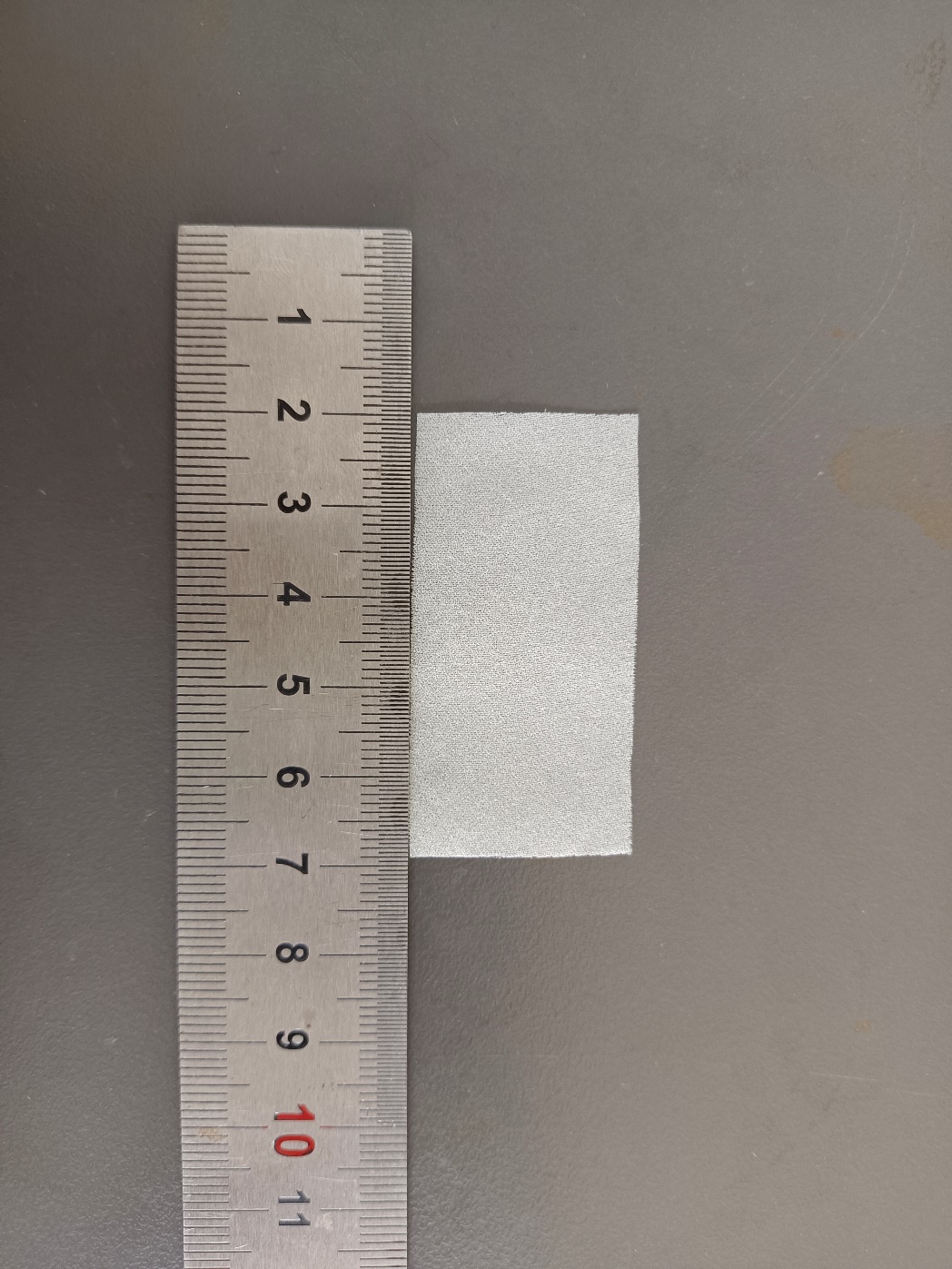
Fig.S58 Digital photograph of simulated rotten/pulverized silk fabric treated with 10% PEI + 0% PPEGDE + 1% IPP


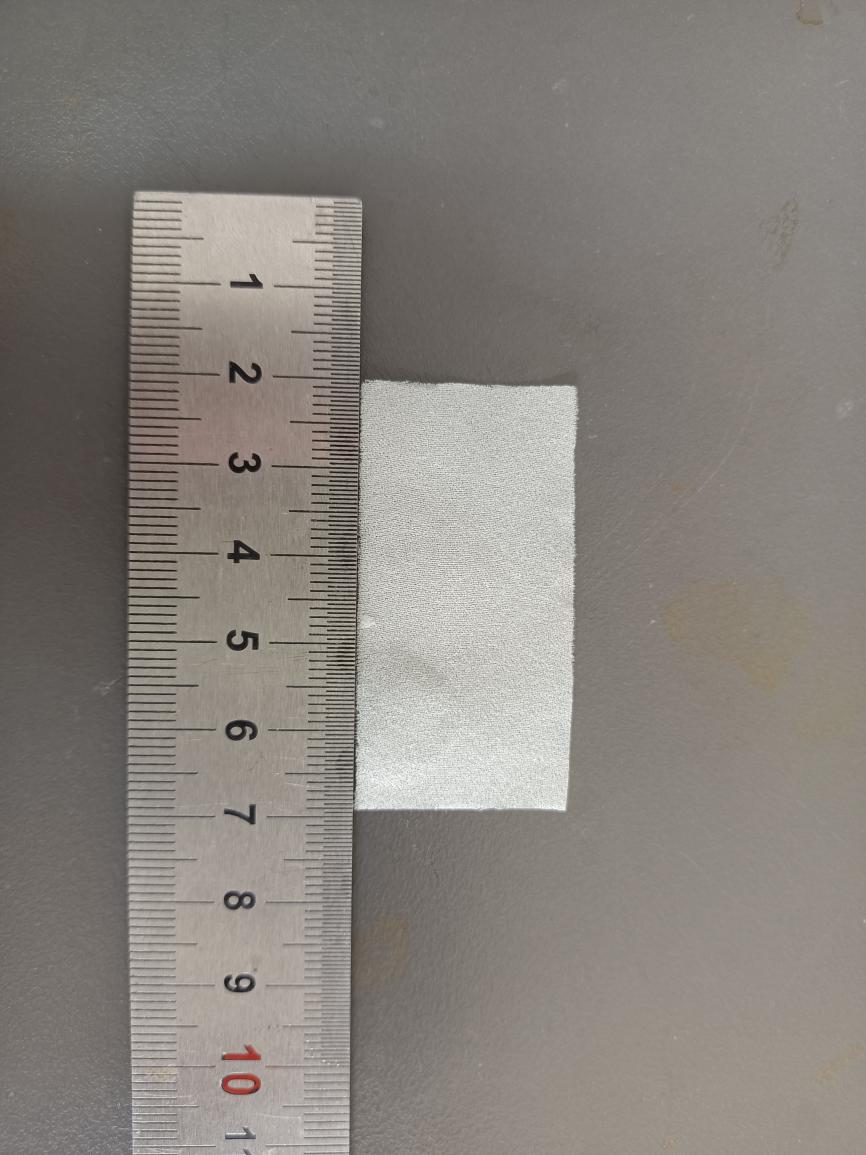
Fig.S59 Digital photograph of simulated rotten/pulverized silk fabric treated with 10% PEI + 0.5% PPEGDE + 1% IPP

Fig.S60 Digital photograph of simulated rotten/pulverized silk fabric treated with
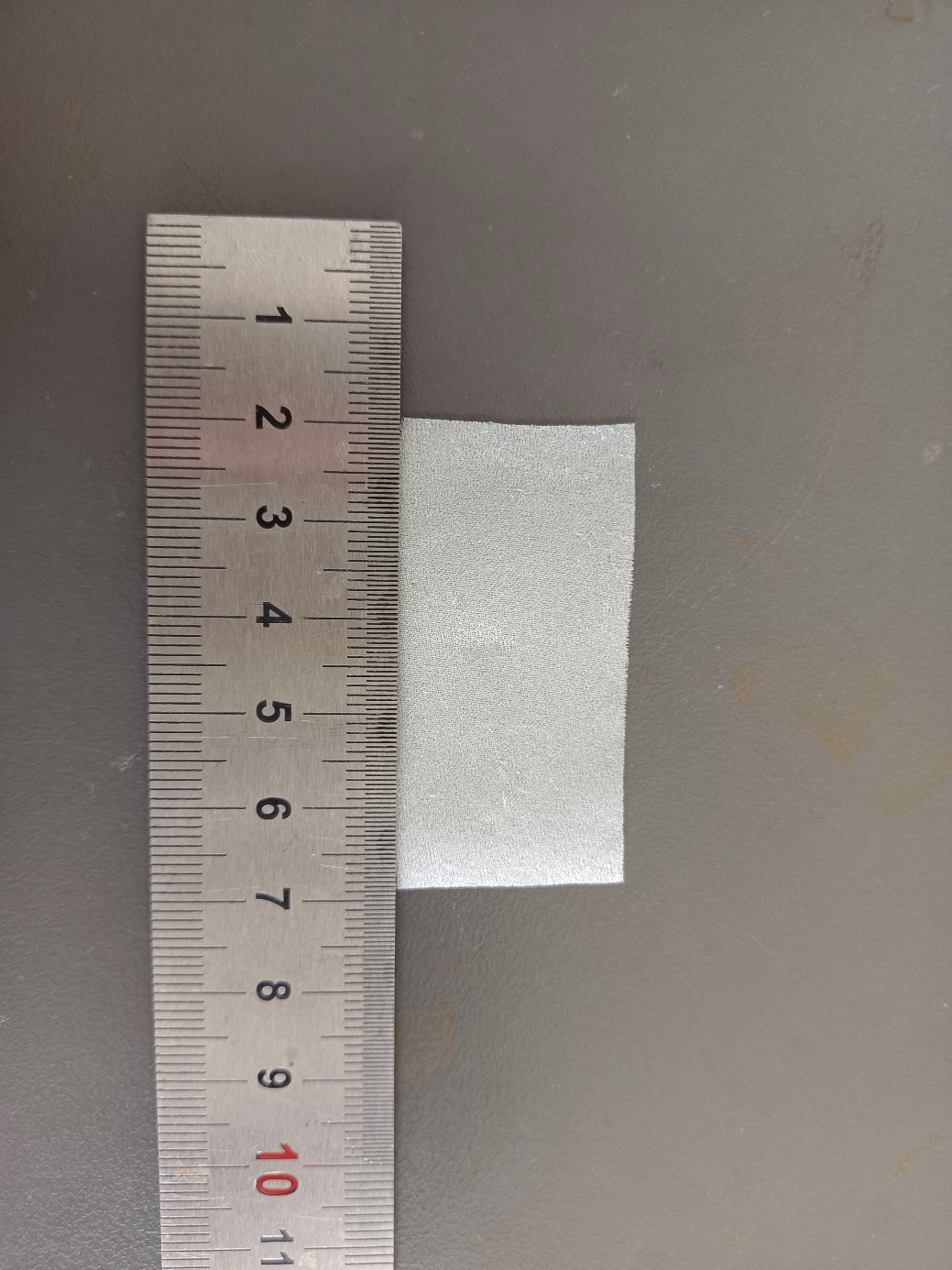
10% PEI + 1% PPEGDE + 1% IPP


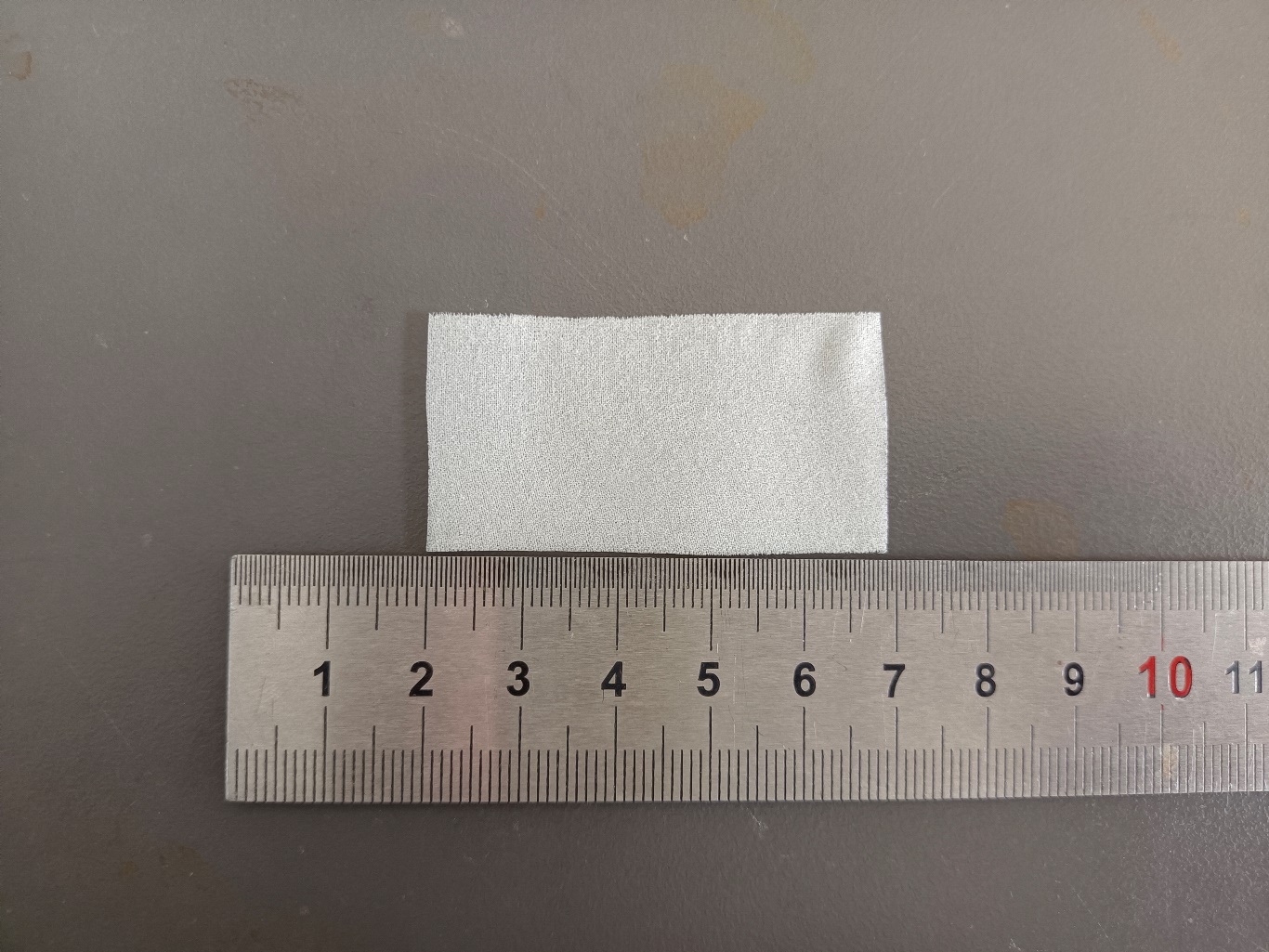


Fig.S61 Digital photograph of simulated rotten/pulverized silk fabric treated with 10% PEI + 1.5% PPEGDE + 1% IPP


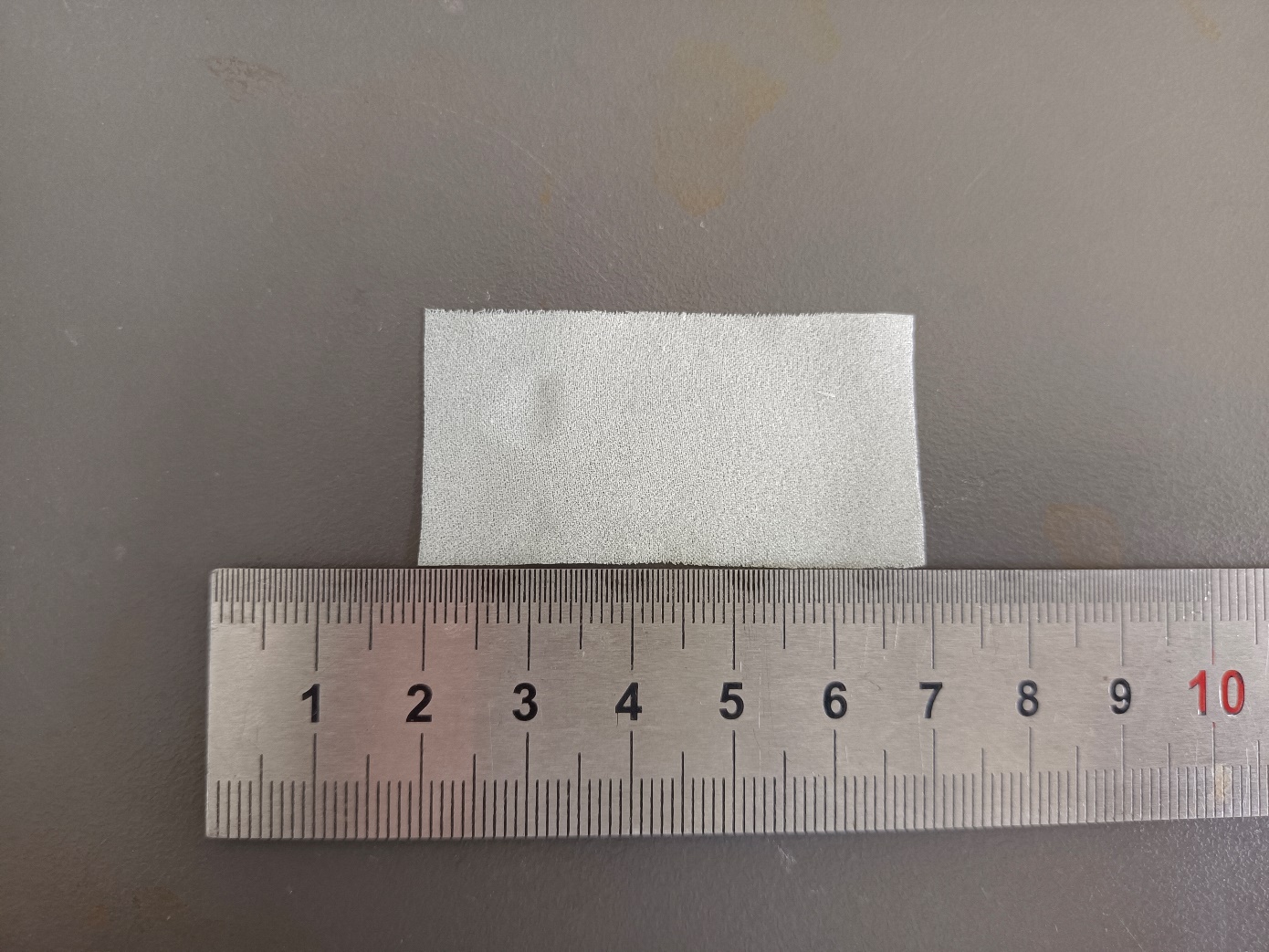


Fig.S62 Digital photograph of simulated rotten/pulverized silk fabric treated with 10% PEI + 2% PPEGDE + 1% IPP


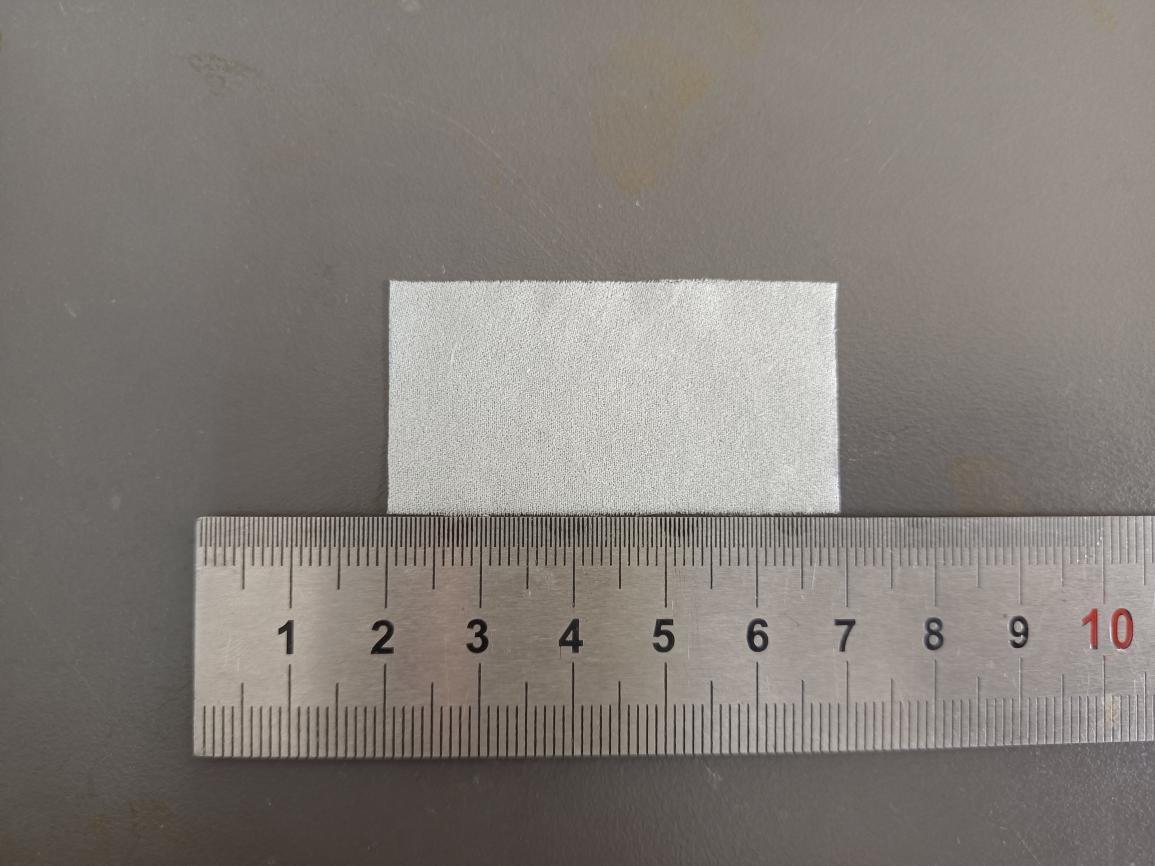


Fig.S63 Digital photograph of simulated rotten/pulverized silk fabric treated with 10% PEI + 2.5% PPEGDE + 1% IPP


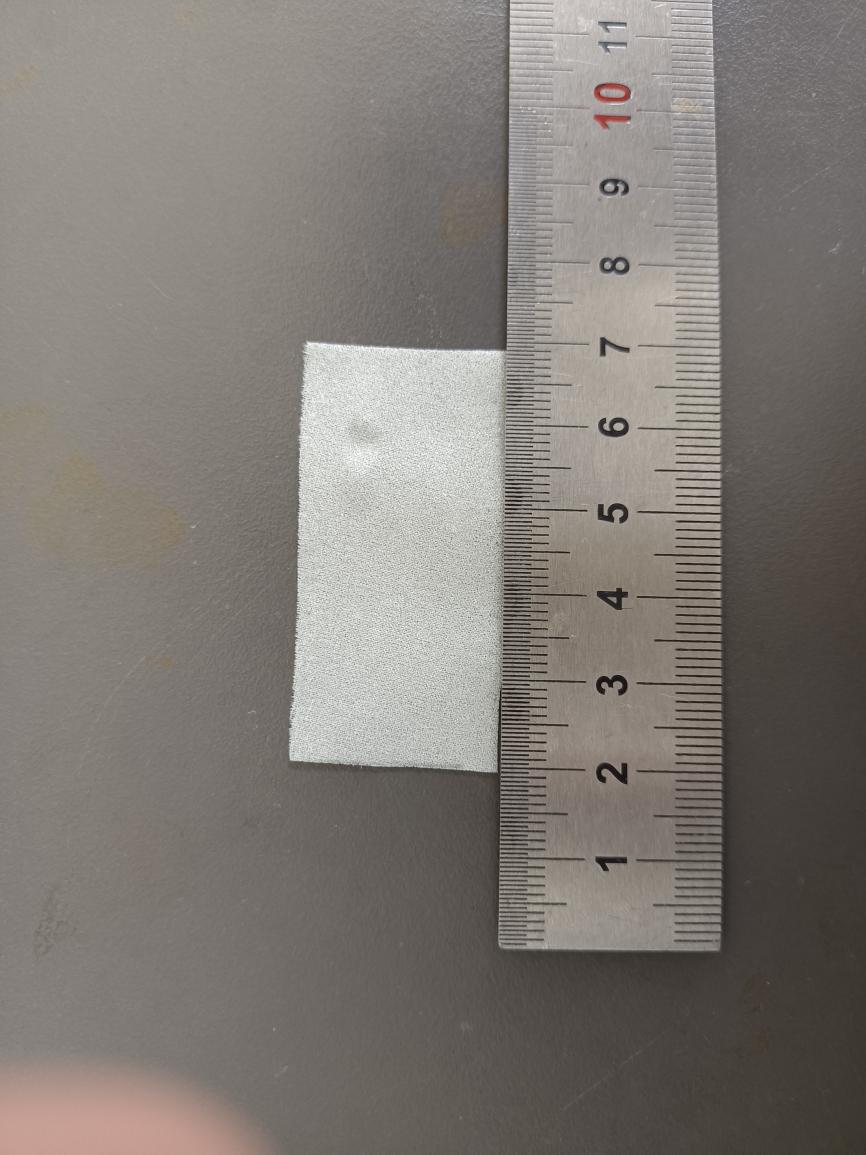
Fig.S64 Digital photograph of simulated rotten/pulverized silk fabric treated with 10% PEI + 3% PPEGDE + 1% IPP


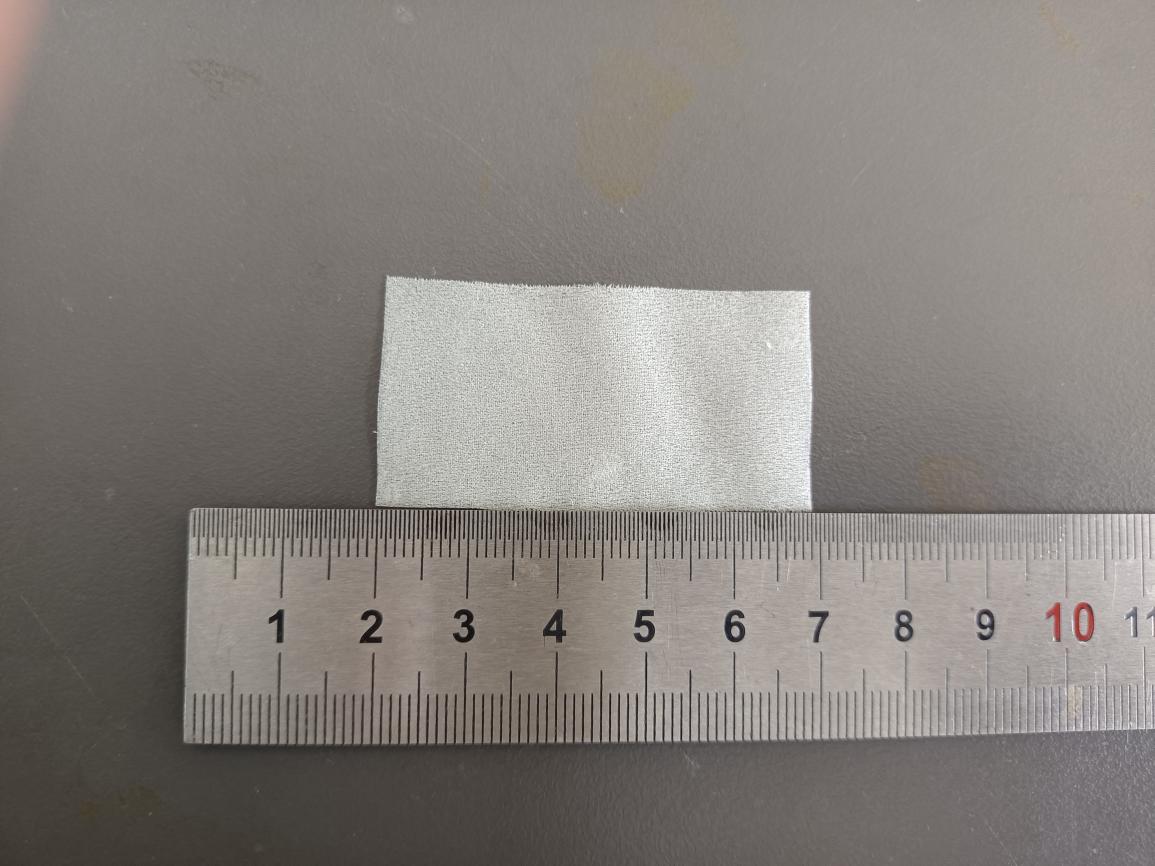


Fig.S65 Digital photograph of simulated rotten/pulverized silk fabric treated with 10% PEI + 3.5% PPEGDE + 1% IPP


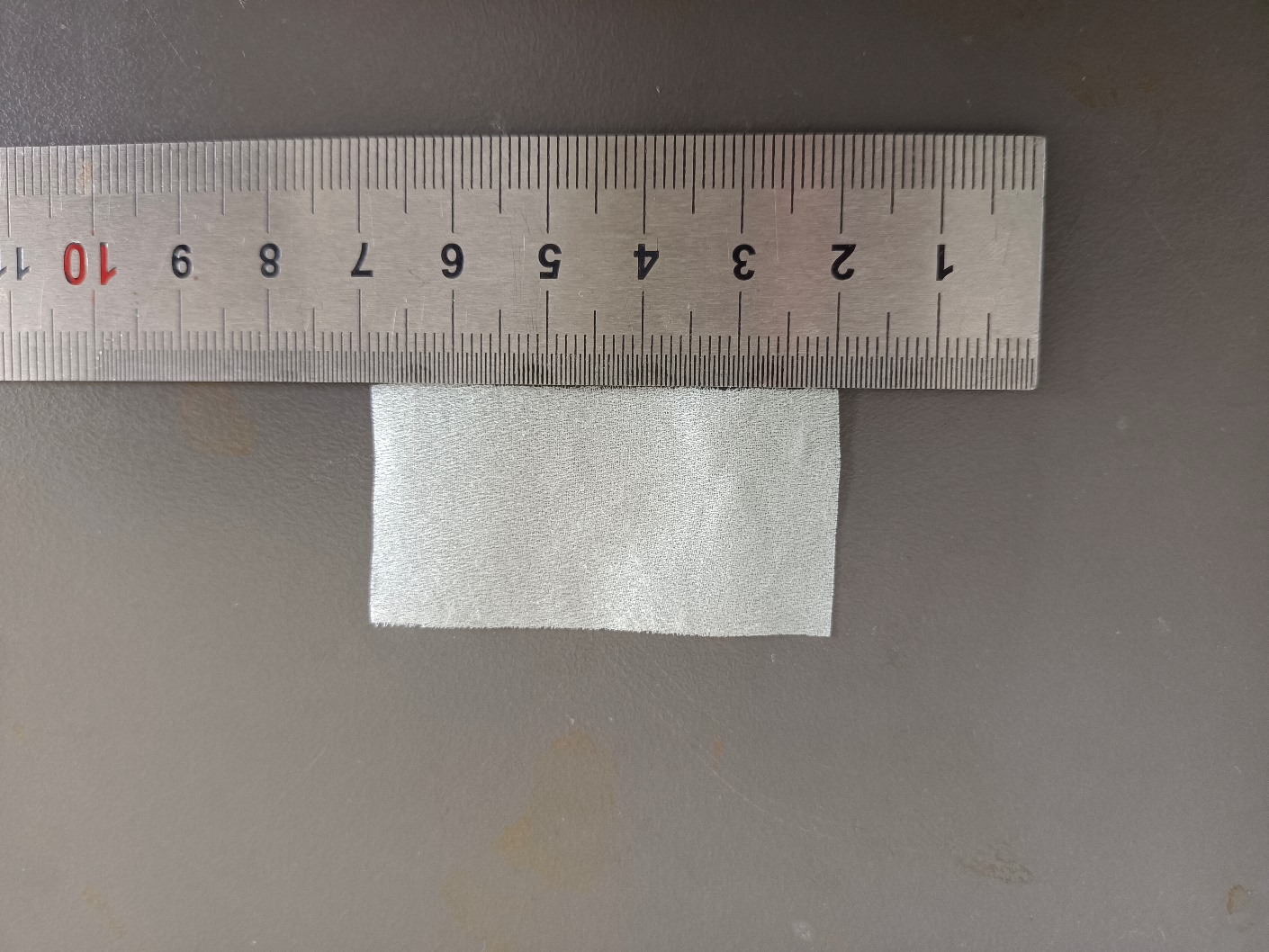


Fig.S66 Digital photograph of simulated rotten/pulverized silk fabric treated with 10% PEI + 4% PPEGDE + 1% IPP


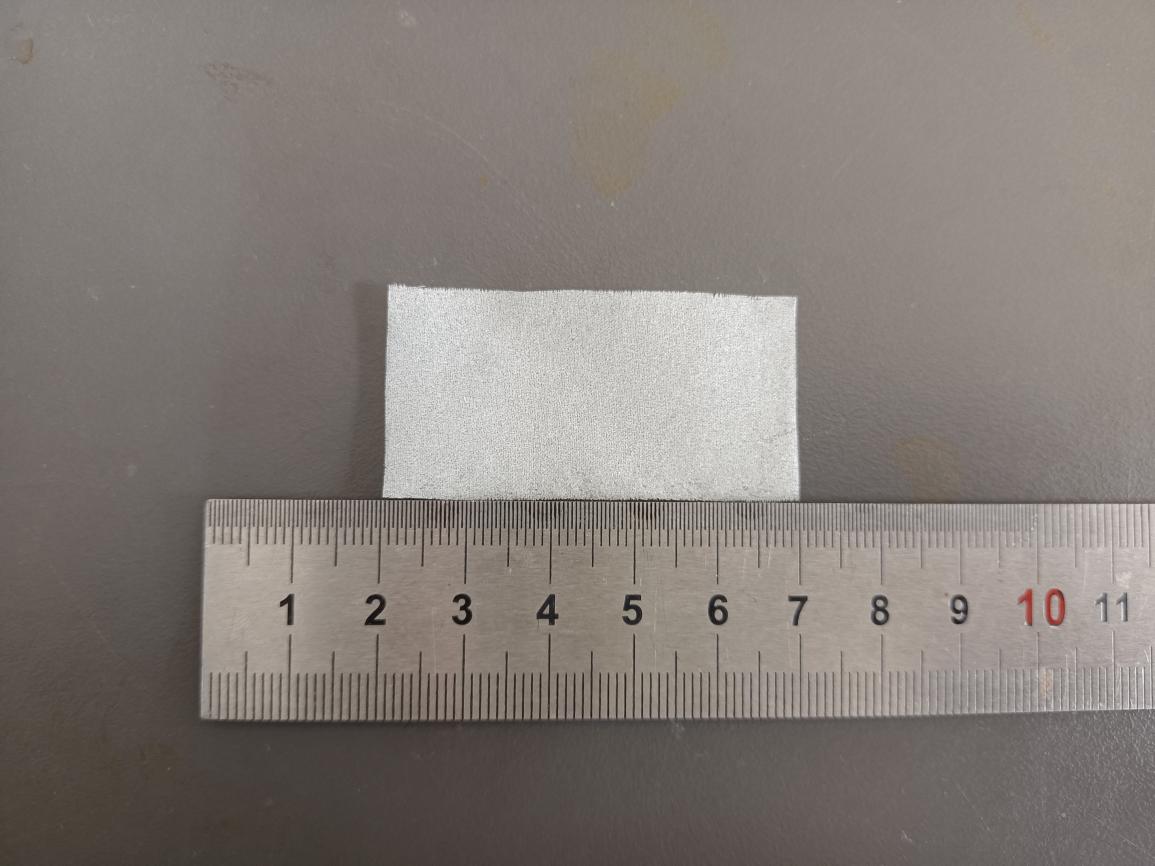


Fig.S67 Digital photograph of simulated rotten/pulverized silk fabric treated with 10% PEI + 4.5% PPEGDE + 1% IPP


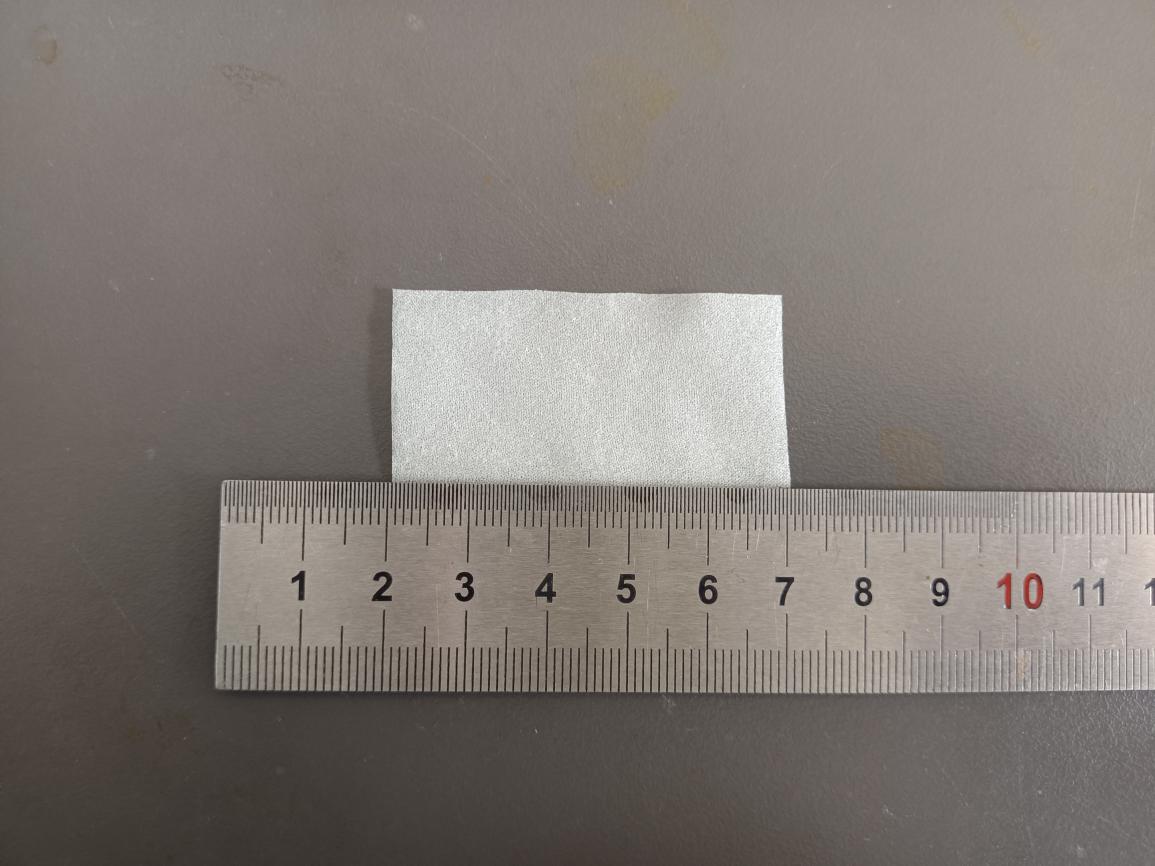


Fig.S68 Digital photograph of simulated rotten/pulverized silk fabric treated with 10% PEI + 5% PPEGDE + 1% IPP


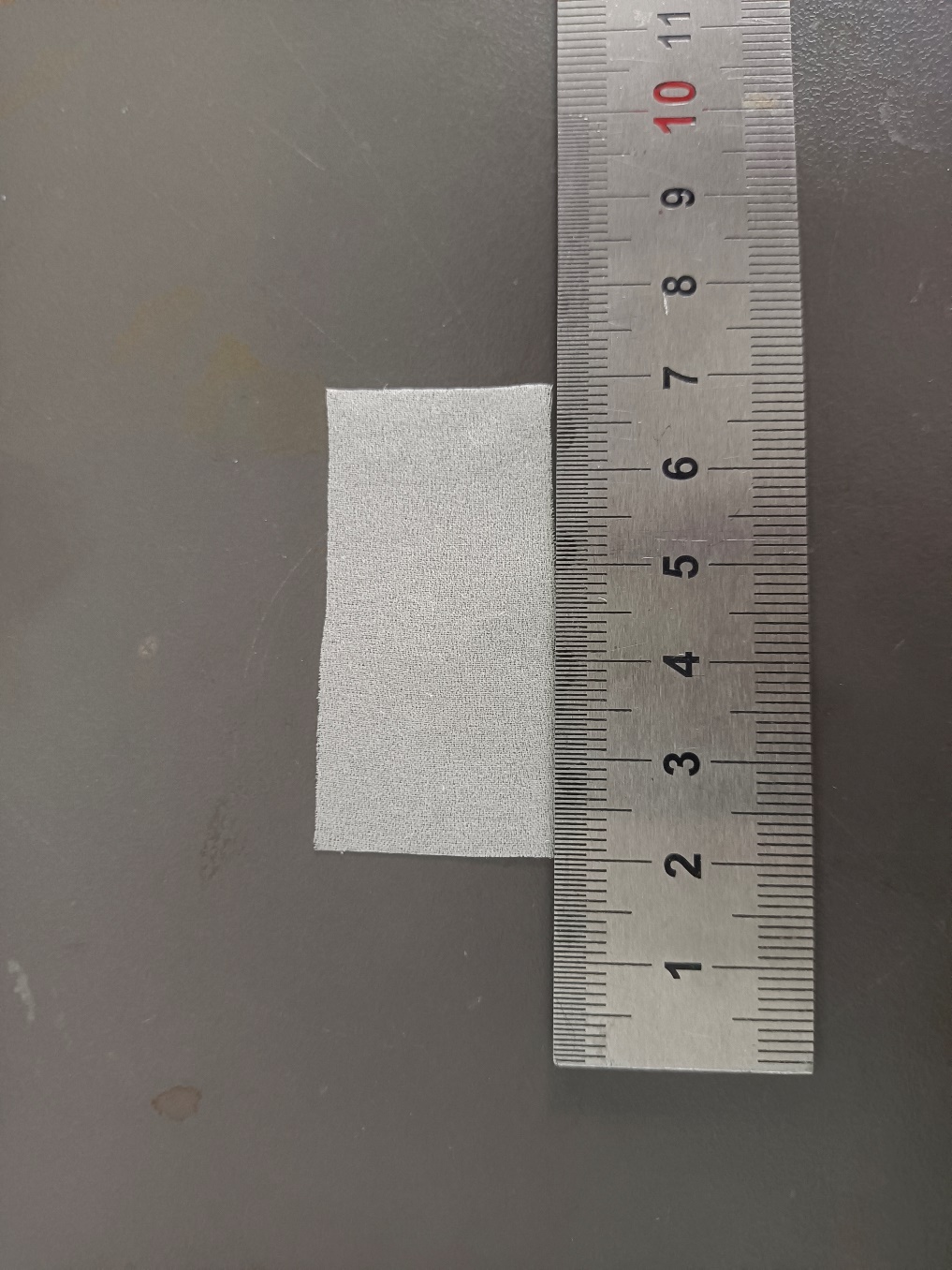
Fig.S69 Digital photograph of simulated rotten/pulverized silk fabric treated with 12% PEI + 0% PPEGDE + 1% IPP


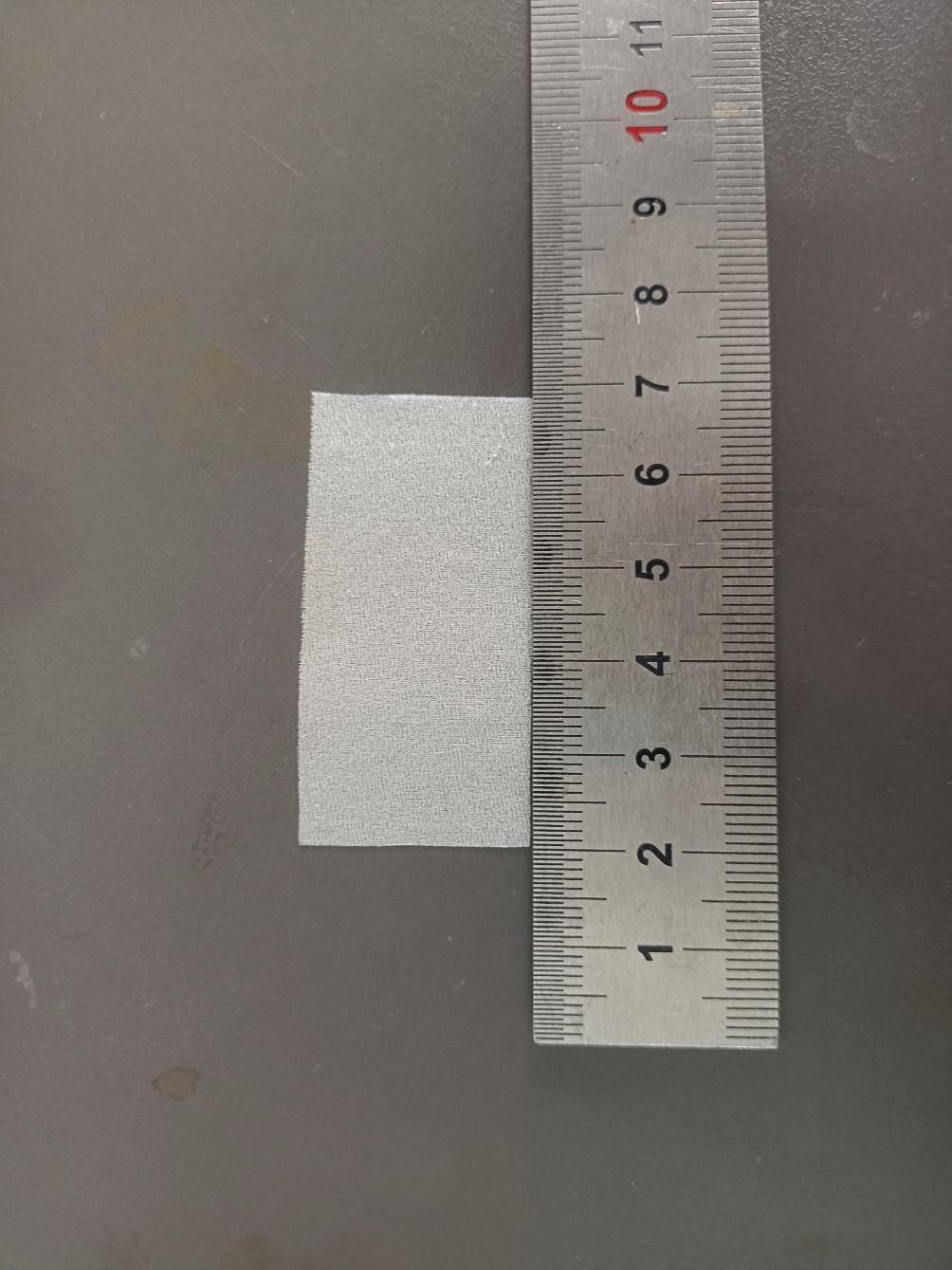
Fig.S70 Digital photograph of simulated rotten/pulverized silk fabric treated with 12% PEI + 0.5% PPEGDE + 1% IPP


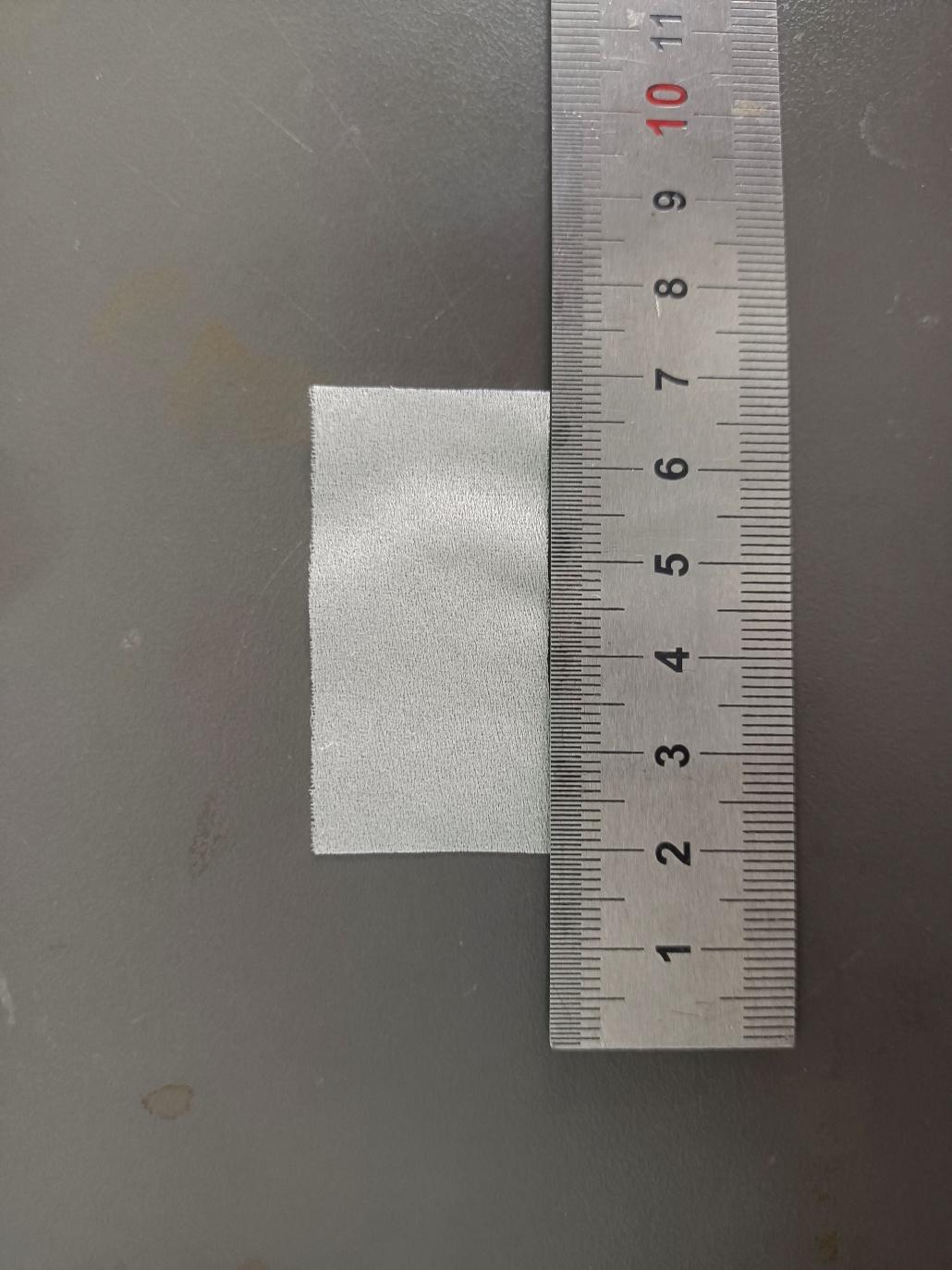
Fig.S71 Digital photograph of simulated rotten/pulverized silk fabric treated with 12% PEI + 1% PPEGDE + 1% IPP


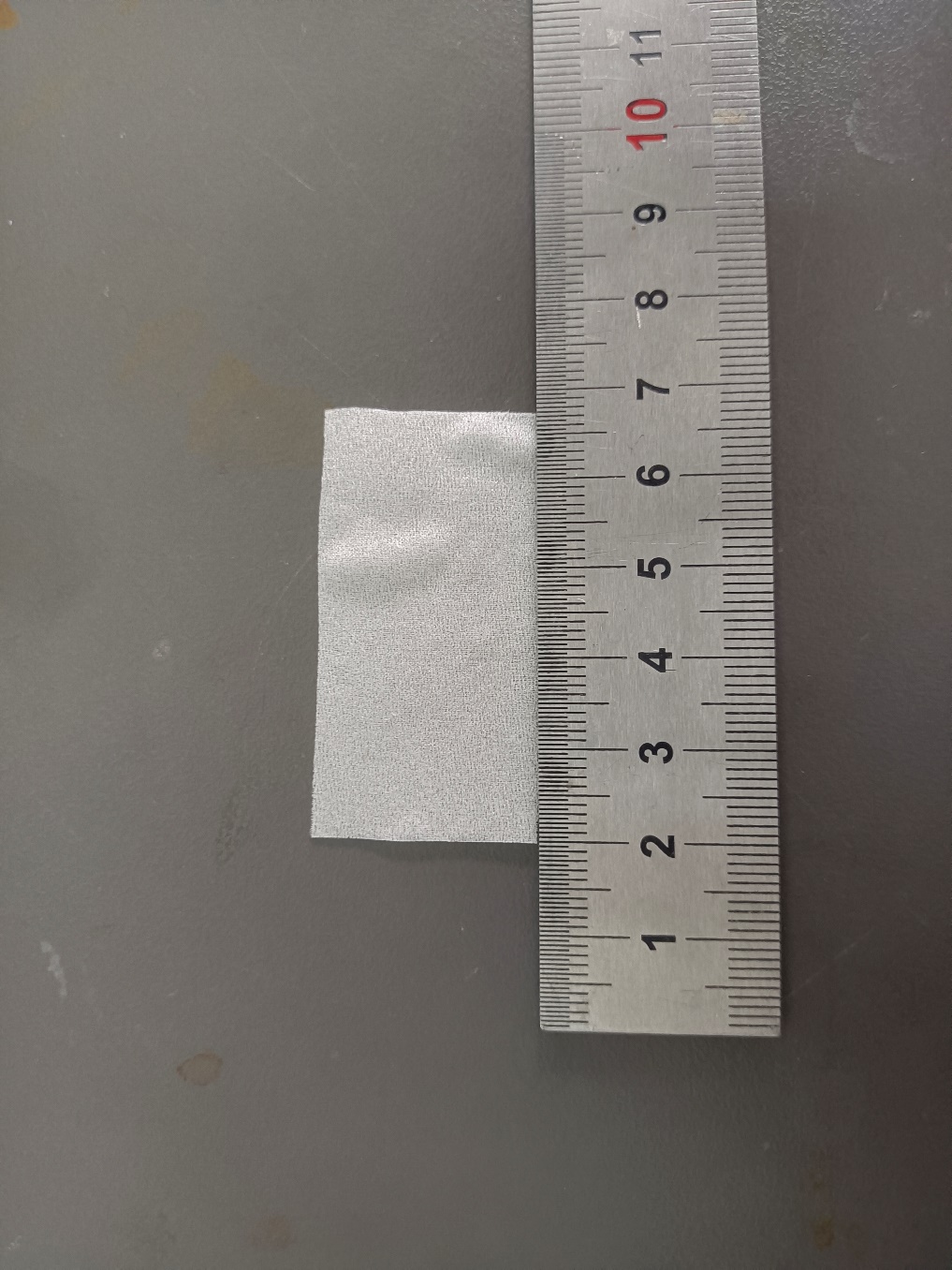
Fig.S72 Digital photograph of simulated rotten/pulverized silk fabric treated with 12% PEI + 1.5% PPEGDE + 1% IPP


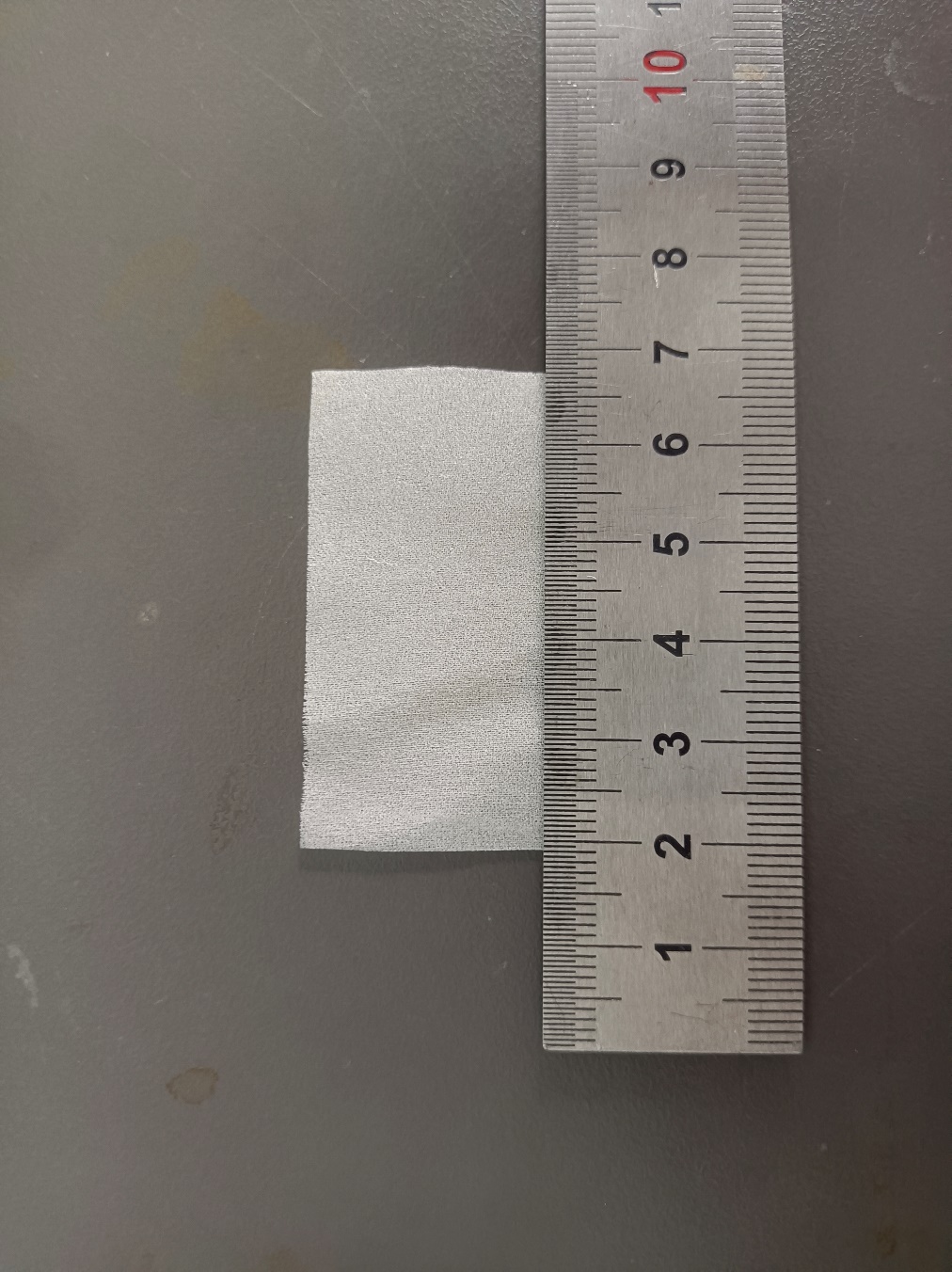
Fig.S73 Digital photograph of simulated rotten/pulverized silk fabric treated with 12% PEI + 2% PPEGDE + 1% IPP


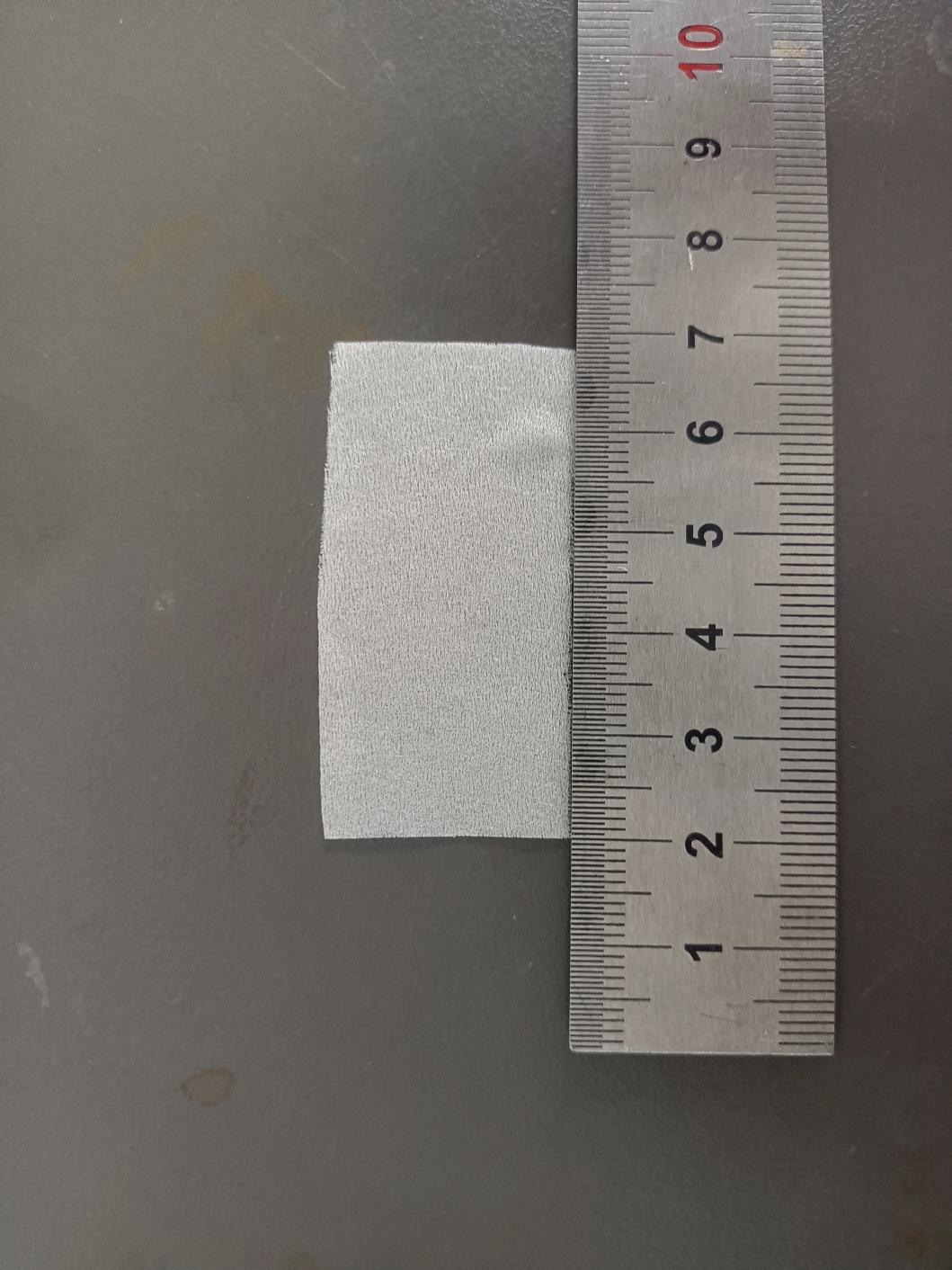
Fig.S74 Digital photograph of simulated rotten/pulverized silk fabric treated with 12% PEI + 2.5% PPEGDE + 1% IPP


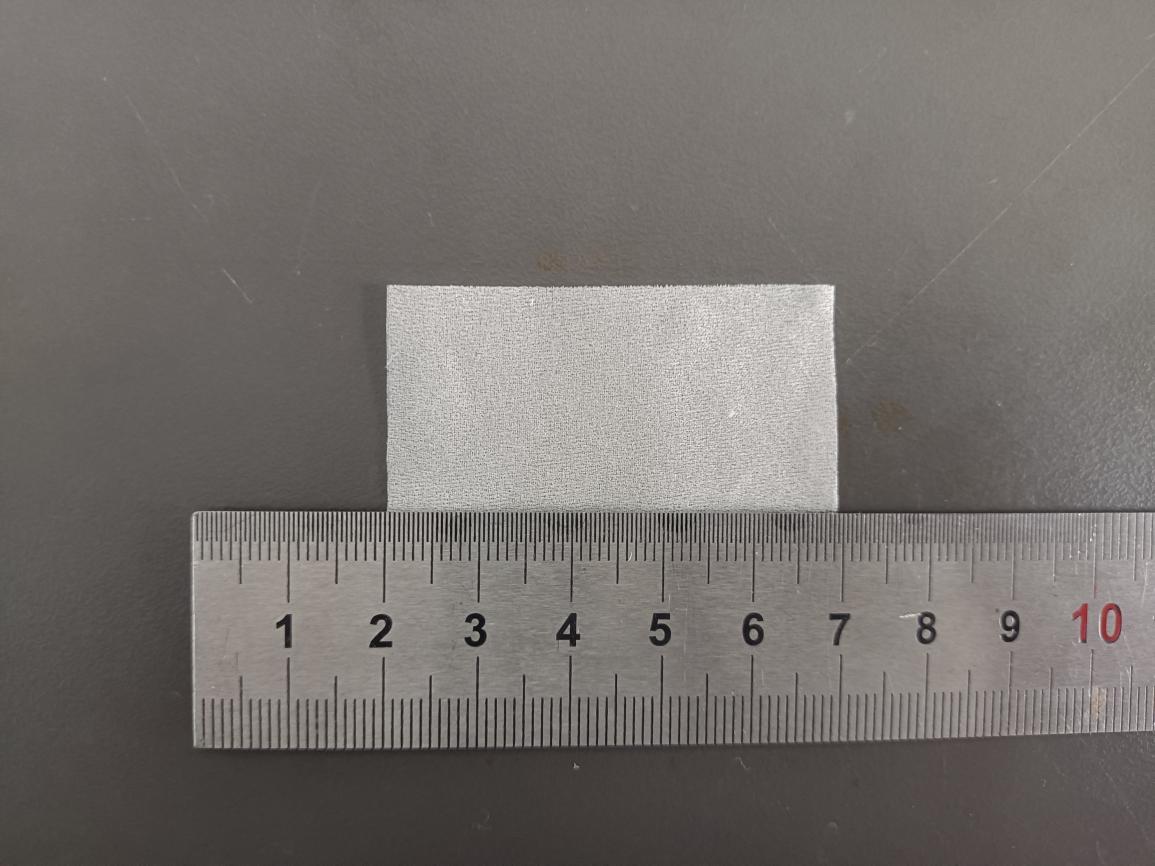


Fig.S75 Digital photograph of simulated rotten/pulverized silk fabric treated with 12% PEI + 3% PPEGDE + 1% IPP


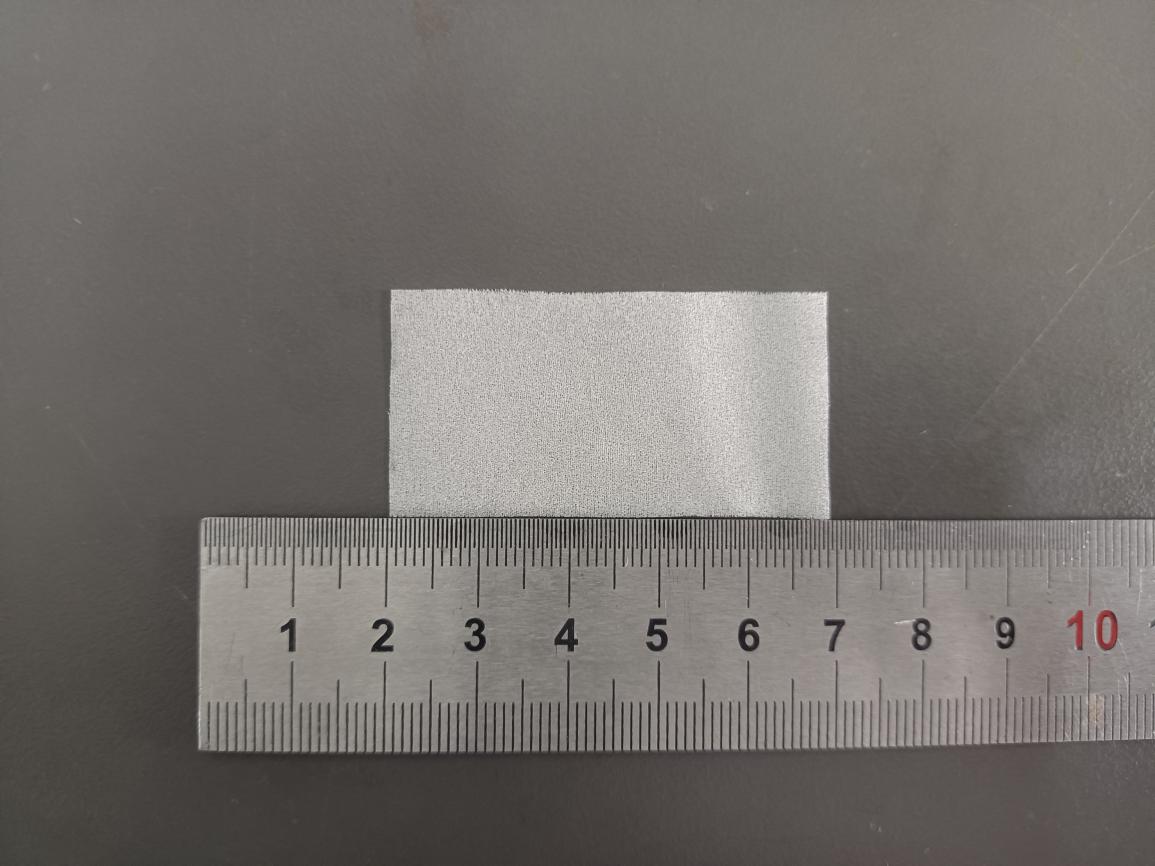


Fig.S76 Digital photograph of simulated rotten/pulverized silk fabric treated with 12% PEI + 3.5% PPEGDE + 1% IPP


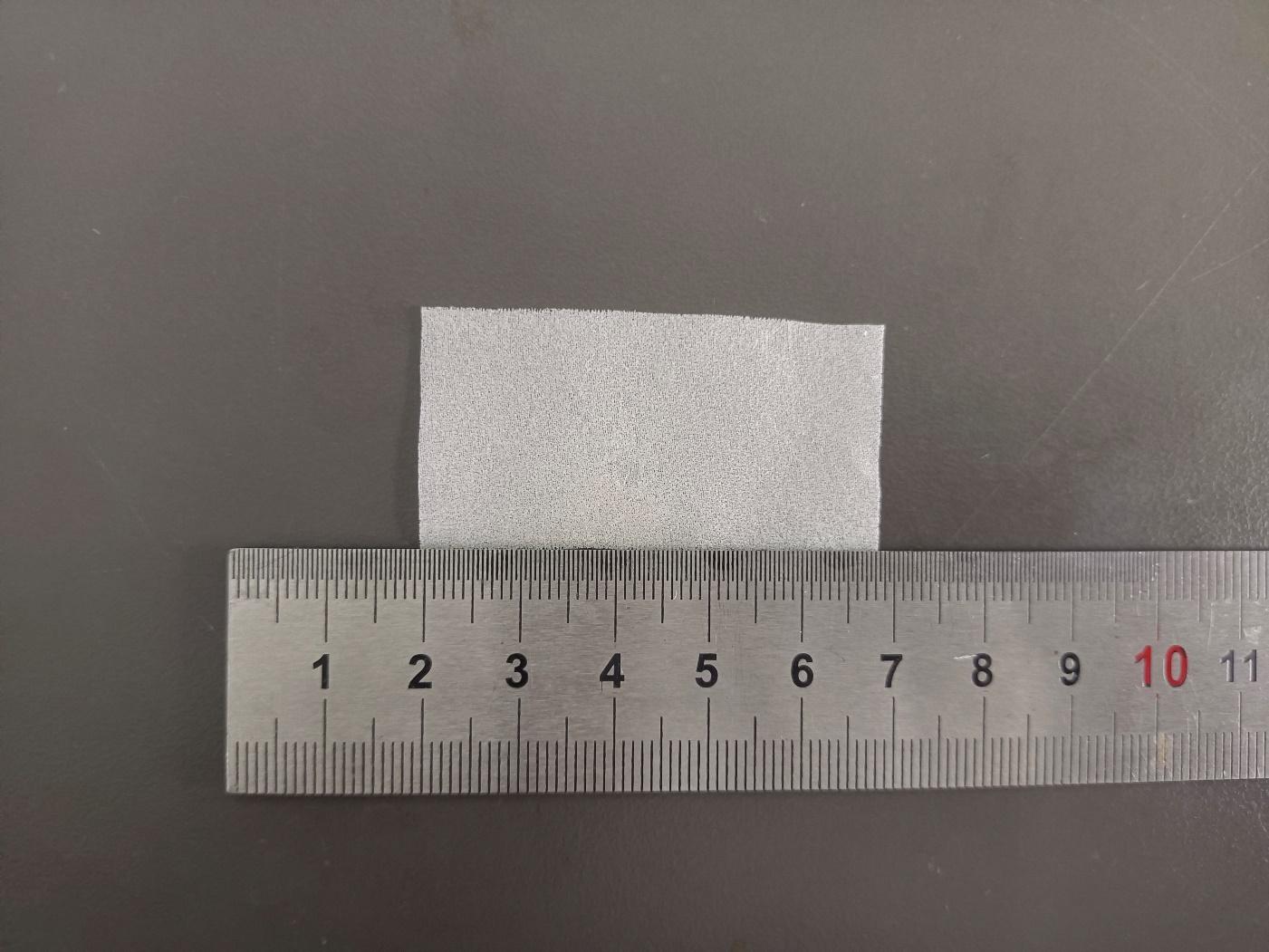


Fig.S77 Digital photograph of simulated rotten/pulverized silk fabric treated with 12% PEI + 4% PPEGDE + 1% IPP


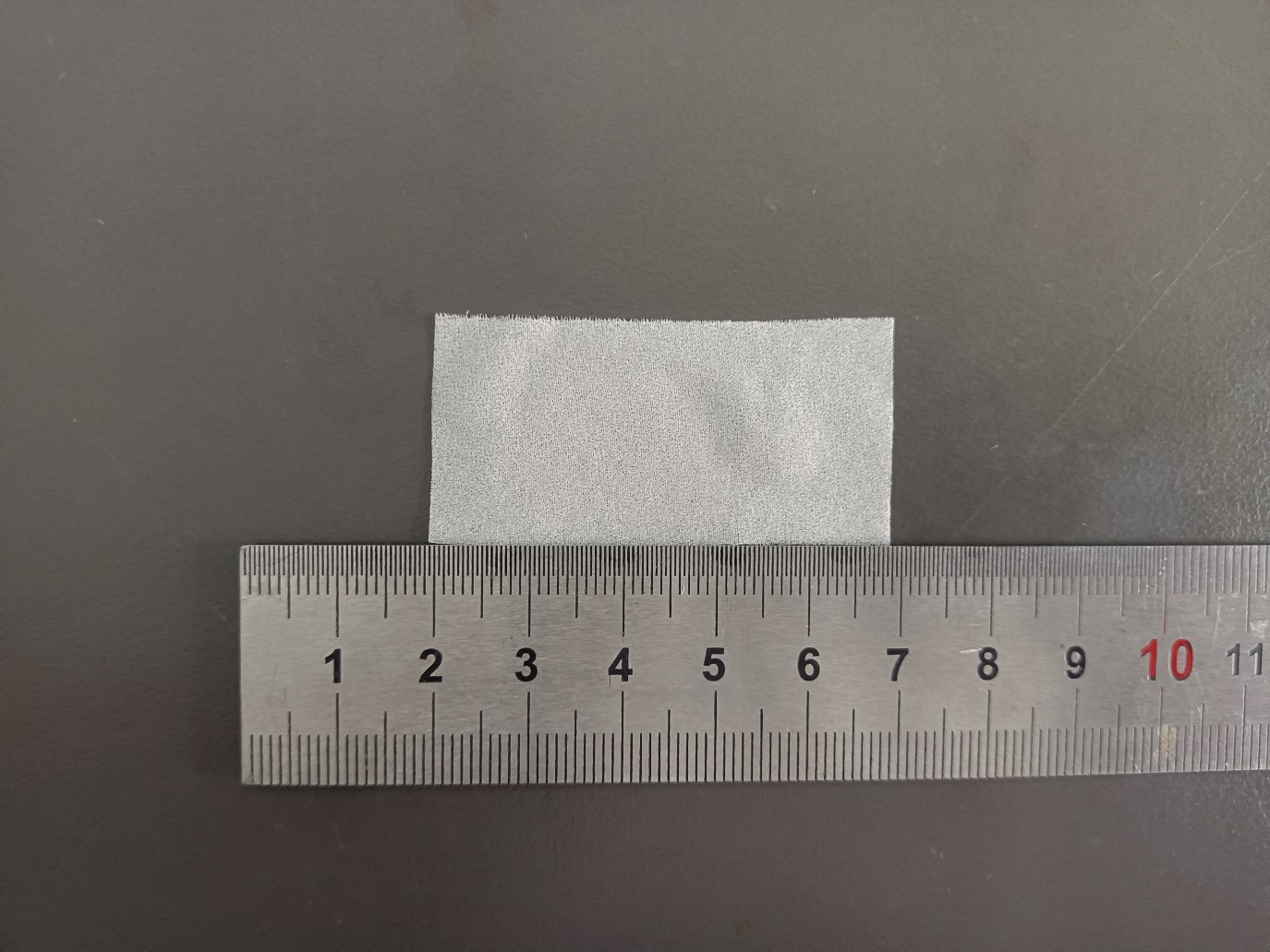


Fig.S78 Digital photograph of simulated rotten/pulverized silk fabric treated with 12% PEI + 4.5% PPEGDE + 1% IPP


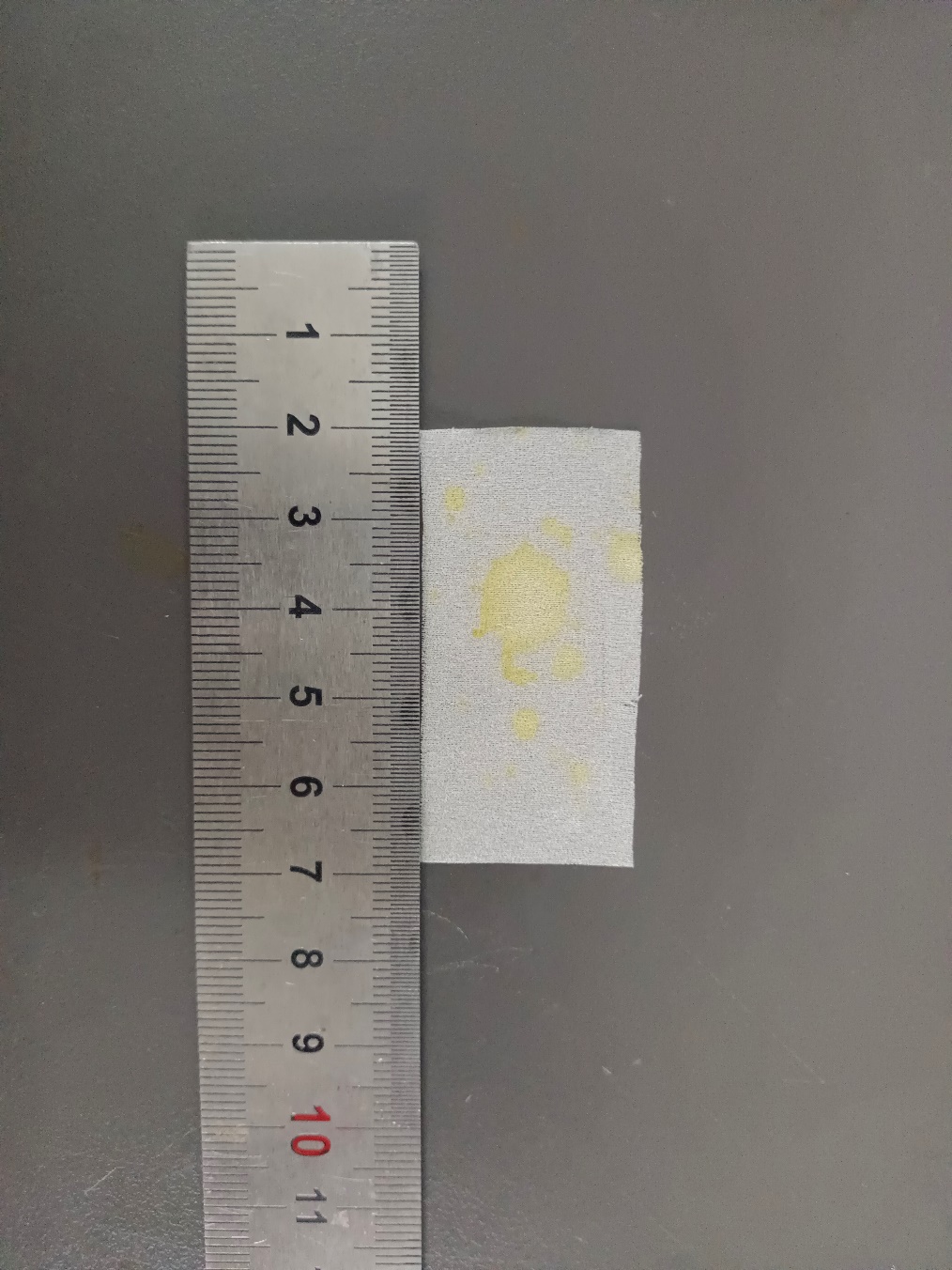
Fig.S79 Digital photograph of simulated rotten/pulverized silk fabric treated with 12% PEI + 5% PPEGDE + 1% IPP


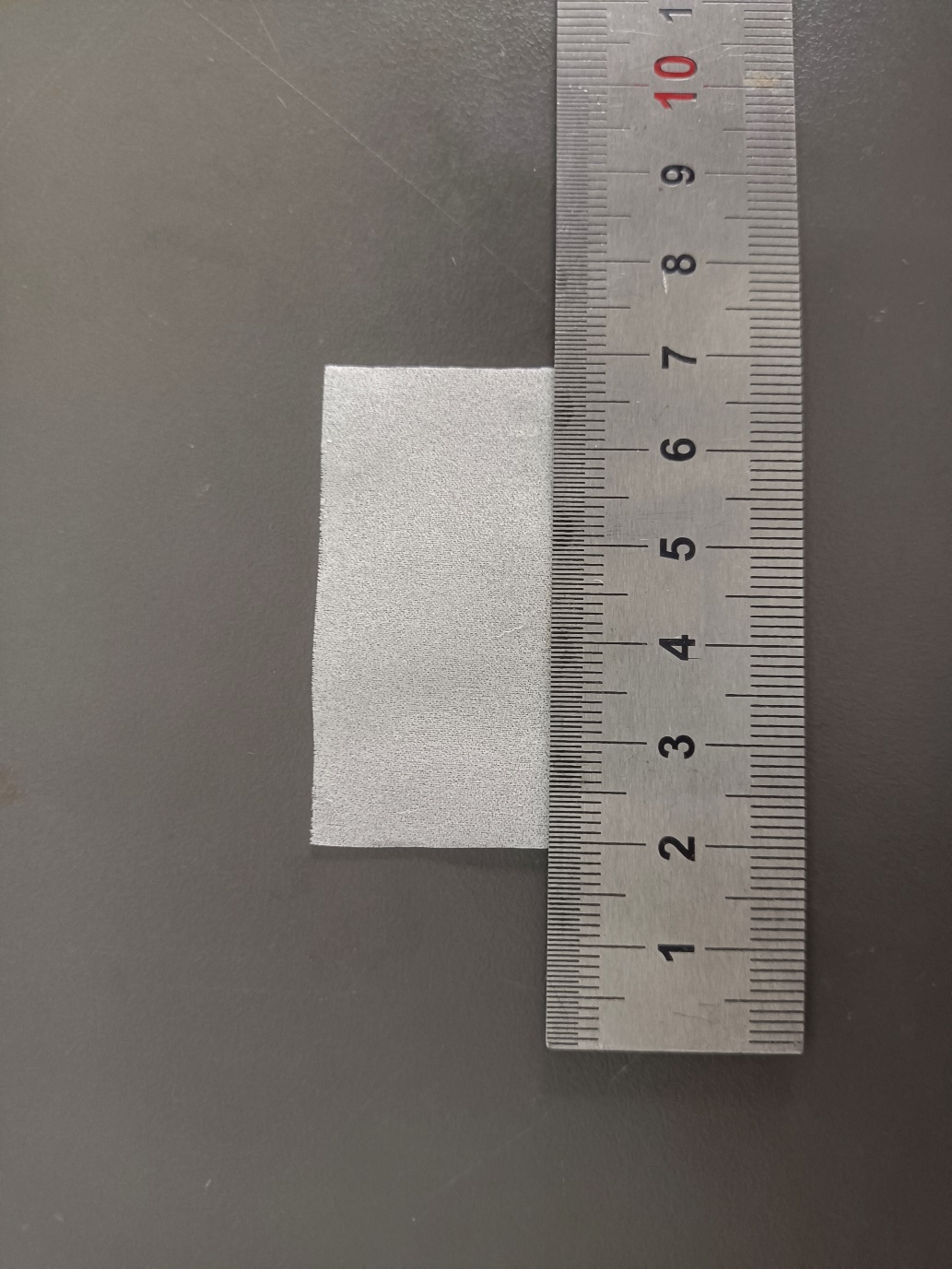
Fig.S80 Digital photograph of simulated rotten/pulverized silk fabric treated with 14% PEI + 0% PPEGDE + 1% IPP


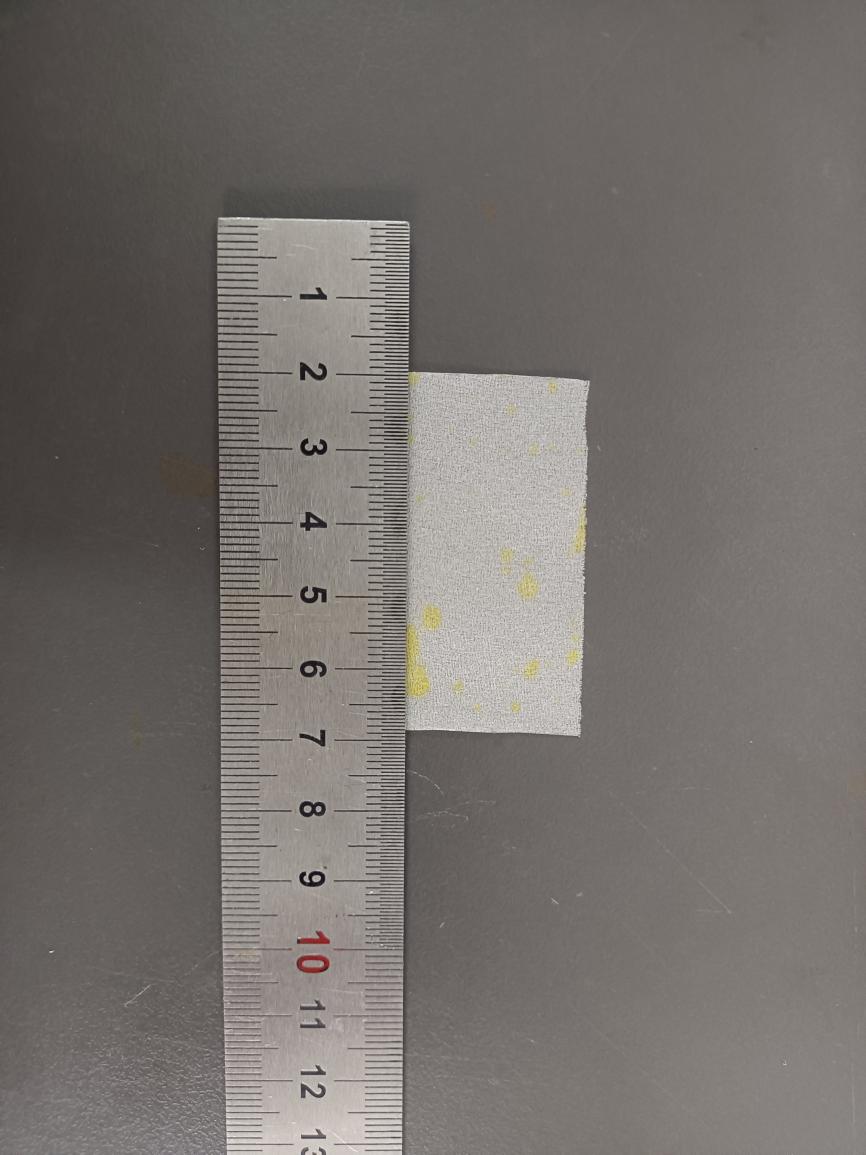
Fig.S81 Digital photograph of simulated rotten/pulverized silk fabric treated with 14% PEI + 0.5% PPEGDE + 1% IPP


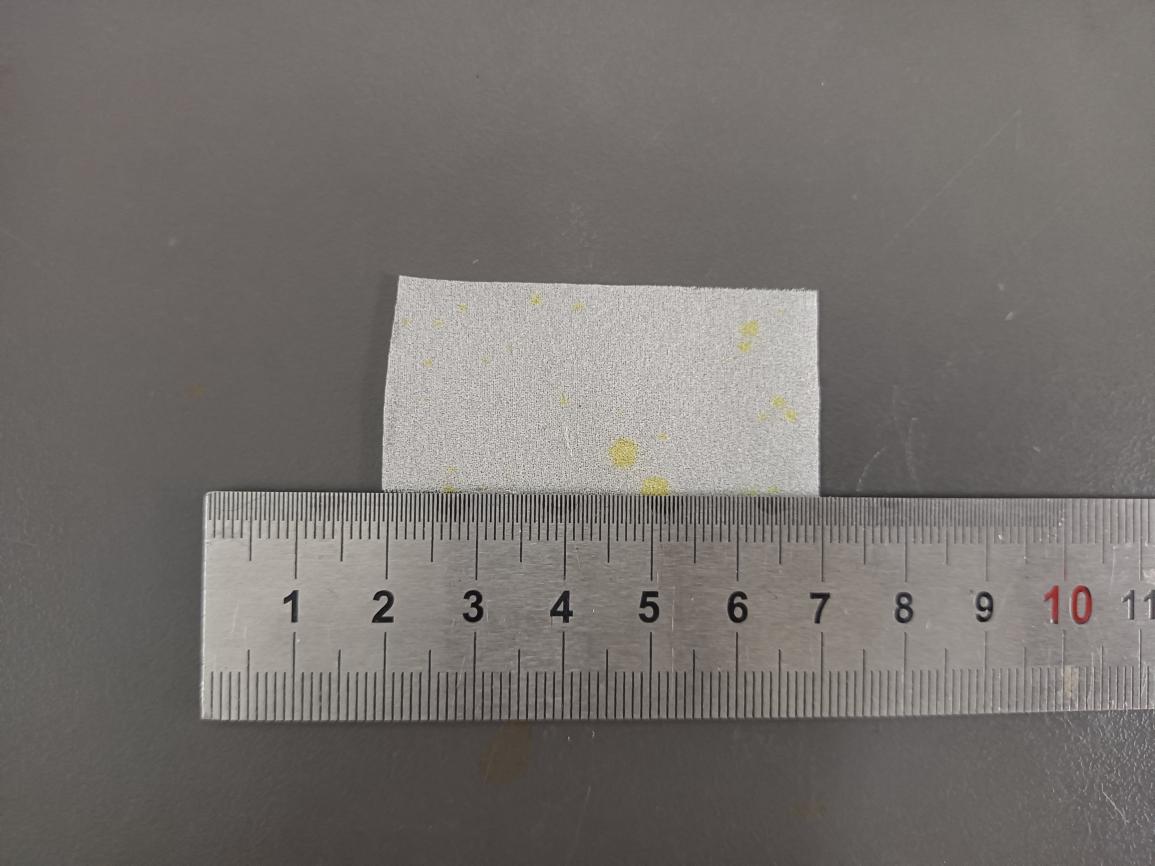


Fig.S82 Digital photograph of simulated rotten/pulverized silk fabric treated with 14% PEI + 1% PPEGDE + 1% IPP


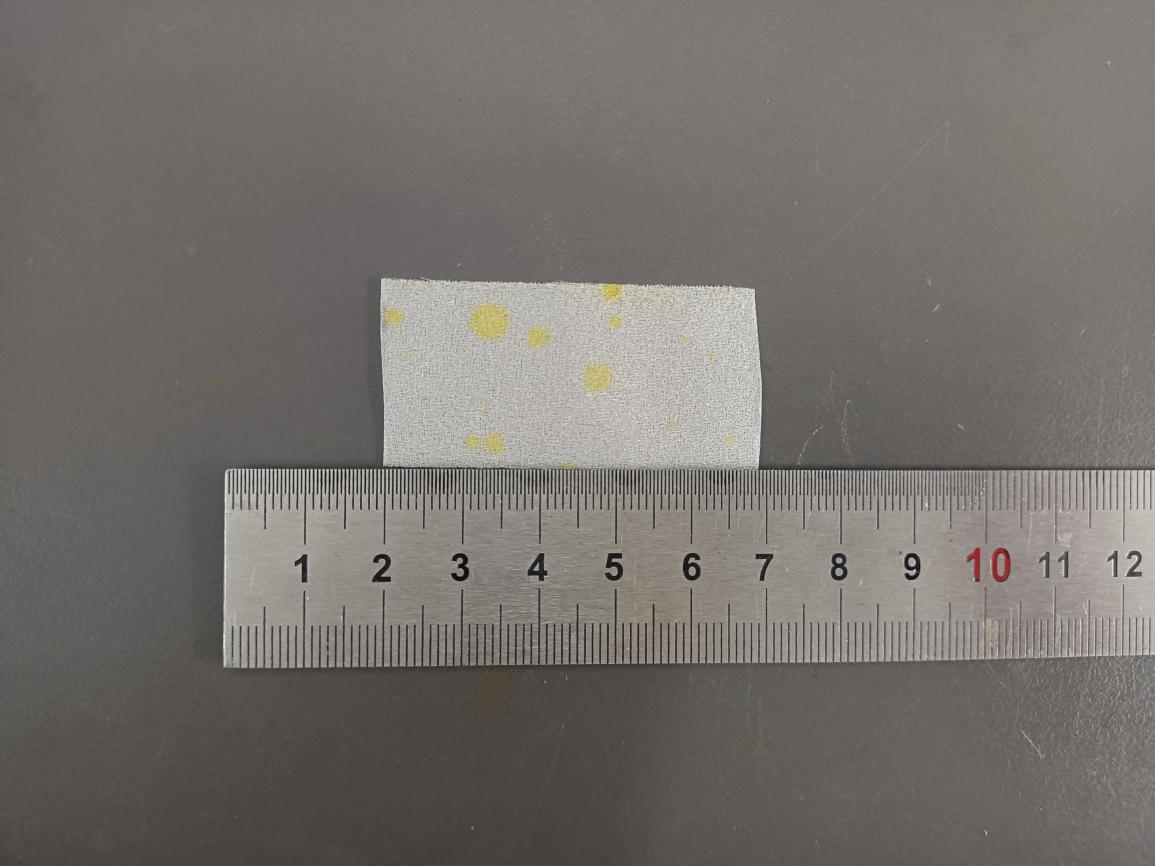


Fig.S83 Digital photograph of simulated rotten/pulverized silk fabric treated with 14% PEI + 1.5% PPEGDE + 1% IPP


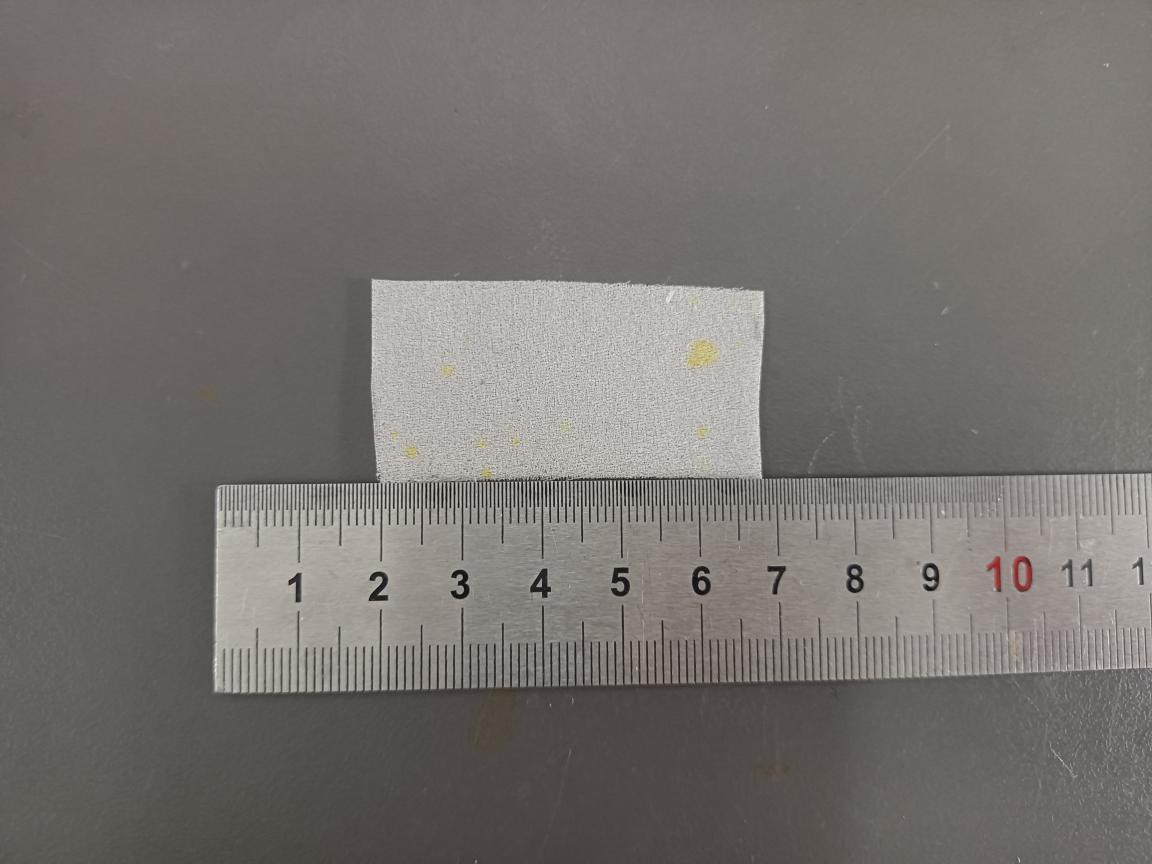


Fig.S84 Digital photograph of simulated rotten/pulverized silk fabric treated with 14% PEI + 2% PPEGDE + 1% IPP


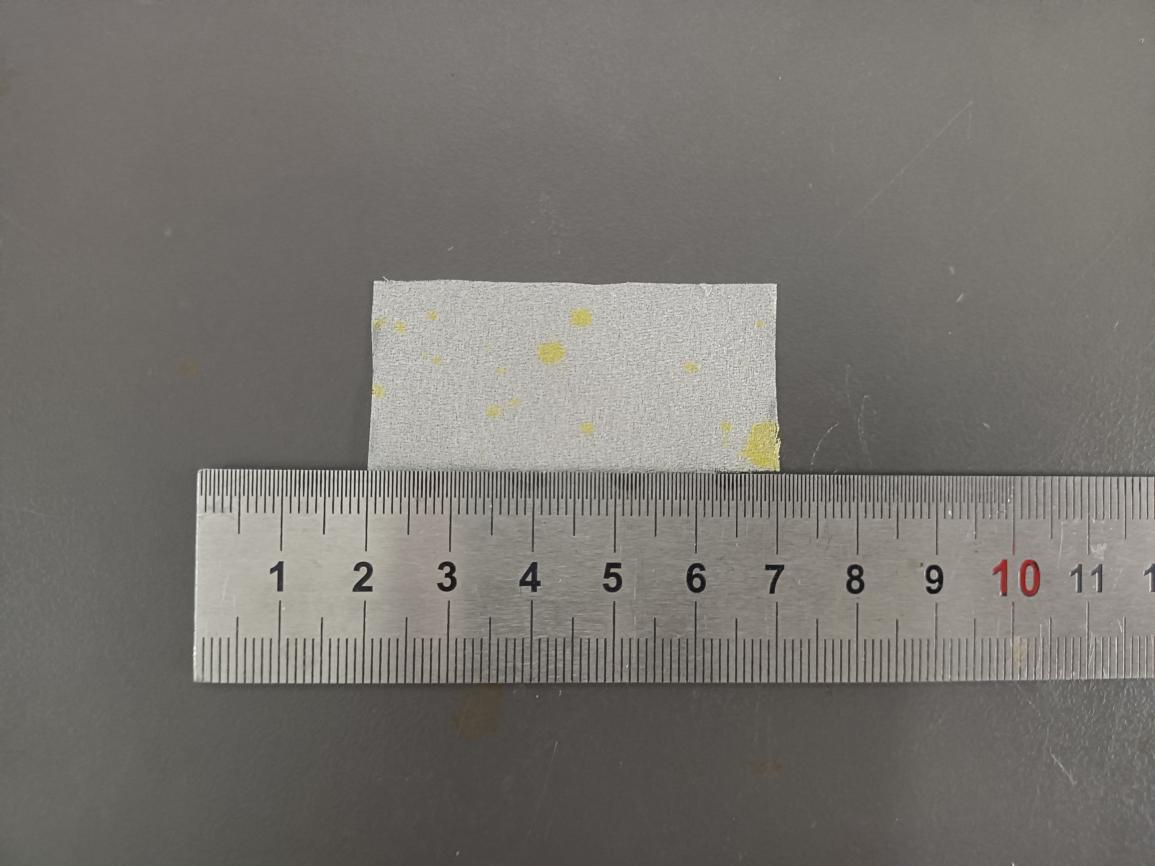


Fig.S85 Digital photograph of simulated rotten/pulverized silk fabric treated with 14% PEI + 2.5% PPEGDE + 1% IPP


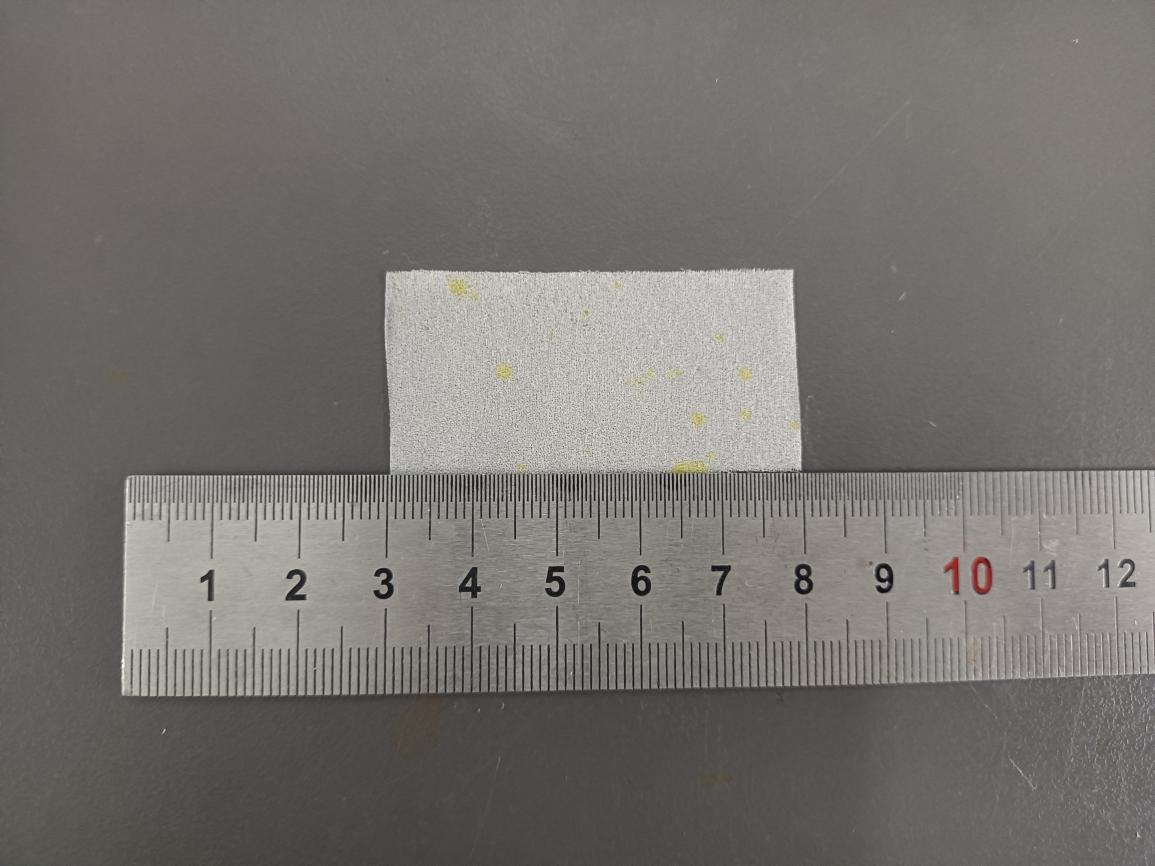


Fig.S86 Digital photograph of simulated rotten/pulverized silk fabric treated with 14% PE I+ 3% PPEGDE + 1% IPP


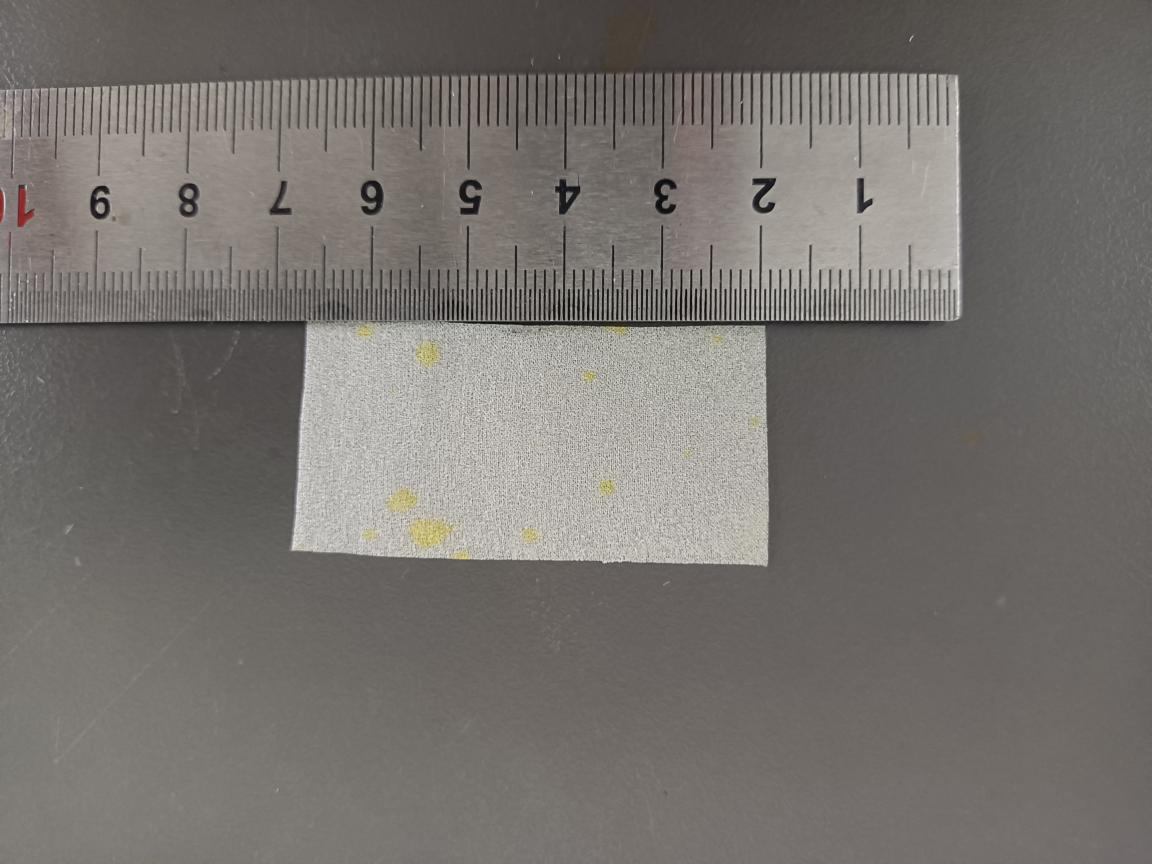


Fig.S87 Digital photograph of simulated rotten/pulverized silk fabric treated with 14% PEI + 3.5% PPEGDE + 1% IPP


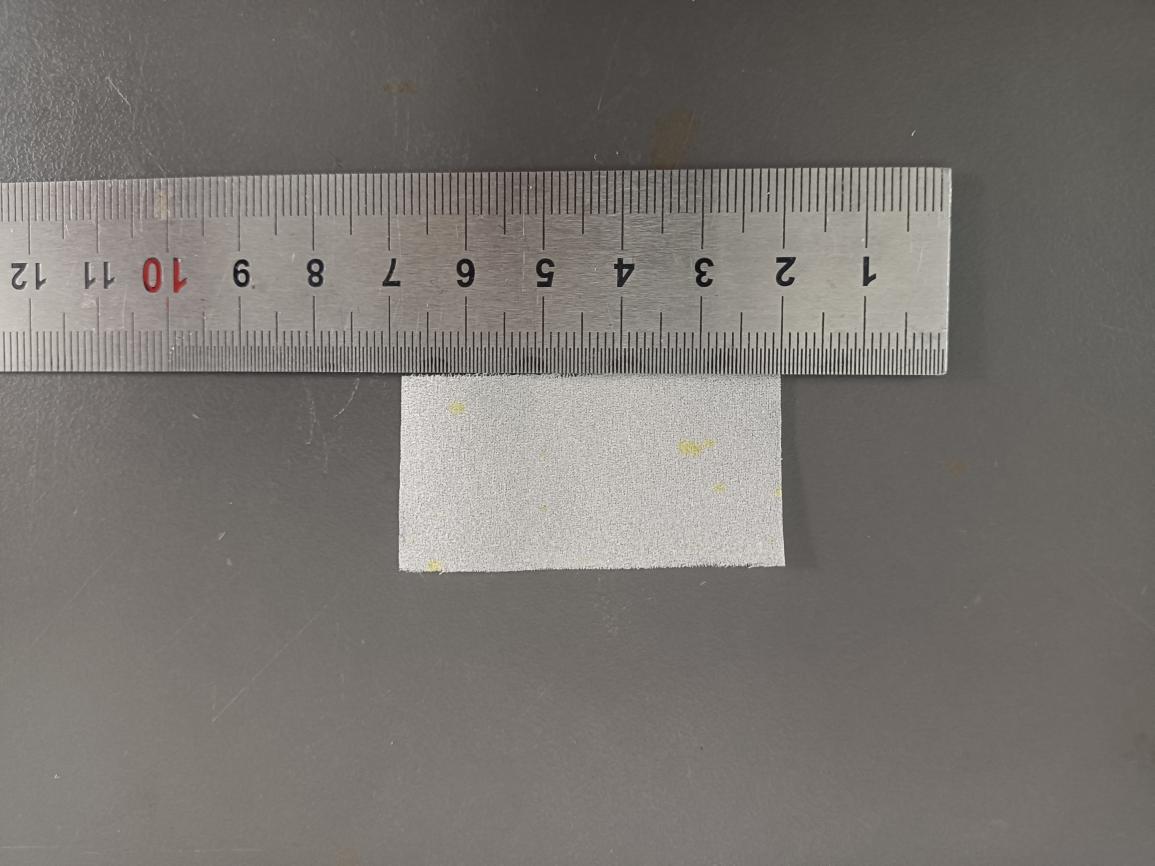


Fig.S88 Digital photograph of simulated rotten/pulverized silk fabric treated with 14% PEI + 4% PPEGDE + 1% IPP


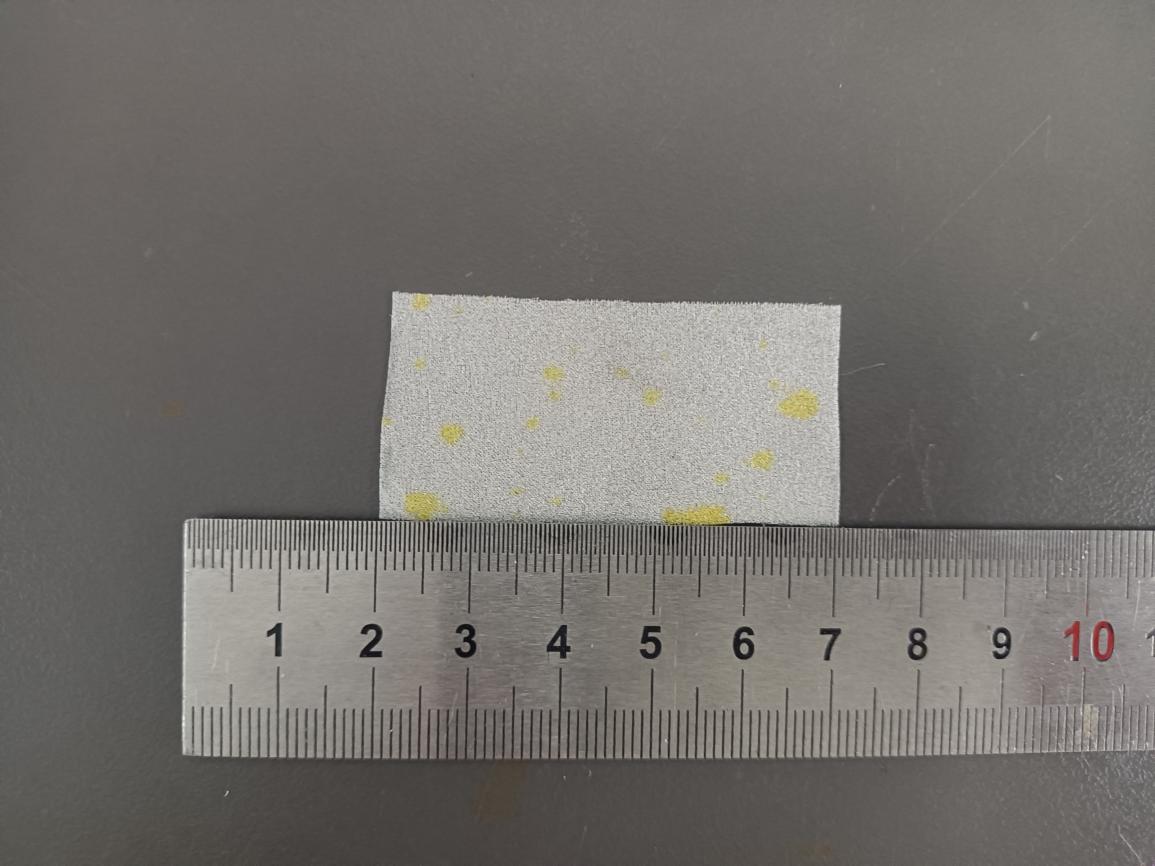


Fig.S89 Digital photograph of simulated rotten/pulverized silk fabric treated with 14% PEI + 4.5% PPEGDE + 1% IPP


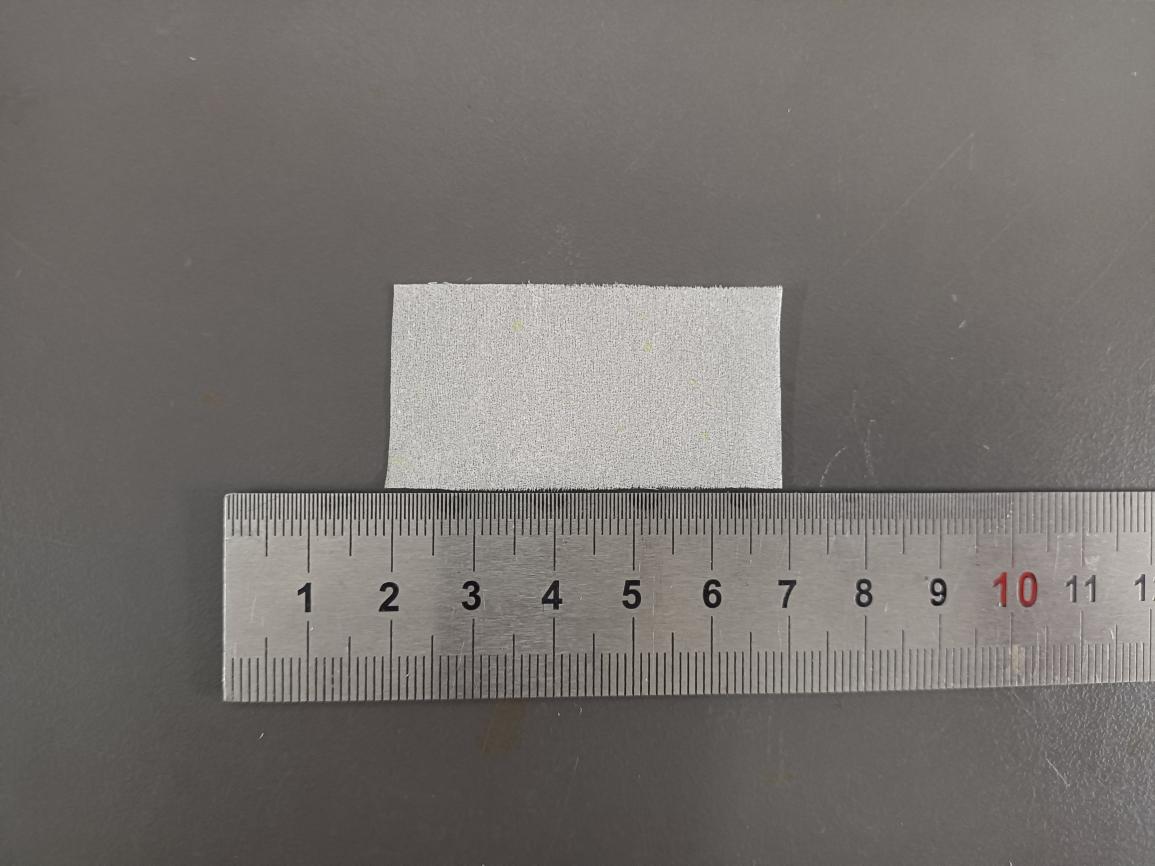


Fig.S90 Digital photograph of simulated rotten/pulverized silk fabric treated with 14% PEI + 5% PPEGDE + 1% IPP


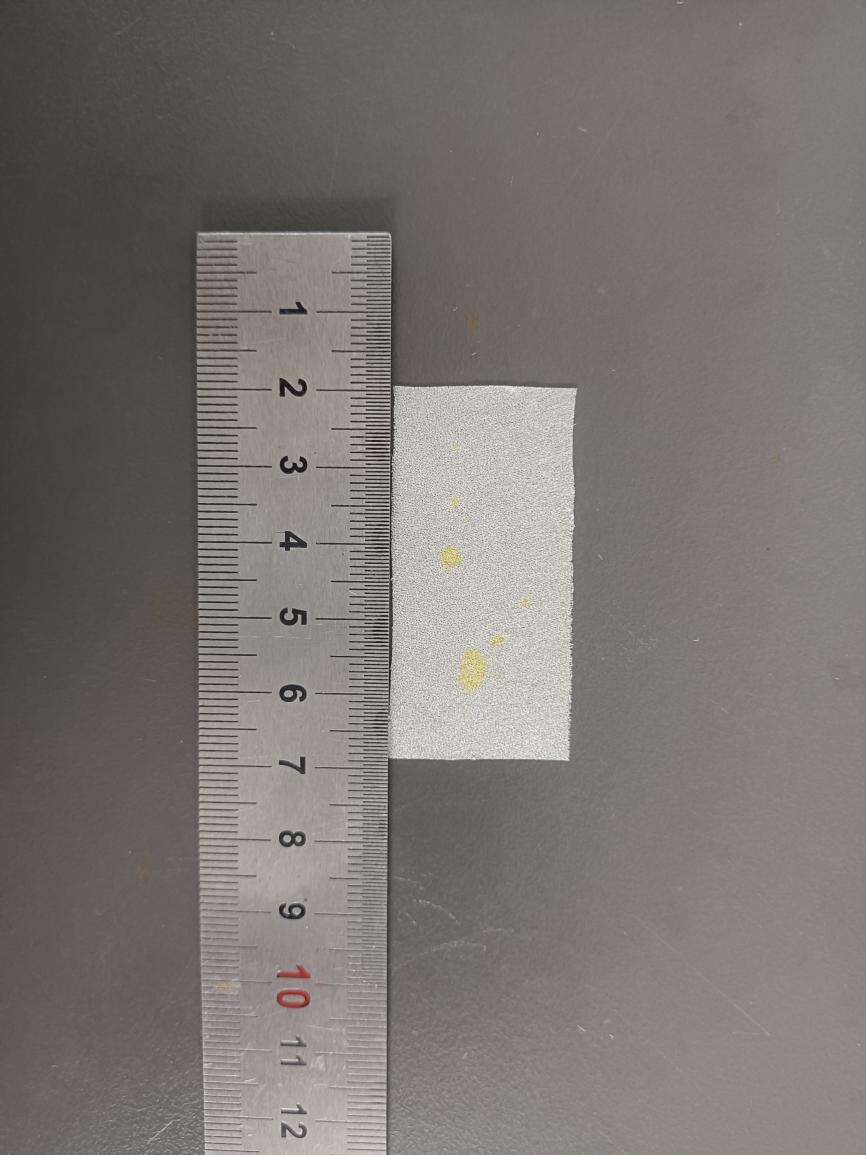
Fig.S91 Digital photograph of simulated rotten/pulverized silk fabric treated with 16% PEI + 0% PPEGDE + 1% IPP


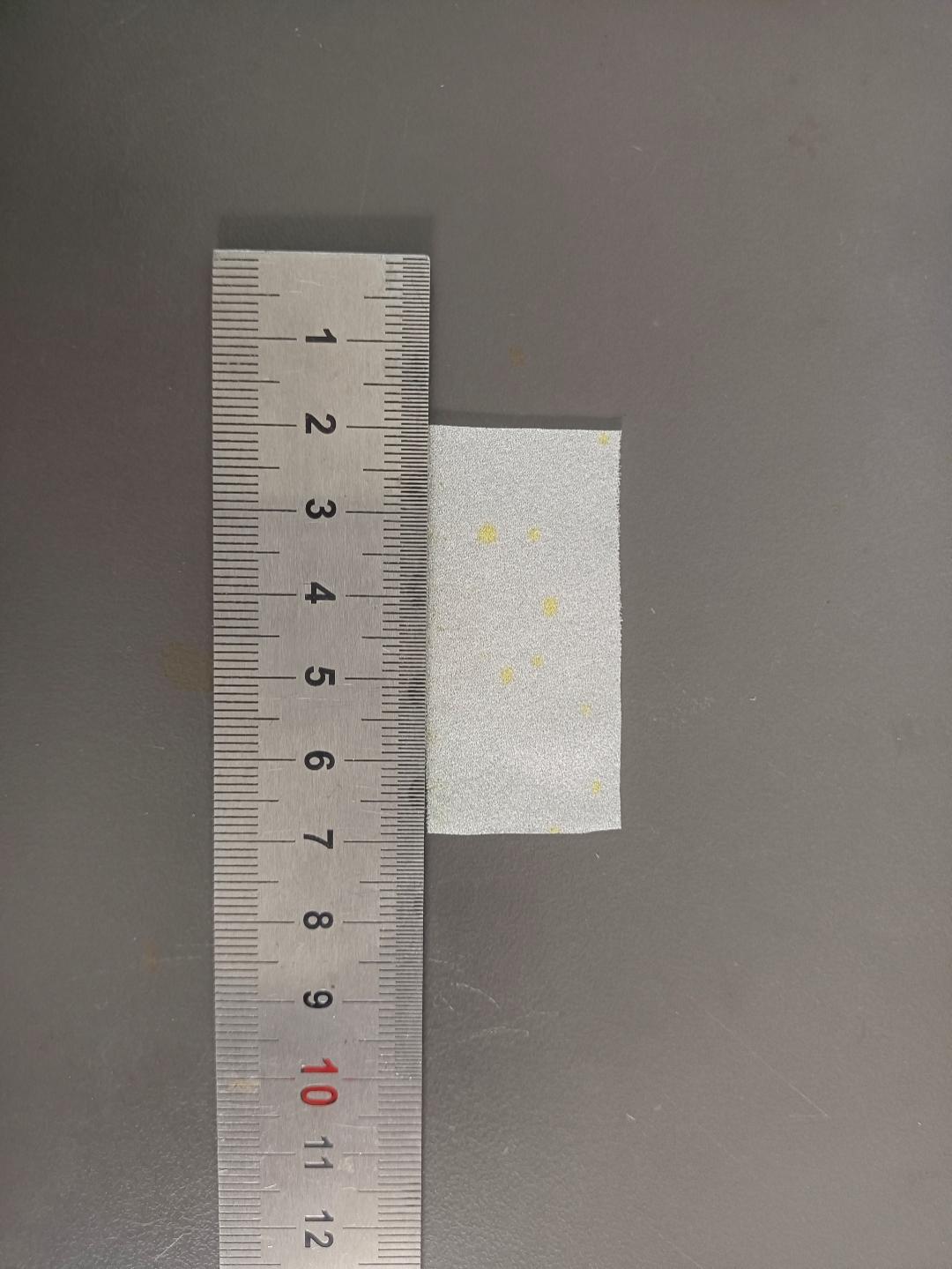
Fig.S92 Digital photograph of simulated rotten/pulverized silk fabric treated with 16% PEI + 0.5% PPEGDE + 1% IPP


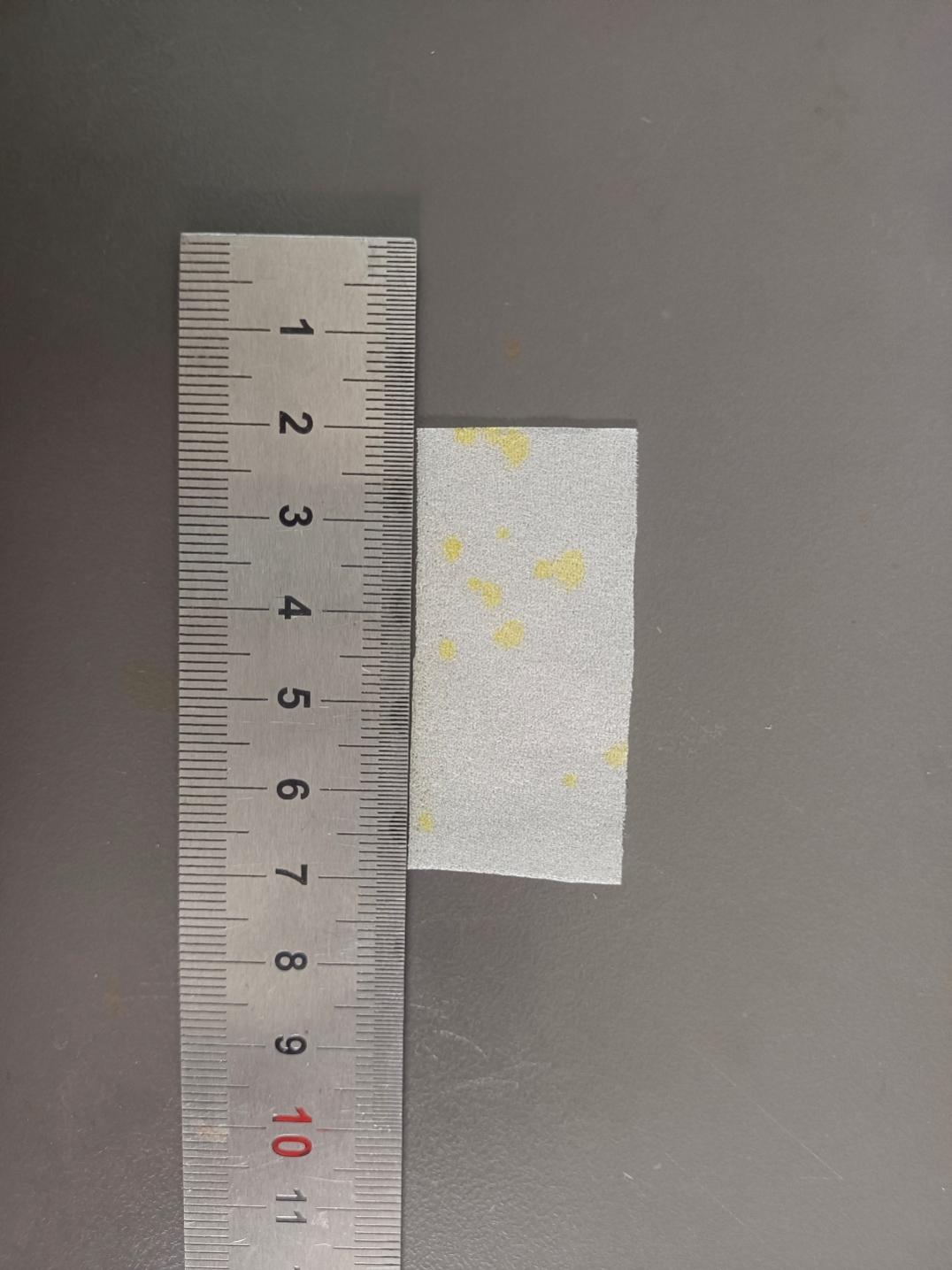
Fig.S93 Digital photograph of simulated rotten/pulverized silk fabric treated with 16% PEI + 1% PPEGDE + 1% IPP


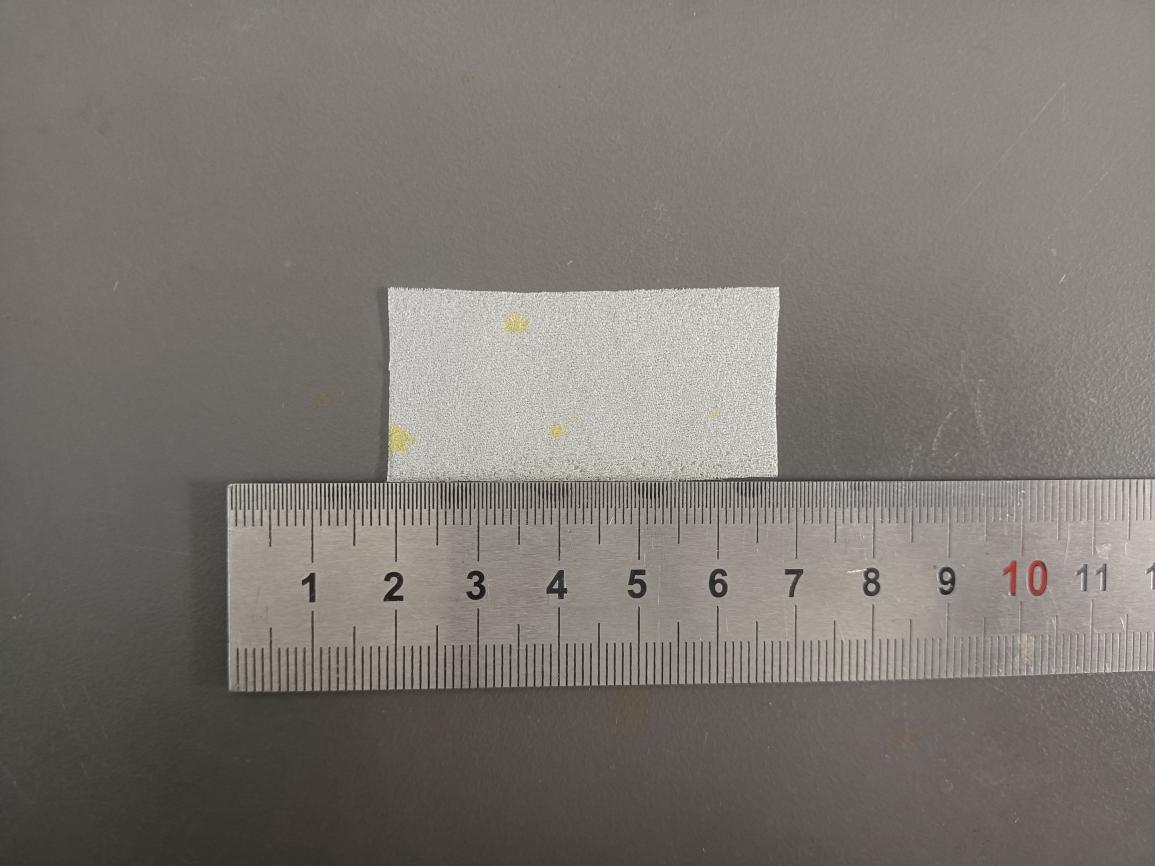


Fig.S94 Digital photograph of simulated rotten/pulverized silk fabric treated with 16% PEI+ 1.5% PPEGDE + 1% IPP


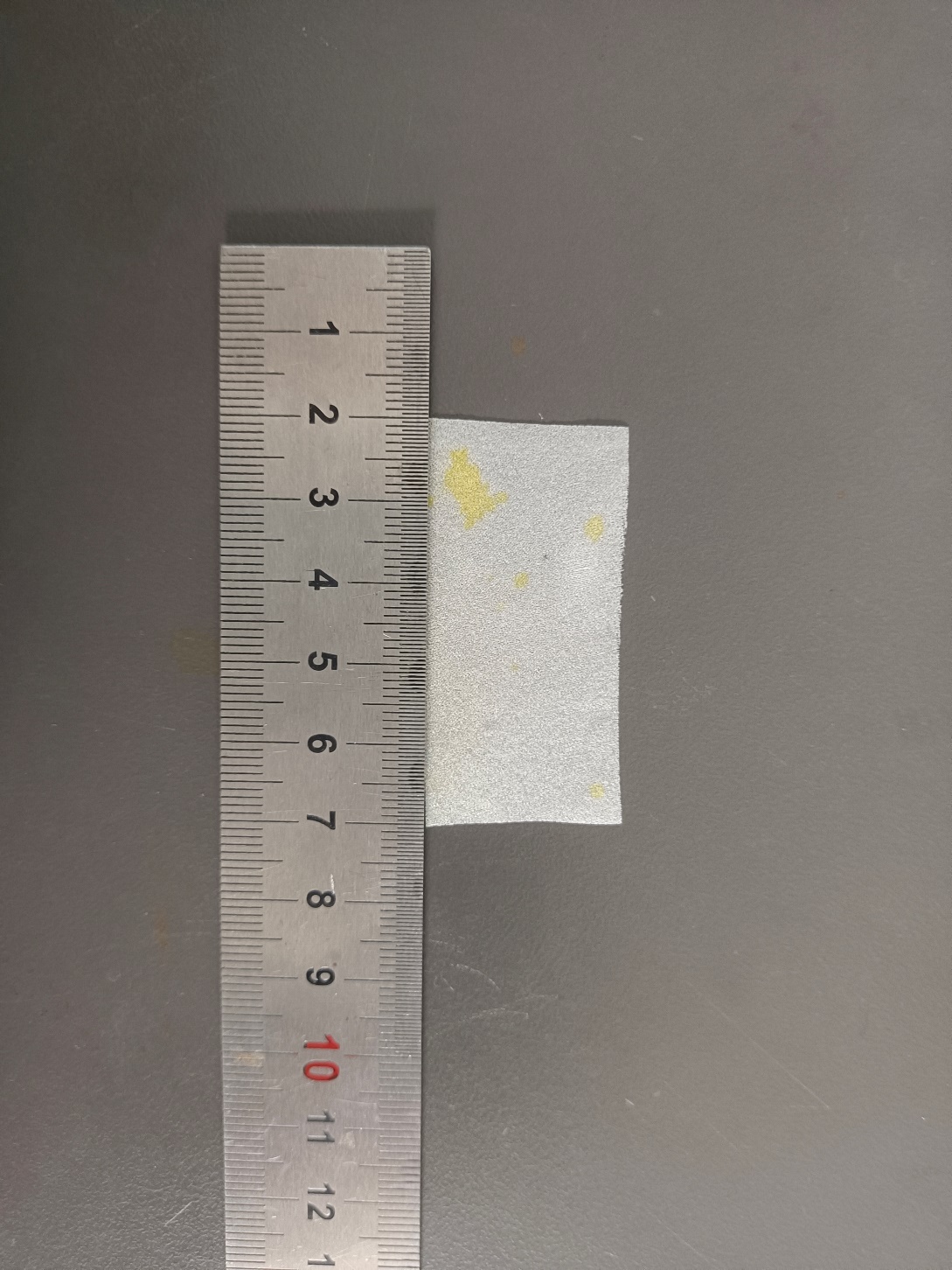
Fig.S95 Digital photograph of simulated rotten/pulverized silk fabric treated with 16% PEI + 2% PPEGDE + 1% IPP


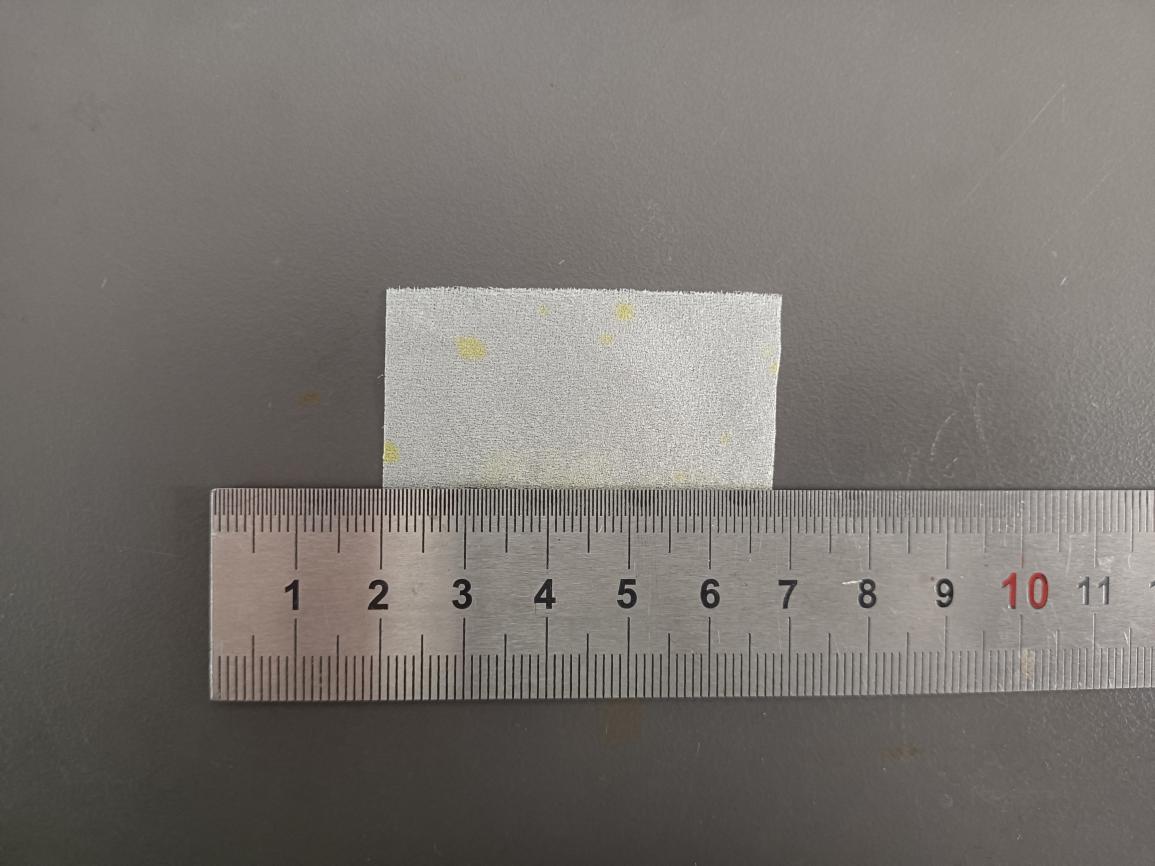


Fig.S96 Digital photograph of simulated rotten/pulverized silk fabric treated with 16% PEI +2.5% PPEGDE + 1% IPP


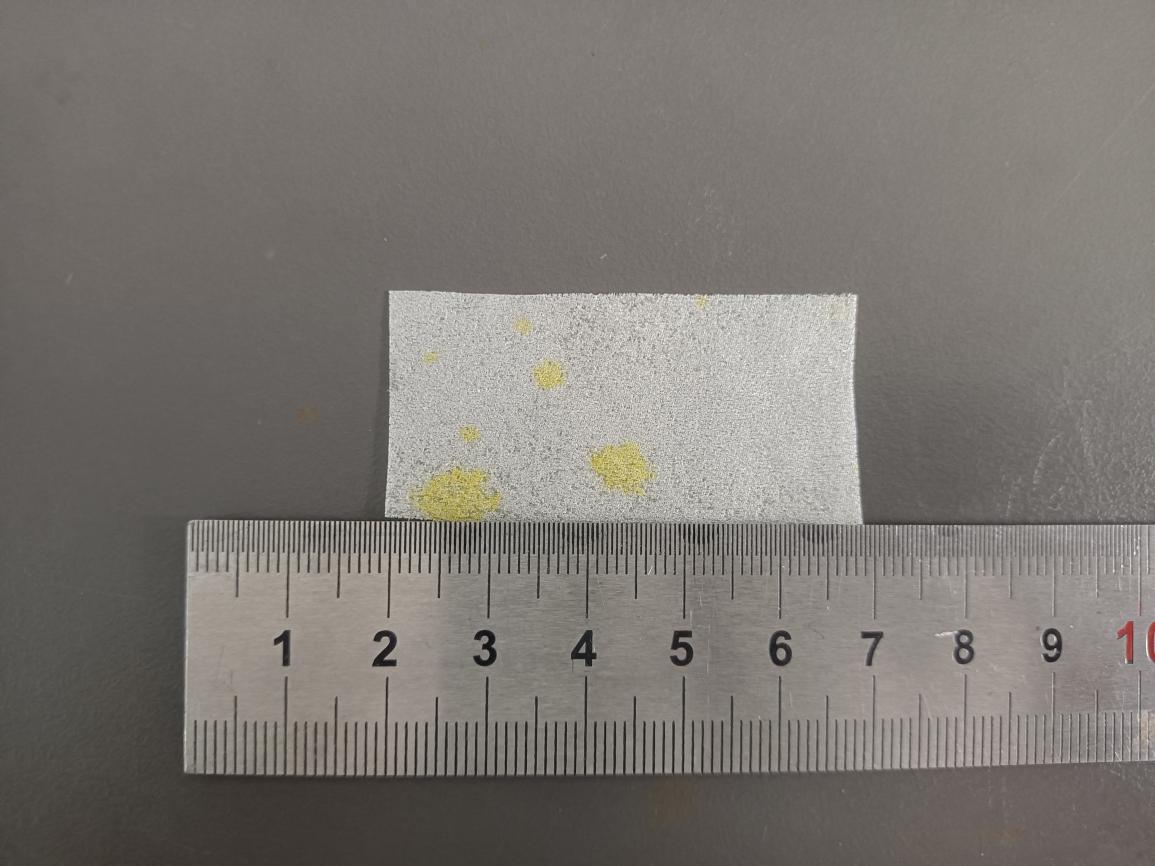


Fig.S97 Digital photograph of simulated rotten/pulverized silk fabric treated with 16% PEI + 3% PPEGDE + 1% IPP


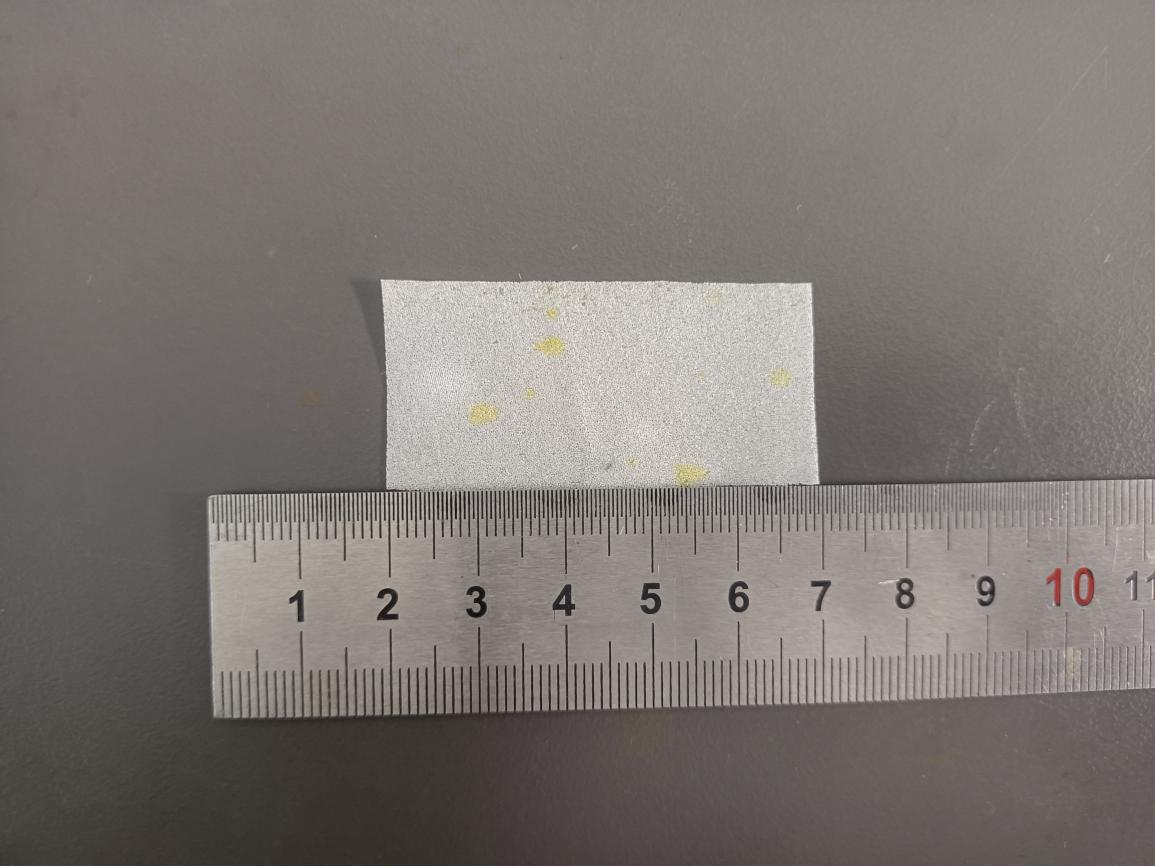


Fig.S98 Digital photograph of simulated rotten/pulverized silk fabric treated with 16% PEI + 3.5% PPEGDE + 1% IPP


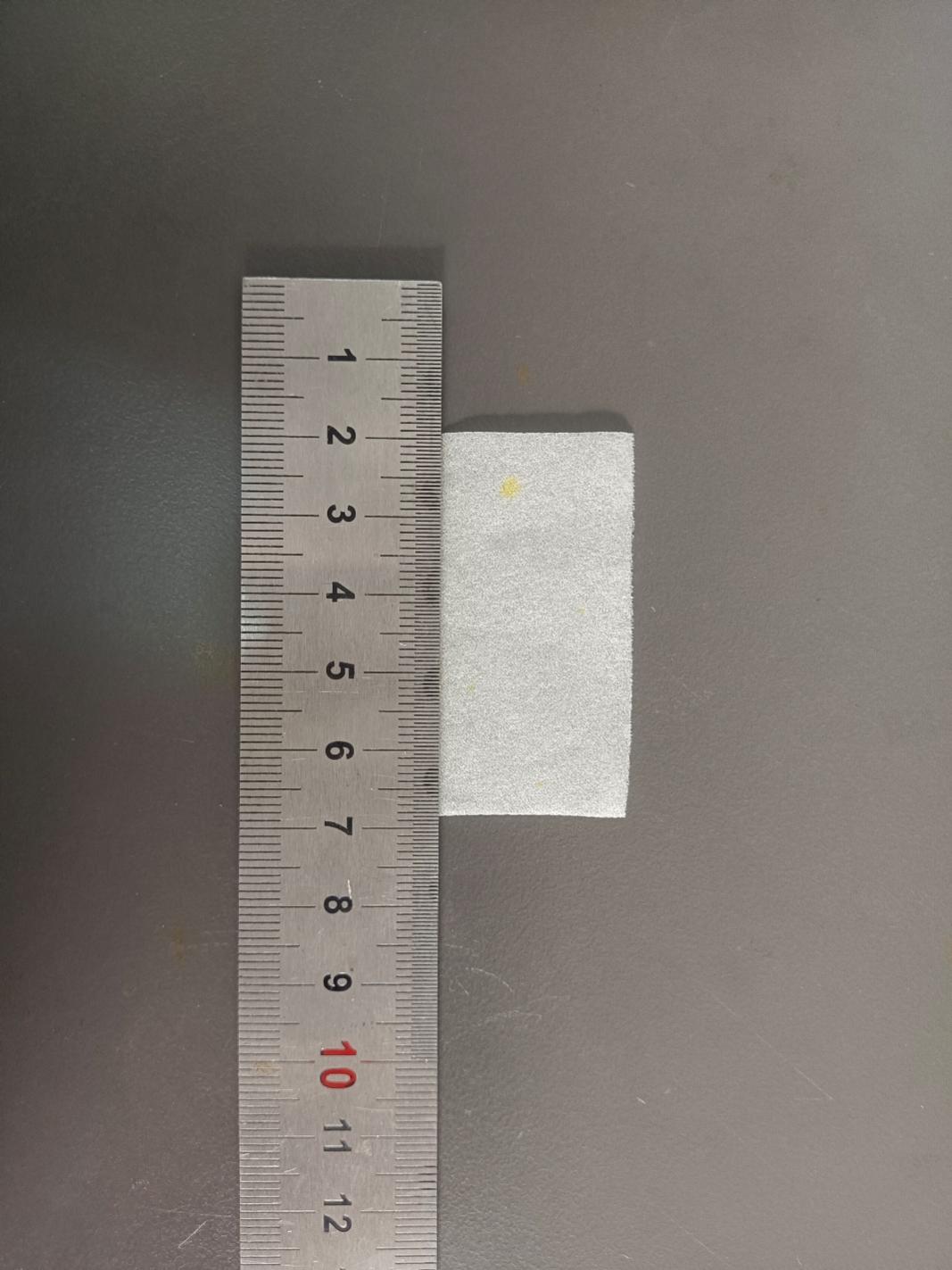
Fig.S99 Digital photograph of simulated rotten/pulverized silk fabric treated with 16% PEI + 4% PPEGDE + 1% IPP

Fig.S100 Digital photograph of simulated rotten/pulverized silk fabric treated with 16% PEI +4.5% PPEGDE + 1% IPP

Fig.S101 Digital photograph of simulated rotten/pulverized silk fabric treated with 16% PEI + 5% PPEGDE + 1% IPP

Fig.S102 Digital photograph of simulated rotten/pulverized silk fabric treated with 18% PEI + 0% PPEGDE + 1% IPP

Fig.S103 Digital photograph of simulated rotten/pulverized silk fabric treated with 18% PEI + 0.5% PPEGDE + 1% IPP

Fig.S104 Digital photograph of simulated rotten/pulverized silk fabric treated with 18% PEI + 1% PPEGDE + 1% IPP

Fig.S105 Digital photograph of simulated rotten/pulverized silk fabric treated with 18% PEI + 1.5% PPEGDE + 1% IPP

Fig.S106 Digital photograph of simulated rotten/pulverized silk fabric treated with 18% PEI + 2% PPEGDE + 1% IPP

Fig.S107 Digital photograph of simulated rotten/pulverized silk fabric treated with 18% PEI + 2.5% PPEGDE + 1% IPP

Fig.S108 Digital photograph of simulated rotten/pulverized silk fabric treated with 18% PEI + 3% PPEGDE + 1% IPP

Fig.S109 Digital photograph of simulated rotten/pulverized silk fabric treated with 18% PEI + 3.5% PPEGDE + 1% IPP

Fig.S110 Digital photograph of simulated rotten/pulverized silk fabric treated with 18% PEI + 4% PPEGDE + 1% IPP

Fig.S111 Digital photograph of simulated rotten/pulverized silk fabric treated with 18% PEI + 4.5% PPEGDE + 1% IPP

Fig.S112 Digital photograph of simulated rotten/pulverized silk fabric treated with 18% PEI + 5% PPEGDE + 1% IPP

Fig.S113 Digital photograph of simulated rotten/pulverized silk fabric treated with 20% PEI + 0% PPEGDE + 1% IPP

Fig.S114 Digital photograph of simulated rotten/pulverized silk fabric treated with 20% PEI + 0.5% PPEGDE + 1% IPP

Fig.S115 Digital photograph of simulated rotten/pulverized silk fabric treated with 20% PEI + 1% PPEGDE + 1% IPP

Fig.S116 Digital photograph of simulated rotten/pulverized silk fabric treated with 20% PEI + 1.5% PPEGDE + 1% IPP

Fig.S117 Digital photograph of simulated rotten/pulverized silk fabric treated with 20% PEI + 2% PPEGDE + 1% IPP

Fig.S118 Digital photograph of simulated rotten/pulverized silk fabric treated with 20% PEI + 2.5% PPEGDE + 1% IPP

Fig.S119 Digital photograph of simulated rotten/pulverized silk fabric treated with 20% PEI + 3% PPEGDE + 1% IPP

Fig.S120 Digital photograph of simulated rotten/pulverized silk fabric treated with 20% PEI + 3.5% PPEGDE + 1% IPP

Fig.S121 Digital photograph of simulated rotten/pulverized silk fabric treated with 20% PEI + 4% PPEGDE + 1% IPP

Fig.S122 Digital photograph of simulated rotten/pulverized silk fabric treated with 20% PEI + 4.5% PPEGDE + 1% IPP

Fig.S122 Digital photograph of simulated rotten/pulverized silk fabric treated with 20% PEI + 5% PPEGDE + 1% IPP

Table. S1 Stiffness tests of simulated rotten/pulverized silk fabrics with different treatments

| Sample | Weight (g/m^2^) | Stiffness (mN·cm) |
| --- | --- | --- |
| Original silk fabric | 65.20 | 7.86 |
|  |  |  |
|  |  |  |
| Rotten/pulverized silk fabric | 58.00 | 10.35 |
|  |  |  |
|  |  |  |
| 0 wt% PEI + 0 wt% PPEGDE | 59.20 | 2.24 |
|  |  |  |
|  |  |  |
| 0 wt% PEI + 0.5 wt% PPEGDE | 60.20 | 2.95 |
|  |  |  |
|  |  |  |
| 0 wt% PEI + 1 wt% PPEGDE | 60.50 | 3.82 |
|  |  |  |
|  |  |  |
| 0 wt % PEI + 1.5 wt% PPEGDE | 60.70 | 5.24 |
|  |  |  |
|  |  |  |
| 0 wt% PEI + 2 wt% PPEGDE | 65.20 | 5.88 |
|  |  |  |
|  |  |  |
| 0 wt% PEI + 2.5 wt% PPEGDE | 65.32 | 6.17 |
|  |  |  |
|  |  |  |
| 0 wt% PEI + 3 wt% PPEGDE | 65.51 | 7.69 |
|  |  |  |
|  |  |  |
| 0 wt% PEI + 3.5 wt% PPEGDE | 84.00 | 10.60 |
|  |  |  |
|  |  |  |
| 0 wt% PEI + 4 wt% PPEGDE | 70.80 | 12.43 |
|  |  |  |
|  |  |  |
| 0 wt% PEI + 4.5 wt% PPEGDE | 71.10 | 15.71 |
|  |  |  |
|  |  |  |
| 0 wt% PEI + 5 wt% PPEGDE | 71.25 | 17.32 |
|  |  |  |
|  |  |  |
| 2 wt% PEI + 0 wt% PPEGDE | 56.00 | 2.56 |
|  |  |  |
|  |  |  |
| 2 wt% PEI + 0.5 wt% PPEGDE | 56.80 | 4.71 |
|  |  |  |
|  |  |  |
| 2 wt% PEI + 1 wt% PPEGDE | 61.60 | 4.93 |
|  |  |  |
|  |  |  |
| 2 wt% PEI + 1.5 wt% PPEGDE | 70.40 | 6.78 |
|  |  |  |
|  |  |  |
| 2 wt% PEI + 2 wt% PPEGDE | 74.00 | 7.69 |
|  |  |  |
|  |  |  |
| 2 wt% PEI + 2.5 wt% PPEGDE | 78.00 | 8.79 |
|  |  |  |
|  |  |  |
| 2 wt% PEI + 3 wt% PPEGDE | 84.80 | 10.16 |
|  |  |  |
|  |  |  |
| 2 wt% PEI + 3.5 wt% PPEGDE | 85.60 | 10.88 |
|  |  |  |
|  |  |  |
| 2 wt% PEI + 4 wt% PPEGDE | 87.20 | 16.46 |
|  |  |  |
|  |  |  |
| 2 wt% PEI + 4.5 wt% PPEGDE | 87.40 | 18.45 |
|  |  |  |
|  |  |  |
| 2 wt% PEI + 5 wt% PPEGDE | 87.50 | 18.86 |
|  |  |  |
|  |  |  |
| 4 wt% PEI + 0 wt% PPEGDE | 76.00 | 2.59 |
|  |  |  |
|  |  |  |
| 4 wt% PEI + 0.5 wt% PPEGDE | 77.60 | 3.09 |
|  |  |  |
|  |  |  |
| 4 wt% PEI + 1 wt% PPEGDE | 84.00 | 5.52 |
|  |  |  |
|  |  |  |
| 4 wt% PEI + 1.5 wt% PPEGDE | 95.60 | 6.82 |
|  |  |  |
|  |  |  |
| 4 wt% PEI + 2 wt% PPEGDE | 96.00 | 8.39 |
|  |  |  |
|  |  |  |
| 4 wt% PEI + 2.5 wt% PPEGDE | 96.20 | 8.73 |
|  |  |  |
|  |  |  |
| 4 wt% PEI + 3 wt% PPEGDE | 96.30 | 9.37 |
|  |  |  |
|  |  |  |
| 4 wt% PEI + 3.5 wt% PPEGDE | 112.00 | 13.97 |
|  |  |  |
|  |  |  |
| 4 wt% PEI + 4 wt% PPEGDE | 115.60 | 14.28 |
|  |  |  |
|  |  |  |
| 4 wt% PEI + 4.5 wt% PPEGDE | 117.30 | 15.22 |
|  |  |  |
|  |  |  |
| 4 wt% PEI + 5 wt% PPEGDE | 117.50 | 23.29 |
|  |  |  |
|  |  |  |
| 6 wt% PEI + 0 wt% PPEGDE | 78.40 | 5.12 |
|  |  |  |
|  |  |  |
| 6 wt% PEI + 0.5 wt% PPEGDE | 78.60 | 9.60 |
|  |  |  |
|  |  |  |
| 6 wt% PEI + 1 wt% PPEGDE | 80.40 | 16.95 |
|  |  |  |
|  |  |  |
| 6 wt% PEI + 1.5 wt% PPEGDE | 97.60 | 17.75 |
|  |  |  |
|  |  |  |
| 6 wt% PEI + 2 wt% PPEGDE | 98.80 | 19.27 |
|  |  |  |
|  |  |  |
| 6 wt% PEI + 2.5 wt% PPEGDE | 99.20 | 19.51 |
|  |  |  |
|  |  |  |
| 6 wt% PEI + 3 wt% PPEGDE | 101.30 | 20.44 |
|  |  |  |
|  |  |  |
| 6 wt% PEI + 3.5 wt% PPEGDE | 101.60 | 20.82 |
|  |  |  |
|  |  |  |
| 6 wt% PEI + 4 wt% PPEGDE | 101.80 | 45.00 |
|  |  |  |
|  |  |  |
| 6 wt% PEI + 4.5 wt% PPEGDE | 102.20 | 45.49 |
|  |  |  |
|  |  |  |
| 6 wt% PEI + 5 wt% PPEGDE | 110.40 | 48.98 |
|  |  |  |
|  |  |  |
| 8 wt% PEI + 0 wt% PPEGDE | 78.00 | 12.86 |
|  |  |  |
|  |  |  |
| 8 wt% PEI + 0.5 wt% PPEGDE | 79.60 | 14.14 |
|  |  |  |
|  |  |  |
| 8 wt% PEI + 1 wt% PPEGDEDE | 90.00 | 15.71 |
|  |  |  |
|  |  |  |
| 8 wt% PEI + 1.5 wt% PPEGDE | 93.20 | 16.32 |
|  |  |  |
|  |  |  |
| 8 wt% PEI + 2 wt% PPEGDE | 97.20 | 17.20 |
|  |  |  |
|  |  |  |
| 8 wt% PEI + 2.5 wt% PPEGDE | 98.30 | 18.56 |
|  |  |  |
|  |  |  |
| 8 wt% PEI + 3 wt% PPEGDE | 105.60 | 19.65 |
|  |  |  |
|  |  |  |
| 8 wt% PEI + 3.5 wt% PPEGDE | 105.80 | 28.63 |
|  |  |  |
|  |  |  |
| 8 wt% PEI + 4 wt% PPEGDE | 105.00 | 30.52 |
|  |  |  |
|  |  |  |
| 8 wt% PEI + 4.5 wt% PPEGDE | 105.30 | 32.64 |
|  |  |  |
|  |  |  |
| 8 wt% PEI + 5 wt% PPEGDE | 105.60 | 33.86 |
|  |  |  |
|  |  |  |
| 10 wt% PEI + 0 wt% PPEGDE | 96.00 | 7.69 |
|  |  |  |
|  |  |  |
| 10 wt% PEI + 0.5 wt% PPEGDE | 103.00 | 8.33 |
|  |  |  |
|  |  |  |
| 10 wt% PEI + 1wt%PPEGDE | 105.00 | 9.68 |
|  |  |  |
|  |  |  |
| 10 wt% PEI + 1.5 wt% PPEGDE | 107.00 | 11.25 |
|  |  |  |
|  |  |  |
| 10 wt% PEI + 2 wt% PPEGDE | 108.50 | 12.86 |
|  |  |  |
|  |  |  |
| 10 wt% PEI + 2.5 wt% PPEGDE | 109.20 | 13.85 |
|  |  |  |
|  |  |  |
| 10 wt% PEI + 3 wt% PPEGDE | 110.30 | 15.68 |
|  |  |  |
|  |  |  |
| 10 wt% PEI + 3.5 wt% PPEGDE | 111.60 | 22.10 |
|  |  |  |
|  |  |  |
| 10 wt% PEI + 4 wt% PPEGDE | 112.40 | 22.36 |
|  |  |  |
|  |  |  |
| 10 wt% PEI + 4.5 wt% PPEGDE | 113.80 | 23.81 |
|  |  |  |
|  |  |  |
| 10 wt% PEI + 5 wt% PPEGDE | 115.20 | 25.71 |
|  |  |  |
|  |  |  |
| 12 wt% PEI + 0 wt% PPEGDE | 82.00 | 8.79 |
|  |  |  |
|  |  |  |
| 12 wt% PEI + 0.5 wt% PPEGDE | 98.00 | 11.11 |
|  |  |  |
|  |  |  |
| 12 wt% PEI + 1 wt% PPEGDE | 103.00 | 14.91 |
|  |  |  |
|  |  |  |
| 12 wt% PEI + 1.5 wt% PPEGDE | 105.00 | 16.13 |
|  |  |  |
|  |  |  |
| 12 wt% PEI + 2 wt% PPEGDE | 107.00 | 20.20 |
|  |  |  |
|  |  |  |
| 12 wt% PEI + 2.5 wt% PPEGDE | 108.00 | 23.57 |
|  |  |  |
|  |  |  |
| 12 wt% PEI + 3 wt% PPEGDE | 112.00 | 24.96 |
|  |  |  |
|  |  |  |
| 12 wt% PEI + 3.5 wt% PPEGDE | 116.30 | 25.37 |
|  |  |  |
|  |  |  |
| 12 wt% PEI + 4 wt% PPEGDE | 118.90 | 29.79 |
|  |  |  |
|  |  |  |
| 12 wt% PEI + 4.5 wt% PPEGDE | 120.50 | 40.41 |
|  |  |  |
|  |  |  |
| 12 wt% PEI + 5 wt% PPEGDE | 124.10 | 42.79 |
|  |  |  |
|  |  |  |
| 14 wt% PEI + 0 wt% PPEGDE | 90.00 | 4.47 |
|  |  |  |
|  |  |  |
| 14 wt% PEI + 0.5 wt% PPEGDE | 91.00 | 4.53 |
|  |  |  |
|  |  |  |
| 14 wt% PEI + 1 wt% PPEGDE | 97.00 | 10.40 |
|  |  |  |
|  |  |  |
| 14 wt% PEI + 1.5 wt% PPEGDE | 102.00 | 11.11 |
|  |  |  |
|  |  |  |
| 14 wt% PEI + 2 wt% PPEGDE | 105.00 | 16.91 |
|  |  |  |
|  |  |  |
| 14 wt% PEI + 2.5 wt% PPEGDE | 108.00 | 18.02 |
|  |  |  |
|  |  |  |
| 14 wt% PEI + 3 wt% PPEGDE | 111.00 | 19.23 |
|  |  |  |
|  |  |  |
| 14 wt% PEI + 3.5 wt% PPEGDE | 112.00 | 23.58 |
|  |  |  |
|  |  |  |
| 14 wt% PEI + 4 wt% PPEGDE | 119.00 | 24.51 |
|  |  |  |
|  |  |  |
| 14 wt% PEI + 4.5wt% PPEGDE | 127.00 | 33.04 |
|  |  |  |
|  |  |  |
| 14 wt% PEI + 5 wt% PPEGDE | 130.00 | 65.00 |
|  |  |  |
|  |  |  |
| 16 wt% PEI + 0 wt% PPEGDE | 104.00 | 8.33 |
|  |  |  |
|  |  |  |
| 16 wt% PEI + 0.5 wt% PPEGDE | 106.00 | 12.53 |
|  |  |  |
|  |  |  |
| 16 wt% PEI + 1 wt% PPEGDE | 109.00 | 12.82 |
|  |  |  |
|  |  |  |
| 16 wt% PEI + 1.5 wt% PPEGDE | 112.00 | 14.14 |
|  |  |  |
|  |  |  |
| 16 wt% PEI + 2 wt% PPEGDE | 118.00 | 18.63 |
|  |  |  |
|  |  |  |
| 16 wt% PEI + 2.5 wt% PPEGDE | 119.00 | 21.99 |
|  |  |  |
|  |  |  |
| 16 wt% PEI + 3 wt% PPEGDE | 121.00 | 23.57 |
|  |  |  |
|  |  |  |
| 16 wt% PEI + 3.5 wt% PPEGDE | 122.00 | 23.81 |
|  |  |  |
|  |  |  |
| 16 wt% PEI + 4 wt % PPEGDE | 123.00 | 24.85 |
|  |  |  |
|  |  |  |
| 16 wt% PEI + 4.5 wt% PPEGDE | 125.00 | 33.16 |
|  |  |  |
|  |  |  |
| 16 wt% PEI + 5 wt% PPEGDE | 126.00 | 36.52 |
|  |  |  |
|  |  |  |
| 18 wt% PEI + 0 wt% PPEGDE | 95.00 | 24.92 |
|  |  |  |
|  |  |  |
| 18 wt% PEI + 0.5 wt% PPEGDE | 106.00 | 40.02 |
|  |  |  |
|  |  |  |
| 18 wt% PEI + 1 wt% PPEGDE | 125.00 | 40.21 |
|  |  |  |
|  |  |  |
| 18 wt% PEI + 1.5 wt% PPEGDE | 135.00 | 41.63 |
|  |  |  |
|  |  |  |
| 18 wt% PEI + 2 wt% PPEGDE | 136.00 | 43.00 |
|  |  |  |
|  |  |  |
| 18 wt% PEI + 2.5 wt% PPEGDE | 138.00 | 45.03 |
|  |  |  |
|  |  |  |
| 18 wt% PEI + 3 wt% PPEGDE | 139.00 | 45.99 |
|  |  |  |
|  |  |  |
| 18 wt% PEI + 3.5 wt% PPEGDE | 140.00 | 46.51 |
|  |  |  |
|  |  |  |
| 18 wt% PEI + 4 wt% PPEGDE | 142.00 | 46.52 |
|  |  |  |
|  |  |  |
| 18 wt% PEI + 4.5 wt% PPEGDE | 143.00 | 46.87 |
|  |  |  |
|  |  |  |
| 18 wt% PEI + 5 wt% PPEGDE | 150.00 | 46.98 |
|  |  |  |
|  |  |  |
| 20 wt% PEI + 0 wt% PPEGDE | 96.00 | 11.79 |
|  |  |  |
|  |  |  |
| 20 wt% PEI + 0.5 wt% PPEGDE | 102.00 | 12.50 |
|  |  |  |
|  |  |  |
| 20 wt% PEI + 1 wt% PPEGDE | 106.00 | 12.86 |
|  |  |  |
|  |  |  |
| 20 wt% PEI + 1.5 wt% PPEGDE | 110.00 | 14.29 |
|  |  |  |
|  |  |  |
| 20 wt% PEI + 2 wt% PPEGDE | 112.00 | 20.20 |
|  |  |  |
|  |  |  |
| 20 wt% PEI + 2.5 wt% PPEGDE | 114.00 | 20.30 |
|  |  |  |
|  |  |  |
| 20 wt% PEI + 3 wt% PPEGDE | 122.00 | 23.57 |
|  |  |  |
|  |  |  |
| 20 wt% PEI + 3.5 wt% PPEGDE | 123.00 | 31.43 |
|  |  |  |
|  |  |  |
| 20 wt% PEI + 4 wt% PPEGDE | 132.00 | 33.28 |
|  |  |  |
|  |  |  |
| 20 wt% PEI + 4.5 wt% PPEGDE | 133.00 | 45.07 |
|  |  |  |
|  |  |  |
| 20 wt% PEI + 5 wt% PPEGDE | 153.00 | 46.20 |
|  |  |  |
|  |  |  |

Table.S2 Chromaticity variations of simulated rotten/pulverized silk fabrics with different treatments compared with original silk fabric

| Sample | ∆L^*^ | ∆a^*^ | ∆b^*^ | ∆E | Average |
| --- | --- | --- | --- | --- | --- |
| Rotten/pulverized silk fabric | -3.85 | -0.99 | -2.92 | 4.93 | 4.86 ± 0.12 |
|  | -3.88 | -0.98 | -2.90 | 4.94 |  |
|  | -3.87 | -0.98 | -2.51 | 4.72 |  |
| 0 wt% PEI + 0 wt% PPEGDE | 0.92 | -3.49 | -8.80 | 9.51 | 9.45 ± 0.06 |
|  | 0.84 | -3.31 | -8.80 | 9.44 |  |
|  | 1.26 | -3.22 | -8.73 | 9.39 |  |
| 0 wt% PEI + 0.5 wt% PPEGDE | 1.72 | -3.06 | -9.24 | 9.88 | 9.89 ± 0.08 |
|  | 1.38 | -2.98 | -9.41 | 9.97 |  |
|  | 1.59 | -3.14 | -9.17 | 9.82 |  |
| 0 wt% PEI + 1 wt% PPEGDE | 1.39 | -3.05 | -9.44 | 10.02 | 9.97 ± 0.04 |
|  | 1.89 | -3.09 | -9.27 | 9.95 |  |
|  | 1.46 | -3.07 | -9.35 | 9.94 |  |
| 0 wt% PEI + 1.5 wt% PPEGDE | 1.45 | -3.07 | -9.39 | 9.99 | 10.02 ± 0.04 |
|  | 1.74 | -3.22 | -9.37 | 10.06 |  |
|  | 1.51 | -3.08 | -9.40 | 10.01 |  |
| 0 wt% PEI + 2 wt% PPEGDE | 1.66 | -3.13 | -9.47 | 10.11 | 10.02 ± 0.09 |
|  | 1.23 | -3.12 | -9.35 | 9.94 |  |
|  | 1.52 | -3.12 | -9.39 | 10.01 |  |
| 0 wt% PEI + 2.5 wt% PPEGDE | 1.72 | -3.17 | -9.41 | 10.08 | 10.09 ± 0.02 |
|  | 1.53 | -3.04 | -9.53 | 10.12 |  |
|  | 1.59 | -3.06 | -9.47 | 10.08 |  |
| 0 wt % PEI + 3 wt% PPEGDE | 1.32 | -3.24 | -9.22 | 9.86 | 9.93 ± 0.07 |
|  | 1.19 | -3.24 | -9.34 | 9.99 |  |
|  | 1.68 | -3.22 | -9.24 | 9.93 |  |
| 0 wt% PEI + 3.5 wt% PPEGDE | 1.95 | -3.31 | -9.26 | 10.02 | 10.16 ± 0.15 |
|  | 2.11 | -3.18 | -9.40 | 10.15 |  |
|  | 2.25 | -3.13 | -9.57 | 10.31 |  |
| 0 wt% PEI + 4 wt% PPEGDE | 2.10 | -3.18 | -9.64 | 10.36 | 10.35 ± 0.04 |
|  | 2.18 | -3.21 | -9.64 | 10.39 |  |
|  | 1.96 | -3.18 | -9.61 | 10.31 |  |
| 0 wt% PEI + 4.5 wt% PPEGDE | 2.21 | -3.21 | -9.68 | 10.43 | 10.48 ± 0.06 |
|  | 2.26 | -3.17 | -9.80 | 10.54 |  |
|  | 2.12 | -3.17 | -9.75 | 10.47 |  |
| 0 wt% PEI + 5 wt% PPEGDE | 1.79 | -3.29 | -9.75 | 10.45 | 10.47 ± 0.02 |
|  | 1.96 | -3.29 | -9.74 | 10.47 |  |
|  | 2.11 | -3.31 | -9.73 | 10.49 |  |
| 2 wt% PEI + 0 wt% PPEGDE | -1.73 | -2.91 | -2.41 | 4.16 | 4.17 ± 0.02 |
|  | -1.84 | -2.84 | -2.40 | 4.15 |  |
|  | -1.51 | -2.96 | -2.56 | 4.19 |  |
| 2 wt% PEI + 0.5 wt% PPEGDE | -1.10 | -3.00 | -2.42 | 4.01 | 4.00 ± 0.01 |
|  | -1.81 | -2.53 | -2.49 | 3.99 |  |
|  | -1.29 | -2.90 | -2.41 | 3.99 |  |
| 2 wt% PEI + 1 wt% PPEGDE | -0.98 | -2.89 | -2.70 | 4.07 | 4.05 ± 0.03 |
|  | -1.32 | -2.94 | -2.39 | 4.01 |  |
|  | -1.03 | -2.81 | -2.75 | 4.06 |  |
| 2 wt% PEI + 1.5 wt% PPEGDE | -1.68 | -2.67 | -2.68 | 4.14 | 4.17 ± 0.03 |
|  | -2.32 | -2.55 | -2.36 | 4.17 |  |
|  | -1.75 | -2.72 | -2.67 | 4.20 |  |
| 2 wt% PEI + 2 wt% PPEGDE | -1.39 | -2.90 | -2.80 | 4.27 | 4.27 ± 0.01 |
|  | -0.36 | -3.04 | -3.03 | 4.31 |  |
|  | -1.47 | -2.98 | -2.62 | 4:24 |  |
| 2 wt% PEI + 2.5 wt% PPEGDE | 0.38 | -3.63 | -3.55 | 4.24 | 4.26 ± 0.05 |
|  | 0.28 | -3.82 | -3.48 | 4.25 |  |
|  | -0.25 | -3.65 | -3.57 | 4.29 |  |
| 2 wt% PEI + 3 wt% PPEGDE | -2.21 | -2.81 | -2.41 | 4.31 | 4.33 ± 0.03 |
|  | -1.97 | -3.02 | -2.47 | 4.37 |  |
|  | -2.52 | -2.61 | -2.32 | 4.31 |  |
| 2 wt% PEI + 3.5 wt % PPEGDE | -2.33 | -2.67 | -3.05 | 4.68 | 4.69 ± 0.02 |
|  | -3.16 | -2.54 | -2.40 | 4.72 |  |
|  | -2.38 | -2.66 | -3.03 | 4.68 |  |
| 2 wt% PEI + 4 wt% PPEGDE | -0.33 | -3.2 | -4.58 | 5.58 | 5.54 ± 0.05 |
|  | -0.80 | -3.16 | -4.42 | 5.49 |  |
|  | -0.18 | -3.39 | -4.40 | 5.56 |  |
| 2 wt% PEI + 4.5 wt% PPEGDE | -2.00 | -2.80 | -2.95 | 4.41 | 4.36 ± 0.03 |
|  | -1.73 | -2.66 | -3.03 | 4.38 |  |
|  | -2.27 | -2.29 | -2.91 | 4.34 |  |
| 2 wt% PEI + 5 wt% PPEGDE | -1.10 | -2.73 | -2.81 | 4.07 | 4.11 ± 0.04 |
|  | -0.36 | -2.82 | -3.02 | 4.14 |  |
|  | -1.34 | -2.83 | -2.88 | 4.12 |  |
| 4 wt% PEI + 0 wt% PPEGDE | -3.03 | -3.05 | -1.05 | 4.43 | 4.36 ± 0.06 |
|  | -2.86 | -3.09 | -1.01 | 4.33 |  |
|  | -2.86 | -3.09 | -1.03 | 4.33 |  |
| 4 wt% PEI + 0.5 wt% PPEGDE | -2.99 | -2.97 | -1.20 | 4.38 | 4.37 ± 0.01 |
|  | -2.94 | -2.92 | -1.39 | 4.37 |  |
|  | -2.27 | -3.17 | -1.97 | 4.37 |  |
| 4 wt% PEI + 1 wt% PPEGDE | -2.24 | -3.15 | -1.32 | 4.08 | 4.10 ± 0.04 |
|  | -2.33 | -3.25 | -1.10 | 4.15 |  |
|  | -1.95 | -3.19 | -1.60 | 4.07 |  |
| 4 wt% PEI + 1.5 wt% PPEGDE | -0.18 | -3.97 | -3.70 | 5.43 | 5.43 ± 0.01 |
|  | -0.21 | -3.98 | -3.68 | 5.42 |  |
|  | -1.07 | -3.53 | -3.99 | 5.44 |  |
| 4 wt% PEI + 2 wt% PPEGDE | -0.76 | -3.67 | -4.03 | 5.51 | 5.53 ± 0.02 |
|  | -0.41 | -3.70 | -4.09 | 5.53 |  |
|  | -0.4 | -3.67 | -4.15 | 5.55 |  |
| 4 wt % PEI + 2.5 wt % PPEGDE | -1.27 | -3.73 | -3.94 | 5.57 | 5.60 ± 0.04 |
|  | -1.71 | -3.62 | -3.91 | 5.60 |  |
|  | -1.04 | -3.71 | -4.12 | 5.64 |  |
| 4 wt% PEI + 3 wt% PPEGDE | -3.28 | -3.29 | -3.29 | 5.65 | 5.66 ± 0.01 |
|  | -3.18 | -3.26 | -3.35 | 5.65 |  |
|  | -3.18 | -3.26 | -3.37 | 5.67 |  |
| 4 wt% PEI + 3.5 wt% PPEGDE | -1.75 | -3.46 | -3.20 | 5.03 | 5.04 ± 0.02 |
|  | -2.30 | -3.37 | -2.99 | 5.06 |  |
|  | -1.75 | -3.48 | -3.18 | 5.03 |  |
| 4 wt% PEI + 4 wt% PPEGDE | -2.35 | -3.34 | -3.43 | 5.33 | 5.31 ± 0.02 |
|  | -2.37 | -3.48 | -3.27 | 5.30 |  |
|  | -2.54 | -3.54 | -3.01 | 5.30 |  |
| 4 wt% PEI + 4.5 wt% PPEGDE | -1.28 | -3.83 | -3.63 | 5.43 | 5.45 ± 0.02 |
|  | -1.76 | -3.88 | -3.41 | 5.46 |  |
|  | -1.73 | -3.89 | -3.41 | 5.45 |  |
| 4 wt% PEI + 5 wt% PPEGDE | -1.06 | -3.98 | -3.57 | 5.45 | 5.44 ± 0.01 |
|  | -1.25 | -4.09 | -3.36 | 5.44 |  |
|  | -1.03 | -3.69 | -3.56 | 5.44 |  |
| 6 wt% PEI + 0 wt% PPEGDE | -1.87 | -3.68 | -3.61 | 5.49 | 5.50 ± 0.01 |
|  | -1.20 | -3.78 | -3.82 | 5.51 |  |
|  | -0.92 | -3.82 | -3.85 | 5.50 |  |
| 6 wt% PEI + 0.5 wt% PPEGDE | -0.33 | -3.64 | -4.23 | 5.59 | 5.57 ± 0.02 |
|  | -0.10 | -3.70 | -4.17 | 5.57 |  |
|  | -0.40 | -3.61 | -4.21 | 5.56 |  |
| 6 wt% PEI + 1 wt% PPEGDE | -0.24 | -3.57 | -4.40 | 5.67 | 5.65 ± 0.03 |
|  | -0.38 | -3.57 | -4.36 | 5.65 |  |
|  | -0.51 | -3.50 | -4.37 | 5.62 |  |
| 6 wt%PEI + 1.5 wt% PPEGDE | -0.62 | -3.67 | -4.32 | 5.70 | 5.69 ± 0.02 |
|  | -0.59 | -3.66 | -4.33 | 5.70 |  |
|  | -0.57 | -3.64 | -4.31 | 5.67 |  |
| 6 wt% PEI + 2 wt% PPEGDE | -0.59 | -3.84 | -4.26 | 5.77 | 5.75 ± 0.02 |
|  | -1.29 | -3.50 | -4.39 | 5.76 |  |
|  | -1.28 | -3.48 | -4.37 | 5.73 |  |
| 6 wt% PEI + 2.5 wt% PPEGDE | -0.13 | -3.82 | -4.38 | 5.82 | 5.81 ± 0.01 |
|  | -0.61 | -3.77 | -4.36 | 5.80 |  |
|  | -1.30 | -3.48 | -4.49 | 5.82 |  |
| 6 wt% PEI + 3 wt% PPEGDE | -0.68 | -3.83 | -4.34 | 5.83 | 5.84 ± 0.01 |
|  | -0.85 | -3.85 | -4.33 | 5.85 |  |
|  | -1.10 | -3.83 | -4.29 | 5.85 |  |
| 6 wt% PEI + 3.5 wt% PPEGDE | -2.22 | -3.54 | -4.16 | 5.90 | 5.88 ± 0.03 |
|  | -2.69 | -3.46 | -3.88 | 5.85 |  |
|  | -1.60 | -3.63 | -4.37 | 5.90 |  |
| 6 wt% PEI + 4 wt% PPEGDE | -3.29 | -3.24 | -3.39 | 5.91 | 5.92 ± 0.04 |
|  | -3.31 | -3.18 | -3.69 | 5.89 |  |
|  | -2.50 | -3.21 | -4.36 | 5.97 |  |
| 6 wt% PEI + 4.5 wt% PPEGDE | -0.77 | -3.79 | -3.92 | 5.51 | 5.51 ± 0.01 |
|  | -0.78 | -3.82 | -3.89 | 5.51 |  |
|  | -1.29 | -3.67 | -3.88 | 5.50 |  |
| 6 wt% PEI + 5 wt% PPEGDE | -1.94 | -3.43 | -3.30 | 5.14 | 5.12 ± 0.02 |
|  | -1.27 | -3.59 | -3.42 | 5.11 |  |
|  | -1.31 | -3.61 | -3.40 | 5.12 |  |
| 8 wt% PEI + 0 wt% PPEGDE | -1.71 | -3.43 | -4.34 | 5.79 | 5.80 ± 0.01 |
|  | -1.76 | -3.47 | -4.31 | 5.81 |  |
|  | -1.68 | -3.73 | -4.43 | 5.80 |  |
| 8 wt% PEI + 0.5 wt% PPEGDE | -1.58 | -3.77 | -4.23 | 5.88 | 5.88 ± 0.02 |
|  | -1.57 | -3.64 | -4.37 | 5.90 |  |
|  | -1.58 | -3.75 | -4.24 | 5.87 |  |
| 8 wt% PEI + 1 wt% PPEGDE | -1.41 | -3.81 | -4.35 | 5.95 | 5.94 ± 0.01 |
|  | -1.42 | -3.62 | -4.51 | 5.95 |  |
|  | -1.37 | -3.35 | -4.69 | 5.93 |  |
| 8 wt% PEI + 1.5 wt% PPEGDE | -1.62 | -3.65 | -4.16 | 5.77 | 5.77 ± 0.02 |
|  | -1.89 | -3.54 | -4.16 | 5.78 |  |
|  | -1.61 | -3.67 | -4.13 | 5.75 |  |
| 8 wt% PEI + 2 wt% PPEGDE | -1.60 | -3.62 | -4.29 | 5.84 | 5.85±0.02 |
|  | -1.51 | -3.62 | -4.32 | 5.84 |  |
|  | -1.90 | -3.54 | -4.30 | 5.88 |  |
| 8 wt% PEI + 2.5 wt% PPEGDE | -1.25 | 3.96 | -4.33 | 6.00 | 6.01 ± 0.03 |
|  | -1.60 | -3.99 | -4.27 | 6.05 |  |
|  | -1.22 | -3.95 | -4.33 | 5.99 |  |
| 8 wt% PEI + 3 wt% PPEGDE | -1.77 | -3.81 | -4.65 | 6.27 | 6.26 ± 0.01 |
|  | -1.92 | -3.81 | -4.56 | 6.25 |  |
|  | -1.91 | -3.80 | -4.58 | 6.25 |  |
| 8 wt% PEI + 3.5 wt% PPEGDE | -4.34 | -3.34 | -3.11 | 6.29 | 6.28 ± 0.01 |
|  | -4.28 | -3.36 | -3.14 | 6.28 |  |
|  | -4.27 | -3.36 | -3.14 | 6.27 |  |
| 8 wt% PEI + 4 wt% PPEGDE | -1.57 | -3.77 | -4.22 | 5.88 | 5.88 ± 0.00 |
|  | -1.55 | -3.77 | -4.23 | 5.88 |  |
|  | -1.55 | -3.78 | -4.23 | 5.88 |  |
| 8 wt% PEI + 4.5 wt% PPEGDE | -0.44 | -4.11 | -4.31 | 5.97 | 5.98 ± 0.01 |
|  | -0.42 | -4.11 | -4.33 | 5.98 |  |
|  | -0.42 | -4.11 | -4.32 | 5.98 |  |
| 8 wt% PEI + 5 wt% PPEGDE | -1.89 | -3.89 | -4.22 | 6.05 | 6.04 ± 0.02 |
|  | -1.87 | -3.89 | -4.23 | 6.05 |  |
|  | -1.67 | -3.82 | -4.33 | 6.01 |  |
| 10 wt% PEI + 0 wt% PPEGDE | -2.77 | -3.28 | -3.80 | 5.73 | 5.73 ± 0.01 |
|  | -3.16 | -3.25 | -3.51 | 5.74 |  |
|  | -3.10 | -3.26 | -3.53 | 5.72 |  |
| 10 wt% PEI + 0.5 wt% PPEGDE | -3.74 | -3.32 | -3.75 | 6.25 | 6.24 ± 0.01 |
|  | -3.69 | -3.33 | -3.76 | 6.23 |  |
|  | -3.67 | -3.33 | -3.76 | 6.23 |  |
| 10 wt% PEI + 1 wt% PPEGDE | -2.70 | -3.34 | -4.09 | 5.93 | 5.92 ± 0.01 |
|  | -2.66 | -3.34 | -4.09 | 5.92 |  |
|  | -2.64 | -3.35 | -4.10 | 5.92 |  |
| 10 wt% PEI + 1.5 wt% PPEGDE | -3.27 | -3.40 | -3.60 | 5.94 | 5.93 ± 0.01 |
|  | -3.22 | -3.41 | -3.62 | 5.93 |  |
|  | -3.19 | -3.42 | -3.63 | 5.92 |  |
| 10 wt% PEI + 2 wt% PPEGDE | -3.98 | -3.20 | -3.12 | 5.99 | 5.97 ± 0.02 |
|  | -3.90 | -3.29 | -3.11 | 5.97 |  |
|  | -3.84 | -3.30 | -3.12 | 5.95 |  |
| 10 wt% PEI + 2.5 wt% PPEGDE | -3.84 | -3.18 | -3.45 | 6.07 | 6.06 ± 0.01 |
|  | -3.82 | -3.18 | -3.46 | 6.06 |  |
|  | -3.81 | -3.19 | -3.46 | 6.06 |  |
| 10 wt% PEI + 3 wt% PPEGDE | -3.98 | -3.59 | -2.84 | 6.07 | 6.06 ± 0.01 |
|  | -3.96 | -3.60 | -2.85 | 6.06 |  |
|  | -3.95 | -3.60 | -2.82 | 6.05 |  |
| 10 wt% PEI + 3.5 wt% PPEGDE | -3.60 | -3.95 | -3.27 | 6.27 | 6.24 ± 0.03 |
|  | -3.48 | -3.97 | -3.31 | 6.23 |  |
|  | -3.44 | -3.98 | -3.32 | 6.22 |  |
| 10 wt% PEI + 4 wt% PPEGDE | -4.28 | -3.43 | -3.04 | 6.27 | 6.28 ± 0.01 |
|  | -4.31 | -3.42 | -3.04 | 6.29 |  |
|  | -4.28 | -3.43 | -3.04 | 6.27 |  |
| 10 wt%PEI + 4.5 wt% PPEGDE | -3.92 | -3.73 | -3.26 | 6.32 | 6.30 ± 0.02 |
|  | -3.82 | -3.75 | -3.29 | 6.29 |  |
|  | -3.80 | -3.75 | -3.30 | 6.28 |  |
| 10 wt% PEI + 5 wt% PPEGDE | -3.81 | -4.28 | -2.65 | 6.32 | 6.30 ± 0.02 |
|  | -3.45 | -4.43 | -2.85 | 6.30 |  |
|  | -3.43 | -4.43 | -2.86 | 6.29 |  |
| 12 wt% PEI + 0 wt% PPEGDE | -3.41 | -3.09 | -3.33 | 5.68 | 5.64 ± 0.03 |
|  | -3.19 | -3.14 | -3.42 | 5.63 |  |
|  | -3.16 | -3.14 | -3.42 | 5.62 |  |
| 12 wt% PEI + 0.5 wt% PPEGDE | -3.68 | -3.11 | -3.16 | 5.76 | 5.71 ± 0.04 |
|  | -3.50 | -3.20 | -3.20 | 5.69 |  |
|  | -3.48 | -3.15 | -3.21 | 5.68 |  |
| 12 wt% PEI + 1 wt% PPEGDE | -4.11 | -3.05 | -2.83 | 5.68 | 5.71 ± 0.06 |
|  | -3.75 | -3.11 | -2.92 | 5.68 |  |
|  | -3.72 | -3.10 | -3.16 | 5.78 |  |
| 12 wt% PEI + 1.5 wt% PPEGDE | -3.04 | -3.27 | -3.55 | 5.70 | 5.73 ± 0.03 |
|  | -3.20 | -3.21 | -3.54 | 5.75 |  |
|  | -3.06 | -3.24 | -3.61 | 5.74 |  |
| 12 wt% PEI + 2 wt% PPEGDE | -3.41 | -3.16 | -3.49 | 5.81 | 5.77 ± 0.03 |
|  | -3.21 | -3.19 | -3.55 | 5.75 |  |
|  | -3.19 | -3.19 | -3.56 | 5.75 |  |
| 12 wt% PEI + 2.5 wt% PPEGDE | -3.74 | -3.34 | -2.99 | 5.84 | 5.80 ± 0.04 |
|  | -3.57 | -3.38 | -3.03 | 5.78 |  |
|  | -3.56 | -3.38 | -3.04 | 5.77 |  |
| 12 wt% PEI + 3 wt% PPEGDE | -3.49 | -2.91 | -3.60 | 5.80 | 5.80 ± 0.01 |
|  | -3.19 | -3.04 | -3.77 | 5.80 |  |
|  | -3.57 | -3.02 | -3.42 | 5.79 |  |
| 12 wt% PEI + 3.5 wt% PPEGDE | -3.20 | -3.31 | -3.78 | 5.95 | 5.92 ± 0.03 |
|  | -3.01 | -3.34 | -3.83 | 5.90 |  |
|  | -2.99 | -3.34 | -3.84 | 5.90 |  |
| 12 wt% PEI + 4 wt% PPEGDE | -2.36 | -3.12 | -4.21 | 5.74 | 5.74 ± 0.00 |
|  | -2.30 | -3.13 | -4.22 | 5.74 |  |
|  | -2.30 | -3.13 | -4.23 | 5.74 |  |
| 12% PEI + 4.5 wt% PPEGDE | -3.29 | -2.96 | -3.92 | 5.91 | 5.87 ± 0.03 |
|  | -3.09 | -3.02 | -3.95 | 5.85 |  |
|  | -3.08 | -3.02 | -3.95 | 5.85 |  |
| 12 wt% PEI + 5 wt% PPEGDE | -2.92 | -3.06 | -3.91 | 5.76 | 5.74 ± 0.02 |
|  | -2.77 | -3.09 | -3.96 | 5.73 |  |
|  | -2.76 | -3.09 | -3.96 | 5.73 |  |
| 14 wt% PEI + 0 wt% PPEGDE | -2.61 | -3.06 | -3.83 | 5.56 | 5.55 ± 0.01 |
|  | -2.58 | -3.07 | -3.84 | 5.55 |  |
|  | -2.57 | -3.07 | -3.84 | 5.55 |  |
| 14 wt% PEI + 0.5 wt% PPEGDE | -3.72 | -3.29 | -2.70 | 5.65 | 5.64 ± 0.01 |
|  | -3.70 | -3.29 | -2.70 | 5.64 |  |
|  | -3.69 | -3.29 | -2.71 | 5.64 |  |
| 14 wt% PEI + 1%PPEGDE | -3.70 | -3.16 | -3.12 | 5.78 | 5.75 ± 0.03 |
|  | -3.56 | -3.18 | -3.17 | 5.74 |  |
|  | -3.65 | -3.19 | -3.18 | 5.73 |  |
| 14 wt% PEI + 1.5 wt% PPEGDE | -4.04 | -3.10 | -2.94 | 5.88 | 5.87 ± 0.01 |
|  | -3.94 | -3.13 | -3.01 | 5.86 |  |
|  | -3.93 | -3.13 | -3.01 | 5.86 |  |
| 14 wt% PEI + 2 wt% PPEGDE | -4.06 | -2.96 | -3.15 | 5.93 | 5.89 ± 0.03 |
|  | -3.96 | -2.97 | -3.16 | 5.87 |  |
|  | -3.96 | -2.97 | -3.16 | 5.87 |  |
| 14 wt% PEI + 2.5 wt% PPEGDE | -4.29 | -3.12 | -2.71 | 5.96 | 5.92 ± 0.03 |
|  | -4.18 | -3.14 | -2.73 | 5.90 |  |
|  | -4.17 | -3.15 | -2.73 | 5.90 |  |
| 14 wt% PEI + 3 wt% PPEGDE | -4.58 | -3.14 | -2.29 | 6.01 | 5.99 ± 0.02 |
|  | -4.54 | -3.14 | -2.29 | 5.98 |  |
|  | -4.54 | -3.14 | -2.29 | 5.98 |  |
| 14 wt% PEI + 3.5 wt% PPEGDE | -4.50 | -3.17 | -2.70 | 6.13 | 6.08 ± 0.04 |
|  | -4.38 | -3.20 | -2.71 | 6.06 |  |
|  | -4.37 | -3.19 | -2.72 | 6.06 |  |
| 14 wt% PEI + 4 wt% PPEGDE | -4.40 | -3.26 | -2.77 | 6.14 | 6.13 ± 0.01 |
|  | -4.35 | -3.27 | -2.79 | 6.12 |  |
|  | -4.34 | -3.27 | -2.80 | 6.12 |  |
| 14 wt% PEI + 4.5 wt% PPEGDE | -4.76 | -3.15 | -2.27 | 6.15 | 6.08 ± 0.06 |
|  | -4.58 | -3.20 | -2.32 | 6.05 |  |
|  | -4.57 | -3.20 | -2.33 | 6.05 |  |
| 14 wt% PEI+5 wt% PPEGDE | -4.66 | -3.22 | -2.68 | 6.27 | 6.23 ± 0.03 |
|  | -4.58 | -3.24 | -2.69 | 6.22 |  |
|  | -4.57 | -3.24 | -2.69 | 6.21 |  |
| 16 wt% PEI + 0 wt% PPEGDE | -2.69 | -2.97 | -3.32 | 5.20 | 5.19 ± 0.01 |
|  | -2.59 | -2.99 | -3.36 | 5.19 |  |
|  | -2.58 | -2.99 | -3.36 | 5.18 |  |
| 16 wt% PEI + 0.5 wt% PPEGDE | -3.72 | -2.73 | -2.48 | 5.24 | 5.21 ± 0.03 |
|  | -3.62 | -2.76 | -2.52 | 5.20 |  |
|  | -3.61 | -2.76 | -2.52 | 5.19 |  |
| 16 wt% PEI + 1 wt% PPEGDE | -3.01 | -3.06 | -3.01 | 5.24 | 5.23 ± 0.01 |
|  | -3.00 | -3.06 | -3.00 | 5.23 |  |
|  | -3.00 | -3.06 | -3.00 | 5.23 |  |
| 16 wt% PEI + 1.5 wt% PPEGDE | -3.13 | -2.92 | -3.17 | 5.33 | 5.31 ± 0.02 |
|  | -3.01 | -2.95 | -3.21 | 5.30 |  |
|  | -3.01 | -2.95 | -3.21 | 5.29 |  |
| 16 wt% PEI + 2 wt% PPEGDE | -2.93 | -3.09 | -3.33 | 5.40 | 5.39 ± 0.01 |
|  | -2.88 | -3.10 | -3.34 | 5.39 |  |
|  | -2.87 | -3.10 | -3.34 | 5.38 |  |
| 16 wt% PEI + 2.5 wt% PPEGDE | -3.44 | -3.00 | -3.05 | 5.49 | 5.48 ± 0.01 |
|  | -3.40 | -3.00 | -3.07 | 5.47 |  |
|  | -3.40 | -3.01 | -3.07 | 5.47 |  |
| 16 wt% PEI + 3 wt% PPEGDE | -4.11 | -2.98 | -2.49 | 5.660 | 5.63 ± 0.05 |
|  | -4.11 | -2.98 | -2.49 | 5.66 |  |
|  | -3.95 | -3.01 | -2.53 | 5.58 |  |
| 16 wt% PEI + 3.5 wt% PPEGDE | -4.52 | -3.01 | -1.95 | 5.77 | 5.74 ± 0.03 |
|  | -4.38 | -3.05 | -2.04 | 5.72 |  |
|  | -4.38 | -3.05 | -2.05 | 5.72 |  |
| 16 wt% PEI + 4 wt% PPEGDE | -3.60 | -2.98 | -3.39 | 5.77 | 5.74 ± 0.03 |
|  | -3.99 | -2.89 | -2.94 | 5.74 |  |
|  | -3.95 | -2.90 | -2.96 | 5.72 |  |
| 16 wt% PEI + 4.5 wt% PPEGDE | -3.89 | -3.12 | -3.30 | 5.98 | 5.95 ± 0.02 |
|  | -3.80 | -3.14 | -3.32 | 5.94 |  |
|  | -3.79 | -3.14 | -3.32 | 5.94 |  |
| 16 wt% PEI + 5 wt% PPEGDE | -4.76 | -3.02 | -3.16 | 6.46 | 6.45 ± 0.01 |
|  | -4.72 | -3.02 | -3.17 | 6.44 |  |
|  | -4.71 | -3.03 | -3.17 | 6.44 |  |
| 18 wt% PEI + 0 wt% PPEGDE | -4.60 | -2.94 | -1.71 | 5.72 | 5.68 ± 0.03 |
|  | -4.50 | -2.98 | -1.72 | 5.66 |  |
|  | -4.49 | -2.98 | -1.72 | 5.66 |  |
| 18 wt% PEI + 0.5 wt% PPEGDE | -5.17 | -3.02 | -1.49 | 6.17 | 6.15 ± 0.02 |
|  | -5.13 | -3.03 | -1.49 | 6.14 |  |
|  | -5.12 | -3.04 | -1.50 | 6.14 |  |
| 18 wt% PEI+ 1 wt% PPEGDE | -5.39 | -3.02 | -0.99 | 6.25 | 6.18 ± 0.06 |
|  | -5.24 | -3.05 | -1.04 | 6.15 |  |
|  | -5.23 | -3.05 | -1.04 | 6.14 |  |
| 18 wt% PEI +1.5 wt% PPEGDE | -5.24 | -2.71 | -1.89 | 6.20 | 6.20 ± 0.02 |
|  | -5.22 | -2.68 | -1.96 | 6.19 |  |
|  | -5.13 | -2.94 | -1.93 | 6.22 |  |
| 18 wt% PEI + 2 wt% PPEGDE | -5.15 | -2.96 | -2.30 | 6.37 | 6.36 ± 0.01 |
|  | -5.12 | -2.96 | -2.30 | 6.35 |  |
|  | -5.12 | -2.96 | -2.30 | 6.35 |  |
| 18 wt% PEI + 2.5 wt% PPEGDE | -5.78 | -3.38 | 1.19 | 6.80 | 6.70 ± 0.08 |
|  | -5.60 | -3.41 | 1.14 | 6.66 |  |
|  | -5.59 | -3.41 | 1.14 | 6.65 |  |
| 18 wt% PEI + 3 wt% PPEGDE | -6.91 | -3.54 | 3.98 | 8.73 | 8.75 ± 0.02 |
|  | -6.95 | -3.54 | 3.99 | 8.76 |  |
|  | -6.93 | -3.54 | 3.98 | 8.75 |  |
| 18 wt% PEI + 3.5 wt% PPEGDE | -5.28 | -3.64 | 3.13 | 7.14 | 7.05 ± 0.08 |
|  | -5.09 | -3.68 | 3.11 | 7.01 |  |
|  | -5.08 | -3.68 | 3.11 | 6.99 |  |
| 18 wt% PEI + 4 wt% PPEGDE | -5.20 | -3.97 | 2.97 | 7.19 | 7.14 ± 0.04 |
|  | -5.11 | -3.98 | 2.96 | 7.12 |  |
|  | -5.10 | -3.98 | 2.96 | 7.12 |  |
| 18 wt% PEI + 4.5 wt% PPEGDE | -5.79 | -3.78 | 2.28 | 7.28 | 7.17 ± 0.10 |
|  | -5.57 | -3.82 | 2.23 | 7.11 |  |
|  | -5.56 | -3.82 | 2.23 | 7.11 |  |
| 18 % PEI + 5 wt% PPEGDE | -5.00 | -4.50 | 5.62 | 8.76 | 8.76 ± 0.00 |
|  | -4.99 | -4.50 | 5.62 | 8.76 |  |
|  | -4.99 | -4.50 | 5.62 | 8.76 |  |
| 20 wt% PEI + 0 wt% PPEGDE | -5.03 | -2.69 | -0.48 | 5.72 | 5.74 ± 0.02 |
|  | -5.07 | -2.69 | -0.48 | 5.76 |  |
|  | -5.04 | -2.69 | -0.48 | 5.74 |  |
| 20 wt% PEI + 0.5 wt% PPEGDE | -4.78 | -2.72 | -0.89 | 5.57 | 5.51 ± 0.05 |
|  | -4.67 | -2.74 | -0.91 | 5.49 |  |
|  | -4.66 | -2.74 | -0.91 | 5.48 |  |
| 20 wt% PEI + 1 wt% PPEGDE | -5.05 | -2.73 | -0.48 | 5.76 | 5.61 ± 0.13 |
|  | -4.74 | -2.83 | -0.54 | 5.55 |  |
|  | -4.71 | -2.84 | -0.54 | 5.52 |  |
| 20 wt% PEI + 1.5 wt% PPEGDE | -5.09 | -2.48 | -1.45 | 5.85 | 5.70 ± 0.13 |
|  | -4.79 | -2.53 | -1.54 | 5.63 |  |
|  | -4.76 | -2.53 | -1.55 | 5.61 |  |
| 20 wt% PEI + 2 wt% PPEGDE | -4.95 | -2.51 | -1.85 | 5.85 | 5.77 ± 0.07 |
|  | -4.77 | -2.55 | -1.91 | 5.74 |  |
|  | -4.75 | -2.56 | -1.92 | 5.73 |  |
| 20 wt% PEI + 2.5 wt% PPEGDE | -5.98 | -3.41 | 0.98 | 6.47 | 6.23 ± 0.06 |
|  | -5.73 | -3.43 | 1.15 | 6.21 |  |
|  | -5.67 | -3.44 | 0.85 | 6.01 |  |
| 20 wt% PEI + 3 wt% PPEGDE | -5.96 | -2.85 | 1.25 | 6.72 | 6.47 ± 0.27 |
|  | -5.71 | -2.90 | 1.19 | 6.51 |  |
|  | -5.69 | -2.90 | 1.18 | 6.19 |  |
| 20 wt% PEI+ 3.5 wt% PPEGDE | -5.49 | -2.95 | 0.58 | 6.26 | 6.10 ± 0.14 |
|  | -5.20 | -3.01 | 0.53 | 6.03 |  |
|  | -5.18 | -3.01 | 0.52 | 6.01 |  |
| 20 wt% PEI + 4 wt% PPEGDE | -5.26 | -3.25 | 0.70 | 6.22 | 6.12 ± 0.09 |
|  | -5.05 | -3.28 | 0.69 | 6.07 |  |
|  | -5.04 | -3.28 | 0.69 | 6.06 |  |
| 20 wt% PEI + 4.5 wt% PPEGDE | -5.57 | -2.79 | -0.43 | 6.24 | 6.22 ± 0.08 |
|  | -5.62 | -2.73 | -0.76 | 6.29 |  |
|  | -5.43 | -2.75 | -0.80 | 6.14 |  |
| 20 wt% PEI + 5 wt% PPEGDE | -5.69 | -2.78 | 0.05 | 6.33 | 6.31 ± 0.05 |
|  | -5.56 | -2.46 | -1.48 | 6.25 |  |
|  | -5.61 | -2.46 | -1.64 | 6.34 |  |

Fig.S123 Tensile stress-strain curves of original silk fabric (3 replicates)

Fig.S124 Tensile stress-strain curves of rotten/pulverized silk fabric (3 replicates)

Fig.S125 Tensile stress-strain curves of rotten/pulverized silk fabric treated with 0%PEI+0%PPEGDE+1% IPP (3 replicates)

Fig.S126 Tensile stress-strain curves of rotten/pulverized silk fabric treated with 0%PEI+0.5%PPEGDE+1% IPP (3 replicates)

Fig.S127 Tensile stress-strain curves of rotten/pulverized silk fabric treated with 0%PEI+1%PPEGDE+1% IPP (3 replicates)

Fig.S128 Tensile stress-strain curves of rotten/pulverized silk fabric treated with 0%PEI+1.5%PPEGDE+1% IPP (3 replicates)

Fig.S129 Tensile stress-strain curves of rotten/pulverized silk fabric treated with 0%PEI+2%PPEGDE+1% IPP (3 replicates)

Fig.S130 Tensile stress-strain curves of rotten/pulverized silk fabric treated with 0%PEI+2.5%PPEGDE+1% IPP (3 replicates)

Fig.S131 Tensile stress-strain curves of rotten/pulverized silk fabric treated with 0%PEI+3%PPEGDE+1% IPP (3 replicates)

Fig.S132 Tensile stress-strain curves of rotten/pulverized silk fabric treated with 0%PEI+3.5%PPEGDE+1% IPP (3 replicates)

Fig.S133 Tensile stress-strain curves of rotten/pulverized silk fabric treated with 0%PEI+4%PPEGDE+1% IPP (3 replicates)

Fig.S134 Tensile stress-strain curves of rotten/pulverized silk fabric treated with 0%PEI+4.5%PPEGDE+1% IPP (3 replicates)

Fig.S135 Tensile stress-strain curves of rotten/pulverized silk fabric treated with 0%PEI+5%PPEGDE+1% IPP (3 replicates)

Fig.S136 Tensile stress-strain curves of rotten/pulverized silk fabric treated with 2%PEI+0%PPEGDE+1% IPP (3 replicates)

Fig.S137 Tensile stress-strain curves of rotten/pulverized silk fabric treated with 2%PEI+0.5%PPEGDE+1% IPP (3 replicates)

Fig.S138 Tensile stress-strain curves of rotten/pulverized silk fabric treated with 2%PEI+1%PPEGDE+1% IPP (3 replicates)

Fig.S139 Tensile stress-strain curves of rotten/pulverized silk fabric treated with 2%PEI+1.5%PPEGDE+1% IPP (3 replicates)

Fig.S140 Tensile stress-strain curves of rotten/pulverized silk fabric treated with 2%PEI+2%PPEGDE+1% IPP (3 replicates)

Fig.S141 Tensile stress-strain curves of rotten/pulverized silk fabric treated with 2%PEI+2.5%PPEGDE+1% IPP (3 replicates)

Fig.S142 Tensile stress-strain curves of rotten/pulverized silk fabric treated with 2%PEI+3%PPEGDE+1% IPP (3 replicates)

Fig.S143 Tensile stress-strain curves of rotten/pulverized silk fabric treated with 2%PEI+3.5%PPEGDE+1% IPP (3 replicates)

Fig.S144 Tensile stress-strain curves of rotten/pulverized silk fabric treated with 2%PEI+4%PPEGDE+1% IPP (3 replicates)

Fig.S145 Tensile stress-strain curves of rotten/pulverized silk fabric treated with 2%PEI+4.5%PPEGDE+1% IPP (3 replicates)

Fig.S146 Tensile stress-strain curves of rotten/pulverized silk fabric treated with 2%PEI+5%PPEGDE+1% IPP (3 replicates)

Fig.S147 Tensile stress-strain curves of rotten/pulverized silk fabric treated with 4%PEI+0%PPEGDE+1% IPP (3 replicates)

Fig.S148 Tensile stress-strain curves of rotten/pulverized silk fabric treated with 4%PEI+0.5%PPEGDE+1% IPP (3 replicates)

Fig.S149 Tensile stress-strain curves of rotten/pulverized silk fabric treated with 4%PEI+1%PPEGDE+1% IPP (3 replicates)

Fig.S150 Tensile stress-strain curves of rotten/pulverized silk fabric treated with 4%PEI+1.5%PPEGDE+1% IPP (3 replicates)

Fig.S151 Tensile stress-strain curves of rotten/pulverized silk fabric treated with 4%PEI+2%PPEGDE+1% IPP (3 replicates)

Fig.S152 Tensile stress-strain curves of rotten/pulverized silk fabric treated with 4%PEI+2.5%PPEGDE+1% IPP (3 replicates)

Fig.S153 Tensile stress-strain curves of rotten/pulverized silk fabric treated with 4%PEI+3%PPEGDE+1% IPP (3 replicates)

Fig.S154 Tensile stress-strain curves of rotten/pulverized silk fabric treated with 4%PEI+3.5%PPEGDE+1% IPP (3 replicates)

Fig.S155 Tensile stress-strain curves of rotten/pulverized silk fabric treated with 4%PEI+4%PPEGDE+1% IPP (3 replicates)

Fig.S156 Tensile stress-strain curves of rotten/pulverized silk fabric treated with 4%PEI+4.5%PPEGDE+1% IPP (3 replicates)

Fig.S157 Tensile stress-strain curves of rotten/pulverized silk fabric treated with 4%PEI+5%PPEGDE+1% IPP (3 replicates)

Fig.S158 Tensile stress-strain curves of rotten/pulverized silk fabric treated with 6%PEI+0%PPEGDE+1% IPP (3 replicates)

Fig.S159 Tensile stress-strain curves of rotten/pulverized silk fabric treated with 6%PEI+0.5%PPEGDE+1% IPP (3 replicates)

Fig.S160 Tensile stress-strain curves of rotten/pulverized silk fabric treated with 6%PEI+1%PPEGDE+1% IPP (3 replicates)

Fig.S161 Tensile stress-strain curves of rotten/pulverized silk fabric treated with 6%PEI+1.5%PPEGDE+1% IPP (3 replicates)

Fig.S162 Tensile stress-strain curves of rotten/pulverized silk fabric treated with 6%PEI+2%PPEGDE+1% IPP (3 replicates)

Fig.S163 Tensile stress-strain curves of rotten/pulverized silk fabric treated with 6%PEI+2.5%PPEGDE+1% IPP (3 replicates)

Fig.S164 Tensile stress-strain curves of rotten/pulverized silk fabric treated with 6%PEI+3%PPEGDE+1% IPP (3 replicates)

Fig.S165 Tensile stress-strain curves of rotten/pulverized silk fabric treated with 6%PEI+3.5%PPEGDE+1% IPP (3 replicates)

Fig.S166 Tensile stress-strain curves of rotten/pulverized silk fabric treated with 6%PEI+4%PPEGDE+1% IPP (3 replicates)

Fig.S167 Tensile stress-strain curves of rotten/pulverized silk fabric treated with 6%PEI+4.5%PPEGDE+1% IPP (3 replicates)

Fig.S168 Tensile stress-strain curves of rotten/pulverized silk fabric treated with 6%PEI+5%PPEGDE+1% IPP (3 replicates)

Fig.S169 Tensile stress-strain curves of rotten/pulverized silk fabric treated with 8%PEI+0%PPEGDE+1% IPP (3 replicates)

Fig.S170 Tensile stress-strain curves of rotten/pulverized silk fabric treated with 8%PEI+0.5%PPEGDE+1% IPP (3 replicates)

Fig.S171 Tensile stress-strain curves of rotten/pulverized silk fabric treated with 8%PEI+1%PPEGDE+1% IPP (3 replicates)

Fig.S172 Tensile stress-strain curves of rotten/pulverized silk fabric treated with 8%PEI+1.5%PPEGDE+1% IPP (3 replicates)

Fig.S173 Tensile stress-strain curves of rotten/pulverized silk fabric treated with 8%PEI+2%PPEGDE+1% IPP (3 replicates)

Fig.S174 Tensile stress-strain curves of rotten/pulverized silk fabric treated with 8%PEI+2.5%PPEGDE+1% IPP (3 replicates)

FigS.175 Tensile stress-strain curves of rotten/pulverized silk fabric treated with 8%PEI+3%PPEGDE+1% IPP (3 replicates)

Fig.S176 Tensile stress-strain curves of rotten/pulverized silk fabric treated with 8%PEI+3.5%PPEGDE+1% IPP (3 replicates)

Fig.S177 Tensile stress-strain curves of rotten/pulverized silk fabric treated with 8%PEI+4%PPEGDE+1% IPP (3 replicates)

Fig.S178 Tensile stress-strain curves of rotten/pulverized silk fabric treated with 8%PEI+4.5%PPEGDE+1% IPP (3 replicates)

Fig.S179 Tensile stress-strain curves of rotten/pulverized silk fabric treated with 8%PEI+5%PPEGDE+1% IPP (3 replicates)

Fig.S180 Tensile stress-strain curves of rotten/pulverized silk fabric treated with 10%PEI+0%PPEGDE+1% IPP (3 replicates)

Fig.S181 Tensile stress-strain curves of rotten/pulverized silk fabric treated with 10%PEI+0.5%PPEGDE+1% IPP (3 replicates)

Fig.S182 Tensile stress-strain curves of rotten/pulverized silk fabric treated with 10%PEI+1%PPEGDE+1% IPP (3 replicates)

Fig.S183 Tensile stress-strain curves of rotten/pulverized silk fabric treated with 10%PEI+1.5%PPEGDE+1% IPP (3 replicates)

Fig.S184 Tensile stress-strain curves of rotten/pulverized silk fabric treated with 10%PEI+2%PPEGDE+1% IPP (3 replicates)

Fig.S185 Tensile stress-strain curves of rotten/pulverized silk fabric treated with 10%PEI+2.5%PPEGDE+1% IPP (3 replicates)

Fig.S186 Tensile stress-strain curves of rotten/pulverized silk fabric treated with 10%PEI+3%PPEGDE+1% IPP (3 replicates)

Fig.S187 Tensile stress-strain curves of rotten/pulverized silk fabric treated with 10%PEI+3.5%PPEGDE+1% IPP (3 replicates)

Fig.S188 Tensile stress-strain curves of rotten/pulverized silk fabric treated with 10%PEI+4%PPEGDE+1% IPP (3 replicates)

Fig.S189 Tensile stress-strain curves of rotten/pulverized silk fabric treated with 10%PEI+4.5%PPEGDE+1% IPP (3 replicates)

Fig.S190 Tensile stress-strain curves of rotten/pulverized silk fabric treated with 10%PEI+5%PPEGDE+1% IPP (3 replicates)

Fig.S191 Tensile stress-strain curves of rotten/pulverized silk fabric treated with 12%PEI+0%PPEGDE+1% IPP (3 replicates)

Fig.S192 Tensile stress-strain curves of rotten/pulverized silk fabric treated with 12%PEI+0.5%PPEGDE+1% IPP (3 replicates)

Fig.S193 Tensile stress-strain curves of rotten/pulverized silk fabric treated with 12%PEI+1%PPEGDE+1% IPP (3 replicates)

Fig.S194 Tensile stress-strain curves of rotten/pulverized silk fabric treated with 12%PEI+1.5%PPEGDE+1% IPP (3 replicates)

Fig.S195 Tensile stress-strain curves of rotten/pulverized silk fabric treated with 12%PEI+2%PPEGDE+1% IPP (3 replicates)

Fig.S196 Tensile stress-strain curves of rotten/pulverized silk fabric treated with 12%PEI+2.5%PPEGDE+1% IPP (3 replicates)

Fig.S197 Tensile stress-strain curves of rotten/pulverized silk fabric treated with 12%PEI+3%PPEGDE+1% IPP (3 replicates)

Fig.S198 Tensile stress-strain curves of rotten/pulverized silk fabric treated with 12%PEI+3.5%PPEGDE+1% IPP (3 replicates)

Fig.S199 Tensile stress-strain curves of rotten/pulverized silk fabric treated with 12%PEI+4%PPEGDE+1% IPP (3 replicates)

Fig.S200 Tensile stress-strain curves of rotten/pulverized silk fabric treated with 12%PEI+4.5%PPEGDE+1% IPP (3 replicates)

Fig.S201 Tensile stress-strain curves of rotten/pulverized silk fabric treated with 12%PEI+5%PPEGDE+1% IPP (3 replicates)

Fig.S202 Tensile stress-strain curves of rotten/pulverized silk fabric treated with 14%PEI+0%PPEGDE+1% IPP (3 replicates)

Fig.S203 Tensile stress-strain curves of rotten/pulverized silk fabric treated with 14%PEI+0.5%PPEGDE+1% IPP (3 replicates)

Fig.S204 Tensile stress-strain curves of rotten/pulverized silk fabric treated with 14%PEI+1%PPEGDE+1% IPP (3 replicates)

Fig.S205 Tensile stress-strain curves of rotten/pulverized silk fabric treated with 14%PEI+1.5%PPEGDE+1% IPP (3 replicates)

Fig.S206 Tensile stress-strain curves of rotten/pulverized silk fabric treated with 14%PEI+2%PPEGDE+1% IPP (3 replicates)

Fig.S207 Tensile stress-strain curves of rotten/pulverized silk fabric treated with 14%PEI+2.5%PPEGDE+1% IPP (3 replicates)

Fig.S208 Tensile stress-strain curves of rotten/pulverized silk fabric treated with 14%PEI+3%PPEGDE+1% IPP (3 replicates)

Fig.S209 Tensile stress-strain curves of rotten/pulverized silk fabric treated with 14%PEI+3.5%PPEGDE+1% IPP (3 replicates)

Fig.S210 Tensile stress-strain curves of rotten/pulverized silk fabric treated with 14%PEI+4%PPEGDE+1% IPP (3 replicates)

Fig.S211 Tensile stress-strain curves of rotten/pulverized silk fabric treated with 14%PEI+4.5%PPEGDE+1% IPP (3 replicates)

Fig.S212 Tensile stress-strain curves of rotten/pulverized silk fabric treated with 14%PEI+5%PPEGDE+1% IPP (3 replicates)

Fig.S213 Tensile stress-strain curves of rotten/pulverized silk fabric treated with 16%PEI+0%PPEGDE+1% IPP (3 replicates)

Fig.S214 Tensile stress-strain curves of rotten/pulverized silk fabric treated with 16%PEI+0.5%PPEGDE+1% IPP (3 replicates)

Fig.S215 Tensile stress-strain curves of rotten/pulverized silk fabric treated with 16%PEI+1%PPEGDE+1% IPP (3 replicates)

Fig.S216 Tensile stress-strain curves of rotten/pulverized silk fabric treated with 16%PEI+1.5%PPEGDE+1% IPP (3 replicates)

Fig.S217 Tensile stress-strain curves of rotten/pulverized silk fabric treated with 16%PEI+2%PPEGDE+1% IPP (3 replicates)

Fig.S218 Tensile stress-strain curves of rotten/pulverized silk fabric treated with 16%PEI+2.5%PPEGDE+1% IPP (3 replicates)

Fig.S219 Tensile stress-strain curves of rotten/pulverized silk fabric treated with 16%PEI+3%PPEGDE+1% IPP (3 replicates)

Fig.S220 Tensile stress-strain curves of rotten/pulverized silk fabric treated with 16%PEI+3.5%PPEGDE+1% IPP (3 replicates)

Fig.S221 Tensile stress-strain curves of rotten/pulverized silk fabric treated with 16%PEI+4%PPEGDE+1% IPP (3 replicates)

Fig.S222 Tensile stress-strain curves of rotten/pulverized silk fabric treated with 16%PEI+4.5%PPEGDE+1% IPP (3 replicates)

Fig.S223 Tensile stress-strain curves of rotten/pulverized silk fabric treated with 16%PEI+5%PPEGDE+1% IPP (3 replicates)

Fig.S224 Tensile stress-strain curves of rotten/pulverized silk fabric treated with 18%PEI+0%PPEGDE+1% IPP (3 replicates)

Fig.S225 Tensile stress-strain curves of rotten/pulverized silk fabric treated with 18%PEI+0.5%PPEGDE+1% IPP (3 replicates)

Fig.S226 Tensile stress-strain curves of rotten/pulverized silk fabric treated with 18%PEI+1%PPEGDE+1% IPP (3 replicates)

Fig.S227 Tensile stress-strain curves of rotten/pulverized silk fabric treated with 18%PEI+1.5%PPEGDE+1% IPP (3 replicates)

Fig.S228 Tensile stress-strain curves of rotten/pulverized silk fabric treated with 18%PEI+2%PPEGDE+1% IPP (3 replicates)

Fig.S229 Tensile stress-strain curves of rotten/pulverized silk fabric treated with 18%PEI+2.5%PPEGDE+1% IPP (3 replicates)

Fig.S230 Tensile stress-strain curves of rotten/pulverized silk fabric treated with 18%PEI+3%PPEGDE+1% IPP (3 replicates)

Fig.S231 Tensile stress-strain curves of rotten/pulverized silk fabric treated with 18%PEI+3.5%PPEGDE+1% IPP (3 replicates)

Fig.S232 Tensile stress-strain curves of rotten/pulverized silk fabric treated with 18%PEI+4%PPEGDE+1% IPP (3 replicates)

Fig.S233 Tensile stress-strain curves of rotten/pulverized silk fabric treated with 18%PEI+4.5%PPEGDE+1% IPP (3 replicates)

Fig.S234 Tensile stress-strain curves of rotten/pulverized silk fabric treated with 18%PEI+5%PPEGDE+1% IPP (3 replicates)

Fig.S234 Tensile stress-strain curves of rotten/pulverized silk fabric treated with 20%PEI+0%PPEGDE+1% IPP (3 replicates)

Fig.S235 Tensile stress-strain curves of rotten/pulverized silk fabric treated with 20%PEI+0.5%PPEGDE+1% IPP (3 replicates)

Fig.S236 Tensile stress-strain curves of rotten/pulverized silk fabric treated with 20%PEI+1%PPEGDE+1% IPP (3 replicates)

Fig.S237 Tensile stress-strain curves of rotten/pulverized silk fabric treated with 20%PEI+1.5%PPEGDE+1% IPP (3 replicates)

Fig.S238 Tensile stress-strain curves of rotten/pulverized silk fabric treated with 20%PEI+2%PPEGDE+1% IPP (3 replicates)

Fig.S239 Tensile stress-strain curves of rotten/pulverized silk fabric treated with 20%PEI+2.5%PPEGDE+1% IPP (3 replicates)

Fig.S240 Tensile stress-strain curves of rotten/pulverized silk fabric treated with 20%PEI+3%PPEGDE+1% IPP (3 replicates)

Fig.S241 Tensile stress-strain curves of rotten/pulverized silk fabric treated with 20%PEI+3.5%PPEGDE+1% IPP (3 replicates)

Fig.S242 Tensile stress-strain curves of rotten/pulverized silk fabric treated with 20%PEI+4%PPEGDE+1% IPP (3 replicates)

Fig.S243 Tensile stress-strain curves of rotten/pulverized silk fabric treated with 20%PEI+4.5%PPEGDE+1% IPP (3 replicates)

Fig.S244 Tensile stress-strain curves of rotten/pulverized silk fabric treated with 20%PEI+5%PPEGDE+1% IPP (3 replicates)

Table.S3 Mechanical properties of simulated rotten/crushed silk fabrics and original silk fabrics with different treatments

| Sample | Fracture strength（MPa） | Fracture estrain（mm/mm) | Fracture energy（KJ/m^2^） | Young's modulus（MPa） |
| --- | --- | --- | --- | --- |
| Original silk fabric | 34.08 ± 0.93 | 0.19 ± 0.03 | 82.41 ± 11.89 | 3.74 ± 0.08 |
|  |  |  |  |  |
|  |  |  |  |  |
| Rotten/pulverized silk fabric | 0.15 ± 0.01 | 0.04 ± 0.00 | 0.08 ± 0.01 | 2.98 ± 0.40 |
|  |  |  |  |  |
|  |  |  |  |  |
| 0%PEI+0%PEGDE | 0.14 ± 0.01 | 0.09 ± 0.01 | 0.40 ± 0.17 | 1.39 ± 0.03 |
|  |  |  |  |  |
|  |  |  |  |  |
| 0%PEI+0.5%PEGDE | 0.22 ± 0.01 | 0.11 ± 0.01 | 0.38 ± 0.05 | 1.43 ± 0.21 |
|  |  |  |  |  |
|  |  |  |  |  |
| 0%PEI+1%PEGDE | 0.42 ± 0.05 | 0.15 ± 0.01 | 1.01 ± 0.20 | 2.72 ± 0.24 |
|  |  |  |  |  |
|  |  |  |  |  |
| 0%PEI+1.5%PEGDE | 0.54 ± 0.06 | 0.19 ± 0.02 | 1.57 ± 0.37 | 2.40 ± 0.40 |
|  |  |  |  |  |
|  |  |  |  |  |
| 0%PEI+2%PEGDE | 0.60 ± 0.03 | 0.17 ± 0.01 | 1.50 ± 0.09 | 3.37 ± 0.66 |
|  |  |  |  |  |
|  |  |  |  |  |
| 0%PEI+2.5%PEGDE | 0.52 ± 0.03 | 0.20 ± 0.01 | 1.58 ± 0.22 | 2.20 ± 0.60 |
|  |  |  |  |  |
|  |  |  |  |  |
| 0%PEI+3%PEGDE | 0.51 ± 0.08 | 0.22 ± 0.01 | 1.44 ± 0.21 | 1.66 ± 0.05 |
|  |  |  |  |  |
|  |  |  |  |  |
| 0%PEI+3.5%PEGDE | 0.43 ± 0.01 | 0.21 ± 0.01 | 1.29 ± 0.07 | 1.53 ± 0.23 |
|  |  |  |  |  |
|  |  |  |  |  |
| 0%PEI+4%PEGDE | 1.40 ± 0.13 | 0.20 ± 0.00 | 0.90 ± 0.15 | 4.01 ± 0.45 |
|  |  |  |  |  |
|  |  |  |  |  |
| 0%PEI+4.5%PEGDE | 0.46 ± 0.03 | 0.19 ± 0.03 | 1.35 ± 0.37 | 1.92 ± 0.15 |
|  |  |  |  |  |
|  |  |  |  |  |
| 0%PEI+5%PEGDE | 0.49 ± 0.01 | 0.23 ± 0.01 | 1.46 ± 0.07 | 1.56 ± 0.07 |
|  |  |  |  |  |
|  |  |  |  |  |
| 2%PEI+0%PEGDE | 0.15 ± 0.00 | 0.09 ± 0.00 | 0.24 ± 0.01 | 2.10 ± 0.30 |
|  |  |  |  |  |
|  |  |  |  |  |
| 2%PEI+0.5%PEGDE | 0.29 ± 0.01 | 0.22 ± 0.08 | 1.70 ± 0.17 | 0.26 ± 0.04 |
|  |  |  |  |  |
|  |  |  |  |  |
| 2%PEI+1%PEGDE | 0.33 ± 0.02 | 0.29· ± 0.08 | 2.17 ± 0.99 | 1.66 ± 0.13 |
|  |  |  |  |  |
|  |  |  |  |  |
| 2%PEI+1.5%PEGDE | 0.47 ± 0.06 | 0.30 ± 0.08 | 2.01 ± 0.86 | 1.49 ± 0.03 |
|  |  |  |  |  |
|  |  |  |  |  |
| 2%PEI+2%PEGDE | 1.01 ± 0.03 | 0.29 ± 0.02 | 3.84 ± 0.66 | 2.92 ± 0.25 |
|  |  |  |  |  |
|  |  |  |  |  |
| 2%PEI+2.5%PEGDE | 1.23 ± 0.01 | 0.28 ± 0.03 | 4.06 ± 0.36 | 2.65 ± 0.55 |
|  |  |  |  |  |
|  |  |  |  |  |
| 2%PEI+3%PEGDE | 1.40 ± 0.14 | 0.32 ± 0.03 | 4.45 ± 0.54 | 3.37 ± 0.01 |
|  |  |  |  |  |
|  |  |  |  |  |
| 2%PEI+3.5%PEGDE | 1.20 ± 0.06 | 0.30 ± 0.03 | 4.63 ± 0.54 | 3.61 ± 0.17 |
|  |  |  |  |  |
|  |  |  |  |  |
| 2%PEI+4%PEGDE | 1.50 ± 0.13 | 0.29 ± 0.01 | 3.94 ± 0.55 | 3.91 ± 0.10 |
|  |  |  |  |  |
|  |  |  |  |  |
| 2%PEI+4.5%PEGDE | 1.10 ± 0.08 | 0.26 ± 0.01 | 4.01 ± 0.39 | 2.90 ± 0.34 |
|  |  |  |  |  |
|  |  |  |  |  |
| 2%PEI+5%PEGDE | 1.09 ± 0.09 | 0.27 ± 0.02 | 3.74 ± 0.20 | 3.05 ± 0.15 |
|  |  |  |  |  |
|  |  |  |  |  |
| 4%PEI+0%PEGDE | 0.21 ± 0.01 | 0.05 ± 0.01 | 0.15 ± 0.02 | 2.78 ± 0.12 |
|  |  |  |  |  |
|  |  |  |  |  |
| 4%PEI+0.5%PEGDE | 0.23 ± 0.00 | 0.06 ± 0.01 | 0.21 ± 0.04 | 1.71 ± 0.17 |
|  |  |  |  |  |
|  |  |  |  |  |
| 4%PEI+1%PEGDE | 0.46 ± 0.06 | 0.10 ± 0.02 | 0.57 ± 0.08 | 2.54 ± 0.24 |
|  |  |  |  |  |
|  |  |  |  |  |
| 4%PEI+1.5%PEGDE | 0.76 ± 0.15 | 0.13 ± 0.02 | 0.95 ± 0.16 | 0.70 ± 0.17 |
|  |  |  |  |  |
|  |  |  |  |  |
| 4%PEI+2%PEGDE | 0.95 ± 0.05 | 0.12 ± 0.01 | 1.27 ± 0.18 | 1.35 ± 0.28 |
|  |  |  |  |  |
|  |  |  |  |  |
| 4%PEI+2.5%PEGDE | 0.72 ± 0.07 | 0.11 ± 0.02 | 0.90 ± 0.31 | 3.16 ± 0.08 |
|  |  |  |  |  |
|  |  |  |  |  |
| 4%PEI+3%PEGDE | 0.79 ± 0.04 | 0.12 ± 0.01 | 1.16 ± 0.13 | 2.32 ± 0.03 |
|  |  |  |  |  |
|  |  |  |  |  |
| 4%PEI+3.5%PEGDE | 1.32 ± 0.02 | 0.10 ± 0.00 | 1.50 ± 0.22 | 4.98 ± 0.22 |
|  |  |  |  |  |
|  |  |  |  |  |
| 4%PEI+4%PEGDE | 1.55 ± 0.05 | 0.11 ± 0.01 | 1.90 ± 0.12 | 3.87 ± 0.30 |
|  |  |  |  |  |
|  |  |  |  |  |
| 4%PEI+4.5%PEGDE | 2.03 ± 0.26 | 0.14 ± 0.03 | 2.80 ± 0.16 | 4.34 ± 0.07 |
|  |  |  |  |  |
|  |  |  |  |  |
| 4%PEI+5%PEGDE | 2.22 ± 0.10 | 0.12 ± 0.01 | 2.89 ± 0.49 | 4.87 ± 0.07 |
|  |  |  |  |  |
|  |  |  |  |  |
| 6%PEI+0%PEGDE | 0.17 ± 0.00 | 0.02 ± 0.00 | 0.08 ± 0.01 | 23.02 ± 0.80 |
|  |  |  |  |  |
|  |  |  |  |  |
| 6%PEI+0.5%PEGDE | 0.22 ± 0.01 | 0.13 ± 0.01 | 0.44 ± 0.03 | 4.08 ± 0.31 |
|  |  |  |  |  |
|  |  |  |  |  |
| 6%PEI+1%PEGDE | 0.24 ± 0.01 | 0.08 ± 0.02 | 0.30 ± 0.07 | 2.45 ± 0.38 |
|  |  |  |  |  |
|  |  |  |  |  |
| 6%PEI+1.5%PEGDE | 0.19 ± 0.01 | 0.05 ± 0.02 | 0.17 ± 0.07 | 4.89 ± 0.22 |
|  |  |  |  |  |
|  |  |  |  |  |
| 6%PEI+2%PEGDE | 0.39 ± 0.02 | 0.05 ± 0.00 | 0.29 ± 0.06 | 6.15 ± 0.13 |
|  |  |  |  |  |
|  |  |  |  |  |
| 6%PEI+2.5%PEGDE | 0.45 ± 0.04 | 0.08 ± 0.01 | 0.56 ± 0.15 | 7.63 ± 0.18 |
|  |  |  |  |  |
|  |  |  |  |  |
| 6%PEI+3%PEGDE | 0.81 ± 0.10 | 0.03 ± 0.00 | 0.36 ± 0.01 | 7.81 ± 0.09 |
|  |  |  |  |  |
|  |  |  |  |  |
| 6%PEI+3.5%PEGDE | 0.37 ± 0.03 | 0.10 ± 0.03 | 0.59 ± 0.22 | 6.09 ± 0.15 |
|  |  |  |  |  |
|  |  |  |  |  |
| 6%PEI+4%PEGDE | 0.43 ± 0.05 | 0.09 ± 0.02 | 0.64 ± 0.27 | 5.07 ± 0.59 |
|  |  |  |  |  |
|  |  |  |  |  |
| 6%PEI+4.5%PEGDE | 0.76 ± 0.06 | 0.19 ± 0.02 | 2.38 ± 0.41 | 5.26 ± 0.35 |
|  |  |  |  |  |
|  |  |  |  |  |
| 6%PEI+5%PEGDE | 0.49 ± 0.04 | 0.12 ± 0.03 | 1.05 ± 0.36 | 5.66 ± 0.20 |
|  |  |  |  |  |
|  |  |  |  |  |
| 8%PEI+0%PEGDE | 0.20 ± 0.01 | 0.03 ± 0.01 | 0.09 ± 0.03 | 2.90 ± 0.19 |
|  |  |  |  |  |
|  |  |  |  |  |
| 8%PEI+0.5%PEGDE | 0.25 ± 0.01 | 0.19 ± 0.01 | 0.83 ± 0.05 | 1.65 ± 0.08 |
|  |  |  |  |  |
|  |  |  |  |  |
| 8%PEI+1%PEGDE | 0.18 ± 0.01 | 0.13 ± 0.02 | 0.42 ± 0.05 | 2.32 ± 0.25 |
|  |  |  |  |  |
|  |  |  |  |  |
| 8%PEI+1.5%PEGDE | 0.58 ± 0.04 | 0.10 ± 0.01 | 1.09 ± 0.15 | 8.26 ± 0.24 |
|  |  |  |  |  |
|  |  |  |  |  |
| 8%PEI+2%PEGDE | 0.59 ± 0.02 | 0.03 ± 0.00 | 0.29 ± 0.00 | 14.78 ± 4.68 |
|  |  |  |  |  |
|  |  |  |  |  |
| 8%PEI+2.5%PEGDE | 0.48 ± 0.02 | 0.07 ± 0.00 | 0.57 ± 0.08 | 8.71 ± 0.59 |
|  |  |  |  |  |
|  |  |  |  |  |
| 8%PEI+3%PEGDE | 0.62 ± 0.04 | 0.10 ± 0.01 | 1.03 ± 0.30 | 17.48 ± 1.20 |
|  |  |  |  |  |
|  |  |  |  |  |
| 8%PEI+3.5%PEGDE | 1.54 ± 0.11 | 0.03 ± 0.00 | 0.44 ± 0.10 | 26.28 ± 0.16 |
|  |  |  |  |  |
|  |  |  |  |  |
| 8%PEI+4%PEGDE | 0.69 ± 0.02 | 0.08 ± 0.00 | 1.01 ± 0.07 | 11.53 ± 0.50 |
|  |  |  |  |  |
|  |  |  |  |  |
| 8%PEI+4.5%PEGDE | 0.68 ± 0.06 | 0.11 ± 0.02 | 1.27 ± 0.31 | 9.45 ± 0.24 |
|  |  |  |  |  |
|  |  |  |  |  |
| 8%PEI+5%PEGDE | 0.79 ± 0.05 | 0.11 ± 0.01 | 1.49 ± 0.12 | 9.14 ± 0.53 |
|  |  |  |  |  |
|  |  |  |  |  |
| 10%PEI+0%PEGDE | 0.20 ± 0.01 | 0.03 ± 0.01 | 0.09 ± 0.03 | 2.90 ± 0.19 |
|  |  |  |  |  |
|  |  |  |  |  |
| 10%PEI+0.5%PEGDE | 0.56 ± 0.03 | 0.05 ± 0.01 | 0.34 ± 0.08 | 10.96 ± 0.73 |
|  |  |  |  |  |
|  |  |  |  |  |
| 10%PEI+1%PEGDE | 0.98 ± 0.02 | 0.04 ± 0.01 | 0.48 ± 0.09 | 12.76 ± 0.15 |
|  |  |  |  |  |
|  |  |  |  |  |
| 10%PEI+1.5%PEGDE | 0.74 ± 0.02 | 0.04 ± 0.01 | 0.39 ± 0.22 | 12.86 ± 0.12 |
|  |  |  |  |  |
|  |  |  |  |  |
| 10%PEI+2%PEGDE | 1.29 ± 0.06 | 0.05 ± 0.00 | 0.92 ± 0.09 | 16.64 ± 0.29 |
|  |  |  |  |  |
|  |  |  |  |  |
| 10%PEI+2.5%PEGDE | 1.47 ± 0.06 | 0.05 ± 0.00 | 1.20 ± 0.03 | 14.41 ± 0.15 |
|  |  |  |  |  |
|  |  |  |  |  |
| 10%PEI+3%PEGDE | 1.24 ± 0.07 | 0.05 ± 0.02 | 0.96 ± 0.21 | 15.38 ± 0.13 |
|  |  |  |  |  |
|  |  |  |  |  |
| 10%PEI+3.5%PEGDE | 1.20 ± 0.12 | 0.04 ± 0.00 | 0.60 ± 0.12 | 5.03 ± 0.97 |
|  |  |  |  |  |
|  |  |  |  |  |
| 10%PEI+4%PEGDE | 1.54 ± 0.16 | 0.05 ± 0.00 | 1.08 ± 0.10 | 10.76 ± 0.14 |
|  |  |  |  |  |
|  |  |  |  |  |
| 10%PEI+4.5%PEGDE | 1.29 ± 0.06 | 0.06 ± 0.01 | 0.92 ± 0.21 | 10.22 ± 0.83 |
|  |  |  |  |  |
|  |  |  |  |  |
| 10%PEI+5%PEGDE | 1.74 ± 0.06 | 0.07 ± 0.00 | 1.57 ± 0.20 | 11.20 ± 0.30 |
|  |  |  |  |  |
|  |  |  |  |  |
| 12%PEI+0%PEGDE | 1.14 ± 0.09 | 0.08 ± 0.02 | 1.26 ± 0.35 | 10.86 ± 1.49 |
|  |  |  |  |  |
|  |  |  |  |  |
| 12%PEI+0.5%PEGDE | 1.22 ± 0.12 | 0.09 ± 0.01 | 1.43 ± 0.30 | 9.34 ± 0.19 |
|  |  |  |  |  |
|  |  |  |  |  |
| 12%PEI+1%PEGDE | 1.46 ± 0.08 | 0.10 ± 0.01 | 1.66 ± 0.21 | 7.98 ± 0.88 |
|  |  |  |  |  |
|  |  |  |  |  |
| 12%PEI+1.5%PEGDE | 1.40 ± 0.05 | 0.10 ± 0.01 | 1.65 ± 0.30 | 8.64 ± 0.24 |
|  |  |  |  |  |
|  |  |  |  |  |
| 12%PEI+2%PEGDE | 1.60 ± 0.06 | 0.09 ± 0.00 | 1.49 ± 0.29 | 9.60 ± 0.26 |
|  |  |  |  |  |
|  |  |  |  |  |
| 12%PEI+2.5%PEGDE | 1.49 ± 0.02 | 0.09 ± 0.01 | 1.53 ± 0.18 | 10.43 ± 0.22 |
|  |  |  |  |  |
|  |  |  |  |  |
| 12%PEI+3%PEGDE | 1.52 ± 0.03 | 0.08 ± 0.00 | 1.58 ± 0.14 | 11.43 ± 0.22 |
|  |  |  |  |  |
|  |  |  |  |  |
| 12%PEI+3.5%PEGDE | 1.69 ± 0.09 | 0.07 ± 0.01 | 1.78 ± 0.09 | 17.67 ± 0.29 |
|  |  |  |  |  |
|  |  |  |  |  |
| 12%PEI+4%PEGDE | 1.81 ± 0.07 | 0.07 ± 0.01 | 1.94 ± 0.25 | 18.84 ± 0.83 |
|  |  |  |  |  |
|  |  |  |  |  |
| 12%PEI+4.5%PEGDE | 2.67 ± 0.11 | 0.06 ± 0.00 | 2.23 ± 0.52 | 20.33 ± 0.28 |
|  |  |  |  |  |
|  |  |  |  |  |
| 12%PEI+5%PEGDE | 2.25 ± 0.02 | 0.04 ± 0.01 | 1.23 ± 0.11 | 23.40 ± 0.33 |
|  |  |  |  |  |
|  |  |  |  |  |
| 14%PEI+0%PEGDE | 1.41 ± 0.06 | 0.06 ± 0.01 | 1.13 ± 0.27 | 12.61 ± 0.33 |
|  |  |  |  |  |
|  |  |  |  |  |
| 14%PEI+0.5%PEGDE | 1.84 ± 0.02 | 0.07 ± 0.01 | 1.79 ± 0.15 | 14.65 ± 0.14 |
|  |  |  |  |  |
|  |  |  |  |  |
| 14%PEI+1%PEGDE | 2.15 ± 0.15 | 0.07 ± 0.00 | 2.30 ± 0.19 | 16.84 ± 0.44 |
|  |  |  |  |  |
|  |  |  |  |  |
| 14%PEI+1.5%PEGDE | 2.22 ± 0.01 | 0.08 ± 0.01 | 2.82 ± 0.36 | 19.91 ± 0.90 |
|  |  |  |  |  |
|  |  |  |  |  |
| 14%PEI+2%PEGDE | 2.57 ± 0.02 | 0.08 ± 0.01 | 2.91 ± 0.31 | 21.36 ± 0.24 |
|  |  |  |  |  |
|  |  |  |  |  |
| 14%PEI+2.5%PEGDE | 2.10 ± 0.00 | 0.09 ± 0.00 | 2.60 ± 0.23 | 22.07 ± 0.16 |
|  |  |  |  |  |
|  |  |  |  |  |
| 14%PEI+3%PEGDE | 1.83 ± 0.01 | 0.09 ± 0.01 | 2.37 ± 0.33 | 23.63 ± 0.26 |
|  |  |  |  |  |
|  |  |  |  |  |
| 14%PEI+3.5%PEGDE | 2.39 ± 0.19 | 0.09 ± 0.00 | 2.81 ± 0.20 | 18.84 ± 0.83 |
|  |  |  |  |  |
|  |  |  |  |  |
| 14%PEI+4%PEGDE | 2.49 ± 0.02 | 0.08 ± 0.00 | 2.48 ± 0.28 | 19.12 ± 0.40 |
|  |  |  |  |  |
|  |  |  |  |  |
| 14%PEI+4.5%PEGDE | 2.11 ± 0.04 | 0.08 ± 0.00 | 2.43 ± 0.24 | 17.22 ± 1.57 |
|  |  |  |  |  |
|  |  |  |  |  |
| 14%PEI+5%PEGDE | 2.36 ± 0.06 | 0.09 ± 0.01 | 2.78 ± 0.54 | 17.57 ± 0.09 |
|  |  |  |  |  |
|  |  |  |  |  |
| 16%PEI+0%PEGDE | 1.53 ± 0.04 | 0.05 ± 0.00 | 1.19 ± 0.21 | 13.45 ± 0.17 |
|  |  |  |  |  |
|  |  |  |  |  |
| 16%PEI+0.5%PEGDE | 1.67 ± 0.01 | 0.06 ± 0.01 | 1.66 ± 0.21 | 20.82 ± 1.04 |
|  |  |  |  |  |
|  |  |  |  |  |
| 16%PEI+1%PEGDE | 1.65 ± 0.02 | 0.06 ± 0.01 | 1.55 ± 0.26 | 17.71 ± 1.34 |
|  |  |  |  |  |
|  |  |  |  |  |
| 16%PEI+1.5%PEGDE | 1.93 ± 0.07 | 0.05 ± 0.01 | 1.66 ± 0.35 | 33.28 ± 0.45 |
|  |  |  |  |  |
|  |  |  |  |  |
| 16%PEI+2%PEGDE | 2.00 ± 0.05 | 0.06 ± 0.01 | 1.67 ± 0.19 | 20.57 ± 0.14 |
|  |  |  |  |  |
|  |  |  |  |  |
| 16%PEI+2.5%PEGDE | 2.07 ± 0.10 | 0.06 ± 0.01 | 1.94 ± 0.21 | 33.43 ± 0.46 |
|  |  |  |  |  |
|  |  |  |  |  |
| 16%PEI+3%PEGDE | 2.04 ± 0.07 | 0.07 ± 0.01 | 2.35 ± 0.52 | 31.22 ± 2.97 |
|  |  |  |  |  |
|  |  |  |  |  |
| 16%PEI+3.5%PEGDE | 1.55 ± 0.10 | 0.05 ± 0.01 | 1.16 ± 0.41 | 29.76 ± 0.47 |
|  |  |  |  |  |
|  |  |  |  |  |
| 16%PEI+4%PEGDE | 1.94 ± 0.01 | 0.07 ± 0.01 | 2.28 ± 0.44 | 28.09 ± 2.90 |
|  |  |  |  |  |
|  |  |  |  |  |
| 16%PEI+4.5%PEGDE | 2.07 ± 0.15 | 0.04 ± 0.01 | 1.28 ± 0.60 | 25.11 ± 4.25 |
|  |  |  |  |  |
|  |  |  |  |  |
| 16%PEI+5%PEGDE | 2.25 ± 0.05 | 0.06 ± 0.01 | 1.78 ± 0.49 | 19.61 ± 1.10 |
|  |  |  |  |  |
|  |  |  |  |  |
| 18%PEI+0%PEGDE | 1.76 ± 0.10 | 0.08 ± 0.01 | 2.23 ± 0.61 | 18.82 ± 4.96 |
|  |  |  |  |  |
|  |  |  |  |  |
| 18%PEI+0.5%PEGDE | 1.85 ± 0.07 | 0.09 ± 0.02 | 2.76 ± 0.45 | 23.17 ± 1.73 |
|  |  |  |  |  |
|  |  |  |  |  |
| 18%PEI+1%PEGDE | 2.22 ± 0.08 | 0.12 ± 0.02 | 3.67 ± 0.54 | 14.78 ± 4.07 |
|  |  |  |  |  |
|  |  |  |  |  |
| 18%PEI+1.5%PEGDE | 1.68 ± 0.03 | 0.12 ± 0.01 | 2.95 ± 0.26 | 13.18 ± 3.14 |
|  |  |  |  |  |
|  |  |  |  |  |
| 18%PEI+2%PEGDE | 1.64 ± 0.02 | 0.11 ± 0.01 | 2.78 ± 0.47 | 10.24 ± 2.39 |
|  |  |  |  |  |
|  |  |  |  |  |
| 18%PEI+2.5%PEGDE | 2.07 ± 0.10 | 0.06 ± 0.01 | 1.94 ± 0.21 | 30.36 ± 3.34 |
|  |  |  |  |  |
|  |  |  |  |  |
| 18%PEI+3%PEGDE | 1.53 ± 0.10 | 0.10 ± 0.01 | 2.21 ± 0.61 | 10.55 ± 1.01 |
|  |  |  |  |  |
|  |  |  |  |  |
| 18%PEI+3.5%PEGDE | 1.80 ± 0.13 | 0.10 ± 0.01 | 2.42 ± 0.50 | 16.40 ± 0.40 |
|  |  |  |  |  |
|  |  |  |  |  |
| 18%PEI+4%PEGDE | 1.55 ± 0.09 | 0.08 ± 0.01 | 1.69 ± 0.15 | 13.73 ± 2.33 |
|  |  |  |  |  |
|  |  |  |  |  |
| 18%PEI+4.5%PEGDE | 2.38 ± 0.06 | 0.09 ± 0.01 | 2.90 ± 0.11 | 18.92 ± 0.80 |
|  |  |  |  |  |
|  |  |  |  |  |
| 18%PEI+5%PEGDE | 2.68 ± 0.21 | 0.06 ± 0.00 | 1.74 ± 0.30 | 23.02 ± 0.75 |
|  |  |  |  |  |
|  |  |  |  |  |
| 20%PEI+0%PEGDE | 1.25 ± 0.04 | 0.15 ± 0.00 | 2.50 ± 0.07 | 7.31 ± 0.53 |
|  |  |  |  |  |
|  |  |  |  |  |
| 20%PEI+0.5%PEGDE | 1.92 ± 0.08 | 0.14 ± 0.02 | 3.36 ± 0.34 | 8.09 ± 0.87 |
|  |  |  |  |  |
|  |  |  |  |  |
| 20%PEI+1%PEGDE | 1.63 ± 0.01 | 0.14 ± 0.01 | 2.86 ± 0.13 | 9.38 ± 1.08 |
|  |  |  |  |  |
|  |  |  |  |  |
| 20%PEI+1.5%PEGDE | 1.84 ± 0.03 | 0.12 ± 0.01 | 2.79 ± 0.26 | 14.26 ± 1.16 |
|  |  |  |  |  |
|  |  |  |  |  |
| 20%PEI+2%PEGDE | 2.68 ± 0.04 | 0.16 ± 0.00 | 3.34 ± 0.55 | 15.50 ± 0.60 |
|  |  |  |  |  |
|  |  |  |  |  |
| 20%PEI+2.5%PEGDE | 2.76 ± 0.02 | 0.24 ± 0.02 | 4.49 ± 0.31 | 16.43 ± 0.30 |
|  |  |  |  |  |
|  |  |  |  |  |
| 20%PEI+3%PEGDE | 3.07 ± 0.01 | 0.27 ± 0.02 | 5.37 ± 0.12 | 18.95 ± 0.33 |
|  |  |  |  |  |
|  |  |  |  |  |
| 20%PEI+3.5%PEGDE | 3.23 ± 0.01 | 0.25 ± 0.01 | 5.19 ± 0.23 | 22.15 ± 0.14 |
|  |  |  |  |  |
|  |  |  |  |  |
| 20%PEI+4%PEGDE | 3.48 ± 0.02 | 0.22 ± 0.02 | 5.86 ± 0.17 | 27.94 ± 0.39 |
|  |  |  |  |  |
|  |  |  |  |  |
| 20%PEI+4.5%PEGDE | 3.89 ± 0.01 | 0.22 ± 0.04 | 5.91 ± 0.24 | 31.48 ± 0.39 |
|  |  |  |  |  |
|  |  |  |  |  |
| 20%PEI+5%PEGDE | 4.16 ± 0.02 | 0.24 ± 0.00 | 6.07 ± 0.13 | 33.64 ± 0.05 |
|  |  |  |  |  |
|  |  |  |  |  |

Table.S4 Relative intensity of band 1162 cm^-1^ versus band 3073 cm^-1^ of the FT-IR spectra of the original SF, the RPSF, and the PEI/PPEGDE/IPP treated SF

| Sample | I_3073_ | I_1162_ | Relative Intensity |
| --- | --- | --- | --- |
| Original SF | 101.45 | 96.54 | 0.956 |
| Simulated RPSF | 99.135 | 98.03 | 0.988 |
| PEI/PPEGDE/IPP treated SF | 80.72 | 73.92 | 0901 |

Fig.S245 Tearing tests of original silk fabric (3 replicates)

Fig.S246 Tearing tests of rotten/pulverized silk fabric (3 replicates)

Fig.S247 Tearing tests of rotten/pulverized silk fabric treated with 2%PEI+2%PPEGDE+1%IPP (3 replicates)

Fig.S248 Tearing resistance test of rotten/pulverized silk fabric treated with IPP (3 replicates)

Fig.S249 EDS and EDS mapping of original SF, RPSF, and the PEI/PEGDE/IPP treated SF
